# Do the Historical Biogeography and Evolutionary History of the Digenean *Margotrema* spp. across Central Mexico Mirror Those of Their Freshwater Fish Hosts (Goodeinae)?

**DOI:** 10.1371/journal.pone.0101700

**Published:** 2014-07-07

**Authors:** Andrés Martínez-Aquino, Fadia Sara Ceccarelli, Luis E. Eguiarte, Ella Vázquez-Domínguez, Gerardo Pérez-Ponce de León

**Affiliations:** 1 Posgrado en Ciencias Biológicas, Universidad Nacional Autónoma de México, México, D.F., México; 2 Departamento de Zoología, Instituto de Biología, Universidad Nacional Autónoma de México, México, D.F., México; 3 División de Aracnología, Museo Argentino de Ciencias Naturales “Bernardino Rivadavia”, Buenos Aires, Argentina; 4 Departamento de Ecología Evolutiva, Instituto de Ecología, Universidad Nacional Autónoma de México, México, D.F., México; 5 Departamento de Ecología de la Biodiversidad, Instituto de Ecología, Universidad Nacional Autónoma de México, México, D.F., México; Consiglio Nazionale delle Ricerche (CNR), Italy

## Abstract

Host-parasite systems provide an ideal platform to study evolution at different levels, including codivergence in a historical biogeography context. In this study we aim to describe biogeographic and codivergent patterns and associated processes of the Goodeinae freshwater fish and their digenean parasite (*Margotrema* spp.) over the last 6.5 Ma (million years), identifying the main factors (host and/or hydrogeomorphology) that influenced the evolution of *Margotrema*. We obtained a species tree for *Margotrema* spp. using DNA sequence data from mitochondrial and nuclear molecular markers (*COI* and ITS1, respectively) and performed molecular dating to discern divergence events within the genus. The dispersal-extinction-cladogenesis (DEC) model was used to describe the historical biogeography of digeneans and applied to cophylogenetic analyses of *Margotrema* and their goodeine hosts. Our results showed that the evolutionary history of *Margotrema* has been shaped in close association with its geographic context, especially with the geological history of central Mexico during the Pleistocene. Host-specificity has been established at three levels of historical association: a) *Species-Species*, represented by *Xenotaenia resolanae*-*M. resolanae* exclusively found in the Cuzalapa River Basin; b) *Species-Lineage*, represented by *Characodon audax*-*M. bravoae* Lineage II, exclusive to the Upper and Middle Mezquital River Basin, and c) *Tribe-Lineage*, including two instances of historical associations among parasites and hosts at the taxonomical level of tribe, one represented by Ilyodontini-*M. bravoae* Lineage I (distributed across the Ayuquila and Balsas River Basins), and another comprised of Girardinichthyini/Chapalichthyini-*M. bravoae* Lineage III, found only in the Lerma River Basin. We show that the evolutionary history of the parasites is, on several occasions, in agreement with the phylogenetic and biogeographic history of their hosts. A series of biogeographic and host-parasite events explain the codivergence patterns observed, in which cospeciation and colonisation via host-switching and vicariant plus dispersal events are appreciated, at different times during the diversification history of both associates, particularly during the Pleistocene.

## Introduction

Host-parasite associations represent exceptional systems for linking evolution and ecology to obtain a broader view of how biotic interactions shape life on earth. Evolutionary studies of host-parasite systems vary in their approaches and outcomes, ranging from very close evolutionary associations between hosts and their parasites, to the apparent lack of such evolutionary associations. In some cases for example, the ability of the parasite to colonise new hosts plays a greater role in its diversification process than coevolution with the host, resulting in highly incongruent phylogenies, as shown in some host-parasite systems such as pinnipeds and their cestode parasites [1], [2]. On the other hand, strict cospeciation was found between pocket gophers and their chewing lice [3]. As host-parasite systems are wide and varied, a broad spectrum of factors influences the associates' evolution (for reviews on this topic see [4], [5]). The study of a host-parasite association, in terms of the evolution of parasitic organisms, usually focuses on evaluating the geographic distribution and the phylogenetic relationships of the hosts. These two elements can be analysed by probabilistic methods in biogeography [6], as well as through evolutionary tangled trees of hosts and parasites [7]. In turn, hypotheses of evolutionary history and biogeography can be explicitly tested in time and space using parametric biogeography methods [8], [9], [10] coupled with dated species-tree estimation [11] and by contrasting the phylogenetic relationships between two groups that have a narrow biological association in a particular geographic area [10], [12], [13]. Previous studies have shown that geographic features may significantly shape genealogical relationships of hosts and parasites by causing co-differentiation between parasitic organisms and the evolutionary history of their hosts [14]. However, it is difficult to find a biological model of parasitism capable of explaining the process of diversification between hosts and parasites, as outlined by Caira and Jensen [15] and Althoff et al. [16]. The biogeographic “core” parasite fauna, i.e. widely distributed species characteristically associated with - and restricted to - a monophyletic group of host species (see [17]), offer a unique opportunity to test diversification processes between hosts and their parasites.

Central Mexico, and particularly the Trans-Mexican Volcanic Belt (TMVB), has been used to explain diversification because it represents a transition zone between the Neotropical and Nearctic biogeographic regions [18]. The TMVB is considered an area of endemism for different taxa, mainly as a result of its complex hydro-geomorphological history. It is a remarkable area of endemism for the freshwater fish fauna [19]. Goodeines, endemic elements of the central Mexican freshwater fish fauna, are a monophyletic group of cyprinodontiforms that experienced a remarkable diversification in this area ([20] and references therein). The digenean genus *Margotrema* Lamothe-Argumedo, 1972 encompasses species that are relatively common intestinal parasites of goodeines across Mexico [21]. Their members are part of the biogeographic “core” helminth fauna of goodeines [17]. Based on the fact that the evolutionary and biogeographic history of goodeines was deeply influenced by tectovolcanic activity in central Mexico, Martínez-Aquino et al. [21] recently deciphered the genealogical structure of *Margotrema*. The genus contains *M. resolanae* Pérez-Ponce de León, Martínez-Aquino and Mendoza-Garfias, 2013 and three independent genetic lineages within *M. bravoae* Lamothe-Argumedo, 1972. These authors also demonstrated that the parasite is tightly associated with the genus' geographic distribution across different hydrological systems in this region. In addition, the three *M. bravoae* lineages apparently show certain specific association with their hosts, at the goodeine taxonomic level of tribe, representing monophyletic groups. These results support the hypothesis that the distribution patterns and the host associations of the four *Margotrema* lineages were concordant with the hydro-geomorphologic events occurring in central Mexico. Also, that the vicariant and dispersal events associated with the goodeine diversification promoted at the same time the diversification of the *Margotrema* lineages. In this context, two patterns of evolution of *Margotrema* were uncovered: restricted geographic distribution in hydrological systems and host-specificity at the host-species and host-tribe levels [21].

In this study, we explored the evolutionary processes that drove the diversification of a host-parasite association, based on biogeographic and phylogenetic hypotheses of goodeine fishes [20], [22] and the phylogenetic relationships of the parasite *Margotrema* spp. We also examined the potential role that their respective geographic distributions had on each other's evolutionary history. In this context, we established whether the evolution of *Margotrema* spp. was influenced by the complex geographic scenario of central Mexico, by their close association with their goodeine hosts, or by a combination of the two. Therefore, we test the general null hypothesis that the parasite phylogeny is independent of the host phylogeny [23]. Consequently, the main alternative hypothesis is that the biogeographic congruence between the genealogical history of the *Margotrema* lineages and the hydro-geomorphological history of central Mexico is similar to the historical biogeography of their goodein hosts. If this main hypothesis is supported, the following two specific hypotheses can be tested: 1) the biogeographic congruence is further reflected in the evolutionary histories of the Goodeinae tribes and the associated lineages of *Margotrema* and 2) the divergence times of the main clades of Goodeinae and those of *Margotrema* are relatively similar.

## Materials and Methods

### 1. Taxa, molecular dataset and phylogenetic analyses

The present study is a follow-up to a previous work in which Bayesian phylogenetic inference was used to reconstruct phylogenies of 127 individuals belonging to the genus *Margotrema* allowing us to establish topological congruence between various programs and algorithms (for more details see [21]). For the present phylogenetic analyses the same taxa and gene sequences as in the previous study were used: samples of *Margotrema* spp. and published fragments of Cytochrome *c* Oxidase subunit I (*COI*; mitochondrial DNA) and Internal Transcribed Spacer 1 (ITS1; nuclear DNA), encompassing 750 and 831 base pairs including gaps, respectively [21]. We constructed a combined dataset to perform a multispecies coalescent analysis as implemented in *BEAST v1.7.4 [24] to obtain a species tree to infer the genealogical relationship between *M. resolanae* and the three *M. bravoae* lineages [21]. The aligned data file in nexus format was deposited in the Dryad Digital Repository (DRYAD) [25], DOI: 10.5061/dryad.bq7q0.

### 2. Divergence dating

To determine an accurate time frame for phylogenetic divergence processes for each *Margotrema* lineage (see Table 1), we estimated mean node ages and their 95% highest posterior densities (HPDs) using a Bayesian relaxed molecular clock method [26] implemented in *BEAST. In this framework, tests of evolutionary hypotheses are not conditioned to a single tree topology, allowing for the simultaneous evaluation of topology and divergence times, while incorporating their uncertainty. Heterozygote sites in the nuclear gene fragment were identified using “Find Heterozygotes Plugin” in Geneiuos Pro v5.1.7 [27], using a threshold of 90% peak heights. We applied the same optimal model as the one obtained by Martínez-Aquino et al. [21]: *COI* with HKY+I+G and ITS1 with HKY+G invariant sites with *BEAST package (BEAUti v1.7.4; [24]). An uncorrelated relaxed log-normal molecular clock was applied to model rate variation across branches and the uniform Yule tree prior was chosen, appropriate for hierarchical rather than reticulate relationships. In absence of specific information on substitution rates of the gene fragments for our species, we applied lognormal distributions with a mean of 1 and standard deviation of 0.33 to both markers, allowing for auto-optimizations as the runs progressed. Based on the geographic distribution of *Margotrema* (Martínez-Aquino et al. unpublished data), we applied a geological calibration based on the uplifting of the western zone of the TMVB, which started around 11 million years ago (Ma) [28], [29]. This age was set as a maximum for the Most Recent Common Ancestor (MRCA) of *Margotrema* spp. Monophyly was not enforced for any of the other nodes. Each terminal of the species tree was set to incorporate the individuals from one lineage found in one of the 12 areas chosen (see below for more details). Two separate analyses were run for 50 million generations each with a sampling frequency of one in every 1000 generations and outputs were combined using LogCombiner v. 1.7.4 [24]. Tracer v. 1.5 (available online from http://beast.bio.ed.ac.uk/) was used to assess convergence of the model parameters, where an effective sample size (ESS) value >200 was considered adequate. Branch support for the different tree topologies was evaluated by Posterior Probability (PP) values for nodal support (PP>0.95). The tree with the highest clade probability was chosen from the *BEAST output files using the program TreeAnnotator v.1.7 [24]. The *BEAST phylogenetic reconstructions were run through the CIPRES Science Gateway V. 3.3 [30]. The *BEAST.xml input file was deposited in DRYAD DOI: 10.5061/dryad.bq7q0.

**Table 1:** Parasite-host-area associations of *Margotrema* species and lineages used in this study.

Parasite species (lineages)	Host species (code)	Area (sub-basin) (code)	Hydrological systems
*Margotrema resolanae*	*Xenotaenia resolanae* (m)	Cuzalapa River (I)	Cuzalapa River
*Margotrema bravoae* Lineage I	*Codoma ornata* (h)	Lower Conchos River (A)	Conchos River Basin
*Margotrema bravoae* Lineage I	*Allodontichthys zonistius* (g) *Ilyodon furcidens* (k)	Armería-Ayuquila River (H)	Ayuquila River
*Margotrema bravoae* Lineage I	*Chapalichthys pardalis* (j) *Ilyodon whitei* (l)	Lower Balsas River (K)	Balsas River Basin
*Margotrema bravoae* Lineage I	*Ilyodon whitei* (l)	Upper Balsas River (L)	Balsas River Basin
*Margotrema bravoae* Lineage II	*Characodon audax* (i)	Upper and Middle Mezquital River (B)	Mezquital River Basin
*Margotrema bravoae* Lineage III	*Zoogoneticus purhepechus* (o)	Lower Lerma River (C)	Lerma River Basin
*Margotrema bravoae* Lineage III	*Allotoca zacapuensis* (f) *Zoogoneticus quitzeoensis* (n)	Zacapu Lake (D)	Lerma River Basin
*Margotrema bravoae* Lineage III	*Alloophorus robustus* (e) *Zoogoneticus quitzeoensis* (n)	Cuitzeo Lake (E)	Lerma River Basin
*Margotrema bravoae* Lineage III	*Allotoca diazi* (a) *Allotoca duguesi* (b)	Pátzcuaro Lake (F)	Lerma River Basin
*Margotrema bravoae* Lineage III	*Allotoca meeki* (c)	Zirahuén Lake (G)	Lerma River Basin
*Margotrema bravoae* Lineage III	*Neoophorus regalis* (d)	Cotija (J)	Lerma River Basin

Letters in brackets next to host and area names correspond to the codes used in the biogeographic and cophylogenetic analyses.

### 3. Historical biogeography

**Figure 1:**
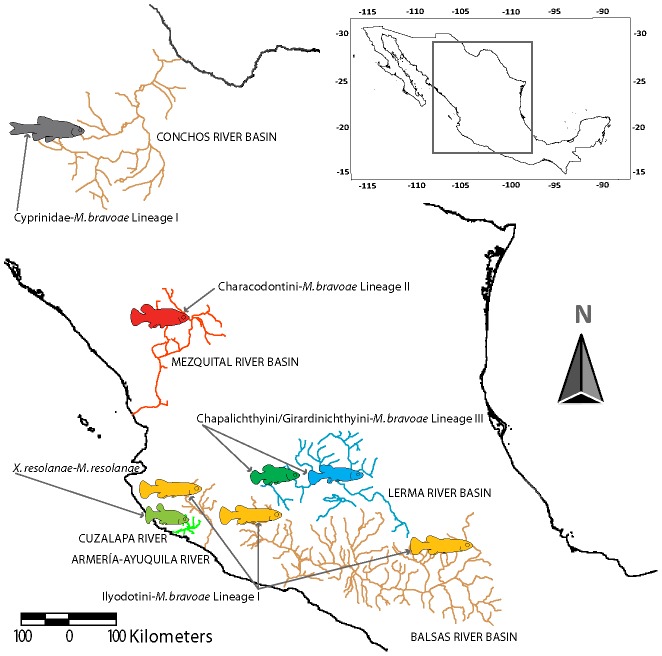
Pattern of geographic distribution of *Margotrema* spp. and their associated goodeins from central Mexico. Hydrological systems in colours correspond to distributions of each lineage of *Margotrema* spp. collected in this study: green  =  *Margotrema resolanae*; orange  =  *Margotrema bravoae* Linaege I; blue  =  *Margotrema bravoae* Linaege II; red  =  *Margotrema bravoae* Linaege III. Coloured fish outlines correspond to the family/tribe(s)/species associated with each *Margotrema* lineage: grey fish  =  Cyprinidae (in *Codoma ornata*); red fish  =  Characodontini (in *Characodon audax*); yellow fish  =  Ilyodontini; blue fish  =  Chapalichthyini and Girardinichthyini; green fish  =  *Xenotaenia resolanae* (of the tribe Ilyodontini).

To uncover the events that influenced the historical biogeography and diversification processes of *Margotrema* spp., we applied dispersal–extinction–cladogenesis models (DEC) using the program Lagrange v. 20130526 [8], [9] with the dated ultrametric area tree obtained from *BEAST. We coded the geographic distribution of the terminal taxa into 12 different areas, which were used to recover the main clades within *Margotrema* spp. (see [21]), following the area codes used by Domínguez-Domínguez et al. [20], [22] (Table 1; Fig. 1; Fig. S1). The 12 areas were delimited based on the Mexican hydrological basins and sub-basins map produced by CONABIO (www.conabio.gob.mx). All maps shown in this study were modified in DIVA-GIS 7.5 from sources ([31] freely available through www.diva-gis.org). The DEC algorithm permits uncovering dispersal and extinction events along branches of a phylogeny, while estimating the ranges of the MRCA and descendent species at each node, returning maximum likelihood (ML) and relative probability values for each area/event as well as providing a global ML value for the total analysis. This value allows for comparisons of different runs incorporating varying degrees of spatio-temporal constraints. Accordingly, we compared three models varying in maximum number of ancestral areas and dispersal probability constraints, keeping the same adjacency matrix for all three runs. Dispersal probability constraints were set to consider the presence of the central Mexican Palaeolakes and thus increased dispersal probabilities between the areas included during the Pleistocene. The input files for the analyses were constructed using the web-based *Lagrange configurator* (http://www.reelab.net/lagrange/configurator/index) and are available from DRYAD; DOI: 10.5061/dryad.bq7q0.

### 4. Cophylogenetic analyses

To test evolutionary associations such as codivergence between goodeine fishes and their *Margotrema* parasites, we implemented a DEC model using Lagrange. Trees involving the association of two taxa were used to infer their common evolutionary history, both in a geographic (area and organism trees) and in a cophylogenetic (host and parasite) scenario. Considering that biogeographic investigations can be paralleled to cophylogenetic ones, the following analogies were used, as study units, in accordance with Page and Charleston [32]: area/host like organism/parasite, dispersal/host-switch, vicariance/cospeciation, sympatric speciation/parasite speciation in one host and extinction/parasite extinction (lineage loss). In this context, we built two matrices of 15 “areas ( =  hosts)” (see Table 1 for codes), in which the maximum “range” size for ancestral areas ( =  host species) was set to two. The dispersal probabilities were constrained using similar premises to those used for the geographic DEC analyses (input files in DRYAD; DOI: 10.5061/dryad.bq7q0) taking into account the genealogical relationships between *Margotrema* spp. and their geographic areas. Also, the following three terms were used in accordance with Charleston [12]. Codivergence, also referred to as cospeciation, implies an event where a parasite lineage (species) infecting a host lineage diverges into two new lineages following the divergence of their host (approximately at the same time). Duplication, an event where the parasite lineage diverges into two new lineages, independently of its host, and both new lineages remain on that host lineage. Host-switching, defined as the event where the parasite diverges by switching from one host to establish in another host lineage. Following Choudhury et al. [33], parasite speciation may either be concomitant with and resulting from host speciation (cospeciation) or follow the colonisation of a new host from an existing one (host-switching).

We also compared the phylogenies of the subfamily Goodeinae and *Margotrema* to establish whether a significant match existed between host and parasite trees. We used a statistic test of *p* values with a 95% of confidence intervals with TreeMap 3b [12], employing the tree topologies obtained from cytochrome *b* gene sequences from the hosts and *COI*+ITS1 sequences from four lineages of *Margotrema* spp. The dated tree from the probabilistic analyses of Goodeinae [20] was obtained from a co-author of the cited study and edited with MESQUITE 2.72 [34], to select terminal taxa that are found in a host-parasite association with *Margotrema*, removing the remaining terminal taxa from the tree. Similarly, the dated molecular phylogeny of *Margotrema* spp. was edited in MESQUITE 2.72, where samples of each locality were trimmed to a single terminal taxon. This was done because TreeMap 3b only reconciles strictly dichotomous trees. The input file for TreeMap is available from DRYAD (DOI: 10.5061/dryad.bq7q0).

## Results

### 1. Divergence times

**Figure 2:**
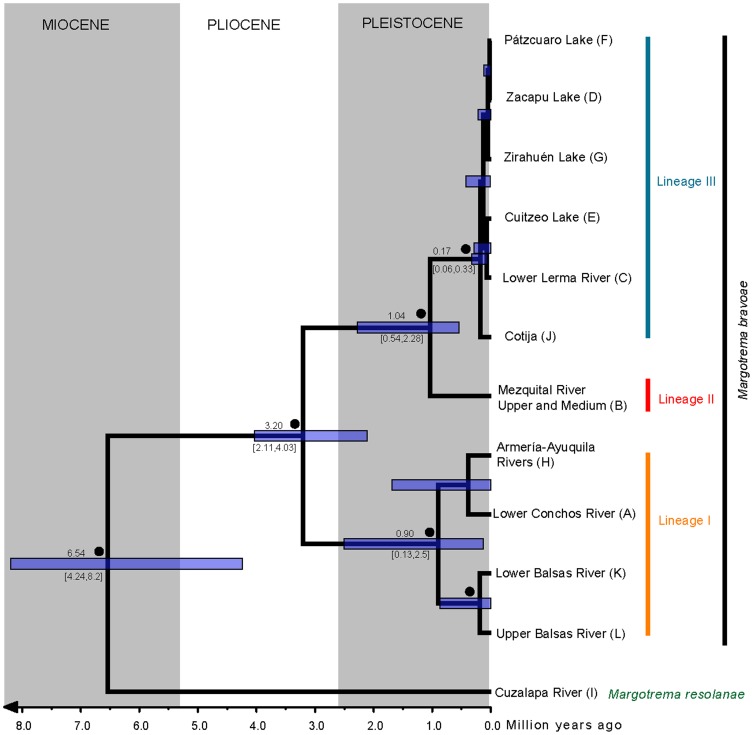
Molecular dating of cladogenetic events. Ultrametric tree derived from *BEAST and the combined dataset showing divergence time estimates of selected clades including 95% confidence intervals (blue bars). Black circles above branches represent Bayesian posterior probability (BPP) values ≥0.95. The terminals (areas/lineages) correspond to codes described in Table 1.

The divergence time estimates for the MRCA of the *M. resolanae + M. bravoae* clade was around 6.53 Ma (Fig. 2). The divergence time between the ancestor of Lineage I and ancestor of Lineages II and III was dated at 3.20 Ma, while Lineages II and III diverged approximately 1.04 Ma.

### 2. Historical Biogeography

**Figure 3:**
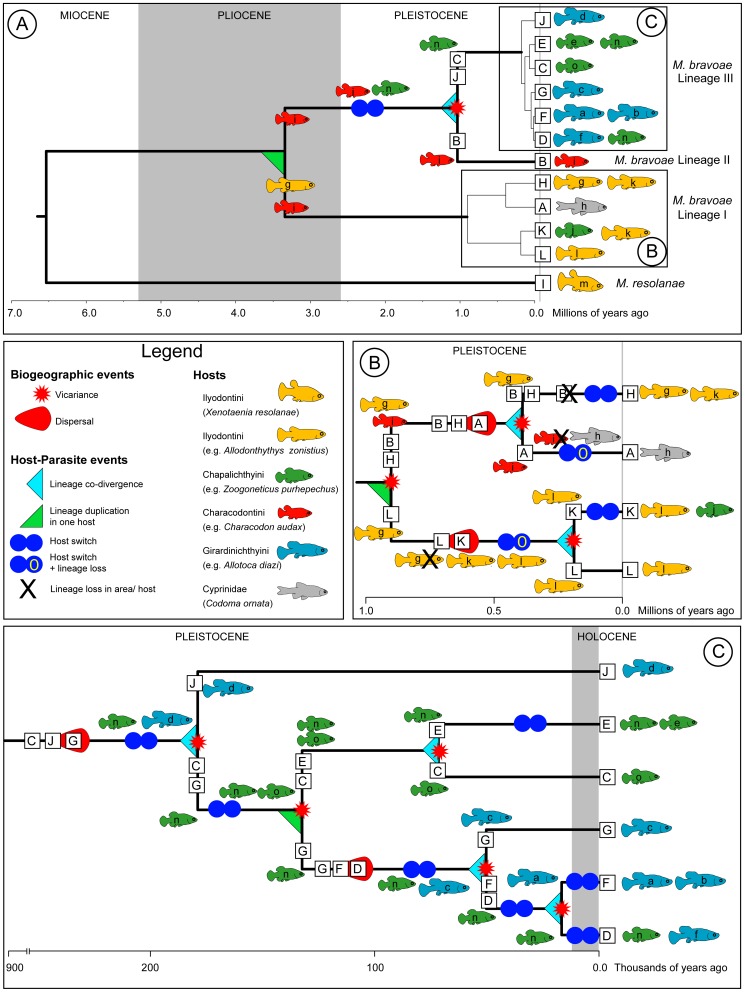
Biogeographic and host-parasite events of the evolution of *Margotrema* spp. from central Mexico associated to Goodeinae. A) Evolution of *M. resolanae* and of each *M. bravoae* lineage. B) Specific events that influenced the evolution of *M. bravoae* Lineage I. C) Specific events that influenced the evolution of *M. bravoae* Lineage III. Squares on branches and at terminal nodes with uppercase letters represent areas and coloured fish shapes with lowercase letters represent current (at terminals) or ancestral (along branches) hosts. The area and host codes correspond to the letters found in Table 1. Additionally, fish shapes are coloured according to their tribes (as in the in-figure Legend). Postulated historical biogeographic and host-parasite events are represented by the different shapes explained in the figure itself.

The ancestral area for *Margotrema bravoae* and *M. resolanae* was recovered with extremely low relative probabilities, making its unequivocal identification almost impossible. This was the case whether or not dispersal probability constraints were enforced in the DEC analyses. Enforcing dispersal constraints increased the global ML of the run, but generally decreased the ML and relative probability of the individual events (see File S1A). For this reason we considered areas and events from all runs as long as they showed combined relative probabilities >0.7 and were in agreement between runs. Overall, low probabilities were also recovered for the ancestral area of *M. bravoae*. On the other hand, the ancestral areas of *M. bravoae* lineages II and III were recovered with relatively high probabilities that support the ancestral area containing the Upper and Middle Mezquital River [B] and a combination of the Cotija area [J] plus one of the areas of the Paleolakes (either the Lower Lerma River [C], Zacapu Lake [D], Cuitzeo Lake [E], Pátzcuaro Lake [F] or Zirahuén Lake [G]). A vicariant event was found separating the MRCA of *M. bravoae* lineages II and III (populations found in area B from the ones found in the remaining ancestral areas) during the Pleistocene, approximately 1.04 Ma (see Figs. 2, 3A and File S1A). The recovered ancestral area of the MRCA of *M. bravoae* Lineage III included a combination of the Cotija area [J] plus two of the areas of the Paleolakes (either the Lower Lerma River [C], Zacapu Lake [D], Cuitzeo Lake [E], Pátzcuaro Lake [F] or Zirahuén Lake [G]; Fig. 3B, only one area shown), with a vicariant event separating the populations in Cotija from the populations of the remaining areas, approximately 170,000 years ago. Subsequent vicariant events, which progressively fragmented the ancestral populations from the areas of present-day central Mexico that once formed part of the greater Paleolakes during the Pleistocene (Figs. 2 and 3B), were uncovered. The ancestral area of *M. bravoae* Lineage I was found to span the Upper and Middle Mezquital River [B], the Armería-Ayuquila River [H] and the Upper Balsas River [L]. Maximum likelihood values lend support to a vicariant event separating the ancestral populations of the Upper Balsas River from those found in areas B and H, with interrupted gene flow causing diversification approximately 900,000 years ago (Figs 2 and 3C).

### 3. Codivergence patterns

The results of our second round of DEC analyses (where we equated parasite-host to species-range) reflect the difficulty of inferring ancestral hosts and events that pre-date the Pleistocene. For the MRCA of *M. bravoae* moderate support (combined relative probabilities >0.5; File S1B) is given to a model of lineage duplication in an ancestral host during the Pliocene, postulated to have been a member of the tribe Characodontini (i), while also being present in a host of the Ilyodontini tribe (g). During the Plio-Pleistocene boundary, a host-switch from the Characodontini (i) to Chapalichthyini (n) occurred in the MRCA of *M. bravoae* Lineages II and III, followed by the MRCA's divergence (approx. 1.04 Ma), as a result of a vicariant event separating the ancestral populations of *C. audax* (i) in the Mezquital River from the other ancestor that colonised Chapalychthyini (n) in the Lerma River (Fig. 3A). Particularly, in *M. bravoae* Lineage III, the ancestor underwent colonisation via host-switching from *Zoogoneticus quitzeoensis* (n) to *Neoophorus regalis* (d) in the Lerma River Basin (Fig. 3B; between 900,000 and 200,000 years ago). Subsequently, other host-switching events occurred once the ancestor was in that particular geographic area. For instance, a host-switching event allowed the colonisation of *Alloophorus robustus* (e), while another five host-switching events permitted the colonisation of *A. diazi* (a), *A. duguesi* (b), *Allotoca meeki* (c), *A. zacapuensis* (f) and *Z. purhepechus* (o) (Fig. 3B; from 0.20 Ma to the present).

In the case of the MRCA of *M. bravoae* Lineage I, we found an association with postulated ancestors of *A. zonistius* (g) + *C. audax* (i) (since 3.20 Ma, Fig. 3A). Initial lineage duplication (in g) allowed the diversification of *M. bravoae* Lineage I, with subsequent colonisations of several host species belonging to the Ilyodontini, i.e. in *A*. *zonistius* (g), *I*. *furcidens* (k) and *I. withei* (l) (since 1.03 Ma, Fig. 3C). Of particular interest is the history of the present-day *M. bravoae* Lineage I found *Codoma ornata* (h) (host species of the family Cyprinidae), where the ancestral population originated in an ancestor of *C. audax* (i). However, this lineage was lost in this host following a host-switch to *C. ornata* in the Lower Conchos River (area A) (Fig. 3C).

**Figure 4:**
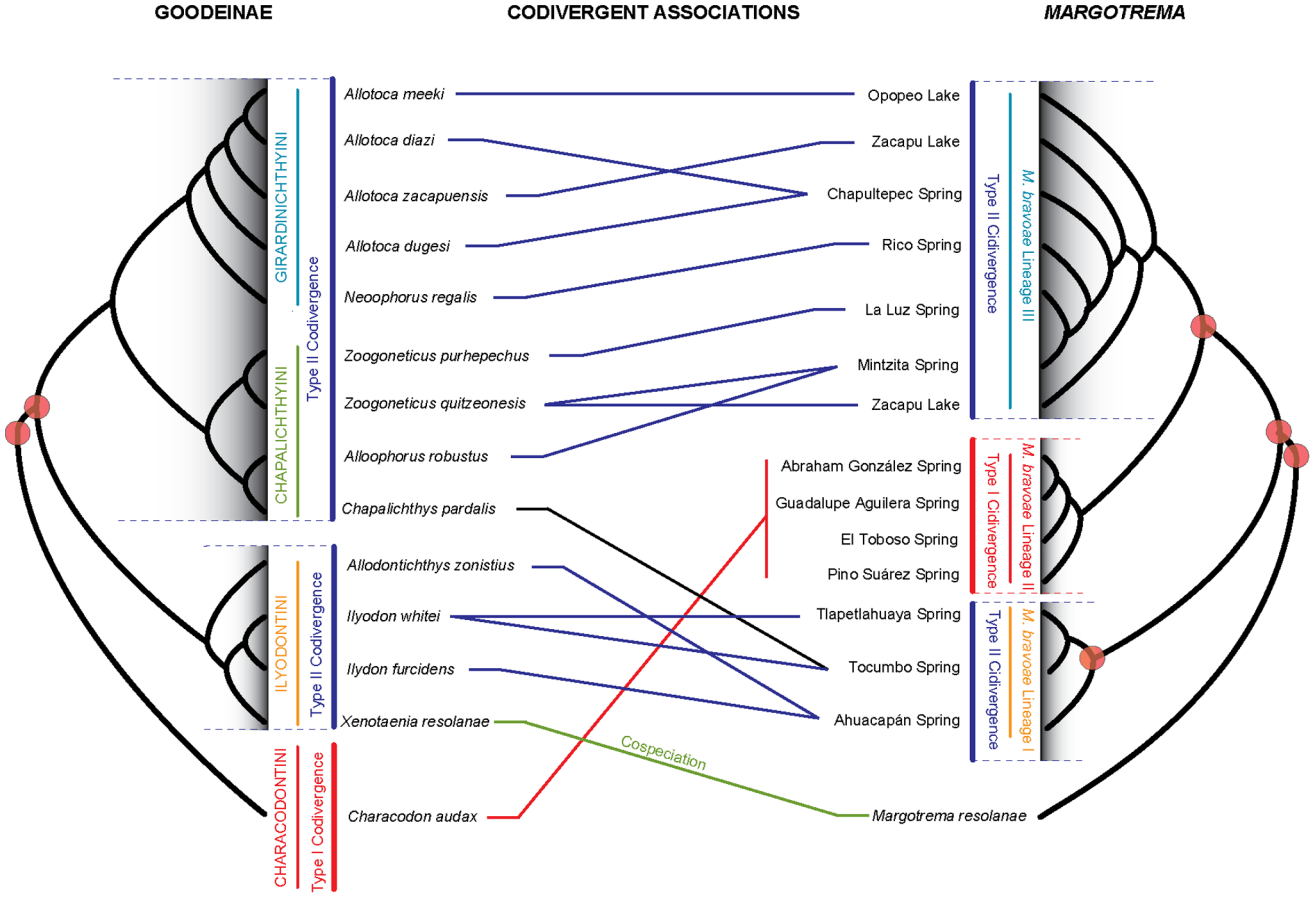
Tanglegram depicting the three levels of codivergent associations between Goodeinae and *Margotrema*. Level 1: *Species-Species*, representing the association *Xenotaenia resolanae*-*Margotrema resolanae* (green line). Level 2: *Species-Lineage*, representing the association *Characodon audax*-*Margotrema bravoae* Lineage II (red line). Level 3: *Tribe-Lineage*, representing the associations between Ilyodontini-*M. bravoae* Lineage I (blue lines) and Girardinichthyini/Chapalichthyini-*M. bravoae* Lineage III (blue lines). Red circles at nodes represent statistically supported codivergence events established by cophylogenetic analysis in TreeMap.

Finally, codivergent events between Goodeinae and *Margotrema*, as found when comparing of the phylogenetic trees of both associates in TreeMap, showed the following three hierarchical levels of codivergence (Fig. 4; also see Fig. 1). Level 1 *Species-Species*: this level represents *strict cospeciation* between *X. resolanae* and *M. resolanae* in the Cuzalapa River; Level 2 *Species-Lineage*: a host species exhibits a close association with a parasite lineage, in this case represented by *M. bravoae* Lineage II parasitising *C. audax* in the Upper and Middle Mezquital River. This level is herein referred to as *Type I Codivergence*, in which the divergence of a parasite lineage occurs in response to the speciation event of its host. Level 3 *Tribe-Lineage*: a monophyletic group of hosts (tribe in this case) exhibits a close association with a parasite lineage. This is represented by *M. bravoae* Lineage I parasitising members of the tribe Ilyodontyni, distributed in several water bodies of the Balsas River Basin, the Ayuquila River and the Lower Conchos River; and by *M. bravoae* Lineage III parasitising tribes Chapalichthyni and Girardinichthyni, in several water bodies exclusive to the Lerma River Basin. This level is herein referred to as *Type II Codivergence.* This codivergence occurs at deeper levels of the phylogenetic history of the hosts (e.g. tribe). In other words, the divergence of the parasite lineage is a result of the diversification process of its host, and subsequent colonisation (host-switching events) of parasite lineages to new hosts strictly belonging to the same tribe (i.e. with close phylogenetic affinities). This may be viewed as vertical transmission *sensu lato* when considering the host tribe as a phylogenetic unit.

## Discussion

The results of the biogeographic analyses reflect the difficulty of making pre-Pleistocene inferences of ancestral areas and events shaping the present distribution of *Margotrema* and their hosts since the divergence of the MRCA of the genus *Margotrema*, ca. 6.53 Ma. Rather than a methodological constraint, this difficulty relates to the fact that we were unable to include the sister group of *Margotrema* in our analyses (see below). This is further complicated by our limited understanding of geological events that took place in central Mexico during the past 7 million years, following a pattern of complex reticulation, i.e. biogeographic scenarios in which areas and biota underwent several events of fragmentation, fusion, and re-fragmentation in iterative cycles of dispersal and vicariance [35]. Similar problems are observed with inferences made regarding the ancestral host at the time of the diversification of the MRCA of *Margotrema*. When discussing ancestral areas in a freshwater fishes-helminth parasite system it is important to keep in mind that the hydrological configuration most likely was very different during past geological epochs (akin to Sanmartín's [35] “range evolution”), even though the ancestral areas may have spanned similar geographic spaces as today. The same applies to the host species, where the present-day species are not the same as the “ancestral” hosts. For these reasons, when talking about ancestral areas and hosts, events and areas/hosts receive increasingly higher support values as the reconstructions approach the present. We will therefore discuss the history of each species and lineage of *Margotrema* in turn, starting from the most derived lineage and moving as far into the past as the statistically supported reconstructions allow.

### 1. Patterns of regional codivergence of Goodeinae*-Margotrema bravoae* across central Mexico

#### 1.1. Girardinichthyini/Chapalichthyini-*Margotrema bravoae* Lineage III: a Pleistocenic model

**Figure 5:**
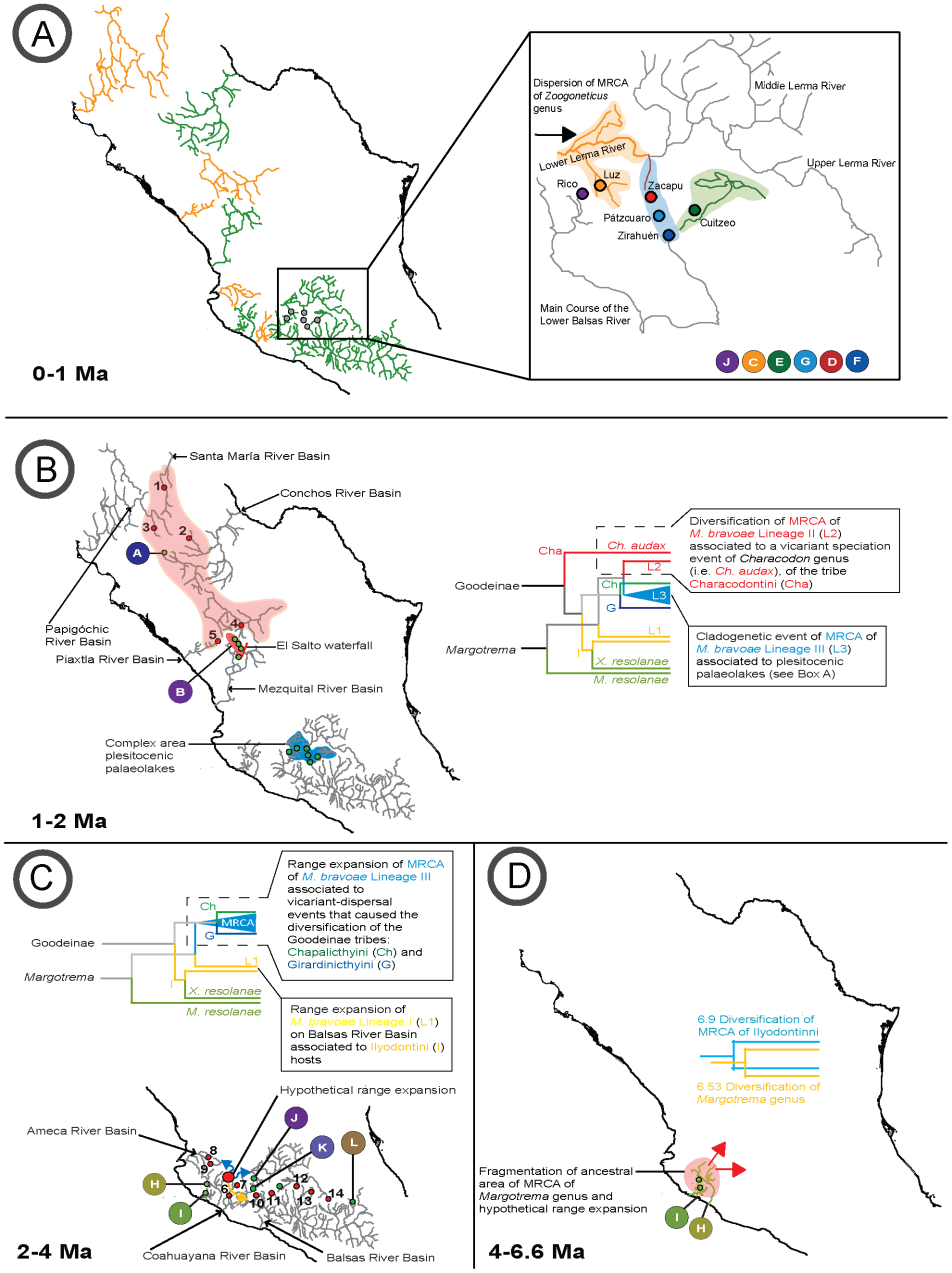
Biogeographic history, codivergence patterns and evolution of *Margotrema* spp. in central Mexico. Hydrological systems in green and orange in Figure 5A correspond to areas where *Margotrema* were collected in this study and previous records, respectively. Full green circles with a capital letter correspond to localities analysed in this study; full red circles with a number indicate *Margotrema* records not collected in this study (Figure 5B; for more details see Table S1). The evolutionary history of *Margotrema* is interpreted as follows. A) Hypothetical area of the Pleistocenic palaeolakes where *M. bravoae* Lineage III is distributed; the orange, blue and green shadows correspond to the phylogenetic relationships of areas. B) Geographic scenario of the diversification of *M. bravoae* Lineage II associated to a vicariant event (El Salto waterfall) that caused the diversification of *Characodon audax* and range expansion of MRCA of *M. bravoae* Lineage III (L3) associated to a complex area of Pleistocenic palaeolakes. The area shaded in red represents a hypothetical ancestral area of *Margotrema* potentially associated to records of *Margotrema* from northern Mexico and the distribution area of fossil records of species of Goodeidae. C) Range expansion of *M. bravoae* Lineage I (L1) along the Balsas River Basin, associated to the biogeographic history of the Ilyodontini [I] tribe; and range expansion of the MRCA of *M. bravoae* Lineage III (L3) associated to vicariant-dispersal events that caused the diversification of the Chapalichthyini and Girardinichthyini tribes. The yellow arrow refers to the dispersal of L1 influenced by the dispersal of its ilyodontini hosts; dark grey arrows refer to dispersal of L3 influenced by the historical patterns of distribution of the chapalichthyines and girardinichthyines. D) Fragmentation of the ancestral area of the most recent common ancestor (MRCA) of *Margotrema* spp. (area shaded in red with red arrows). Diversification process of the MRCA of the tribe Ilyodontini and *Margotrema*, respectively, influenced by vicariant-dispersal events in Cuzalapa River (I) and Armería-Ayuquila River (H).

Our results indicate that the MCRA of *M. bravoae* Lineages II and III was associated with the ancestor of the Characodontini and Chapalychthyini in the Upper and Middle Mezquital and the Cotija and Lower Lerma rivers between 3.2 and 1.03 Ma. The divergence of Lineages II and III was recovered as a vicariant geological event with lineage codivergence leaving *Margotrema* Lineage III associated with an ancestral Chapalychthyini. The subsequent diversification of Lineage III took place between 1 million and 167,000 years ago (Fig. 2), which corresponds with mid- to late Pleistocenic events. The results of the DEC model support the idea of the dispersal of Lineage III during the Pleistocene across hydrological systems of the Lower Lerma River and the Cuitzeo and Zirahuén lakes; apparently, the dispersal and vicariant events occurred in concordance with the diversification processes of their hosts, in turn driven by postulated Pleistocenic events such as the fragmentation of the central Mexican Paleolakes due to a combination of volcanic activity and Paleoclimatic changes [20], [36]. Two area relationships are recovered from this model, the first one between the Zacapu, Pátzcuaro and Zirahuén lakes, which further support the idea of ancestral connections between these water bodies [20], [22] (Fig. 5A). The second, the Cuitzeo Lake and the Lower Lerma River, supports the ancestral connection of this river basin. These area relationships are also supported by the current distribution patterns of several freshwater fish taxa, an idea originally proposed by Álvarez del Villar [37] and more recently by particular phylogenetic and biogeographic analyses [20], [36], [38].

#### 1.2. Characodon audax-Margotrema bravoae Lineage II

As mentioned earlier, lineage codivergence of *M. bravoae* Lineages II and III- Characodontini and Chapalychthyini around 1 Ma left *Margotrema* Lineage II associated with an ancestor of *C. audax*. The presence of the ancestor of the Characodontini in Cotija and the Lower Lerma River is incongruent with the current distribution patterns established for the genus *Characodon*. Nevertheless, the phylogenetic history of goodeines shows that the formation of the Salto waterfall dated 1.8 Ma represents a vicariant event that caused the allopatric speciation of *C. audax* and *C. lateralis* [20], [22] (Fig. 5B). However, no records of *Margotrema* spp. have been established for *C. lateralis*, even though several surveys have been carried out in the area (Martínez-Aquino *et al*. unpublished data). Apparently, the codiverence pattern uncovered in this study, related with the association of *C. audax* and *M. bravoae* Lineage II (exclusive to the Upper and Middle Mezquital river), can be explained by an extinction event of ancestral populations of the Lineage II in the Lower Mezquital River, following the event of cladogenesis in the host caused by the Salto waterfall as a geographic barrier.

#### 1.3. Ilyodontini-*Margotrema bravoae* Lineage I

The differentiation process of the MRCA of *M. bravoae* Lineage I from Lineages II and III was dated at 3.2 Ma. The MRCA of Lineage I was found to have diversified approximately 900,000 years ago in the areas that today correspond to the Balsas, Armería-Ayuquila and Mezquital Rivers, associated with ancestors of the tribes Characodontini and mainly Ilyodontini (Fig. 3C). The entire Ilyodontini tribe diversified in Ameca, Armería-Ayuquila, and Coahuayana-Tamazula river basins [20], and *M. bravoae* Lineage I codiversified through a vertical transmission pattern [39] into derived illiodontins. *Margotrema bravoae* Lineage I is the only lineage found in Ilyodontini, yet Ilyodontini is not the only host group of this lineage. For example, there is a record of *M. bravoae* Lineage I in a member of the Chapalichthyini (*Chapalicthys pardalis* -j-), inhabiting the same river drainages (Martínez-Aquino et al. unpublished data), recovered as a host-switching and sharing event, congruent with a horizontal transmission pattern [39]. Additionally, *M. bravoae* Lineage I has also been found in Cyprinidae (*Codoma ornata*), following a postulated host-switch from and lineage loss in an ancestor of *Characodon audax* in the Lower Conchos River in the last 500,000 years. Although goodeids no longer inhabit the Lower Conchos River, this host-switch-extinction event is highly plausible, as a goodeid fossil (*Empetrichthys erdisi*) was found in the Yaqui River in Sonora, North-western Mexico, a locality close to the area where goodeids are currently distributed (Minckley et al. [40], and citations therein). In addition, recent discoveries of *Margotrema* sp. associated with other cyprinid fish species from hydrological systems in northern Mexico can be seen as a shadow of past events, possibly reflecting this host-switch-extinction event (Table S1 and Fig. 5B).

The evolutionary history and divergence date of *M. bravoae* Lineage I (3.2 Ma) are congruent with the hypothesis of the origin of the genus *Allodontichthys* (3.6–2.9 Ma) in the Ameca, Armería-Ayuquila, and Coahuayana-Tamazula river basins, configured by vicariant events [20] (Fig. 5C). These patterns were associated with geological events that shaped the biogeographic history of several freshwater fish taxa, such as the uprising of the Sierras de Manantlán and Cacoma, the volcanic activity of the Talpa-Mascota graben (geological depression; 3.6 Ma) and the reactivation of the Colima and Tamazula graben in the Pliocene [38], [41].

According to Domínguez-Domínguez et al. [20], the MRCA of *Allodontichthys* and *Ilyodon* began diversifying during the early Pleistocene (ca. 2 Ma), dispersing from the Armería-Ayuquila River into several hydrological systems in central Mexico such as Ameca, Balsas, Coahuayana-Tamazula and Purificación-Mascota river basins. Previous records of *Margotrema* (Martínez-Aquino et al. unpublished data) in illyodontins in the Coahuayana river basin (*Allodonthichthys hubbsi* in El Tule, Jalisco, and *A. tamazulae* in Tamazula River, Jalisco), are congruent with the hypotheses of area expansion of *M. bravoae* Lineage I, following the diversification process of the Ilyodontini tribe. Similarly, these records also support the ancestral connection between the Armería-Ayuquila and Coahuayana hydrological systems where the MRCA of the Ilyodontini was distributed [20].

### 2. Xenotaenia resolanae-Margotrema resolanae: an allopatric cospeciation model

The separation of *M. resolanae* from its MRCA with *M. bravoae* was dated at approximately 6.53 Ma, meaning that the biogeographic history of *M. resolanae* in particular is difficult to reconstruct, especially given the many postulated reticulate geological changes that have taken place in the area during the last 6 to 7 Ma. Nevertheless, the strict host-specificity shown by *M. resolanae* alludes to cospeciation, further supported by the reconstruction carried out in TreeMap. The restricted distribution range of both species may be the result of allopatric speciation by peripheral isolates, where populations of both hosts and parasites were isolated at the edge of the distributional range of the MRCAs, resulting in a strong host-specificity pattern that illustrates a classical model of cospeciation reflecting reciprocal selection, i.e. coevolution. The divergence date of the MRCA of Illydontini (6.9 Ma) and the posterior diversification process of *X. resolanae*, restricted to the Purificación-Mascota River Basin (between 5.6 to 5.1 Ma) support this pattern. Unfortunately, geological information of this region is scarce, where only the volcanic activity of the Talpa-Mascota graben dated ca. 4.6 Ma has been documented [20].

### 3. Early beginnings of *Margotrema*: future investigations into the past

In light of the current available data, any inferences made regarding the geographic range and host association of the MRCA of *Margotrema* are speculative. The ancestral area of *Margotrema* may be similar to that of the MRCA for Goodeinae (divergence dated at 15.5 to 8 Ma; see [20] Fig. 5D). Our results neither support nor reject the Nearctic affinity of this digenean, as postulated by Pérez-Ponce de León et al. [42] and later supported by Curran et al. [43], who found a close sister group relationship between *Margotrema* and *Crepidostomum*, which is a common intestinal parasite of centrarchiids in other parts of North America. To contribute to a more precise understanding of potential ancestral areas and hosts in this host-parasite system, future studies will need to include the sister groups of *Margotrema*.

### 4. Codivergence patterns

This study provides empirical evidence that demonstrates that the historical biogeography and evolutionary history of the digenean *Margotrema* spp. across central Mexico in part mirrors that of their goodeine freshwater fish hosts at varying levels. The diversification process of the genus *Margotrema* found in this study is driven by a combination of geography and host-specificity at three hierarchical levels of codivergence: a) *Species-Species* (Cospeciation), b) *Species-Lineage* (Type I Codivergence) and c) *Tribe-Lineage* (Type II Codivergence). A series of biogeographic and host-parasite events explain these three codivergence patterns. These results clearly show codivergent patterns for the Goodeinae-*Margotrema* association, even though these taxa display intermittent periods of independent evolutionary histories. In this context, the allopatric cospeciation process proposed between *X. resolanae*-*M. resolanae* agrees with a *one by one* model of *coevolution* [44]. Combining the approaches of historical association (*sensu* Page and Charleston [32]) and parametric methods in biogeography [35], we can integrate events, processes and patterns on a temporal scale to discriminate between several hypotheses of codivergence. We have shown in this study that the DEC algorithm can successfully be applied to reconstruct host-parasite evolutionary histories in addition to biogeographic processes (for which it was originally intended) and it can complement other methods in detecting fine-scale events. For example, the multiple host-switching events detected by DEC support the vertical transmission patterns of *M. bravoae* (Lineage I, II and III) throughout the evolutionary history of Goodeinae, allowing for the distinction between “speciation” and “colonisation” events (see [23], [45], [46]).

The codivergent patterns we uncovered between Goodeinae and *M. bravoae* (Lineages I, II and III) support Vernon Kellog's parasitological rule in that “*the parasites evolve more slowly than their hosts*” [33]. The intuitive expectation would be that digeneans should evolve more rapidly than their freshwater fish hosts, since the parasites have shorter generation times compared to their hosts [47], [48]. However, this study shows a higher diversity of host species compared to *Margotrema* on a similar timescale. In addition, individuals of *M. bravoae* Lineage I were found in hydrological systems that are more than 1600 km apart and have been separated for at least 5 Ma [20], suggesting that minimal diversification has taken place in the parasite during the last 900,000 years. These different rates of diversification can be explained by diversifying selection acting more strongly on the fishes, due to their more variable external environment relative to the one of the endohelminth parasites. Divergence times for each host and parasite lineage - keeping in mind that we are talking about different taxonomic levels for the two - are relatively congruent and demonstrate the concordance of the evolutionary and biogeographic history between the two taxa, both in time and space, i.e. the divergence date of *C. audax* is 1.5 Ma, while for *M. bravoae* lineage II it was estimated at 1.04 Ma. This is in agreement with Page [23] mentioning that the relative ages of speciation events in the host and parasite lineages are key to reconstructing the history of the host-parasite assemblages [49], [50]. The coevolutionary patterns herein described follow a geographic mosaic in which the populations differ in their characteristics and specialisations with respect to the species with which they interact [51]. Therefore, the patterns of codivergence uncovered for the Goodeinae-*M. bravoae* associations occur at the three aforementioned levels, and are thus congruent with the geographic mosaic theory. This might also be related with the macroevolutionary mosaic as described by Hoberg and Brooks [52], where environmental changes may drive both the persistence and diversification of host-parasite systems, generating opportunities for host-switching during geographic expansion, but also permitting cospeciation during episodes of geographic isolation. Finally, the geographic scenario produced by the complex geologic and climatic history of the region seems to be the most important determinant that drives the evolution of *Margotrema* spp. and its codivergent association with their goodeine hosts.

## Supporting Information

Figure S1 **Hydrological systems of this study.**
(PDF)

Table S1 **Additional records of** ***Margotrema*** **in freshwater fish species from Mexico.**
(DOCX)

File S1 **Detailed output of DEC analyses in Lagrange for ancestral area (S1A) and host (S1B) reconstructions.**
(DOC)
