# Supplementary material for: Do the Historical Biogeography and Evolutionary History of the Digenean Margotrema spp. across Central Mexico Mirror Those of Their Freshwater Fish Hosts (Goodeinae)?
Source: PLoS One. 2014 Jul 7;9(7):e101700. doi: 10.1371/journal.pone.0101700 (PMC4084993; doi:10.1371/journal.pone.0101700)
Supplement: Figure S1 — Hydrological systems of this study. (PDF) [file pone.0101700.s001.pdf]

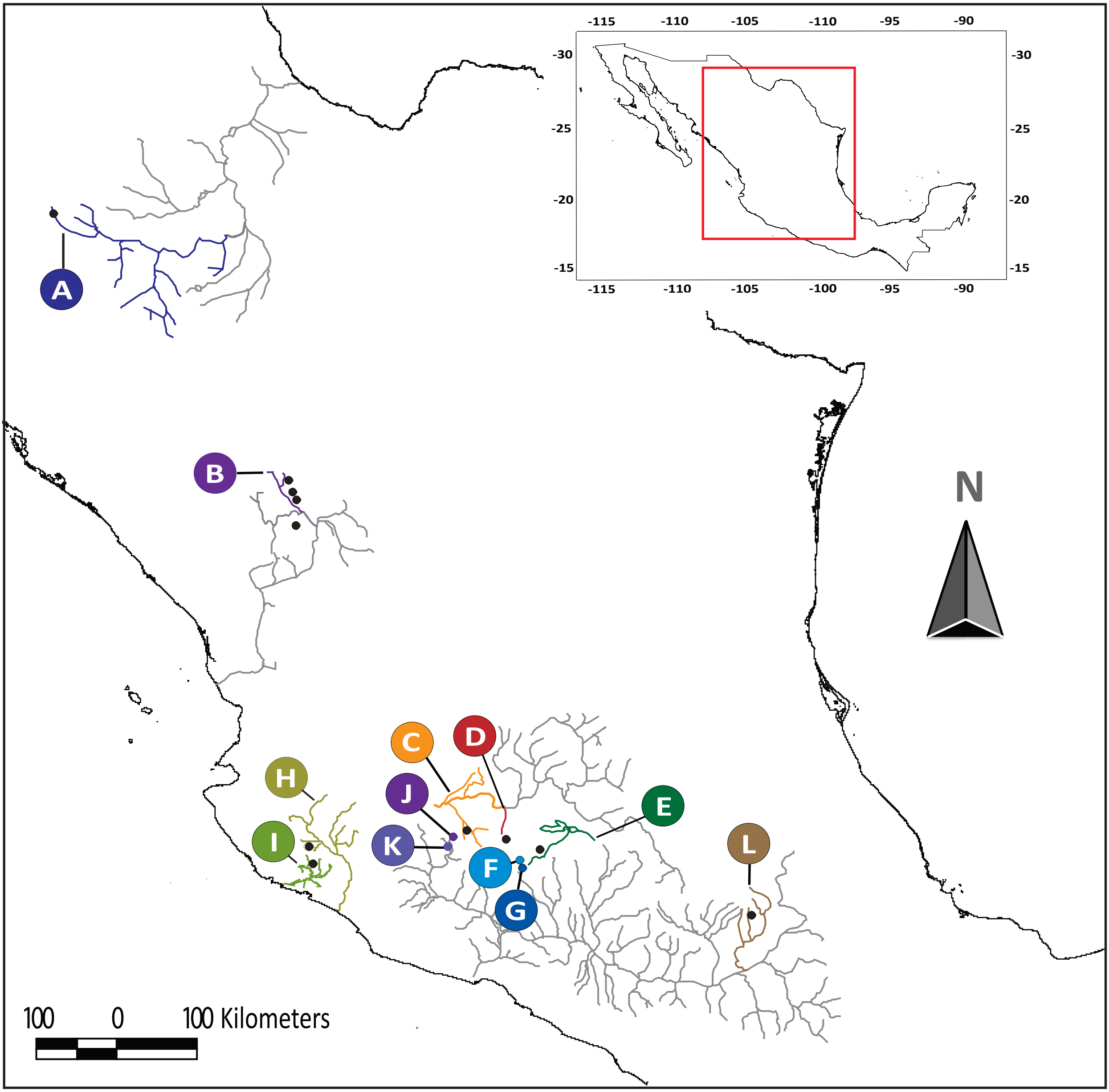

Figure S1. Hydrological systems from this study.

Black dots indicate collecting sites within each of the water bodies, represented by uppercase letters in coloured circles (A = Lower Conchos River; B = Upper and Middle Mezquital River; C = Lower Lerma River; D = Zacapu Lake; E = Cuitzeo Lake; F = Pátzcuaro Lake; G = Zirahuén Lake; H = Armería-Ayuquila Rivers; I = Cuzalapa River; J = Cotija; K = Lower Balsas River; L = Upper Balsas River. Codes correspond to those found in Table 1 of the manuscript.
