# Supplementary material for: Do the Historical Biogeography and Evolutionary History of the Digenean Margotrema spp. across Central Mexico Mirror Those of Their Freshwater Fish Hosts (Goodeinae)?
Source: PLoS One. 2014 Jul 7;9(7):e101700. doi: 10.1371/journal.pone.0101700 (PMC4084993; doi:10.1371/journal.pone.0101700)
Supplement: Table S1 — Additional records of Margotrema in freshwater fish species from Mexico. (DOCX) [file pone.0101700.s002.docx]

| **Table S1.** Additional records of *Margotrema* in freshwater fish species from Mexico. | | | |
| --- | --- | --- | --- |
| **LN** | **Host species** | **Locality** | **Georreferences** |
| 1 | *Cyprinella lutrensis* | Buenaventura, Chihuahua | 29° 50' 16.94'' N; 107° 28' 24.86'' W |
| 2 | *Cyprinidae* | Conchos River, Chihuahua | Unpublished data |
| 3 | *Cyprinidae* | Pagigochic River, Chihuahua | Unpublished data |
| 4 | *Cyprinodon nazas* | Río Guatimape in the Sofía town, Durango | 24° 54’ 41.1’’N; 104° 32’ 7.4’’W |
| 5 | *Codoma ornate* | Río Piaxtla, Municipio de San Dimas, Durango | 24°21'59'' N; 105°31’7.8’’ W |
| 6 | *Allodontichthys hubbsi* | El Tule, Jalisco | 19° 19' 34.2'' N; 103° 22' 15'' W |
| 7 | *Allodontichthys tamazulae* | Río Tamazula, Jalisco | 19° 43' 22.7'' N; 103° 12' 08.5'' W |
| 8 | *Allotoca maculate* | Río San Marcos, Jalisco | 20° 46' 35.7'' N; 104° 09' 52.6'' W |
| 9 | *Ilyodon furcidens* | Río Potrero Grande, Jalisco | 19° 43' 22.7'' N; 103° 12' 08.5'' W |
| 10 | *Neoophorus regalis* | Los Reyes, Michoacán | 19° 33' 43.5'' N; 102° 27' 39'' W |
| 11 | *Ilyodon cortesae* | Manantial Cutzaróndiro, Michoacán | 19° 10' 59'' N; 101° 30' 13'' W |
| 12 | *Girardinichthys multiradiatus* | Canal el Porvenir, Michoacán | 19° 40' 29'' N; 100° 38' 25'' W |
| 13 | *Girardinichthys multiradiatus* | Villa Victoria, Estado de México | 19° 27' 30'' N; 99° 59' 39'' W |
| 14 | *Girardinichthys multiradiatus* | Ciénega La Lagunilla, Estado de México | 19° 08' 30'' N; 99° 30' 12'' W |

Additional records of based on [1,2], Martínez-Aquino et al.`s, unpublished data and G. Pérez-Ponce de León, unpublished data. The locality number (LN) corresponds to the numbers shown in Figure 5B and 5C. For more details see text in the manuscript.

Supplementary References

1. Aguilar-Aguilar R, Rosas-Valdez R, Martínez-Aquino A, Pérez-Rodríguez R, Domínguez-Domínguez O, et al. (2010) Helminth fauna of two cyprinid fish (*Campostoma ornatum* and *Codoma ornata*) from the upper Piaxtla River, Northwestern Mexico. Helminthologia, 47: 251–256. <http://dx.doi.org/10.2478/s11687-010-0039-2>
2. Pérez-Ponce de León G, Mendoza-Garfias B, Rosas-Valdez R, Choudhury A (2013) New host and locality records of freshwater fish helminth parasites in river basin north of the Transmexican Volcanic Belt: another look at biogeographical patterns. Rev Mex Biodivers 84: 556–562.
