# Supplementary material for: Do the Historical Biogeography and Evolutionary History of the Digenean Margotrema spp. across Central Mexico Mirror Those of Their Freshwater Fish Hosts (Goodeinae)?
Source: PLoS One. 2014 Jul 7;9(7):e101700. doi: 10.1371/journal.pone.0101700 (PMC4084993; doi:10.1371/journal.pone.0101700)
Supplement: File S1 — Detailed output of DEC analyses in Lagrange for ancestral area (S1A) and host (S1B) reconstructions. (DOC) [file pone.0101700.s003.doc]

Martínez-Aquino et al. PLOS ONE *Margotrema* manuscript

**SUPPLEMENTARY INFORMATION FILE S1: Detailed output of DEC analyses in Lagrange for ancestral area (S1A) and host (S1B) reconstructions.**

**SUPPLEMENTARY INFORMATION FILE S1A**

***THE FOLLOWING ARE THE LAGRANGE ANCESTRAL HOST RECONSTRUCTION RESULTS***

Lagrange: likelihood analysis of geographic range evolution

Version: 20130526

Author: Richard Ree <rree@fieldmuseum.org>

https://github.com/rhr/lagrange-python

Newick tree with interior nodes labeled:

(((((((lineage3_F:0.0166580112435,lineage3_D:0.0166580112435)N2:0.0336261725424,lineage3_G:0.0502841837859)N4:0.0821043170163,(lineage3_E:0.071546218251,lineage3_C:0.071546218251)N7:0.0608422825512)N8:0.0464777274104,lineage3_J:0.178866228213)N10:0.856628905793,lineage4_B:1.03549513401)N12:2.16772909703,((lineage2_H:0.389153852001,lineage2_A:0.389153852001)N15:0.512338691808,(lineage2_K:0.188078442474,lineage2_L:0.188078442474)N18:0.713414101335)N19:2.30173168723)N20:3.33210518255,lineage1_I:6.53532941359)N22:0.0;

Cladogram (branch lengths not to scale):

--------+ [F] lineage3_F

------N2+

------N4+ --------+ [D] lineage3_D

: :

------N8+ ----------------+ [G] lineage3_G

: :

: : ------------+ [E] lineage3_E

-----N10+ ----------N7+

: : ------------+ [C] lineage3_C

-----N12+ :

: : --------------------------------+ [J] lineage3_J

: :

: ----------------------------------------+ [B] lineage4_B

-----N20+

: : ----------------+ [H] lineage2_H

: : -------------N15+

: : : ----------------+ [A] lineage2_A

N22+ -------------N19+

: : ----------------+ [K] lineage2_K

: -------------N18+

: ----------------+ [L] lineage2_L

:

--------------------------------------------------------+ [I] lineage1_I

***RESULTS OF RUN FROM Margo_loc.lagrange.py INPUT FILE SUBMITTED TO DRYAD***

Global ML at root node:

-lnL = 55.93

dispersal = 0.926

extinction = 0.3693

Ancestral range subdivision/inheritance scenarios ('splits') at

internal nodes.

* Split format: [left|right], where 'left' and 'right' are the ranges

inherited by each descendant branch (on the printed tree, 'left' is

the upper branch, and 'right' the lower branch).

* Only splits within 2 log-likelihood units of the maximum for each

node are shown. 'Rel.Prob' is the relative probability (fraction of

the global likelihood) of a split.

At node N22:

split lnL Rel.Prob

[H|H] -60.41 0.01138

[I|I] -60.51 0.01034

[B|B] -60.85 0.007318

[A|A] -61.23 0.005032

[BHL|H] -61.43 0.004125

[L|L] -61.44 0.004048

[BH|I] -61.49 0.003879

[BH|H] -61.51 0.003791

[H|HI] -61.53 0.003707

[E|E] -61.57 0.003583

[C|C] -61.57 0.003583

[G|G] -61.58 0.003537

[B|BHI] -61.58 0.003518

[F|F] -61.59 0.003494

[D|D] -61.59 0.003494

[ABH|H] -61.61 0.003446

[B|HI] -61.63 0.003352

[H|I] -61.67 0.003236

[J|J] -61.68 0.003187

[K|K] -61.71 0.003094

[BHI|I] -61.72 0.003078

[B|BH] -61.77 0.002917

[B|H] -61.79 0.002859

[HI|I] -61.84 0.002719

[AB|H] -61.86 0.002665

[HL|I] -61.9 0.002568

[HL|H] -61.92 0.00251

[H|BHI] -61.99 0.002335

[H|HIL] -62 0.002319

[BHK|H] -62.04 0.002234

[H|HIJ] -62.05 0.00222

[H|HIK] -62.05 0.00222

[H|CHI] -62.06 0.002193

[H|GHI] -62.06 0.002193

[H|DHI] -62.06 0.002193

[H|FHI] -62.06 0.002193

[H|EHI] -62.06 0.002193

[A|BH] -62.07 0.002168

[HIL|I] -62.1 0.002093

[B|BHL] -62.12 0.002063

[BHJ|H] -62.12 0.002054

[I|HI] -62.16 0.001975

[H|BH] -62.18 0.001936

[B|BHJ] -62.19 0.001914

[B|BHK] -62.19 0.001914

[AB|B] -62.2 0.001895

[H|HL] -62.22 0.001872

[L|HIL] -62.23 0.001846

[BHL|L] -62.23 0.001842

[BHI|H] -62.25 0.001805

[H|HK] -62.27 0.001778

[H|HJ] -62.27 0.001778

[L|HI] -62.27 0.001772

[HK|I] -62.28 0.001761

[BHL|B] -62.28 0.001759

[HJ|I] -62.3 0.001726

[EH|I] -62.3 0.001726

[CH|I] -62.3 0.001726

[HK|H] -62.3 0.001721

[C|HI] -62.3 0.001712

[E|HI] -62.3 0.001712

[GH|I] -62.31 0.001709

[FH|I] -62.32 0.001694

[DH|I] -62.32 0.001694

[B|HL] -62.32 0.001692

[G|HI] -62.32 0.00169

[C|CHI] -62.32 0.001688

[E|EHI] -62.32 0.001688

[HJ|H] -62.32 0.001687

[H|GH] -62.32 0.001687

[H|DH] -62.32 0.001687

[H|FH] -62.32 0.001687

[H|EH] -62.32 0.001687

[H|CH] -62.32 0.001687

[CH|H] -62.32 0.001686

[EH|H] -62.32 0.001686

[GH|H] -62.33 0.00167

[D|HI] -62.33 0.00167

[F|HI] -62.33 0.00167

[G|GHI] -62.33 0.001666

[FH|H] -62.34 0.001655

[DH|H] -62.34 0.001655

[D|DHI] -62.34 0.001646

[F|FHI] -62.34 0.001646

[BH|B] -62.36 0.001617

[A|ABH] -62.37 0.001609

[B|HK] -62.37 0.001608

[B|HJ] -62.37 0.001608

[HI|H] -62.38 0.001594

[BEH|H] -62.41 0.001547

[BCH|H] -62.41 0.001547

[BGH|H] -62.42 0.001529

[BDH|H] -62.43 0.001511

[BFH|H] -62.43 0.001511

[L|H] -62.43 0.001511

[A|B] -62.43 0.001511

[L|HL] -62.44 0.00149

[ABH|B] -62.46 0.00147

[C|H] -62.46 0.001461

[E|H] -62.46 0.001461

[J|HI] -62.46 0.00146

[J|HIJ] -62.47 0.001457

[G|H] -62.48 0.001442

[D|H] -62.49 0.001424

[F|H] -62.49 0.001424

[K|HI] -62.49 0.001418

[K|HIK] -62.5 0.001415

[HIK|I] -62.5 0.00141

[CHI|I] -62.52 0.001385

[EHI|I] -62.52 0.001385

[HIJ|I] -62.53 0.001373

[GHI|I] -62.53 0.001373

[H|BHL] -62.53 0.001369

[DHI|I] -62.53 0.001361

[FHI|I] -62.53 0.001361

[B|ABH] -62.58 0.001299

[C|CH] -62.58 0.001298

[E|EH] -62.58 0.001298

[A|AB] -62.59 0.001287

[G|GH] -62.59 0.001282

[BC|H] -62.6 0.001277

[BE|H] -62.6 0.001277

[B|GH] -62.6 0.001271

[B|FH] -62.6 0.001271

[B|DH] -62.6 0.001271

[B|EH] -62.6 0.001271

[B|CH] -62.6 0.001271

[H|BHJ] -62.6 0.00127

[H|BHK] -62.6 0.00127

[D|DH] -62.61 0.001266

[F|FH] -62.61 0.001266

[BG|H] -62.62 0.001254

[J|H] -62.62 0.001245

[I|BHI] -62.62 0.001244

[I|HIL] -62.63 0.001235

[BD|H] -62.63 0.001233

[BF|H] -62.63 0.001233

[HIL|H] -62.64 0.001227

[K|H] -62.65 0.001209

[I|HIJ] -62.68 0.001182

[I|HIK] -62.68 0.001182

[B|G] -62.69 0.001169

[B|F] -62.69 0.001169

[B|E] -62.69 0.001169

[B|D] -62.69 0.001169

[B|C] -62.69 0.001169

[I|CHI] -62.69 0.001168

[I|GHI] -62.69 0.001168

[I|DHI] -62.69 0.001168

[I|FHI] -62.69 0.001168

[I|EHI] -62.69 0.001168

[J|HJ] -62.69 0.001167

[B|BGH] -62.7 0.001151

[B|BDH] -62.7 0.001151

[B|BEH] -62.7 0.001151

[B|BFH] -62.7 0.001151

[B|BCH] -62.7 0.001151

[K|HK] -62.72 0.001133

[HL|L] -62.73 0.00112

[ABE|B] -62.75 0.001093

[ABC|B] -62.75 0.001093

[L|BHL] -62.76 0.00109

[AB|G] -62.76 0.00109

[AB|F] -62.76 0.00109

[AB|E] -62.76 0.00109

[AB|D] -62.76 0.00109

[AB|C] -62.76 0.00109

[BC|B] -62.76 0.001089

[BE|B] -62.76 0.001089

[ABG|B] -62.77 0.001077

[BG|B] -62.78 0.00107

[ABD|B] -62.78 0.001062

[ABF|B] -62.78 0.001062

[AB|A] -62.79 0.001052

[BD|B] -62.79 0.001052

[BF|B] -62.79 0.001052

[ABE|E] -62.8 0.001048

[ABC|C] -62.8 0.001048

[BE|E] -62.8 0.001044

[BC|C] -62.8 0.001044

[B|AB] -62.8 0.001039

[ABG|G] -62.81 0.001033

[B|BD] -62.82 0.001026

[B|BG] -62.82 0.001026

[B|BE] -62.82 0.001026

[B|BF] -62.82 0.001026

[B|BC] -62.82 0.001026

[BG|G] -62.82 0.001025

[ABF|F] -62.82 0.001018

[ABD|D] -62.82 0.001018

[BH|L] -62.83 0.001015

[I|H] -62.83 0.001011

[EHL|H] -62.83 0.001008

[CHL|H] -62.83 0.001008

[BF|F] -62.83 0.001008

[BD|D] -62.83 0.001008

[GHL|H] -62.84 0.0009983

[FHL|H] -62.85 0.000989

[DHL|H] -62.85 0.000989

[BH|K] -62.87 0.00097

[BH|J] -62.87 0.00097

[BHK|B] -62.89 0.000953

[BHK|K] -62.89 0.0009528

[HKL|H] -62.89 0.0009518

[HJL|H] -62.9 0.0009478

[L|BH] -62.92 0.0009249

[BHJ|B] -62.98 0.000876

[BHJ|J] -62.98 0.0008758

[H|ABH] -62.99 0.0008618

[H|L] -63.01 0.0008471

[J|BHJ] -63.02 0.0008336

[HIK|H] -63.03 0.0008269

[ABH|A] -63.05 0.0008159

[CHI|H] -63.05 0.0008123

[EHI|H] -63.05 0.0008123

[H|B] -63.05 0.0008093

[K|BHK] -63.05 0.0008092

[H|K] -63.05 0.0008091

[H|J] -63.05 0.0008091

[HIJ|H] -63.06 0.0008051

[GHI|H] -63.06 0.0008048

[DHI|H] -63.07 0.0007979

[FHI|H] -63.07 0.0007979

[H|HKL] -63.08 0.0007873

[H|HJL] -63.08 0.0007873

[H|G] -63.1 0.0007758

[H|F] -63.1 0.0007758

[H|E] -63.1 0.0007758

[H|D] -63.1 0.0007758

[H|C] -63.1 0.0007758

[BH|G] -63.1 0.000775

[BH|F] -63.1 0.000775

[BH|E] -63.1 0.000775

[BH|D] -63.1 0.000775

[BH|C] -63.1 0.000775

[CL|H] -63.1 0.0007722

[EL|H] -63.1 0.0007722

[BHI|B] -63.1 0.0007699

[H|GHL] -63.11 0.0007641

[H|EHL] -63.11 0.0007641

[H|FHL] -63.11 0.0007641

[H|DHL] -63.11 0.0007641

[H|CHL] -63.11 0.0007641

[H|BGH] -63.11 0.0007638

[H|BDH] -63.11 0.0007638

[H|BEH] -63.11 0.0007638

[H|BFH] -63.11 0.0007638

[H|BCH] -63.11 0.0007638

[J|BH] -63.11 0.0007624

[A|BD] -63.11 0.0007623

[A|BG] -63.11 0.0007623

[A|BE] -63.11 0.0007623

[A|BF] -63.11 0.0007623

[A|BC] -63.11 0.0007623

[GL|H] -63.12 0.000761

[DL|H] -63.13 0.0007506

[FL|H] -63.13 0.0007506

[C|BH] -63.14 0.0007451

[E|BH] -63.14 0.0007451

[H|HJK] -63.14 0.0007441

[K|BH] -63.14 0.0007401

[CEH|H] -63.15 0.000736

[G|BH] -63.15 0.0007355

[HK|K] -63.15 0.0007338

[EHJ|H] -63.16 0.0007314

[CHJ|H] -63.16 0.0007314

[CGH|H] -63.16 0.0007295

[EGH|H] -63.16 0.0007295

[EHK|H] -63.16 0.0007266

[CHK|H] -63.16 0.0007266

[D|BH] -63.16 0.0007266

[F|BH] -63.16 0.0007266

[CFH|H] -63.17 0.000723

[CDH|H] -63.17 0.000723

[EFH|H] -63.17 0.000723

[DEH|H] -63.17 0.000723

[GHJ|H] -63.17 0.000722

[H|GHK] -63.17 0.0007217

[H|GHJ] -63.17 0.0007217

[H|FHK] -63.17 0.0007217

[H|FHJ] -63.17 0.0007217

[H|EHK] -63.17 0.0007217

[H|DHK] -63.17 0.0007217

[H|EHJ] -63.17 0.0007217

[H|DHJ] -63.17 0.0007217

[H|CHK] -63.17 0.0007217

[H|CHJ] -63.17 0.0007217

[C|HL] -63.17 0.0007204

[E|HL] -63.17 0.0007204

[HJ|J] -63.17 0.0007194

[GHK|H] -63.17 0.0007186

[FGH|H] -63.18 0.000716

[DGH|H] -63.18 0.000716

[DHJ|H] -63.18 0.0007131

[FHJ|H] -63.18 0.0007131

[G|HL] -63.18 0.0007112

[FHK|H] -63.18 0.0007111

[DHK|H] -63.18 0.0007111

[DFH|H] -63.19 0.0007096

[L|HK] -63.19 0.0007082

[L|HJ] -63.19 0.0007082

[H|DEH] -63.19 0.0007059

[H|EGH] -63.19 0.0007059

[H|FGH] -63.19 0.0007059

[H|EFH] -63.19 0.0007059

[H|DGH] -63.19 0.0007059

[H|CFH] -63.19 0.0007059

[H|DFH] -63.19 0.0007059

[H|CGH] -63.19 0.0007059

[H|CEH] -63.19 0.0007059

[H|CDH] -63.19 0.0007059

[D|HL] -63.2 0.0007025

[F|HL] -63.2 0.0007025

[EH|E] -63.21 0.0006896

[CH|C] -63.21 0.0006896

[CL|L] -63.21 0.0006895

[EL|L] -63.21 0.0006895

[CE|H] -63.22 0.000687

[HJK|H] -63.22 0.0006848

[C|HK] -63.22 0.0006845

[E|HK] -63.22 0.0006845

[C|HJ] -63.22 0.0006845

[E|HJ] -63.22 0.0006845

[BC|L] -63.22 0.000684

[BE|L] -63.22 0.000684

[GH|G] -63.22 0.0006829

[CG|H] -63.23 0.0006803

[EG|H] -63.23 0.0006803

[GL|L] -63.23 0.0006796

[B|A] -63.23 0.000677

[FH|F] -63.23 0.0006767

[DH|D] -63.23 0.0006767

[G|HK] -63.23 0.0006758

[G|HJ] -63.23 0.0006758

[CF|H] -63.24 0.0006737

[CD|H] -63.24 0.0006737

[DE|H] -63.24 0.0006737

[EF|H] -63.24 0.0006737

[B|EL] -63.24 0.0006719

[B|GL] -63.24 0.0006719

[B|FL] -63.24 0.0006719

[B|DL] -63.24 0.0006719

[B|CL] -63.24 0.0006719

[BG|L] -63.24 0.0006718

[L|GH] -63.24 0.0006716

[L|FH] -63.24 0.0006716

[L|DH] -63.24 0.0006716

[L|EH] -63.24 0.0006716

[L|CH] -63.24 0.0006716

[DL|L] -63.24 0.0006702

[FL|L] -63.24 0.0006702

[D|HK] -63.25 0.0006675

[F|HK] -63.25 0.0006675

[D|HJ] -63.25 0.0006675

[F|HJ] -63.25 0.0006675

[DG|H] -63.25 0.0006664

[FG|H] -63.25 0.0006664

[CJ|H] -63.25 0.0006639

[EJ|H] -63.25 0.0006639

[BD|L] -63.26 0.0006604

[BF|L] -63.26 0.0006604

[BEH|B] -63.26 0.00066

[BCH|B] -63.26 0.00066

[DF|H] -63.26 0.0006599

[JL|H] -63.27 0.000654

[BC|K] -63.27 0.0006534

[BE|K] -63.27 0.0006534

[BC|J] -63.27 0.0006534

[BE|J] -63.27 0.0006534

[BEL|L] -63.27 0.0006531

[BCL|L] -63.27 0.0006531

[C|L] -63.27 0.0006521

[E|L] -63.27 0.0006521

[GJ|H] -63.27 0.0006521

[BGH|B] -63.27 0.000652

[C|GH] -63.27 0.0006492

[E|GH] -63.27 0.0006492

[C|DH] -63.27 0.0006492

[C|FH] -63.27 0.0006492

[C|EH] -63.27 0.0006492

[E|DH] -63.27 0.0006492

[E|FH] -63.27 0.0006492

[E|CH] -63.27 0.0006492

[BDH|B] -63.28 0.0006445

[BFH|B] -63.28 0.0006445

[L|K] -63.28 0.0006444

[L|J] -63.28 0.0006444

[G|L] -63.28 0.0006437

[HL|B] -63.29 0.0006423

[BG|K] -63.29 0.0006417

[BG|J] -63.29 0.0006417

[DJ|H] -63.29 0.000641

[FJ|H] -63.29 0.000641

[B|FK] -63.29 0.0006409

[B|GJ] -63.29 0.0006409

[B|GK] -63.29 0.0006409

[B|FJ] -63.29 0.0006409

[B|EK] -63.29 0.0006409

[B|EJ] -63.29 0.0006409

[B|DJ] -63.29 0.0006409

[B|DK] -63.29 0.0006409

[B|CK] -63.29 0.0006409

[B|CJ] -63.29 0.0006409

[G|DH] -63.29 0.0006409

[G|FH] -63.29 0.0006409

[G|EH] -63.29 0.0006409

[G|CH] -63.29 0.0006409

[KL|H] -63.29 0.0006392

[D|L] -63.3 0.0006359

[F|L] -63.3 0.0006359

[BGL|L] -63.3 0.0006333

[D|GH] -63.3 0.0006331

[F|GH] -63.3 0.0006331

[D|FH] -63.3 0.0006331

[D|EH] -63.3 0.0006331

[F|DH] -63.3 0.0006331

[D|CH] -63.3 0.0006331

[F|EH] -63.3 0.0006331

[F|CH] -63.3 0.0006331

[BEH|E] -63.3 0.0006326

[BCH|C] -63.3 0.0006326

[CK|H] -63.3 0.0006322

[EK|H] -63.3 0.0006322

[EL|E] -63.3 0.0006315

[CL|C] -63.3 0.0006315

[BD|K] -63.3 0.0006308

[BF|K] -63.3 0.0006308

[BD|J] -63.3 0.0006308

[BF|J] -63.3 0.0006308

[L|HKL] -63.31 0.000627

[L|HJL] -63.31 0.000627

[L|JL] -63.31 0.0006259

[L|KL] -63.31 0.0006259

[BGH|G] -63.31 0.000625

[BEL|B] -63.31 0.0006239

[BCL|B] -63.31 0.0006239

[C|B] -63.32 0.000623

[E|B] -63.32 0.000623

[C|K] -63.32 0.0006229

[E|K] -63.32 0.0006229

[C|J] -63.32 0.0006229

[E|J] -63.32 0.0006229

[GK|H] -63.32 0.0006227

[GL|G] -63.32 0.0006223

[I|BH] -63.32 0.0006187

[A|ABD] -63.32 0.0006186

[A|ABF] -63.32 0.0006186

[A|ABC] -63.32 0.0006186

[A|ABG] -63.32 0.0006186

[A|ABE] -63.32 0.0006186

[BFH|F] -63.32 0.0006178

[BDH|D] -63.32 0.0006178

[L|G] -63.32 0.0006178

[L|F] -63.32 0.0006178

[L|E] -63.32 0.0006178

[L|D] -63.32 0.0006178

[L|C] -63.32 0.0006178

[G|B] -63.33 0.000615

[G|K] -63.33 0.0006149

[G|J] -63.33 0.0006149

[BDL|L] -63.33 0.0006147

[BFL|L] -63.33 0.0006147

[J|HL] -63.33 0.0006143

[FK|H] -63.33 0.0006138

[DK|H] -63.33 0.0006138

[FL|F] -63.33 0.0006138

[DL|D] -63.33 0.0006138

[L|GHL] -63.34 0.0006086

[L|EHL] -63.34 0.0006086

[L|FHL] -63.34 0.0006086

[L|DHL] -63.34 0.0006086

[L|CHL] -63.34 0.0006086

[D|B] -63.34 0.0006076

[F|B] -63.34 0.0006076

[D|K] -63.34 0.0006074

[F|K] -63.34 0.0006074

[D|J] -63.34 0.0006074

[F|J] -63.34 0.0006074

[ABE|A] -63.34 0.0006069

[ABC|A] -63.34 0.0006069

[BGL|B] -63.35 0.000605

[I|HL] -63.36 0.0005982

[BEL|E] -63.36 0.0005981

[BCL|C] -63.36 0.0005981

[ABG|A] -63.36 0.0005979

[C|G] -63.36 0.0005972

[C|F] -63.36 0.0005972

[E|G] -63.36 0.0005972

[E|F] -63.36 0.0005972

[C|E] -63.36 0.0005972

[C|D] -63.36 0.0005972

[E|D] -63.36 0.0005972

[E|C] -63.36 0.0005972

[K|HL] -63.36 0.0005963

[L|EL] -63.37 0.0005917

[L|GL] -63.37 0.0005917

[L|FL] -63.37 0.0005917

[L|DL] -63.37 0.0005917

[L|CL] -63.37 0.0005917

[G|F] -63.37 0.0005896

[G|E] -63.37 0.0005896

[G|D] -63.37 0.0005896

[G|C] -63.37 0.0005896

[ABD|A] -63.37 0.0005895

[ABF|A] -63.37 0.0005895

[E|EHL] -63.37 0.0005882

[C|CHL] -63.37 0.0005882

[E|BEH] -63.37 0.000588

[C|BCH] -63.37 0.000588

[BDL|B] -63.37 0.0005873

[BFL|B] -63.37 0.0005873

[JL|L] -63.38 0.000584

[J|HK] -63.38 0.0005837

[D|G] -63.38 0.0005824

[D|F] -63.38 0.0005824

[F|G] -63.38 0.0005824

[D|E] -63.38 0.0005824

[F|E] -63.38 0.0005824

[F|D] -63.38 0.0005824

[D|C] -63.38 0.0005824

[F|C] -63.38 0.0005824

[G|GHL] -63.39 0.0005807

[G|BGH] -63.39 0.0005805

[BGL|G] -63.39 0.00058

[F|FHL] -63.4 0.0005737

[D|DHL] -63.4 0.0005737

[D|BDH] -63.4 0.0005734

[F|BFH] -63.4 0.0005734

[E|EL] -63.4 0.000572

[C|CL] -63.4 0.000572

[KL|L] -63.4 0.0005708

[I|HK] -63.41 0.0005684

[I|HJ] -63.41 0.0005684

[K|HJ] -63.41 0.0005667

[CJ|J] -63.41 0.0005662

[EJ|J] -63.41 0.0005662

[BCJ|B] -63.41 0.0005653

[BEJ|B] -63.41 0.0005653

[BCJ|J] -63.41 0.0005652

[BEJ|J] -63.41 0.0005652

[G|GL] -63.41 0.0005647

[BDL|D] -63.42 0.000563

[BFL|F] -63.42 0.000563

[CE|E] -63.42 0.0005618

[CE|C] -63.42 0.0005618

[JL|J] -63.43 0.0005579

[F|FL] -63.43 0.0005578

[D|DL] -63.43 0.0005578

[CG|G] -63.43 0.0005563

[EG|G] -63.43 0.0005563

[CG|C] -63.43 0.0005563

[EG|E] -63.43 0.0005563

[GJ|J] -63.43 0.0005562

[J|L] -63.43 0.0005561

[E|EHK] -63.43 0.0005555

[E|EHJ] -63.43 0.0005555

[C|CHK] -63.43 0.0005555

[C|CHJ] -63.43 0.0005555

[J|GH] -63.43 0.0005536

[J|DH] -63.43 0.0005536

[J|FH] -63.43 0.0005536

[J|EH] -63.43 0.0005536

[J|CH] -63.43 0.0005536

[CF|F] -63.44 0.0005509

[CD|D] -63.44 0.0005509

[EF|F] -63.44 0.0005509

[CF|C] -63.44 0.0005509

[DE|E] -63.44 0.0005509

[CD|C] -63.44 0.0005509

[EF|E] -63.44 0.0005509

[DE|D] -63.44 0.0005509

[G|GHK] -63.44 0.0005484

[G|GHJ] -63.44 0.0005484

[HIL|L] -63.44 0.0005479

[DJ|J] -63.45 0.0005468

[FJ|J] -63.45 0.0005468

[E|EK] -63.45 0.0005456

[E|EJ] -63.45 0.0005456

[C|CK] -63.45 0.0005456

[C|CJ] -63.45 0.0005456

[KL|K] -63.45 0.0005452

[DG|G] -63.45 0.000545

[FG|G] -63.45 0.000545

[FG|F] -63.45 0.000545

[DG|D] -63.45 0.000545

[E|DE] -63.45 0.0005445

[E|EG] -63.45 0.0005445

[E|EF] -63.45 0.0005445

[C|CF] -63.45 0.0005445

[C|CG] -63.45 0.0005445

[C|CD] -63.45 0.0005445

[C|CE] -63.45 0.0005445

[E|CE] -63.45 0.0005445

[BGJ|B] -63.45 0.0005438

[BGJ|J] -63.45 0.0005436

[E|DEH] -63.45 0.0005434

[E|EGH] -63.45 0.0005434

[E|EFH] -63.45 0.0005434

[C|CFH] -63.45 0.0005434

[C|CGH] -63.45 0.0005434

[C|CEH] -63.45 0.0005434

[E|CEH] -63.45 0.0005434

[C|CDH] -63.45 0.0005434

[CJ|C] -63.45 0.0005429

[EJ|E] -63.45 0.0005429

[BCJ|C] -63.46 0.0005418

[BEJ|E] -63.46 0.0005418

[F|FHK] -63.46 0.0005418

[D|DHK] -63.46 0.0005418

[F|FHJ] -63.46 0.0005418

[D|DHJ] -63.46 0.0005418

[K|L] -63.46 0.0005398

[DF|F] -63.46 0.0005396

[DF|D] -63.46 0.0005396

[CK|K] -63.46 0.0005392

[EK|K] -63.46 0.0005392

[I|GH] -63.46 0.0005391

[I|DH] -63.46 0.0005391

[I|FH] -63.46 0.0005391

[I|EH] -63.46 0.0005391

[I|CH] -63.46 0.0005391

[G|GJ] -63.46 0.0005386

[G|GK] -63.46 0.0005386

[BH|A] -63.46 0.0005385

[G|EG] -63.46 0.0005375

[G|FG] -63.46 0.0005375

[G|DG] -63.46 0.0005375

[G|CG] -63.46 0.0005375

[K|GH] -63.46 0.0005374

[K|DH] -63.46 0.0005374

[K|FH] -63.46 0.0005374

[K|EH] -63.46 0.0005374

[K|CH] -63.46 0.0005374

[JK|H] -63.46 0.0005368

[G|FGH] -63.47 0.0005364

[G|EGH] -63.47 0.0005364

[G|DGH] -63.47 0.0005364

[G|CGH] -63.47 0.0005364

[HL|K] -63.47 0.0005351

[HL|J] -63.47 0.0005351

[GJ|G] -63.47 0.0005333

[B|DE] -63.47 0.000533

[B|EG] -63.47 0.000533

[B|FG] -63.47 0.000533

[B|EF] -63.47 0.000533

[B|DF] -63.47 0.000533

[B|DG] -63.47 0.000533

[B|CF] -63.47 0.000533

[B|CG] -63.47 0.000533

[B|CD] -63.47 0.000533

[B|CE] -63.47 0.000533

[F|FK] -63.47 0.0005321

[F|FJ] -63.47 0.0005321

[D|DJ] -63.47 0.0005321

[D|DK] -63.47 0.0005321

[GK|K] -63.48 0.0005311

[J|K] -63.48 0.0005311

[D|DE] -63.48 0.000531

[F|FG] -63.48 0.000531

[D|DF] -63.48 0.000531

[F|EF] -63.48 0.000531

[D|DG] -63.48 0.000531

[F|DF] -63.48 0.000531

[F|CF] -63.48 0.000531

[D|CD] -63.48 0.000531

[D|DEH] -63.48 0.0005299

[F|FGH] -63.48 0.0005299

[D|DGH] -63.48 0.0005299

[F|EFH] -63.48 0.0005299

[D|DFH] -63.48 0.0005299

[F|CFH] -63.48 0.0005299

[F|DFH] -63.48 0.0005299

[D|CDH] -63.48 0.0005299

[DJ|D] -63.49 0.0005242

[FJ|F] -63.49 0.0005242

[E|BE] -63.49 0.0005239

[C|BC] -63.49 0.0005239

[BDJ|B] -63.49 0.0005235

[BFJ|B] -63.49 0.0005235

[FK|K] -63.49 0.0005235

[DK|K] -63.49 0.0005235

[BDJ|J] -63.49 0.0005234

[BFJ|J] -63.49 0.0005234

[BC|G] -63.49 0.000522

[BC|F] -63.49 0.000522

[BE|G] -63.49 0.000522

[BE|F] -63.49 0.000522

[BC|E] -63.49 0.000522

[BC|D] -63.49 0.000522

[BE|D] -63.49 0.000522

[BE|C] -63.49 0.000522

[BGJ|G] -63.49 0.0005212

[G|BG] -63.5 0.0005172

[CK|C] -63.5 0.000517

[EK|E] -63.5 0.000517

[J|HJL] -63.5 0.0005168

[J|JL] -63.5 0.0005159

[K|J] -63.51 0.0005156

[B|BGL] -63.51 0.0005143

[B|BFL] -63.51 0.0005143

[B|BEL] -63.51 0.0005143

[B|BDL] -63.51 0.0005143

[B|BCL] -63.51 0.0005143

[HL|G] -63.51 0.0005131

[HL|F] -63.51 0.0005131

[HL|E] -63.51 0.0005131

[HL|D] -63.51 0.0005131

[HL|C] -63.51 0.0005131

[BG|F] -63.51 0.0005127

[BG|E] -63.51 0.0005127

[BG|D] -63.51 0.0005127

[BG|C] -63.51 0.0005127

[D|BD] -63.51 0.0005109

[F|BF] -63.51 0.0005109

[BCK|B] -63.52 0.0005104

[BEK|B] -63.52 0.0005104

[BCK|K] -63.52 0.0005103

[BEK|K] -63.52 0.0005103

[GK|G] -63.52 0.0005092

[J|G] -63.52 0.0005092

[J|F] -63.52 0.0005092

[J|E] -63.52 0.0005092

[J|D] -63.52 0.0005092

[J|C] -63.52 0.0005092

[BD|G] -63.53 0.000504

[BD|F] -63.53 0.000504

[BF|G] -63.53 0.000504

[BD|E] -63.53 0.000504

[BF|E] -63.53 0.000504

[BF|D] -63.53 0.000504

[BD|C] -63.53 0.000504

[BF|C] -63.53 0.000504

[FK|F] -63.53 0.000502

[DK|D] -63.53 0.000502

[BDJ|D] -63.53 0.0005018

[BFJ|F] -63.53 0.0005018

[K|HKL] -63.53 0.0005017

[K|KL] -63.53 0.0005008

[B|ABD] -63.54 0.0004994

[B|ABC] -63.54 0.0004994

[B|ABF] -63.54 0.0004994

[B|ABG] -63.54 0.0004994

[B|ABE] -63.54 0.0004994

[BGK|B] -63.55 0.0004949

[BGK|K] -63.55 0.0004948

[K|G] -63.55 0.0004944

[K|F] -63.55 0.0004944

[K|E] -63.55 0.0004944

[K|D] -63.55 0.0004944

[K|C] -63.55 0.0004944

[J|JK] -63.55 0.000492

[B|BCK] -63.55 0.0004911

[B|BGK] -63.55 0.0004911

[B|BGJ] -63.55 0.0004911

[B|BEJ] -63.55 0.0004911

[B|BFK] -63.55 0.0004911

[B|BEK] -63.55 0.0004911

[B|BFJ] -63.55 0.0004911

[B|BCJ] -63.55 0.0004911

[B|BDK] -63.55 0.0004911

[B|BDJ] -63.55 0.0004911

[BCK|C] -63.56 0.0004893

[BEK|E] -63.56 0.0004893

[J|HJK] -63.56 0.0004885

[BCE|B] -63.56 0.0004878

[BCG|B] -63.57 0.0004817

[BEG|B] -63.57 0.0004817

[BFK|B] -63.58 0.0004802

[BDK|B] -63.58 0.0004802

[BFK|K] -63.58 0.0004801

[BDK|K] -63.58 0.0004801

[K|JK] -63.58 0.0004776

[BCF|B] -63.59 0.0004754

[BCD|B] -63.59 0.0004754

[BDE|B] -63.59 0.0004754

[BEF|B] -63.59 0.0004754

[BGK|G] -63.59 0.0004744

[K|HJK] -63.59 0.0004742

[J|GHJ] -63.59 0.0004737

[J|FHJ] -63.59 0.0004737

[J|EHJ] -63.59 0.0004737

[J|DHJ] -63.59 0.0004737

[J|CHJ] -63.59 0.0004737

[BFG|B] -63.6 0.0004686

[BDG|B] -63.6 0.0004686

[BCE|E] -63.6 0.0004676

[BCE|C] -63.6 0.0004676

[J|GJ] -63.61 0.0004653

[J|FJ] -63.61 0.0004653

[J|EJ] -63.61 0.0004653

[J|DJ] -63.61 0.0004653

[J|CJ] -63.61 0.0004653

[BDF|B] -63.61 0.0004624

[BCG|G] -63.62 0.0004617

[BEG|G] -63.62 0.0004617

[BCG|C] -63.62 0.0004617

[BEG|E] -63.62 0.0004617

[BFK|F] -63.62 0.0004603

[BDK|D] -63.62 0.0004603

[K|GHK] -63.62 0.0004599

[K|FHK] -63.62 0.0004599

[K|EHK] -63.62 0.0004599

[K|DHK] -63.62 0.0004599

[K|CHK] -63.62 0.0004599

[JK|K] -63.62 0.0004578

[JK|J] -63.62 0.0004578

[BCF|F] -63.63 0.0004557

[BCF|C] -63.63 0.0004557

[BCD|D] -63.63 0.0004557

[BDE|E] -63.63 0.0004557

[BEF|F] -63.63 0.0004557

[BDE|D] -63.63 0.0004557

[BCD|C] -63.63 0.0004557

[BEF|E] -63.63 0.0004557

[K|FK] -63.64 0.0004517

[K|GK] -63.64 0.0004517

[K|EK] -63.64 0.0004517

[K|DK] -63.64 0.0004517

[K|CK] -63.64 0.0004517

[EHL|L] -63.64 0.0004502

[CHL|L] -63.64 0.0004502

[B|BDF] -63.64 0.00045

[B|BDE] -63.64 0.00045

[B|BDG] -63.64 0.00045

[B|BCF] -63.64 0.00045

[B|BCD] -63.64 0.00045

[B|BFG] -63.64 0.00045

[B|BEG] -63.64 0.00045

[B|BCG] -63.64 0.00045

[B|BCE] -63.64 0.00045

[B|BEF] -63.64 0.00045

[BFG|G] -63.64 0.0004491

[BFG|F] -63.64 0.0004491

[BDG|G] -63.64 0.0004491

[BDG|D] -63.64 0.0004491

[GHL|L] -63.65 0.0004457

[BDF|F] -63.66 0.0004432

[BDF|D] -63.66 0.0004432

[FHL|L] -63.66 0.0004416

[DHL|L] -63.66 0.0004416

[HK|B] -63.66 0.0004404

[HJ|B] -63.68 0.0004317

[HI|L] -63.69 0.0004271

[HKL|L] -63.7 0.0004249

[HJL|L] -63.7 0.0004232

[H|AB] -63.73 0.0004138

[EHL|E] -63.73 0.0004123

[CHL|C] -63.73 0.0004123

[GHL|G] -63.74 0.0004082

[HI|B] -63.74 0.0004081

[HI|K] -63.74 0.000408

[HI|J] -63.74 0.000408

[HKL|K] -63.74 0.0004059

[FHL|F] -63.75 0.0004044

[DHL|D] -63.75 0.0004044

[HJL|J] -63.75 0.0004042

[CL|B] -63.77 0.0003953

[EL|B] -63.77 0.0003953

[H|JL] -63.78 0.0003929

[H|KL] -63.78 0.0003929

[HI|G] -63.78 0.0003911

[HI|F] -63.78 0.0003911

[HI|E] -63.78 0.0003911

[HI|D] -63.78 0.0003911

[HI|C] -63.78 0.0003911

[GL|B] -63.79 0.0003896

[DL|B] -63.8 0.0003842

[FL|B] -63.8 0.0003842

[HK|L] -63.8 0.0003841

[HJ|L] -63.82 0.0003766

[CH|L] -63.82 0.0003765

[EH|L] -63.82 0.0003765

[H|JK] -63.82 0.0003747

[GH|L] -63.83 0.0003729

[H|EL] -63.83 0.0003715

[H|GL] -63.83 0.0003715

[H|FL] -63.83 0.0003715

[H|DL] -63.83 0.0003715

[H|CL] -63.83 0.0003715

[FH|L] -63.84 0.0003695

[DH|L] -63.84 0.0003695

[HK|J] -63.85 0.0003669

[BC|A] -63.86 0.0003627

[BE|A] -63.86 0.0003627

[EH|B] -63.87 0.0003597

[CH|B] -63.87 0.0003597

[HJ|K] -63.87 0.0003597

[EH|K] -63.87 0.0003596

[CH|K] -63.87 0.0003596

[CH|J] -63.87 0.0003596

[EH|J] -63.87 0.0003596

[BG|A] -63.87 0.0003563

[GH|B] -63.87 0.0003562

[GH|K] -63.88 0.0003562

[GH|J] -63.88 0.0003562

[H|FK] -63.88 0.0003544

[H|GJ] -63.88 0.0003544

[H|GK] -63.88 0.0003544

[H|FJ] -63.88 0.0003544

[H|EK] -63.88 0.0003544

[H|EJ] -63.88 0.0003544

[H|DJ] -63.88 0.0003544

[H|DK] -63.88 0.0003544

[H|CK] -63.88 0.0003544

[H|CJ] -63.88 0.0003544

[H|DE] -63.88 0.0003537

[H|EG] -63.88 0.0003537

[H|FG] -63.88 0.0003537

[H|EF] -63.88 0.0003537

[H|DF] -63.88 0.0003537

[H|DG] -63.88 0.0003537

[H|CF] -63.88 0.0003537

[H|CG] -63.88 0.0003537

[H|CD] -63.88 0.0003537

[H|CE] -63.88 0.0003537

[FH|B] -63.88 0.000353

[DH|B] -63.88 0.000353

[FH|K] -63.88 0.0003529

[FH|J] -63.88 0.0003529

[DH|K] -63.88 0.0003529

[DH|J] -63.88 0.0003529

[HIK|K] -63.89 0.0003526

[HK|G] -63.89 0.0003518

[HK|F] -63.89 0.0003518

[HK|E] -63.89 0.0003518

[HK|D] -63.89 0.0003518

[HK|C] -63.89 0.0003518

[BD|A] -63.89 0.0003502

[BF|A] -63.89 0.0003502

[HJ|G] -63.91 0.0003449

[HJ|F] -63.91 0.0003449

[HJ|E] -63.91 0.0003449

[HJ|D] -63.91 0.0003449

[HJ|C] -63.91 0.0003449

[CH|G] -63.91 0.0003448

[CH|F] -63.91 0.0003448

[EH|G] -63.91 0.0003448

[EH|F] -63.91 0.0003448

[CH|E] -63.91 0.0003448

[EH|D] -63.91 0.0003448

[CH|D] -63.91 0.0003448

[EH|C] -63.91 0.0003448

[HIJ|J] -63.91 0.0003434

[GH|F] -63.92 0.0003415

[GH|E] -63.92 0.0003415

[GH|D] -63.92 0.0003415

[GH|C] -63.92 0.0003415

[H|BD] -63.92 0.0003403

[H|BG] -63.92 0.0003403

[H|BE] -63.92 0.0003403

[H|BF] -63.92 0.0003403

[H|BC] -63.92 0.0003403

[CJ|B] -63.92 0.0003398

[EJ|B] -63.92 0.0003398

[FH|G] -63.93 0.0003384

[DH|G] -63.93 0.0003384

[DH|F] -63.93 0.0003384

[FH|E] -63.93 0.0003384

[FH|D] -63.93 0.0003384

[DH|E] -63.93 0.0003384

[FH|C] -63.93 0.0003384

[DH|C] -63.93 0.0003384

[GJ|B] -63.94 0.0003338

[CHI|C] -63.94 0.0003321

[EHI|E] -63.94 0.0003321

[CL|K] -63.95 0.0003293

[CL|J] -63.95 0.0003293

[EL|K] -63.95 0.0003293

[EL|J] -63.95 0.0003293

[GHI|G] -63.95 0.0003291

[DJ|B] -63.96 0.0003281

[FJ|B] -63.96 0.0003281

[DHI|D] -63.96 0.0003262

[FHI|F] -63.96 0.0003262

[L|BD] -63.97 0.0003252

[L|BG] -63.97 0.0003252

[L|BE] -63.97 0.0003252

[L|BF] -63.97 0.0003252

[L|BC] -63.97 0.0003252

[GL|K] -63.97 0.0003246

[GL|J] -63.97 0.0003246

[CK|B] -63.97 0.0003236

[EK|B] -63.97 0.0003236

[DL|K] -63.98 0.0003201

[FL|K] -63.98 0.0003201

[DL|J] -63.98 0.0003201

[FL|J] -63.98 0.0003201

[GK|B] -63.99 0.0003188

[C|AB] -63.99 0.0003185

[E|AB] -63.99 0.0003185

[CL|G] -64 0.0003157

[CL|F] -64 0.0003157

[CL|E] -64 0.0003157

[CL|D] -64 0.0003157

[EL|G] -64 0.0003157

[EL|F] -64 0.0003157

[EL|D] -64 0.0003157

[EL|C] -64 0.0003157

[G|AB] -64 0.0003145

[FK|B] -64 0.0003142

[DK|B] -64 0.0003142

[EHJ|J] -64.01 0.0003119

[CHJ|J] -64.01 0.0003119

[GL|F] -64.01 0.0003112

[GL|E] -64.01 0.0003112

[GL|D] -64.01 0.0003112

[GL|C] -64.01 0.0003112

[D|AB] -64.01 0.0003106

[F|AB] -64.01 0.0003106

[EHK|K] -64.01 0.0003099

[CHK|K] -64.01 0.0003099

[CEL|L] -64.02 0.0003086

[GHJ|J] -64.02 0.0003079

[FL|G] -64.02 0.0003069

[DL|G] -64.02 0.0003069

[DL|F] -64.02 0.0003069

[DL|E] -64.02 0.0003069

[FL|E] -64.02 0.0003069

[FL|D] -64.02 0.0003069

[FL|C] -64.02 0.0003069

[DL|C] -64.02 0.0003069

[CE|L] -64.02 0.0003067

[GHK|K] -64.03 0.0003065

[CGL|L] -64.03 0.0003052

[EGL|L] -64.03 0.0003052

[DHJ|J] -64.03 0.0003041

[FHJ|J] -64.03 0.0003041

[CG|L] -64.03 0.0003037

[EG|L] -64.03 0.0003037

[FHK|K] -64.04 0.0003033

[DHK|K] -64.04 0.0003033

[C|JL] -64.04 0.0003025

[E|JL] -64.04 0.0003025

[C|KL] -64.04 0.0003025

[E|KL] -64.04 0.0003025

[CFL|L] -64.04 0.0003019

[CDL|L] -64.04 0.0003019

[EFL|L] -64.04 0.0003019

[DEL|L] -64.04 0.0003019

[CEH|E] -64.04 0.0003009

[CEH|C] -64.04 0.0003009

[CF|L] -64.04 0.0003008

[CD|L] -64.04 0.0003008

[DE|L] -64.04 0.0003008

[EF|L] -64.04 0.0003008

[EHJ|E] -64.05 0.000299

[CHJ|C] -64.05 0.000299

[G|JL] -64.05 0.0002986

[G|KL] -64.05 0.0002986

[L|JK] -64.05 0.0002984

[CGH|G] -64.05 0.0002983

[EGH|G] -64.05 0.0002983

[CGH|C] -64.05 0.0002983

[EGH|E] -64.05 0.0002983

[DGL|L] -64.05 0.0002982

[FGL|L] -64.05 0.0002982

[DG|L] -64.05 0.0002975

[FG|L] -64.05 0.0002975

[EHK|E] -64.06 0.0002971

[CHK|C] -64.06 0.0002971

[CJ|L] -64.06 0.0002964

[EJ|L] -64.06 0.0002964

[CFH|F] -64.06 0.0002956

[EFH|F] -64.06 0.0002956

[CDH|D] -64.06 0.0002956

[EFH|E] -64.06 0.0002956

[CFH|C] -64.06 0.0002956

[CDH|C] -64.06 0.0002956

[DEH|E] -64.06 0.0002956

[DEH|D] -64.06 0.0002956

[GHJ|G] -64.06 0.0002952

[D|JL] -64.06 0.000295

[F|JL] -64.06 0.000295

[D|KL] -64.06 0.000295

[F|KL] -64.06 0.000295

[DFL|L] -64.06 0.0002949

[DF|L] -64.06 0.0002946

[GHK|G] -64.07 0.0002938

[CE|B] -64.07 0.0002931

[CE|K] -64.07 0.000293

[CE|J] -64.07 0.000293

[FGH|G] -64.07 0.0002928

[FGH|F] -64.07 0.0002928

[DGH|G] -64.07 0.0002928

[DGH|D] -64.07 0.0002928

[HJK|K] -64.07 0.000292

[HJK|J] -64.07 0.000292

[FHJ|F] -64.08 0.0002916

[DHJ|D] -64.08 0.0002916

[GJ|L] -64.08 0.0002911

[FHK|F] -64.08 0.0002907

[DHK|D] -64.08 0.0002907

[CG|B] -64.08 0.0002902

[EG|B] -64.08 0.0002902

[DFH|F] -64.08 0.0002902

[DFH|D] -64.08 0.0002902

[CG|K] -64.08 0.0002901

[CG|J] -64.08 0.0002901

[EG|K] -64.08 0.0002901

[EG|J] -64.08 0.0002901

[C|JK] -64.09 0.0002885

[E|JK] -64.09 0.0002885

[CF|B] -64.09 0.0002874

[CD|B] -64.09 0.0002874

[DE|B] -64.09 0.0002874

[EF|B] -64.09 0.0002874

[CF|K] -64.09 0.0002873

[CD|K] -64.09 0.0002873

[CF|J] -64.09 0.0002873

[CD|J] -64.09 0.0002873

[DE|K] -64.09 0.0002873

[EF|K] -64.09 0.0002873

[EF|J] -64.09 0.0002873

[DE|J] -64.09 0.0002873

[DJ|L] -64.09 0.0002862

[FJ|L] -64.09 0.0002862

[C|EL] -64.09 0.000286

[C|GL] -64.09 0.000286

[C|FL] -64.09 0.000286

[E|GL] -64.09 0.000286

[E|FL] -64.09 0.000286

[C|DL] -64.09 0.000286

[E|DL] -64.09 0.000286

[E|CL] -64.09 0.000286

[G|JK] -64.1 0.0002848

[DG|B] -64.1 0.0002843

[FG|B] -64.1 0.0002843

[DG|K] -64.1 0.0002842

[DG|J] -64.1 0.0002842

[FG|K] -64.1 0.0002842

[FG|J] -64.1 0.0002842

[CJL|L] -64.1 0.0002837

[EJL|L] -64.1 0.0002837

[CJ|K] -64.1 0.0002831

[EJ|K] -64.1 0.0002831

[CEL|E] -64.11 0.0002826

[CEL|C] -64.11 0.0002826

[G|EL] -64.11 0.0002823

[G|FL] -64.11 0.0002823

[G|DL] -64.11 0.0002823

[G|CL] -64.11 0.0002823

[CK|L] -64.11 0.0002823

[EK|L] -64.11 0.0002823

[L|FK] -64.11 0.0002822

[L|GJ] -64.11 0.0002822

[L|GK] -64.11 0.0002822

[L|FJ] -64.11 0.0002822

[L|EK] -64.11 0.0002822

[L|EJ] -64.11 0.0002822

[L|DJ] -64.11 0.0002822

[L|DK] -64.11 0.0002822

[L|CK] -64.11 0.0002822

[L|CJ] -64.11 0.0002822

[L|DE] -64.11 0.0002817

[L|EG] -64.11 0.0002817

[L|FG] -64.11 0.0002817

[L|EF] -64.11 0.0002817

[L|DF] -64.11 0.0002817

[L|DG] -64.11 0.0002817

[L|CF] -64.11 0.0002817

[L|CG] -64.11 0.0002817

[L|CE] -64.11 0.0002817

[L|CD] -64.11 0.0002817

[DF|B] -64.11 0.0002815

[DF|K] -64.11 0.0002814

[DF|J] -64.11 0.0002814

[D|JK] -64.11 0.0002813

[F|JK] -64.11 0.0002813

[CE|G] -64.11 0.0002809

[CE|F] -64.11 0.0002809

[CE|D] -64.11 0.0002809

[CGL|G] -64.12 0.0002795

[CGL|C] -64.12 0.0002795

[EGL|G] -64.12 0.0002795

[EGL|E] -64.12 0.0002795

[GJL|L] -64.12 0.0002791

[JL|K] -64.12 0.0002789

[D|EL] -64.12 0.0002789

[D|GL] -64.12 0.0002789

[F|EL] -64.12 0.0002789

[D|FL] -64.12 0.0002789

[F|GL] -64.12 0.0002789

[F|DL] -64.12 0.0002789

[D|CL] -64.12 0.0002789

[F|CL] -64.12 0.0002789

[CG|F] -64.12 0.0002782

[CG|E] -64.12 0.0002782

[CG|D] -64.12 0.0002782

[EG|F] -64.12 0.0002782

[EG|D] -64.12 0.0002782

[EG|C] -64.12 0.0002782

[GJ|K] -64.12 0.0002781

[GK|L] -64.12 0.000278

[CFL|F] -64.13 0.0002764

[CDL|D] -64.13 0.0002764

[CFL|C] -64.13 0.0002764

[CDL|C] -64.13 0.0002764

[EFL|F] -64.13 0.0002764

[EFL|E] -64.13 0.0002764

[DEL|E] -64.13 0.0002764

[DEL|D] -64.13 0.0002764

[CF|G] -64.13 0.0002755

[CD|G] -64.13 0.0002755

[CD|F] -64.13 0.0002755

[CF|E] -64.13 0.0002755

[CF|D] -64.13 0.0002755

[CD|E] -64.13 0.0002755

[DE|G] -64.13 0.0002755

[DE|F] -64.13 0.0002755

[EF|G] -64.13 0.0002755

[EF|D] -64.13 0.0002755

[DE|C] -64.13 0.0002755

[EF|C] -64.13 0.0002755

[DJL|L] -64.13 0.0002748

[FJL|L] -64.13 0.0002748

[FK|L] -64.14 0.000274

[DK|L] -64.14 0.000274

[CKL|L] -64.14 0.0002735

[EKL|L] -64.14 0.0002735

[DJ|K] -64.14 0.0002734

[FJ|K] -64.14 0.0002734

[DGL|G] -64.14 0.0002731

[FGL|G] -64.14 0.0002731

[FGL|F] -64.14 0.0002731

[DGL|D] -64.14 0.0002731

[C|FK] -64.14 0.0002728

[E|FK] -64.14 0.0002728

[C|GJ] -64.14 0.0002728

[C|GK] -64.14 0.0002728

[C|FJ] -64.14 0.0002728

[C|EK] -64.14 0.0002728

[C|EJ] -64.14 0.0002728

[E|GJ] -64.14 0.0002728

[C|DJ] -64.14 0.0002728

[E|GK] -64.14 0.0002728

[E|FJ] -64.14 0.0002728

[C|DK] -64.14 0.0002728

[E|DJ] -64.14 0.0002728

[E|DK] -64.14 0.0002728

[E|CK] -64.14 0.0002728

[E|CJ] -64.14 0.0002728

[KL|J] -64.14 0.0002726

[DG|F] -64.14 0.0002725

[DG|E] -64.14 0.0002725

[FG|E] -64.14 0.0002725

[FG|D] -64.14 0.0002725

[DG|C] -64.14 0.0002725

[FG|C] -64.14 0.0002725

[C|DE] -64.14 0.0002723

[C|EG] -64.14 0.0002723

[C|FG] -64.14 0.0002723

[E|FG] -64.14 0.0002723

[C|EF] -64.14 0.0002723

[C|DF] -64.14 0.0002723

[C|DG] -64.14 0.0002723

[E|DF] -64.14 0.0002723

[E|DG] -64.14 0.0002723

[E|CF] -64.14 0.0002723

[E|CG] -64.14 0.0002723

[E|CD] -64.14 0.0002723

[L|BGL] -64.15 0.0002718

[L|BFL] -64.15 0.0002718

[L|BEL] -64.15 0.0002718

[L|BDL] -64.15 0.0002718

[L|BCL] -64.15 0.0002718

[L|JKL] -64.15 0.0002715

[CJ|G] -64.15 0.0002714

[CJ|F] -64.15 0.0002714

[CJ|E] -64.15 0.0002714

[CJ|D] -64.15 0.0002714

[EJ|G] -64.15 0.0002714

[EJ|F] -64.15 0.0002714

[EJ|D] -64.15 0.0002714

[EJ|C] -64.15 0.0002714

[CJL|J] -64.15 0.000271

[EJL|J] -64.15 0.000271

[DFL|F] -64.15 0.0002701

[DFL|D] -64.15 0.0002701

[DF|G] -64.15 0.0002698

[DF|E] -64.15 0.0002698

[DF|C] -64.15 0.0002698

[CK|J] -64.15 0.0002696

[EK|J] -64.15 0.0002696

[GKL|L] -64.15 0.0002695

[G|FK] -64.15 0.0002693

[G|FJ] -64.15 0.0002693

[G|EK] -64.15 0.0002693

[G|EJ] -64.15 0.0002693

[G|DJ] -64.15 0.0002693

[G|DK] -64.15 0.0002693

[G|CK] -64.15 0.0002693

[G|CJ] -64.15 0.0002693

[G|DE] -64.16 0.0002688

[G|EF] -64.16 0.0002688

[G|DF] -64.16 0.0002688

[G|CF] -64.16 0.0002688

[G|CE] -64.16 0.0002688

[G|CD] -64.16 0.0002688

[J|BD] -64.16 0.000268

[J|BG] -64.16 0.000268

[J|BE] -64.16 0.000268

[J|BF] -64.16 0.000268

[J|BC] -64.16 0.000268

[JL|G] -64.16 0.0002674

[JL|F] -64.16 0.0002674

[JL|E] -64.16 0.0002674

[JL|D] -64.16 0.0002674

[JL|C] -64.16 0.0002674

[GJ|F] -64.16 0.0002666

[GJ|E] -64.16 0.0002666

[GJ|D] -64.16 0.0002666

[GJ|C] -64.16 0.0002666

[GJL|J] -64.16 0.0002666

[D|FK] -64.17 0.000266

[D|GJ] -64.17 0.000266

[D|GK] -64.17 0.000266

[D|FJ] -64.17 0.000266

[D|EK] -64.17 0.000266

[D|EJ] -64.17 0.000266

[F|GK] -64.17 0.000266

[F|GJ] -64.17 0.000266

[F|EK] -64.17 0.000266

[F|EJ] -64.17 0.000266

[F|DJ] -64.17 0.000266

[F|DK] -64.17 0.000266

[D|CK] -64.17 0.000266

[D|CJ] -64.17 0.000266

[F|CK] -64.17 0.000266

[F|CJ] -64.17 0.000266

[DKL|L] -64.17 0.0002658

[FKL|L] -64.17 0.0002658

[GK|J] -64.17 0.0002656

[D|EG] -64.17 0.0002655

[D|FG] -64.17 0.0002655

[F|DE] -64.17 0.0002655

[F|EG] -64.17 0.0002655

[D|EF] -64.17 0.0002655

[D|CF] -64.17 0.0002655

[F|DG] -64.17 0.0002655

[D|CG] -64.17 0.0002655

[F|CG] -64.17 0.0002655

[D|CE] -64.17 0.0002655

[F|CD] -64.17 0.0002655

[F|CE] -64.17 0.0002655

[E|BEL] -64.18 0.0002627

[C|BCL] -64.18 0.0002627

[DJL|J] -64.18 0.0002624

[FJL|J] -64.18 0.0002624

[DJ|G] -64.18 0.0002621

[DJ|F] -64.18 0.0002621

[DJ|E] -64.18 0.0002621

[FJ|G] -64.18 0.0002621

[FJ|E] -64.18 0.0002621

[DJ|C] -64.18 0.0002621

[FJ|D] -64.18 0.0002621

[FJ|C] -64.18 0.0002621

[C|BD] -64.18 0.0002619

[C|BG] -64.18 0.0002619

[E|BD] -64.18 0.0002619

[C|BE] -64.18 0.0002619

[E|BG] -64.18 0.0002619

[C|BF] -64.18 0.0002619

[E|BF] -64.18 0.0002619

[E|BC] -64.18 0.0002619

[FK|J] -64.18 0.0002618

[DK|J] -64.18 0.0002618

[KL|G] -64.18 0.0002614

[KL|F] -64.18 0.0002614

[KL|E] -64.18 0.0002614

[KL|D] -64.18 0.0002614

[KL|C] -64.18 0.0002614

[CKL|K] -64.19 0.0002612

[EKL|K] -64.19 0.0002612

[L|FKL] -64.19 0.0002602

[L|GJL] -64.19 0.0002602

[L|FJL] -64.19 0.0002602

[L|EKL] -64.19 0.0002602

[L|GKL] -64.19 0.0002602

[L|EJL] -64.19 0.0002602

[L|DJL] -64.19 0.0002602

[L|DKL] -64.19 0.0002602

[L|CJL] -64.19 0.0002602

[L|CKL] -64.19 0.0002602

[K|BD] -64.19 0.0002602

[K|BG] -64.19 0.0002602

[K|BE] -64.19 0.0002602

[K|BF] -64.19 0.0002602

[K|BC] -64.19 0.0002602

[CJL|C] -64.19 0.0002598

[EJL|E] -64.19 0.0002598

[G|BGL] -64.19 0.0002594

[G|BD] -64.2 0.0002586

[G|BE] -64.2 0.0002586

[G|BF] -64.2 0.0002586

[G|BC] -64.2 0.0002586

[CK|G] -64.2 0.0002585

[CK|F] -64.2 0.0002585

[CK|E] -64.2 0.0002585

[CK|D] -64.2 0.0002585

[EK|G] -64.2 0.0002585

[EK|F] -64.2 0.0002585

[EK|D] -64.2 0.0002585

[EK|C] -64.2 0.0002585

[J|KL] -64.2 0.0002579

[GKL|K] -64.2 0.0002574

[D|BDL] -64.2 0.0002562

[F|BFL] -64.2 0.0002562

[GJL|G] -64.21 0.0002556

[D|BG] -64.21 0.0002554

[F|BD] -64.21 0.0002554

[D|BE] -64.21 0.0002554

[D|BF] -64.21 0.0002554

[F|BG] -64.21 0.0002554

[F|BE] -64.21 0.0002554

[D|BC] -64.21 0.0002554

[F|BC] -64.21 0.0002554

[C|ABC] -64.21 0.0002551

[E|ABE] -64.21 0.0002551

[GK|F] -64.21 0.0002546

[GK|E] -64.21 0.0002546

[GK|D] -64.21 0.0002546

[GK|C] -64.21 0.0002546

[L|DEL] -64.21 0.0002543

[L|CGL] -64.21 0.0002543

[L|CFL] -64.21 0.0002543

[L|DFL] -64.21 0.0002543

[L|EFL] -64.21 0.0002543

[L|CEL] -64.21 0.0002543

[L|FGL] -64.21 0.0002543

[L|EGL] -64.21 0.0002543

[L|CDL] -64.21 0.0002543

[L|DGL] -64.21 0.0002543

[CEJ|J] -64.21 0.0002539

[DKL|K] -64.21 0.0002539

[FKL|K] -64.21 0.0002539

[G|ABG] -64.22 0.0002518

[DJL|D] -64.22 0.0002516

[FJL|F] -64.22 0.0002516

[E|EKL] -64.22 0.0002515

[E|EJL] -64.22 0.0002515

[C|CJL] -64.22 0.0002515

[C|CKL] -64.22 0.0002515

[FK|G] -64.23 0.000251

[DK|G] -64.23 0.000251

[DK|F] -64.23 0.000251

[FK|E] -64.23 0.000251

[FK|D] -64.23 0.000251

[DK|E] -64.23 0.000251

[FK|C] -64.23 0.000251

[DK|C] -64.23 0.000251

[C|BCK] -64.23 0.0002508

[E|BEJ] -64.23 0.0002508

[E|BEK] -64.23 0.0002508

[C|BCJ] -64.23 0.0002508

[CGJ|J] -64.23 0.0002506

[EGJ|J] -64.23 0.0002506

[CKL|C] -64.23 0.0002505

[EKL|E] -64.23 0.0002505

[K|JL] -64.23 0.0002504

[D|ABD] -64.23 0.0002488

[F|ABF] -64.23 0.0002488

[G|GJL] -64.24 0.0002483

[G|GKL] -64.24 0.0002483

[G|BGK] -64.24 0.0002476

[G|BGJ] -64.24 0.0002476

[CDJ|J] -64.24 0.0002474

[CFJ|J] -64.24 0.0002474

[EFJ|J] -64.24 0.0002474

[DEJ|J] -64.24 0.0002474

[GKL|G] -64.24 0.0002468

[C|CGL] -64.25 0.0002458

[C|CFL] -64.25 0.0002458

[E|DEL] -64.25 0.0002458

[C|CEL] -64.25 0.0002458

[E|EFL] -64.25 0.0002458

[E|CEL] -64.25 0.0002458

[C|CDL] -64.25 0.0002458

[E|EGL] -64.25 0.0002458

[F|FKL] -64.25 0.0002453

[F|FJL] -64.25 0.0002453

[D|DJL] -64.25 0.0002453

[D|DKL] -64.25 0.0002453

[F|BFK] -64.25 0.0002446

[F|BFJ] -64.25 0.0002446

[D|BDK] -64.25 0.0002446

[D|BDJ] -64.25 0.0002446

[CEG|G] -64.25 0.0002446

[CEG|E] -64.25 0.0002446

[CEG|C] -64.25 0.0002446

[DGJ|J] -64.25 0.000244

[FGJ|J] -64.25 0.000244

[J|EL] -64.25 0.0002439

[J|GL] -64.25 0.0002439

[J|FL] -64.25 0.0002439

[J|DL] -64.25 0.0002439

[J|CL] -64.25 0.0002439

[CEJ|E] -64.26 0.0002435

[CEJ|C] -64.26 0.0002435

[DKL|D] -64.26 0.0002434

[FKL|F] -64.26 0.0002434

[G|CGL] -64.26 0.0002427

[G|FGL] -64.26 0.0002427

[G|EGL] -64.26 0.0002427

[G|DGL] -64.26 0.0002427

[CDE|E] -64.26 0.0002424

[CDE|D] -64.26 0.0002424

[CEF|F] -64.26 0.0002424

[CEF|E] -64.26 0.0002424

[CDE|C] -64.26 0.0002424

[CEF|C] -64.26 0.0002424

[DFJ|J] -64.27 0.0002408

[CEK|K] -64.27 0.0002405

[CGJ|G] -64.27 0.0002403

[CGJ|C] -64.27 0.0002403

[EGJ|G] -64.27 0.0002403

[EGJ|E] -64.27 0.0002403

[CDG|G] -64.27 0.0002401

[CFG|G] -64.27 0.0002401

[CFG|F] -64.27 0.0002401

[CDG|D] -64.27 0.0002401

[EFG|G] -64.27 0.0002401

[EFG|F] -64.27 0.0002401

[EFG|E] -64.27 0.0002401

[DEG|G] -64.27 0.0002401

[CDG|C] -64.27 0.0002401

[DEG|E] -64.27 0.0002401

[CFG|C] -64.27 0.0002401

[DEG|D] -64.27 0.0002401

[D|DEL] -64.27 0.0002397

[F|CFL] -64.27 0.0002397

[D|DFL] -64.27 0.0002397

[F|DFL] -64.27 0.0002397

[F|EFL] -64.27 0.0002397

[D|CDL] -64.27 0.0002397

[F|FGL] -64.27 0.0002397

[D|DGL] -64.27 0.0002397

[JK|L] -64.27 0.0002397

[E|EJK] -64.28 0.0002386

[C|CJK] -64.28 0.0002386

[CDF|F] -64.28 0.0002379

[CDF|D] -64.28 0.0002379

[DEF|F] -64.28 0.0002379

[DEF|E] -64.28 0.0002379

[DEF|D] -64.28 0.0002379

[CDF|C] -64.28 0.0002379

[CGK|K] -64.28 0.0002378

[EGK|K] -64.28 0.0002378

[CDJ|D] -64.28 0.0002372

[CFJ|F] -64.28 0.0002372

[CDJ|C] -64.28 0.0002372

[EFJ|F] -64.28 0.0002372

[CFJ|C] -64.28 0.0002372

[EFJ|E] -64.28 0.0002372

[DEJ|E] -64.28 0.0002372

[DEJ|D] -64.28 0.0002372

[K|EL] -64.28 0.0002367

[K|GL] -64.28 0.0002367

[K|FL] -64.28 0.0002367

[K|DL] -64.28 0.0002367

[K|CL] -64.28 0.0002367

[G|GJK] -64.29 0.0002356

[DFG|G] -64.29 0.0002355

[DFG|F] -64.29 0.0002355

[DFG|D] -64.29 0.0002355

[CFK|K] -64.29 0.0002351

[CDK|K] -64.29 0.0002351

[EFK|K] -64.29 0.0002351

[DEK|K] -64.29 0.0002351

[DGJ|G] -64.3 0.0002339

At node N20:

split lnL Rel.Prob

[B|BHL] -58.11 0.1133

[B|ABH] -59.63 0.02488

[B|BHK] -59.78 0.02134

[B|HL] -60.05 0.01627

[H|BHL] -60.26 0.01323

[L|BHL] -60.4 0.01155

[BEJ|B] -60.48 0.01065

[BCJ|B] -60.48 0.01065

[BGJ|B] -60.58 0.00957

[BDJ|B] -60.7 0.008551

[BFJ|B] -60.7 0.008551

[A|ABH] -60.85 0.007366

[B|BHJ] -60.93 0.006803

[B|BH] -60.99 0.00637

[BCE|B] -61.18 0.0053

[A|BH] -61.18 0.005257

[BEG|B] -61.19 0.005202

[BCG|B] -61.19 0.005202

[C|BH] -61.2 0.005159

[E|BH] -61.2 0.005159

[BEF|B] -61.25 0.00492

[BCF|B] -61.25 0.00492

[BDE|B] -61.25 0.00492

[BCD|B] -61.25 0.00492

[ABE|B] -61.25 0.004905

[ABC|B] -61.25 0.004905

[B|ABE] -61.26 0.004862

[B|ABG] -61.26 0.004862

[B|ABF] -61.26 0.004862

[B|ABD] -61.26 0.004862

[B|ABC] -61.26 0.004862

[G|BH] -61.27 0.004827

[BE|H] -61.29 0.004731

[BC|H] -61.29 0.004731

[BCK|B] -61.32 0.004579

[BEK|B] -61.32 0.004579

[B|BDL] -61.32 0.004578

[B|BCL] -61.32 0.004578

[B|BEL] -61.32 0.004578

[B|BFL] -61.32 0.004578

[B|BGL] -61.32 0.004578

[BFG|B] -61.33 0.004535

[BDG|B] -61.33 0.004535

[ABG|B] -61.33 0.004523

[F|BH] -61.34 0.004515

[D|BH] -61.34 0.004515

[C|HL] -61.35 0.004468

[E|HL] -61.35 0.004468

[J|BH] -61.35 0.004457

[B|BHI] -61.35 0.004452

[BG|H] -61.36 0.004411

[BEL|B] -61.37 0.004339

[BCL|B] -61.37 0.004339

[BDF|B] -61.39 0.004291

[BGK|B] -61.41 0.004184

[G|HL] -61.41 0.00418

[ABD|B] -61.42 0.004165

[ABF|B] -61.42 0.004165

[BF|H] -61.43 0.004111

[BD|H] -61.43 0.004111

[B|HK] -61.46 0.003999

[BGL|B] -61.47 0.003958

[F|HL] -61.48 0.003911

[D|HL] -61.48 0.003911

[B|BEH] -61.5 0.003813

[B|BCH] -61.5 0.003813

[B|BGH] -61.5 0.003813

[B|BFH] -61.5 0.003813

[B|BDH] -61.5 0.003813

[BDK|B] -61.51 0.00381

[BFK|B] -61.51 0.00381

[AB|H] -61.54 0.003695

[BDL|B] -61.56 0.003599

[BFL|B] -61.56 0.003599

[BE|A] -61.57 0.003563

[BC|A] -61.57 0.003563

[B|B] -61.6 0.003479

[BE|L] -61.63 0.003365

[BC|L] -61.63 0.003365

[BG|A] -61.64 0.003322

[J|HL] -61.7 0.003144

[BG|L] -61.7 0.003138

[ABE|A] -61.71 0.003117

[ABC|A] -61.71 0.003117

[BF|A] -61.71 0.003096

[BD|A] -61.71 0.003096

[BCH|B] -61.72 0.00307

[BEH|B] -61.72 0.00307

[BF|L] -61.77 0.002925

[BD|L] -61.77 0.002925

[H|ABH] -61.78 0.002906

[ABG|A] -61.79 0.002875

[BGH|B] -61.8 0.002834

[BHJ|B] -61.82 0.002795

[BEH|H] -61.84 0.00273

[BCH|H] -61.84 0.00273

[ABD|A] -61.87 0.002647

[ABF|A] -61.87 0.002647

[BDH|B] -61.88 0.002613

[BFH|B] -61.88 0.002613

[B|AB] -61.89 0.002591

[E|AB] -61.9 0.002557

[C|AB] -61.9 0.002557

[BEL|L] -61.91 0.002534

[BCL|L] -61.91 0.002534

[BGH|H] -61.92 0.00252

[H|BHK] -61.93 0.002493

[BHJ|H] -61.93 0.002485

[G|AB] -61.97 0.002392

[BDH|H] -62 0.002324

[BFH|H] -62 0.002324

[BGL|L] -62.01 0.002311

[K|BHK] -62.01 0.002305

[F|AB] -62.04 0.002238

[D|AB] -62.04 0.002238

[BDL|L] -62.1 0.002101

[BFL|L] -62.1 0.002101

[K|BH] -62.11 0.002089

[L|BH] -62.16 0.001975

[BE|B] -62.18 0.001941

[BC|B] -62.18 0.001941

[BG|B] -62.25 0.00181

[EJ|B] -62.27 0.001772

[CJ|B] -62.27 0.001772

[BF|B] -62.32 0.001687

[BD|B] -62.32 0.001687

[GJ|B] -62.36 0.001614

[E|ABE] -62.36 0.001613

[C|ABC] -62.36 0.001613

[J|BHJ] -62.39 0.001568

[C|BCL] -62.42 0.001519

[E|BEL] -62.42 0.001519

[B|H] -62.43 0.001517

[G|ABG] -62.43 0.001509

[EJ|H] -62.44 0.001499

[CJ|H] -62.44 0.001499

[K|HL] -62.46 0.001474

[DJ|B] -62.46 0.001465

[FJ|B] -62.46 0.001465

[A|ABE] -62.48 0.00144

[A|ABG] -62.48 0.00144

[A|ABF] -62.48 0.00144

[A|ABC] -62.48 0.00144

[A|ABD] -62.48 0.00144

[G|BGL] -62.49 0.001421

[B|HJ] -62.49 0.00142

[F|ABF] -62.5 0.001412

[D|ABD] -62.5 0.001412

[GJ|H] -62.53 0.001366

[BH|L] -62.53 0.001365

[BH|A] -62.53 0.001362

[D|BDL] -62.56 0.00133

[F|BFL] -62.56 0.00133

[AB|B] -62.59 0.001289

[E|BEH] -62.61 0.001265

[C|BCH] -62.61 0.001265

[FJ|H] -62.63 0.00124

[DJ|H] -62.63 0.00124

[B|FL] -62.66 0.001206

[B|EL] -62.66 0.001206

[B|GL] -62.66 0.001206

[B|DL] -62.66 0.001206

[B|CL] -62.66 0.001206

[G|BGH] -62.67 0.001184

[B|HI] -62.69 0.00117

[BEJ|E] -62.73 0.001119

[BCJ|C] -62.73 0.001119

[F|BFH] -62.74 0.001107

[D|BDH] -62.74 0.001107

[C|HK] -62.75 0.001098

[E|HK] -62.75 0.001098

[G|HK] -62.82 0.001027

[C|CHL] -62.82 0.001019

[E|EHL] -62.82 0.001019

[ABH|B] -62.83 0.001017

[BGJ|G] -62.84 0.001006

[CE|B] -62.85 0.0009899

[CE|H] -62.86 0.0009849

[H|AB] -62.87 0.0009755

[EG|B] -62.87 0.0009684

[CG|B] -62.87 0.0009684

[EG|H] -62.88 0.0009635

[CG|H] -62.88 0.0009635

[F|HK] -62.88 0.0009611

[D|HK] -62.88 0.0009611

[B|FH] -62.89 0.0009557

[B|EH] -62.89 0.0009557

[B|GH] -62.89 0.0009557

[B|DH] -62.89 0.0009557

[B|CH] -62.89 0.0009557

[H|HKL] -62.89 0.0009547

[G|GHL] -62.89 0.0009533

[B|A] -62.91 0.0009374

[CD|B] -62.93 0.000921

[EF|B] -62.93 0.000921

[DE|B] -62.93 0.000921

[CF|B] -62.93 0.000921

[CJ|L] -62.93 0.0009198

[EJ|L] -62.93 0.0009198

[DE|H] -62.93 0.0009163

[CF|H] -62.93 0.0009163

[CD|H] -62.93 0.0009163

[EF|H] -62.93 0.0009163

[ABH|H] -62.94 0.0009047

[BFJ|F] -62.95 0.0008985

[BDJ|D] -62.95 0.0008985

[D|DHL] -62.96 0.0008918

[F|FHL] -62.96 0.0008918

[K|HKL] -62.97 0.0008828

[BE|K] -62.98 0.0008716

[BC|K] -62.98 0.0008716

[DG|B] -63 0.0008586

[FG|B] -63 0.0008586

[FG|H] -63 0.0008542

[DG|H] -63 0.0008542

[EK|B] -63.01 0.000842

[CK|B] -63.01 0.000842

[GJ|L] -63.02 0.0008379

[L|HKL] -63.02 0.0008335

[AB|A] -63.04 0.0008193

[DF|B] -63.05 0.0008168

[BG|K] -63.05 0.0008127

[DF|H] -63.05 0.0008126

[EL|B] -63.06 0.0008047

[CL|B] -63.06 0.0008047

[H|BHJ] -63.07 0.0007948

[GK|B] -63.09 0.0007786

[J|HK] -63.1 0.0007727

[BH|B] -63.11 0.0007692

[A|AB] -63.11 0.0007672

[FJ|L] -63.12 0.0007609

[DJ|L] -63.12 0.0007609

[BF|K] -63.12 0.0007575

[BD|K] -63.12 0.0007575

[H|BH] -63.14 0.0007442

[GL|B] -63.14 0.0007432

[BHK|B] -63.16 0.00073

[FK|B] -63.17 0.0007188

[DK|B] -63.17 0.0007188

[EK|H] -63.18 0.0007126

[CK|H] -63.18 0.0007126

[J|HJL] -63.2 0.0007028

[BCK|K] -63.21 0.0006937

[BEK|K] -63.21 0.0006937

[FL|B] -63.22 0.0006853

[DL|B] -63.22 0.0006853

[BH|H] -63.22 0.000684

[EL|H] -63.23 0.000681

[CL|H] -63.23 0.000681

[GK|H] -63.26 0.000659

[BHK|H] -63.27 0.0006493

[ABH|A] -63.28 0.0006465

[H|HL] -63.28 0.0006436

[A|A] -63.29 0.0006388

[BGK|K] -63.3 0.0006338

[GL|H] -63.31 0.0006289

[B|BE] -63.34 0.000611

[B|BF] -63.34 0.000611

[B|BG] -63.34 0.000611

[B|BD] -63.34 0.000611

[B|BC] -63.34 0.000611

[FK|H] -63.34 0.0006083

[DK|H] -63.34 0.0006083

[BEJ|J] -63.35 0.0006021

[BCJ|J] -63.35 0.0006021

[CE|L] -63.35 0.0005996

[CG|L] -63.38 0.0005865

[EG|L] -63.38 0.0005865

[FL|H] -63.39 0.0005799

[DL|H] -63.39 0.0005799

[HJ|B] -63.39 0.0005788

[BFK|K] -63.39 0.0005772

[BDK|K] -63.39 0.0005772

[L|HL] -63.42 0.0005619

[CD|L] -63.43 0.0005578

[CF|L] -63.43 0.0005578

[EF|L] -63.43 0.0005578

[DE|L] -63.43 0.0005578

[BCE|E] -63.43 0.0005569

[BCE|C] -63.43 0.0005569

[EH|B] -63.43 0.0005562

[CH|B] -63.43 0.0005562

[BEG|G] -63.45 0.0005466

[BEG|E] -63.45 0.0005466

[BCG|G] -63.45 0.0005466

[BCG|C] -63.45 0.0005466

[BGJ|J] -63.46 0.0005409

[A|BE] -63.49 0.0005226

[A|BG] -63.49 0.0005226

[A|BC] -63.49 0.0005226

[A|BF] -63.49 0.0005226

[A|BD] -63.49 0.0005226

[H|BHI] -63.5 0.0005202

[FG|L] -63.5 0.00052

[DG|L] -63.5 0.00052

[GH|B] -63.5 0.0005184

[BEF|F] -63.5 0.0005169

[BEF|E] -63.5 0.0005169

[BDE|E] -63.5 0.0005169

[BCF|F] -63.5 0.0005169

[BCD|C] -63.5 0.0005169

[BDE|D] -63.5 0.0005169

[BCF|C] -63.5 0.0005169

[BCD|D] -63.5 0.0005169

[ABE|E] -63.51 0.0005154

[ABC|C] -63.51 0.0005154

[E|H] -63.51 0.000513

[C|H] -63.51 0.000513

[BE|F] -63.51 0.0005122

[BC|E] -63.51 0.0005122

[BE|C] -63.51 0.0005122

[BE|G] -63.51 0.0005122

[BC|F] -63.51 0.0005122

[BC|D] -63.51 0.0005122

[BE|D] -63.51 0.0005122

[BC|G] -63.51 0.0005122

[C|BE] -63.52 0.0005092

[C|BG] -63.52 0.0005092

[E|BC] -63.52 0.0005092

[C|BD] -63.52 0.0005092

[E|BG] -63.52 0.0005092

[E|BF] -63.52 0.0005092

[C|BF] -63.52 0.0005092

[E|BD] -63.52 0.0005092

[E|B] -63.53 0.000504

[C|B] -63.53 0.000504

[DF|L] -63.55 0.0004947

[BHI|B] -63.56 0.0004874

[BDJ|J] -63.57 0.0004833

[BFJ|J] -63.57 0.0004833

[FH|B] -63.57 0.0004829

[DH|B] -63.57 0.0004829

[BEK|E] -63.57 0.0004811

[BCK|C] -63.57 0.0004811

[G|H] -63.58 0.0004799

[BG|F] -63.58 0.0004776

[BG|E] -63.58 0.0004776

[BG|D] -63.58 0.0004776

[BG|C] -63.58 0.0004776

[BFG|F] -63.58 0.0004765

[BFG|G] -63.58 0.0004765

[BDG|G] -63.58 0.0004765

[BDG|D] -63.58 0.0004765

[G|BE] -63.58 0.0004765

[G|BD] -63.58 0.0004765

[G|BC] -63.58 0.0004765

[G|BF] -63.58 0.0004765

[ABG|G] -63.59 0.0004752

[G|B] -63.59 0.0004716

[L|BDL] -63.6 0.000467

[L|BCL] -63.6 0.000467

[L|BEL] -63.6 0.000467

[L|BFL] -63.6 0.000467

[L|BGL] -63.6 0.000467

[BEL|E] -63.63 0.000456

[BCL|C] -63.63 0.000456

[BDF|F] -63.64 0.0004509

[BDF|D] -63.64 0.0004509

[E|KL] -63.64 0.0004491

[C|KL] -63.64 0.0004491

[F|H] -63.64 0.000449

[D|H] -63.64 0.000449

[H|HIL] -63.64 0.0004487

[D|BC] -63.65 0.0004457

[F|BE] -63.65 0.0004457

[F|BD] -63.65 0.0004457

[D|BF] -63.65 0.0004457

[D|BE] -63.65 0.0004457

[F|BC] -63.65 0.0004457

[D|BG] -63.65 0.0004457

[F|BG] -63.65 0.0004457

[H|BFH] -63.65 0.0004455

[H|BGH] -63.65 0.0004455

[H|BEH] -63.65 0.0004455

[H|BDH] -63.65 0.0004455

[H|BCH] -63.65 0.0004455

[BD|F] -63.65 0.0004452

[BF|E] -63.65 0.0004452

[BF|G] -63.65 0.0004452

[BD|G] -63.65 0.0004452

[BF|D] -63.65 0.0004452

[BD|E] -63.65 0.0004452

[BF|C] -63.65 0.0004452

[BD|C] -63.65 0.0004452

[F|B] -63.66 0.0004411

[D|B] -63.66 0.0004411

[BGK|G] -63.66 0.0004396

[ABF|F] -63.67 0.0004377

[ABD|D] -63.67 0.0004377

[EK|L] -63.67 0.0004372

[CK|L] -63.67 0.0004372

[A|B] -63.67 0.0004368

[B|BEG] -63.67 0.0004363

[B|BCE] -63.67 0.0004363

[B|BFG] -63.67 0.0004363

[B|BEF] -63.67 0.0004363

[B|BCG] -63.67 0.0004363

[B|BDG] -63.67 0.0004363

[B|BDF] -63.67 0.0004363

[B|BCD] -63.67 0.0004363

[B|BDE] -63.67 0.0004363

[B|BCF] -63.67 0.0004363

[BHI|H] -63.68 0.0004335

[H|H] -63.7 0.000426

[G|KL] -63.71 0.0004202

[J|BG] -63.72 0.0004175

[J|BE] -63.72 0.0004175

[J|BD] -63.72 0.0004175

[J|BF] -63.72 0.0004175

[J|BC] -63.72 0.0004175

[BGL|G] -63.72 0.0004158

[BHL|B] -63.72 0.0004151

[GK|L] -63.75 0.0004043

[AB|E] -63.75 0.0004029

[AB|F] -63.75 0.0004029

[AB|C] -63.75 0.0004029

[AB|G] -63.75 0.0004029

[AB|D] -63.75 0.0004029

[BFK|F] -63.76 0.0004003

[BDK|D] -63.76 0.0004003

[D|KL] -63.78 0.0003931

[F|KL] -63.78 0.0003931

[E|HI] -63.78 0.0003929

[C|HI] -63.78 0.0003929

[L|HIL] -63.78 0.0003917

[E|HJ] -63.78 0.0003899

[C|HJ] -63.78 0.0003899

[BFL|F] -63.82 0.0003781

[BDL|D] -63.82 0.0003781

[FK|L] -63.83 0.0003732

[DK|L] -63.83 0.0003732

[BHL|H] -63.84 0.0003691

[G|HI] -63.84 0.0003676

[G|HJ] -63.85 0.0003648

[J|H] -63.87 0.0003595

[H|CHL] -63.87 0.0003587

[H|DHL] -63.87 0.0003587

[H|GHL] -63.87 0.0003587

[H|FHL] -63.87 0.0003587

[H|EHL] -63.87 0.0003587

[H|HJL] -63.87 0.0003563

[BH|K] -63.88 0.0003537

[E|GL] -63.89 0.0003511

[C|GL] -63.89 0.0003511

[E|FL] -63.89 0.0003511

[C|FL] -63.89 0.0003511

[C|EL] -63.89 0.0003511

[C|DL] -63.89 0.0003511

[E|DL] -63.89 0.0003511

[E|CL] -63.89 0.0003511

[F|HI] -63.91 0.0003439

[D|HI] -63.91 0.0003439

[L|HK] -63.91 0.0003424

[EHJ|H] -63.92 0.0003419

[CHJ|H] -63.92 0.0003419

[F|HJ] -63.92 0.0003412

[D|HJ] -63.92 0.0003412

[EH|L] -63.94 0.0003348

[CH|L] -63.94 0.0003348

[G|EL] -63.96 0.0003285

[G|FL] -63.96 0.0003285

[G|CL] -63.96 0.0003285

[G|DL] -63.96 0.0003285

[BE|J] -63.97 0.0003252

[BC|J] -63.97 0.0003252

[C|FH] -63.97 0.0003251

[C|GH] -63.97 0.0003251

[C|EH] -63.97 0.0003251

[E|FH] -63.97 0.0003251

[E|GH] -63.97 0.0003251

[E|CH] -63.97 0.0003251

[E|DH] -63.97 0.0003251

[C|DH] -63.97 0.0003251

[BCH|C] -63.97 0.0003226

[BEH|E] -63.97 0.0003226

[J|KL] -63.99 0.0003186

[GHJ|H] -64 0.0003156

[L|DHL] -64 0.0003132

[L|CHL] -64 0.0003132

[L|GHL] -64 0.0003132

[L|FHL] -64 0.0003132

[L|EHL] -64 0.0003132

[GH|L] -64.01 0.000312

[L|HJL] -64.01 0.000311

[C|L] -64.02 0.0003074

[E|L] -64.02 0.0003074

[F|GL] -64.02 0.0003073

[F|EL] -64.02 0.0003073

[F|DL] -64.02 0.0003073

[D|FL] -64.02 0.0003073

[D|GL] -64.02 0.0003073

[D|CL] -64.02 0.0003073

[F|CL] -64.02 0.0003073

[D|EL] -64.02 0.0003073

[G|EH] -64.03 0.0003042

[G|FH] -64.03 0.0003042

[G|DH] -64.03 0.0003042

[G|CH] -64.03 0.0003042

[BG|J] -64.04 0.0003032

[B|BGK] -64.05 0.0003002

[B|BEK] -64.05 0.0003002

[B|BFK] -64.05 0.0003002

[B|BDK] -64.05 0.0003002

[B|BCK] -64.05 0.0003002

[BGH|G] -64.05 0.0002978

[DHJ|H] -64.08 0.0002909

[FHJ|H] -64.08 0.0002909

[FH|L] -64.08 0.0002907

[DH|L] -64.08 0.0002907

[G|L] -64.09 0.0002876

[B|BEJ] -64.09 0.0002861

[B|BGJ] -64.09 0.0002861

[B|BDJ] -64.09 0.0002861

[B|BCJ] -64.09 0.0002861

[B|BFJ] -64.09 0.0002861

[D|FH] -64.1 0.0002846

[D|EH] -64.1 0.0002846

[F|GH] -64.1 0.0002846

[F|EH] -64.1 0.0002846

[D|GH] -64.1 0.0002846

[F|CH] -64.1 0.0002846

[F|DH] -64.1 0.0002846

[D|CH] -64.1 0.0002846

[HJ|L] -64.1 0.0002838

[BD|J] -64.11 0.0002826

[BF|J] -64.11 0.0002826

[BDH|D] -64.14 0.0002745

[BFH|F] -64.14 0.0002745

[J|HI] -64.14 0.000274

[D|L] -64.16 0.000269

[F|L] -64.16 0.000269

[CEH|H] -64.16 0.0002668

[EGH|H] -64.19 0.0002593

[CGH|H] -64.19 0.0002593

[EFH|H] -64.24 0.0002473

[CFH|H] -64.24 0.0002473

[DEH|H] -64.24 0.0002473

[CDH|H] -64.24 0.0002473

[J|EL] -64.24 0.0002468

[J|FL] -64.24 0.0002468

[J|CL] -64.24 0.0002468

[J|GL] -64.24 0.0002468

[J|DL] -64.24 0.0002468

[BHL|L] -64.26 0.0002423

[EJ|K] -64.28 0.0002383

[CJ|K] -64.28 0.0002383

[JK|H] -64.3 0.000233

[DGH|H] -64.3 0.0002323

[FGH|H] -64.3 0.0002323

[JL|H] -64.32 0.0002287

[J|GH] -64.33 0.0002268

[J|FH] -64.33 0.0002268

[J|EH] -64.33 0.0002268

[J|DH] -64.33 0.0002268

[J|CH] -64.33 0.0002268

[HK|B] -64.34 0.0002236

[DFH|H] -64.35 0.0002214

[L|L] -64.36 0.0002193

[J|L] -64.37 0.0002176

[GJ|K] -64.37 0.0002171

[C|CHK] -64.41 0.0002085

[E|EHK] -64.41 0.0002085

[EH|H] -64.42 0.0002066

[CH|H] -64.42 0.0002066

[BE|E] -64.43 0.0002039

[BC|C] -64.43 0.0002039

[C|BC] -64.44 0.0002028

[E|BE] -64.44 0.0002028

[EJL|L] -64.44 0.0002017

[CJL|L] -64.44 0.0002017

[H|B] -64.46 0.0001993

[BH|F] -64.47 0.0001972

[BH|G] -64.47 0.0001972

[BH|E] -64.47 0.0001972

[BH|C] -64.47 0.0001972

[BH|D] -64.47 0.0001972

[DJ|K] -64.47 0.0001971

[FJ|K] -64.47 0.0001971

[K|BE] -64.47 0.0001957

[K|BC] -64.47 0.0001957

[K|BF] -64.47 0.0001957

[K|BG] -64.47 0.0001957

[K|BD] -64.47 0.0001957

[H|BC] -64.47 0.0001956

[H|BG] -64.47 0.0001956

[H|BF] -64.47 0.0001956

[H|BE] -64.47 0.0001956

[H|BD] -64.47 0.0001956

[G|GHK] -64.48 0.0001951

[GH|H] -64.49 0.0001925

[CEL|L] -64.5 0.0001907

[E|JL] -64.5 0.0001905

[C|JL] -64.5 0.0001905

[BG|G] -64.5 0.0001902

[G|BG] -64.51 0.0001897

[B|FK] -64.51 0.0001896

[B|GK] -64.51 0.0001896

[B|EK] -64.51 0.0001896

[B|DK] -64.51 0.0001896

[B|CK] -64.51 0.0001896

[L|BE] -64.53 0.0001851

[L|BF] -64.53 0.0001851

[L|BG] -64.53 0.0001851

[L|BD] -64.53 0.0001851

[L|BC] -64.53 0.0001851

[EGL|L] -64.53 0.0001847

[CGL|L] -64.53 0.0001847

[GJL|L] -64.54 0.0001841

[D|DHK] -64.54 0.0001825

[F|FHK] -64.54 0.0001825

[HL|B] -64.56 0.0001798

[FH|H] -64.56 0.0001793

[DH|H] -64.56 0.0001793

[G|JL] -64.57 0.0001782

[F|BF] -64.57 0.0001775

[D|BD] -64.57 0.0001775

[BF|F] -64.57 0.0001772

[BD|D] -64.57 0.0001772

[CHK|H] -64.58 0.0001764

[EHK|H] -64.58 0.0001764

[EFL|L] -64.58 0.0001761

[CFL|L] -64.58 0.0001761

[DEL|L] -64.58 0.0001761

[CDL|L] -64.58 0.0001761

[HJ|H] -64.59 0.0001743

[H|KL] -64.6 0.0001717

[K|H] -64.62 0.0001685

[FJL|L] -64.63 0.0001675

[DJL|L] -64.63 0.0001675

[F|JL] -64.63 0.0001667

[D|JL] -64.63 0.0001667

[CHL|H] -64.64 0.000166

[EHL|H] -64.64 0.000166

[FGL|L] -64.64 0.0001655

[DGL|L] -64.64 0.0001655

[CL|L] -64.64 0.0001653

[EL|L] -64.64 0.0001653

[GHK|H] -64.65 0.0001636

[EJ|F] -64.65 0.0001633

[CJ|F] -64.65 0.0001633

[EJ|C] -64.65 0.0001633

[EJ|G] -64.65 0.0001633

[CJ|E] -64.65 0.0001633

[EJ|D] -64.65 0.0001633

[CJ|G] -64.65 0.0001633

[CJ|D] -64.65 0.0001633

[B|F] -64.68 0.0001596

[B|E] -64.68 0.0001596

[B|G] -64.68 0.0001596

[B|D] -64.68 0.0001596

[B|C] -64.68 0.0001596

[L|H] -64.68 0.0001593

[H|HK] -64.69 0.0001582

[BHJ|J] -64.69 0.000158

[DFL|L] -64.69 0.0001575

[CE|K] -64.71 0.0001553

[GHL|H] -64.72 0.0001535

[J|HJK] -64.72 0.0001534

[GL|L] -64.72 0.0001526

[CG|K] -64.73 0.0001519

[EG|K] -64.73 0.0001519

[FHK|H] -64.73 0.0001516

[DHK|H] -64.73 0.0001516

[GJ|E] -64.75 0.0001488

[GJ|F] -64.75 0.0001488

[GJ|D] -64.75 0.0001488

[GJ|C] -64.75 0.0001488

[K|HK] -64.76 0.0001463

[C|BCD] -64.78 0.0001448

[C|BCE] -64.78 0.0001448

[E|BEG] -64.78 0.0001448

[C|BCG] -64.78 0.0001448

[E|BCE] -64.78 0.0001448

[C|BCF] -64.78 0.0001448

[E|BEF] -64.78 0.0001448

[E|BDE] -64.78 0.0001448

[CD|K] -64.78 0.0001445

[DE|K] -64.78 0.0001445

[CF|K] -64.78 0.0001445

[EF|K] -64.78 0.0001445

[JK|L] -64.78 0.0001441

[FHL|H] -64.8 0.0001418

[DHL|H] -64.8 0.0001418

[C|CL] -64.8 0.0001408

[E|EL] -64.8 0.0001408

[FL|L] -64.8 0.0001408

[DL|L] -64.8 0.0001408

[KL|H] -64.84 0.0001361

[G|BEG] -64.84 0.0001354

[G|BFG] -64.84 0.0001354

[G|BCG] -64.84 0.0001354

At node N12:

split lnL Rel.Prob

[CJ|B] -58.71 0.06234

[EJ|B] -58.71 0.06234

[GJ|B] -58.83 0.05514

[DJ|B] -58.96 0.0484

[FJ|B] -58.96 0.0484

[EG|B] -59.54 0.02723

[CG|B] -59.54 0.02723

[CE|B] -59.63 0.02474

[EF|B] -59.67 0.02384

[DE|B] -59.67 0.02384

[CF|B] -59.67 0.02384

[CD|B] -59.67 0.02384

[E|B] -59.7 0.02325

[C|B] -59.7 0.02325

[G|B] -59.82 0.02062

[F|B] -59.94 0.01817

[D|B] -59.94 0.01817

[DG|B] -59.97 0.01771

[FG|B] -59.97 0.01771

[BEJ|B] -60.02 0.01685

[BCJ|B] -60.02 0.01685

[DF|B] -60.1 0.01549

[BGJ|B] -60.16 0.01465

[BFJ|B] -60.32 0.01252

[BDJ|B] -60.32 0.01252

[CK|B] -60.68 0.008708

[EK|B] -60.68 0.008708

[GK|B] -60.79 0.007788

[FK|B] -60.91 0.006892

[DK|B] -60.91 0.006892

[HJ|B] -60.92 0.006829

[BEG|B] -60.96 0.006565

[BCG|B] -60.96 0.006565

[J|BH] -60.98 0.00646

[E|AB] -61.06 0.005918

[C|AB] -61.06 0.005918

[BCE|B] -61.07 0.005891

[BE|B] -61.08 0.00583

[BC|B] -61.08 0.00583

[BDE|B] -61.1 0.005707

[BCF|B] -61.1 0.005707

[BCD|B] -61.1 0.005707

[BEF|B] -61.1 0.005707

[G|AB] -61.18 0.005248

[BG|B] -61.21 0.005132

[D|AB] -61.31 0.004625

[F|AB] -61.31 0.004625

[BF|B] -61.34 0.004472

[BD|B] -61.34 0.004472

[BDG|B] -61.42 0.004144

[BFG|B] -61.42 0.004144

[J|BG] -61.46 0.003979

[J|BF] -61.46 0.003979

[J|BE] -61.46 0.003979

[J|BD] -61.46 0.003979

[J|BC] -61.46 0.003979

[EL|B] -61.56 0.003593

[CL|B] -61.56 0.003593

[BDF|B] -61.56 0.003591

[GL|B] -61.69 0.003178

[FL|B] -61.82 0.002784

[DL|B] -61.82 0.002784

[E|BD] -62.04 0.002242

[E|BF] -62.04 0.002242

[E|BG] -62.04 0.002242

[E|BC] -62.04 0.002242

[C|BG] -62.04 0.002242

[C|BE] -62.04 0.002242

[C|BF] -62.04 0.002242

[C|BD] -62.04 0.002242

[G|BD] -62.16 0.001989

[G|BF] -62.16 0.001989

[G|BE] -62.16 0.001989

[G|BC] -62.16 0.001989

[E|BE] -62.16 0.001975

[C|BC] -62.16 0.001975

[BCK|B] -62.18 0.001946

[BEK|B] -62.18 0.001946

[CH|B] -62.18 0.001935

[EH|B] -62.18 0.001935

[E|BH] -62.19 0.001919

[C|BH] -62.19 0.001919

[F|BG] -62.28 0.001752

[F|BE] -62.28 0.001752

[D|BG] -62.28 0.001752

[F|BD] -62.28 0.001752

[D|BF] -62.28 0.001752

[D|BE] -62.28 0.001752

[F|BC] -62.28 0.001752

[D|BC] -62.28 0.001752

[G|BG] -62.28 0.001752

[BGK|B] -62.31 0.001711

[GH|B] -62.31 0.001711

[G|BH] -62.31 0.001702

[F|BF] -62.41 0.001544

[D|BD] -62.41 0.001544

[F|BH] -62.44 0.0015

[D|BH] -62.44 0.0015

[FH|B] -62.44 0.001498

[DH|B] -62.44 0.001498

[BFK|B] -62.45 0.001477

[BDK|B] -62.45 0.001477

[C|ABC] -62.48 0.001436

[E|ABE] -62.48 0.001436

At node N10:

split lnL Rel.Prob

[EG|J] -58.08 0.1175

[CG|J] -58.08 0.1175

[CD|J] -58.28 0.09587

[DE|J] -58.28 0.09587

[EF|J] -58.28 0.09587

[CF|J] -58.28 0.09587

[CE|J] -58.39 0.08571

[FG|J] -58.94 0.04956

[DG|J] -58.94 0.04956

[DF|J] -59.2 0.03838

[EGJ|J] -61.01 0.00625

[CGJ|J] -61.01 0.00625

[E|J] -61.05 0.006027

[C|J] -61.05 0.006027

[G|J] -61.17 0.005304

[CDJ|J] -61.29 0.004747

[DEJ|J] -61.29 0.004747

[CFJ|J] -61.29 0.004747

[EFJ|J] -61.29 0.004747

[F|J] -61.37 0.004344

[D|J] -61.37 0.004344

[CEJ|J] -61.71 0.003105

[FGJ|J] -62.01 0.002301

[DGJ|J] -62.01 0.002301

[CEG|G] -62.08 0.00214

[CEG|E] -62.08 0.00214

[CEG|C] -62.08 0.00214

[DFJ|J] -62.3 0.001725

[CDE|E] -62.33 0.001678

[CDE|D] -62.33 0.001678

[CDE|C] -62.33 0.001678

[CEF|F] -62.33 0.001678

[CEF|E] -62.33 0.001678

[CEF|C] -62.33 0.001678

[CFG|G] -62.48 0.001441

[DEG|G] -62.48 0.001441

[CFG|F] -62.48 0.001441

[CFG|C] -62.48 0.001441

[DEG|E] -62.48 0.001441

[EFG|G] -62.48 0.001441

[CDG|G] -62.48 0.001441

[EFG|F] -62.48 0.001441

[EFG|E] -62.48 0.001441

[DEG|D] -62.48 0.001441

[CDG|C] -62.48 0.001441

[CDG|D] -62.48 0.001441

[EK|J] -62.71 0.001147

[CK|J] -62.71 0.001147

[GK|J] -62.75 0.001101

[CDF|F] -62.75 0.001095

[CDF|C] -62.75 0.001095

[CDF|D] -62.75 0.001095

[DEF|E] -62.75 0.001095

At node N8:

split lnL Rel.Prob

[G|CE] -58.25 0.09855

[D|CE] -58.49 0.07732

[F|CE] -58.49 0.07732

[DG|E] -58.63 0.06778

[FG|C] -58.63 0.06778

[FG|E] -58.63 0.06778

[DG|C] -58.63 0.06778

[DF|C] -58.89 0.052

[DF|E] -58.89 0.052

[G|C] -59.09 0.04283

[G|E] -59.09 0.04283

[D|C] -59.33 0.0336

[D|E] -59.33 0.0336

[F|C] -59.33 0.0336

[F|E] -59.33 0.0336

[G|CEG] -60.95 0.006658

[D|CDE] -61.19 0.005224

[F|CEF] -61.19 0.005224

[G|CG] -61.46 0.003985

[G|EG] -61.46 0.003985

[D|CD] -61.7 0.003126

[D|DE] -61.7 0.003126

[F|CF] -61.7 0.003126

[F|EF] -61.7 0.003126

[G|EJ] -61.71 0.003089

[G|CJ] -61.71 0.003089

[C|CE] -61.83 0.002747

[E|CE] -61.83 0.002747

[CG|C] -61.84 0.002728

[EG|E] -61.84 0.002728

[DFG|D] -61.87 0.002655

[DFG|G] -61.87 0.002655

[DFG|F] -61.87 0.002655

[D|CJ] -61.96 0.002423

[D|EJ] -61.96 0.002423

[F|CJ] -61.96 0.002423

[F|EJ] -61.96 0.002423

[CFG|C] -61.98 0.00237

[EFG|E] -61.98 0.00237

[DEG|E] -61.98 0.00237

[CDG|C] -61.98 0.00237

[FG|G] -61.99 0.002358

[FG|F] -61.99 0.002358

[DG|D] -61.99 0.002358

[DG|G] -61.99 0.002358

[J|CE] -62.09 0.002129

[GJ|E] -62.09 0.002114

[GJ|C] -62.09 0.002114

[CD|C] -62.22 0.00186

[DE|E] -62.22 0.00186

[CF|C] -62.22 0.00186

At node N4:

split lnL Rel.Prob

[DF|G] -56.31 0.6884

[F|G] -58.03 0.1231

[D|G] -58.03 0.1231

[DFG|G] -60.1 0.01547

At node N2:

split lnL Rel.Prob

[F|D] -55.97 0.9669

At node N7:

split lnL Rel.Prob

[E|C] -56.13 0.825

[C|C] -59.56 0.02666

[E|E] -59.56 0.02666

[CE|C] -59.9 0.01902

[E|CE] -59.9 0.01902

[EG|C] -60.78 0.0079

[E|CG] -60.78 0.0079

[DE|C] -60.9 0.006961

[EF|C] -60.9 0.006961

[E|CF] -60.9 0.006961

At node N19:

split lnL Rel.Prob

[BH|L] -56.83 0.4067

[ABH|H] -58 0.1268

[BH|K] -58.66 0.06543

[BHL|L] -58.84 0.05463

[AB|E] -59.77 0.02159

[AB|C] -59.77 0.02159

[AB|G] -59.78 0.02149

[AB|F] -59.78 0.02139

[AB|D] -59.78 0.02139

[B|CL] -59.9 0.01894

[B|EL] -59.9 0.01894

[B|GL] -59.91 0.01876

[B|DL] -59.92 0.0186

[B|FL] -59.92 0.0186

[H|KL] -60.17 0.01446

[H|HKL] -60.21 0.01385

[BH|J] -60.35 0.01212

[BHK|K] -60.73 0.008309

[BH|E] -60.97 0.006496

[BH|C] -60.97 0.006496

[BH|G] -60.98 0.006454

[BH|F] -60.98 0.006414

[BH|D] -60.98 0.006414

[ABH|B] -61.21 0.005141

[B|BCL] -61.61 0.003443

[B|BEL] -61.61 0.003443

[B|BGL] -61.62 0.003411

At node N15:

split lnL Rel.Prob

[BH|A] -56.89 0.3862

[H|AB] -56.89 0.3859

[H|B] -58.38 0.08659

[B|A] -59.38 0.0319

[ABH|A] -59.43 0.0302

[H|ABH] -59.54 0.02708

[BH|B] -60.39 0.01158

At node N18:

split lnL Rel.Prob

[K|L] -56.58 0.5252

[L|L] -58.44 0.08143

[KL|L] -59.27 0.03556

[HK|L] -59.67 0.02382

[K|HL] -59.68 0.02355

[K|KL] -59.73 0.02252

[EK|L] -59.85 0.01995

[CK|L] -59.85 0.01995

[GK|L] -59.85 0.01989

[FK|L] -59.86 0.01983

[DK|L] -59.86 0.01983

[K|EL] -60.01 0.017

[K|CL] -60.01 0.017

[K|GL] -60.01 0.01695

[K|FL] -60.02 0.0169

[K|DL] -60.02 0.0169

[JK|L] -60.23 0.01361

[K|JL] -60.39 0.01159

[H|L] -61.16 0.00536

[E|L] -61.6 0.00348

[C|L] -61.6 0.00348

[G|L] -61.6 0.003472

[F|L] -61.6 0.003465

[D|L] -61.6 0.003465

[J|L] -61.89 0.002594

[HKL|L] -61.97 0.002403

[EKL|L] -61.99 0.002345

***RESULTS OF RUN FROM Margo_loc2.lagrange.py INPUT FILE SUBMITTED TO DRYAD***

Global ML at root node:

-lnL = 65.62

dispersal = 2.228

extinction = 0.3751

Ancestral range subdivision/inheritance scenarios ('splits') at

internal nodes.

* Split format: [left|right], where 'left' and 'right' are the ranges

inherited by each descendant branch (on the printed tree, 'left' is

the upper branch, and 'right' the lower branch).

* Only splits within 2 log-likelihood units of the maximum for each

node are shown. 'Rel.Prob' is the relative probability (fraction of

the global likelihood) of a split.

At node N22:

split lnL Rel.Prob

[I|I] -68.64 0.04876

[H|H] -68.83 0.04011

[B|B] -69.17 0.02859

[H|HI] -69.83 0.01473

[BH|H] -69.85 0.01448

[G|G] -69.9 0.01385

[H|I] -69.9 0.01383

[E|E] -69.9 0.01378

[C|C] -69.9 0.01378

[F|F] -69.95 0.01317

[D|D] -69.95 0.01317

[A|A] -70.02 0.01228

[L|L] -70.02 0.0122

[B|H] -70.04 0.01193

[B|BH] -70.17 0.01052

[HI|I] -70.23 0.009912

[K|K] -70.26 0.009612

[J|J] -70.29 0.00931

[I|HI] -70.37 0.008653

[GH|H] -70.63 0.006614

[EH|H] -70.64 0.006579

[CH|H] -70.64 0.006579

[HL|H] -70.66 0.006456

[FH|H] -70.69 0.006242

[DH|H] -70.69 0.006242

[H|BH] -70.75 0.005894

[H|HL] -70.75 0.005887

[H|HK] -70.75 0.005886

[H|HJ] -70.75 0.005886

[G|H] -70.75 0.005864

[C|H] -70.76 0.005837

[E|H] -70.76 0.005837

[H|FH] -70.76 0.005836

[H|EH] -70.76 0.005836

[H|GH] -70.76 0.005836

[H|CH] -70.76 0.005836

[H|DH] -70.76 0.005836

[BH|B] -70.77 0.005784

[F|H] -70.8 0.005578

[D|H] -70.8 0.005578

[G|GH] -70.89 0.00512

[E|EH] -70.9 0.005096

[C|CH] -70.9 0.005096

[L|H] -70.91 0.00501

[HK|H] -70.92 0.004979

[F|FH] -70.94 0.00487

[D|DH] -70.94 0.00487

[HJ|H] -70.95 0.004819

[HI|H] -70.96 0.004789

[AB|B] -70.96 0.004772

[BG|B] -70.96 0.00476

[BE|B] -70.97 0.004735

[BC|B] -70.97 0.004735

[B|F] -70.98 0.004696

[B|E] -70.98 0.004696

[B|G] -70.98 0.004696

[B|C] -70.98 0.004696

[B|D] -70.98 0.004696

[BG|G] -70.98 0.00469

[BE|E] -70.98 0.004665

[BC|C] -70.98 0.004665

[BF|B] -71.02 0.004497

[BD|B] -71.02 0.004497

[BF|F] -71.03 0.004432

[BD|D] -71.03 0.004432

[L|HL] -71.04 0.004412

[B|BF] -71.1 0.004151

[B|BG] -71.1 0.004151

[B|BE] -71.1 0.004151

[B|BC] -71.1 0.004151

[B|BD] -71.1 0.004151

[A|B] -71.11 0.00413

[K|H] -71.15 0.003949

[I|H] -71.16 0.003927

[J|H] -71.18 0.003825

[K|HK] -71.28 0.003477

[J|HJ] -71.31 0.003368

[B|AB] -71.44 0.002955

[H|L] -71.53 0.002713

[H|K] -71.53 0.002712

[H|J] -71.53 0.002712

[H|B] -71.54 0.00267

[H|F] -71.56 0.002631

[H|E] -71.56 0.002631

[H|G] -71.56 0.002631

[H|C] -71.56 0.002631

[H|D] -71.56 0.002631

[HL|L] -71.56 0.00262

[GH|G] -71.57 0.002603

[EH|E] -71.57 0.002589

[CH|C] -71.57 0.002589

[A|AB] -71.58 0.002561

[FH|F] -71.62 0.002457

[DH|D] -71.62 0.002457

[G|L] -71.66 0.00238

[G|K] -71.66 0.002379

[G|J] -71.66 0.002379

[C|L] -71.66 0.002369

[E|L] -71.66 0.002369

[C|K] -71.66 0.002368

[C|J] -71.66 0.002368

[E|K] -71.66 0.002368

[E|J] -71.66 0.002368

[AB|A] -71.66 0.002365

[B|A] -71.66 0.002362

[G|B] -71.67 0.002342

[C|B] -71.68 0.002332

[E|B] -71.68 0.002332

[G|F] -71.69 0.002308

[G|E] -71.69 0.002308

[G|C] -71.69 0.002308

[G|D] -71.69 0.002308

[C|F] -71.69 0.002297

[E|F] -71.69 0.002297

[C|E] -71.69 0.002297

[C|G] -71.69 0.002297

[E|G] -71.69 0.002297

[E|C] -71.69 0.002297

[C|D] -71.69 0.002297

[E|D] -71.69 0.002297

[F|L] -71.71 0.002264

[D|L] -71.71 0.002264

[F|K] -71.71 0.002263

[F|J] -71.71 0.002263

[D|K] -71.71 0.002263

[D|J] -71.71 0.002263

[GL|L] -71.71 0.00225

[CL|L] -71.72 0.002235

[EL|L] -71.72 0.002235

[F|B] -71.72 0.002228

[D|B] -71.72 0.002228

[F|E] -71.74 0.002195

[F|G] -71.74 0.002195

[F|C] -71.74 0.002195

[F|D] -71.74 0.002195

[D|F] -71.74 0.002195

[D|E] -71.74 0.002195

[D|G] -71.74 0.002195

[D|C] -71.74 0.002195

[GL|G] -71.74 0.002182

[CL|C] -71.75 0.002168

[EL|E] -71.75 0.002168

[EG|E] -71.76 0.002139

[EG|G] -71.76 0.002139

[CG|G] -71.76 0.002139

[CG|C] -71.76 0.002139

[CE|E] -71.77 0.00213

[CE|C] -71.77 0.00213

[FL|L] -71.78 0.002098

[DL|L] -71.78 0.002098

[FG|F] -71.8 0.002056

[DG|G] -71.8 0.002056

[FG|G] -71.8 0.002056

[DG|D] -71.8 0.002056

[CF|F] -71.81 0.002047

[EF|F] -71.81 0.002047

[CF|C] -71.81 0.002047

[EF|E] -71.81 0.002047

[CD|C] -71.81 0.002047

[DE|E] -71.81 0.002047

[CD|D] -71.81 0.002047

[DE|D] -71.81 0.002047

[G|BG] -71.81 0.00204

[FL|F] -71.81 0.002035

[DL|D] -71.81 0.002035

[G|GL] -71.81 0.002034

[G|GJ] -71.81 0.002033

[G|GK] -71.81 0.002033

[L|K] -71.81 0.002033

[L|J] -71.81 0.002033

[C|BC] -71.82 0.002031

[E|BE] -71.82 0.002031

[C|CL] -71.82 0.002024

[E|EL] -71.82 0.002024

[C|CJ] -71.82 0.002023

[E|EK] -71.82 0.002023

[C|CK] -71.82 0.002023

[E|EJ] -71.82 0.002023

[HK|K] -71.82 0.00202

[G|FG] -71.84 0.001989

[G|EG] -71.84 0.001989

[G|DG] -71.84 0.001989

[G|CG] -71.84 0.001989

[C|CF] -71.84 0.00198

[E|EF] -71.84 0.00198

[E|DE] -71.84 0.00198

[C|CE] -71.84 0.00198

[E|CE] -71.84 0.00198

[E|EG] -71.84 0.00198

[C|CD] -71.84 0.00198

[C|CG] -71.84 0.00198

[L|F] -71.84 0.001972

[L|E] -71.84 0.001972

[L|G] -71.84 0.001972

[L|C] -71.84 0.001972

[L|D] -71.84 0.001972

[DF|F] -71.85 0.001965

[DF|D] -71.85 0.001965

[HJ|J] -71.85 0.001955

[F|BF] -71.86 0.00194

[D|BD] -71.86 0.00194

[F|FL] -71.86 0.001934

[D|DL] -71.86 0.001934

[F|FJ] -71.86 0.001934

[F|FK] -71.86 0.001934

[D|DJ] -71.86 0.001934

[D|DK] -71.86 0.001934

[F|CF] -71.89 0.001892

[F|FG] -71.89 0.001892

[F|EF] -71.89 0.001892

[F|DF] -71.89 0.001892

[D|DE] -71.89 0.001892

[D|CD] -71.89 0.001892

[D|DF] -71.89 0.001892

[D|DG] -71.89 0.001892

[L|JL] -71.95 0.001775

[L|KL] -71.95 0.001775

[L|FL] -71.97 0.001737

[L|CL] -71.97 0.001737

[L|GL] -71.97 0.001737

[L|EL] -71.97 0.001737

[L|DL] -71.97 0.001737

[GK|K] -71.98 0.001728

[CK|K] -71.98 0.001718

[EK|K] -71.98 0.001718

[GK|G] -72.01 0.001676

[CK|C] -72.01 0.001667

[EK|E] -72.01 0.001667

[GJ|J] -72.01 0.001667

[CJ|J] -72.02 0.001658

[EJ|J] -72.02 0.001658

[FK|K] -72.04 0.001629

[DK|K] -72.04 0.001629

[GJ|G] -72.04 0.001617

[CJ|C] -72.05 0.001608

[EJ|E] -72.05 0.001608

[K|L] -72.05 0.001603

[K|J] -72.05 0.001602

[FK|F] -72.07 0.00158

[DK|D] -72.07 0.00158

[FJ|J] -72.07 0.001574

[DJ|J] -72.07 0.001574

At node N20:

split lnL Rel.Prob

[B|H] -68.04 0.08873

[B|BH] -68.12 0.08163

[BG|B] -68.89 0.03785

[BE|B] -68.92 0.03679

[BC|B] -68.92 0.03679

[BD|B] -69.23 0.02684

[BF|B] -69.23 0.02684

[B|B] -69.3 0.02519

[H|HL] -69.46 0.02141

[B|BE] -69.48 0.02093

[B|BD] -69.48 0.02093

[B|BG] -69.48 0.02093

[B|BC] -69.48 0.02093

[B|BF] -69.48 0.02093

[G|H] -69.58 0.0189

[E|H] -69.61 0.01841

[C|H] -69.61 0.01841

[BH|H] -69.66 0.01748

[G|L] -69.78 0.01554

[E|L] -69.81 0.01514

[C|L] -69.81 0.01514

[D|H] -69.9 0.01377

[F|H] -69.9 0.01377

[L|HL] -70.04 0.01194

[D|L] -70.1 0.01132

[F|L] -70.1 0.01132

[BG|G] -70.18 0.01038

[BE|E] -70.21 0.01009

[BC|C] -70.21 0.01009

[BD|D] -70.53 0.00736

[BF|F] -70.53 0.00736

[B|AB] -70.59 0.006892

[B|E] -70.6 0.006831

[B|G] -70.6 0.006831

[B|F] -70.6 0.006831

[B|C] -70.6 0.006831

[B|D] -70.6 0.006831

[BH|B] -70.7 0.006202

[G|B] -70.85 0.005305

[E|B] -70.88 0.005167

[C|B] -70.88 0.005167

[G|GH] -70.97 0.004722

[E|EH] -71 0.0046

[C|CH] -71 0.0046

[AB|B] -71.02 0.004512

[G|BG] -71.03 0.004457

[E|BE] -71.06 0.004341

[C|BC] -71.06 0.004341

[B|A] -71.17 0.003882

[D|B] -71.17 0.003863

[F|B] -71.17 0.003863

[GH|H] -71.24 0.003609

[H|H] -71.25 0.003558

[EH|H] -71.27 0.003514

[CH|H] -71.27 0.003514

[F|FH] -71.29 0.003439

[D|DH] -71.29 0.003439

[H|L] -71.32 0.003346

[G|GL] -71.32 0.003337

[E|EL] -71.34 0.00325

[C|CL] -71.34 0.00325

[D|BD] -71.35 0.003246

[F|BF] -71.35 0.003246

[H|BH] -71.44 0.002966

[GL|L] -71.48 0.00283

[EL|L] -71.51 0.002752

[CL|L] -71.51 0.002752

[H|HK] -71.55 0.002657

[FH|H] -71.56 0.002619

[DH|H] -71.56 0.002619

[G|K] -71.58 0.00257

[E|K] -71.61 0.002503

[C|K] -71.61 0.002503

[D|DL] -71.64 0.00243

[F|FL] -71.64 0.00243

[DL|L] -71.82 0.002022

[FL|L] -71.82 0.002022

[A|B] -71.82 0.00202

[D|K] -71.9 0.001871

[F|K] -71.9 0.001871

[J|H] -71.9 0.001859

[K|H] -71.92 0.001824

[L|H] -71.93 0.001809

[J|L] -72.1 0.001531

[L|L] -72.11 0.001514

[K|L] -72.12 0.001502

[K|HK] -72.12 0.001495

[G|G] -72.13 0.001489

[G|E] -72.15 0.001456

[G|F] -72.15 0.001456

[G|C] -72.15 0.001456

[G|D] -72.15 0.001456

[E|E] -72.15 0.00145

[C|C] -72.15 0.00145

[E|G] -72.17 0.001418

[E|F] -72.17 0.001418

[E|D] -72.17 0.001418

[E|C] -72.17 0.001418

[C|E] -72.17 0.001418

[C|G] -72.17 0.001418

[C|F] -72.17 0.001418

[C|D] -72.17 0.001418

[H|HI] -72.24 0.001324

[H|B] -72.39 0.001144

[D|D] -72.44 0.001084

[F|F] -72.44 0.001084

[D|E] -72.47 0.00106

[D|G] -72.47 0.00106

[D|F] -72.47 0.00106

[F|E] -72.47 0.00106

[F|G] -72.47 0.00106

[D|C] -72.47 0.00106

[F|D] -72.47 0.00106

[F|C] -72.47 0.00106

[G|J] -72.52 0.001001

[E|J] -72.55 0.0009751

[C|J] -72.55 0.0009751

At node N12:

split lnL Rel.Prob

[G|B] -67.52 0.1487

[E|B] -67.55 0.1441

[C|B] -67.55 0.1441

[D|B] -67.92 0.1002

[F|B] -67.92 0.1002

[G|BG] -68.65 0.04816

[E|BE] -68.68 0.04666

[C|BC] -68.68 0.04666

[BG|B] -68.96 0.03515

[BE|B] -69 0.03386

[BC|B] -69 0.03386

[F|BF] -69.04 0.03246

[D|BD] -69.04 0.03246

[BD|B] -69.44 0.02185

At node N10:

split lnL Rel.Prob

[G|J] -67.76 0.1172

[E|J] -67.81 0.1109

[C|J] -67.81 0.1109

[D|J] -68.38 0.06324

[F|J] -68.38 0.06324

[EG|G] -69.1 0.03065

[EG|E] -69.1 0.03065

[CG|G] -69.1 0.03065

[CG|C] -69.1 0.03065

[G|GJ] -69.25 0.02637

[E|EJ] -69.31 0.02495

[C|CJ] -69.31 0.02495

[DE|D] -69.72 0.0165

[DE|E] -69.72 0.0165

[EF|F] -69.72 0.0165

[EF|E] -69.72 0.0165

[CD|D] -69.72 0.0165

[CD|C] -69.72 0.0165

[CF|C] -69.72 0.0165

[CF|F] -69.72 0.0165

[D|DJ] -69.87 0.01423

[F|FJ] -69.87 0.01423

[G|G] -70.71 0.006159

[E|E] -70.76 0.005828

[C|C] -70.76 0.005828

[G|E] -70.77 0.005762

[G|D] -70.77 0.005762

[G|C] -70.77 0.005762

[G|F] -70.77 0.005762

[GJ|J] -70.79 0.005687

[E|G] -70.83 0.005452

[E|D] -70.83 0.005452

[E|C] -70.83 0.005452

[C|G] -70.83 0.005452

[E|F] -70.83 0.005452

[C|E] -70.83 0.005452

[C|D] -70.83 0.005452

[C|F] -70.83 0.005452

[EJ|J] -71.29 0.003439

[CJ|J] -71.29 0.003439

[G|K] -71.3 0.003415

[D|D] -71.32 0.003324

[F|F] -71.32 0.003324

[E|K] -71.35 0.003231

[C|K] -71.35 0.003231

[D|G] -71.39 0.003109

[D|E] -71.39 0.003109

[F|E] -71.39 0.003109

[F|G] -71.39 0.003109

At node N8:

split lnL Rel.Prob

[G|E] -67.24 0.1971

[G|C] -67.24 0.1971

[D|E] -67.87 0.1051

[F|E] -67.87 0.1051

[D|C] -67.87 0.1051

[F|C] -67.87 0.1051

[G|G] -69.49 0.02067

[D|D] -70.12 0.01102

[F|F] -70.12 0.01102

[E|E] -70.17 0.01048

[C|C] -70.17 0.01048

[G|EG] -70.39 0.008459

[G|CG] -70.39 0.008459

[E|CE] -70.62 0.006686

[C|CE] -70.62 0.006686

[DG|D] -70.74 0.005971

[DG|G] -70.74 0.005971

[FG|G] -70.74 0.005971

[FG|F] -70.74 0.005971

[G|J] -70.86 0.005297

[D|DE] -71.02 0.004508

[F|EF] -71.02 0.004508

[D|CD] -71.02 0.004508

At node N4:

split lnL Rel.Prob

[F|G] -66.4 0.4544

[D|G] -66.4 0.4544

[D|DG] -70.05 0.01183

[F|FG] -70.05 0.01183

[DF|F] -70.29 0.009302

[DF|D] -70.29 0.009302

At node N2:

split lnL Rel.Prob

[F|D] -65.64 0.9783

At node N7:

split lnL Rel.Prob

[E|C] -65.89 0.7601

[E|E] -69.02 0.03338

[C|C] -69.02 0.03338

[CE|C] -69.03 0.0328

[E|CE] -69.03 0.0328

[G|C] -69.62 0.01826

[E|G] -69.62 0.01826

[E|D] -70.15 0.01069

[E|F] -70.15 0.01069

At node N19:

split lnL Rel.Prob

[H|L] -66.73 0.3283

[BH|H] -67.36 0.1742

[H|HL] -68.51 0.05544

[B|G] -68.64 0.04858

[B|E] -68.64 0.04842

[B|C] -68.64 0.04842

[B|D] -68.68 0.04687

[B|F] -68.68 0.04687

[HL|L] -68.89 0.03794

[H|K] -69.16 0.02898

[AB|B] -70.13 0.01091

[H|HK] -70.44 0.008026

[B|BG] -70.51 0.00748

[B|BE] -70.51 0.007455

[B|BC] -70.51 0.007455

[B|BD] -70.55 0.007217

[B|BF] -70.55 0.007217

[BH|B] -70.7 0.006209

[BG|G] -70.8 0.005594

[BE|E] -70.81 0.005575

[BC|C] -70.81 0.005575

[BD|D] -70.84 0.005397

[BF|F] -70.84 0.005397

At node N15:

split lnL Rel.Prob

[H|B] -66.11 0.6098

[B|A] -67.57 0.1422

[BH|B] -67.71 0.1233

[B|B] -68.62 0.04956

[H|BH] -69.16 0.02898

At node N18:

split lnL Rel.Prob

[K|L] -66.49 0.4178

[L|L] -67.81 0.1116

[KL|L] -67.88 0.104

[H|L] -68.94 0.03615

[G|L] -68.97 0.03481

[E|L] -68.98 0.03473

[C|L] -68.98 0.03473

[D|L] -69 0.03395

[F|L] -69 0.03395

[K|KL] -69.17 0.02851

[J|L] -69.89 0.01391

[K|H] -70.54 0.007299

[K|G] -70.62 0.006711

[K|E] -70.62 0.006694

[K|C] -70.62 0.006694

[K|D] -70.65 0.006536

[K|F] -70.65 0.006536

[K|K] -70.69 0.006264

[L|HL] -71.47 0.002878

[H|HL] -71.62 0.002467

[G|GL] -71.66 0.002375

[E|EL] -71.66 0.00237

[C|CL] -71.66 0.00237

[D|DL] -71.68 0.002317

[F|FL] -71.68 0.002317

[K|J] -71.76 0.002152

***RESULTS OF RUN FROM Margo_loc3.lagrange.py INPUT FILE SUBMITTED TO DRYAD***

Global ML at root node:

-lnL = 50.01

dispersal = 0.3092

extinction = 0.6331

Ancestral range subdivision/inheritance scenarios ('splits') at

internal nodes.

* Split format: [left|right], where 'left' and 'right' are the ranges

inherited by each descendant branch (on the printed tree, 'left' is

the upper branch, and 'right' the lower branch).

* Only splits within 2 log-likelihood units of the maximum for each

node are shown. 'Rel.Prob' is the relative probability (fraction of

the global likelihood) of a split.

At node N22:

split lnL Rel.Prob

[H|H] -54.92 0.007405

[I|I] -55.47 0.004269

[B|B] -55.52 0.00404

[C|C] -55.53 0.004036

[E|E] -55.53 0.004036

[G|G] -55.53 0.004024

[F|F] -55.53 0.004011

[D|D] -55.53 0.004011

[J|J] -55.58 0.003821

[K|K] -55.58 0.003809

[L|L] -55.58 0.003809

[H|HI] -56.04 0.002417

[HI|I] -56.09 0.002284

[H|I] -56.16 0.002143

[BH|I] -56.38 0.001716

[BH|H] -56.42 0.001647

[BHI|I] -56.43 0.00164

[B|BHI] -56.46 0.001592

[B|HI] -56.47 0.001565

[CH|I] -56.49 0.001534

[EH|I] -56.49 0.001534

[GH|I] -56.5 0.00153

[FH|I] -56.5 0.001526

[DH|I] -56.5 0.001526

[HJ|I] -56.51 0.001513

[HK|I] -56.51 0.001512

[HL|I] -56.51 0.001512

[H|BHI] -56.53 0.001475

[H|HIL] -56.53 0.001472

[H|HIK] -56.53 0.001472

[H|HIJ] -56.53 0.001472

[CH|H] -56.53 0.001472

[EH|H] -56.53 0.001472

[CHI|I] -56.54 0.00147

[EHI|I] -56.54 0.00147

[GH|H] -56.54 0.001469

[GHI|I] -56.54 0.001467

[FH|H] -56.54 0.001465

[DH|H] -56.54 0.001465

[FHI|I] -56.54 0.001463

[DHI|I] -56.54 0.001463

[H|DHI] -56.54 0.001457

[H|EHI] -56.54 0.001457

[H|GHI] -56.54 0.001457

[H|FHI] -56.54 0.001457

[H|CHI] -56.54 0.001457

[HIJ|I] -56.54 0.001457

[HIK|I] -56.54 0.001457

[HIL|I] -56.54 0.001457

[E|EHI] -56.55 0.001454

[C|CHI] -56.55 0.001454

[HJ|H] -56.55 0.001452

[HK|H] -56.55 0.001451

[HL|H] -56.55 0.001451

[G|GHI] -56.55 0.001449

[C|HI] -56.55 0.001447

[E|HI] -56.55 0.001447

[B|BH] -56.55 0.001446

[D|DHI] -56.55 0.001445

[F|FHI] -56.55 0.001445

[G|HI] -56.55 0.001442

[F|HI] -56.56 0.001438

[D|HI] -56.56 0.001438

[J|HIJ] -56.6 0.001383

[L|HIL] -56.6 0.001379

[K|HIK] -56.6 0.001379

[J|HI] -56.61 0.001362

[K|HI] -56.61 0.001358

[L|HI] -56.61 0.001358

[H|BH] -56.63 0.00134

[B|H] -56.63 0.001332

[H|HK] -56.64 0.001325

[H|HL] -56.64 0.001325

[H|HJ] -56.64 0.001325

[HI|H] -56.65 0.001315

[H|GH] -56.67 0.001282

[H|DH] -56.67 0.001282

[H|FH] -56.67 0.001282

[H|EH] -56.67 0.001282

[H|CH] -56.67 0.001282

[C|CH] -56.67 0.001279

[E|EH] -56.67 0.001279

[G|GH] -56.68 0.001275

[F|FH] -56.68 0.001271

[D|DH] -56.68 0.001271

[J|HJ] -56.7 0.001245

[K|HK] -56.71 0.001241

[L|HL] -56.71 0.001241

[C|H] -56.71 0.001231

[E|H] -56.71 0.001231

[G|H] -56.72 0.001227

[F|H] -56.72 0.001223

[D|H] -56.72 0.001223

[J|H] -56.77 0.001159

[K|H] -56.78 0.001156

[L|H] -56.78 0.001156

[ABH|H] -56.78 0.001148

[AB|H] -56.89 0.001029

[BHK|H] -56.9 0.001018

[BHL|H] -56.9 0.001018

[BHJ|H] -56.91 0.001014

[B|BHL] -56.95 0.0009706

[B|BHK] -56.95 0.0009706

[B|BHJ] -56.95 0.0009706

[BHI|H] -56.98 0.0009442

[H|BHL] -57.03 0.0008995

[H|BHK] -57.03 0.0008995

[H|BHJ] -57.03 0.0008995

[BC|C] -57.03 0.0008977

[BE|E] -57.03 0.0008977

[BG|G] -57.03 0.0008942

[BD|D] -57.04 0.0008903

[BF|F] -57.04 0.0008903

[AB|B] -57.06 0.0008669

[B|HL] -57.07 0.0008578

[B|HK] -57.07 0.0008578

[B|HJ] -57.07 0.0008578

[CHI|H] -57.09 0.0008468

[EHI|H] -57.09 0.0008468

[J|BHJ] -57.09 0.0008449

[GHI|H] -57.09 0.0008447

[FHI|H] -57.09 0.0008424

[DHI|H] -57.09 0.0008424

[K|BHK] -57.09 0.0008423

[L|BHL] -57.09 0.0008423

[HIJ|H] -57.1 0.0008391

[BCH|H] -57.1 0.0008389

[BEH|H] -57.1 0.0008389

[HIK|H] -57.1 0.0008389

[HIL|H] -57.1 0.0008389

[BGH|H] -57.1 0.0008364

[BFH|H] -57.1 0.0008335

[BDH|H] -57.1 0.0008335

[BH|B] -57.1 0.0008329

[BC|B] -57.11 0.0008306

[BE|B] -57.11 0.0008306

[BG|B] -57.11 0.0008274

[BD|B] -57.11 0.0008238

[BF|B] -57.11 0.0008238

[BC|H] -57.12 0.0008215

[BE|H] -57.12 0.0008215

[BG|H] -57.12 0.0008183

[BD|H] -57.13 0.0008147

[BF|H] -57.13 0.0008147

[CE|E] -57.13 0.0008087

[CE|C] -57.13 0.0008087

[CG|G] -57.14 0.0008068

[EG|G] -57.14 0.0008068

[EG|E] -57.14 0.0008068

[CG|C] -57.14 0.0008068

[CD|D] -57.14 0.0008047

[EF|E] -57.14 0.0008047

[CD|C] -57.14 0.0008047

[DE|E] -57.14 0.0008047

[CF|C] -57.14 0.0008047

[DE|D] -57.14 0.0008047

[CF|F] -57.14 0.0008047

[EF|F] -57.14 0.0008047

[EH|E] -57.14 0.0008043

[CH|C] -57.14 0.0008043

[FG|G] -57.14 0.0008029

[DG|G] -57.14 0.0008029

[FG|F] -57.14 0.0008029

[DG|D] -57.14 0.0008029

[I|HI] -57.14 0.0008025

[GH|G] -57.14 0.0008024

[DF|D] -57.14 0.0008008

[DF|F] -57.14 0.0008008

[EJ|J] -57.14 0.0008005

[CJ|J] -57.14 0.0008005

[DH|D] -57.14 0.0008003

[FH|F] -57.14 0.0008003

[B|BG] -57.15 0.0007984

[B|BD] -57.15 0.0007984

[B|BE] -57.15 0.0007984

[B|BF] -57.15 0.0007984

[B|BC] -57.15 0.0007984

[GJ|J] -57.15 0.0007981

[CK|K] -57.15 0.0007979

[CL|L] -57.15 0.0007979

[EK|K] -57.15 0.0007979

[EL|L] -57.15 0.0007979

[HJ|J] -57.15 0.0007976

[HK|K] -57.15 0.0007969

[HL|L] -57.15 0.0007969

[EJ|E] -57.15 0.0007962

[CJ|C] -57.15 0.0007962

[GK|K] -57.15 0.0007956

[GL|L] -57.15 0.0007956

[FJ|J] -57.15 0.0007953

[DJ|J] -57.15 0.0007953

[GJ|G] -57.15 0.0007938

[CL|C] -57.15 0.0007937

[EL|E] -57.15 0.0007937

[EK|E] -57.15 0.0007937

[CK|C] -57.15 0.0007937

[FK|K] -57.15 0.000793

[DK|K] -57.15 0.000793

[FL|L] -57.15 0.000793

[DL|L] -57.15 0.000793

[GK|G] -57.15 0.0007914

[GL|G] -57.15 0.0007914

[FJ|F] -57.16 0.0007911

[DJ|D] -57.16 0.0007911

[FK|F] -57.16 0.0007888

[DK|D] -57.16 0.0007888

[FL|F] -57.16 0.0007888

[DL|D] -57.16 0.0007888

[C|CL] -57.17 0.0007772

[E|EK] -57.17 0.0007772

[C|CK] -57.17 0.0007772

[C|CJ] -57.17 0.0007772

[E|EL] -57.17 0.0007772

[E|EJ] -57.17 0.0007772

[G|GL] -57.18 0.0007749

[G|GK] -57.18 0.0007749

[G|GJ] -57.18 0.0007749

[C|CG] -57.18 0.0007741

[C|CE] -57.18 0.0007741

[E|EG] -57.18 0.0007741

[E|CE] -57.18 0.0007741

[E|DE] -57.18 0.0007741

[C|CF] -57.18 0.0007741

[C|CD] -57.18 0.0007741

[E|EF] -57.18 0.0007741

[F|FK] -57.18 0.0007722

[F|FL] -57.18 0.0007722

[D|DK] -57.18 0.0007722

[D|DL] -57.18 0.0007722

[D|DJ] -57.18 0.0007722

[F|FJ] -57.18 0.0007722

[G|DG] -57.18 0.0007718

[G|EG] -57.18 0.0007718

[G|CG] -57.18 0.0007718

[G|FG] -57.18 0.0007718

[F|FG] -57.18 0.0007692

[D|DG] -57.18 0.0007692

[F|DF] -57.18 0.0007692

[F|EF] -57.18 0.0007692

[D|DE] -57.18 0.0007692

[F|CF] -57.18 0.0007692

[D|DF] -57.18 0.0007692

[D|CD] -57.18 0.0007692

[JK|K] -57.19 0.0007644

[JL|L] -57.19 0.0007644

[JK|J] -57.19 0.0007644

[JL|J] -57.19 0.0007644

[KL|K] -57.19 0.0007624

[KL|L] -57.19 0.0007624

[CEH|H] -57.2 0.0007572

[CGH|H] -57.2 0.0007555

[EGH|H] -57.2 0.0007555

[J|BH] -57.2 0.000755

[CDH|H] -57.2 0.0007536

[EFH|H] -57.2 0.0007536

[CFH|H] -57.2 0.0007536

[DEH|H] -57.2 0.0007536

[K|BH] -57.2 0.0007527

[L|BH] -57.2 0.0007527

[DGH|H] -57.21 0.0007518

[FGH|H] -57.21 0.0007518

[DFH|H] -57.21 0.0007499

[CHJ|H] -57.21 0.0007488

[EHJ|H] -57.21 0.0007488

[CHL|H] -57.21 0.0007474

[EHK|H] -57.21 0.0007474

[CHK|H] -57.21 0.0007474

[EHL|H] -57.21 0.0007474

[GHJ|H] -57.21 0.0007468

[J|JK] -57.21 0.0007461

[J|JL] -57.21 0.0007461

[GHL|H] -57.21 0.0007455

[GHK|H] -57.21 0.0007455

[FHJ|H] -57.22 0.0007446

[DHJ|H] -57.22 0.0007446

[K|KL] -57.22 0.0007438

[L|KL] -57.22 0.0007438

[K|JK] -57.22 0.0007438

[L|JL] -57.22 0.0007438

[FHK|H] -57.22 0.0007433

[DHK|H] -57.22 0.0007433

[FHL|H] -57.22 0.0007433

[DHL|H] -57.22 0.0007433

[CE|H] -57.22 0.00074

[CG|H] -57.22 0.0007383

[EG|H] -57.22 0.0007383

[C|BC] -57.22 0.000738

[E|BE] -57.22 0.000738

[CD|H] -57.23 0.0007364

[CF|H] -57.23 0.0007364

[EF|H] -57.23 0.0007364

[DE|H] -57.23 0.0007364

[G|BG] -57.23 0.0007358

[FG|H] -57.23 0.0007347

[DG|H] -57.23 0.0007347

[F|BF] -57.23 0.0007333

[D|BD] -57.23 0.0007333

[DF|H] -57.23 0.0007328

[J|GJ] -57.23 0.0007319

[J|EJ] -57.23 0.0007319

[J|DJ] -57.23 0.0007319

[J|CJ] -57.23 0.0007319

[J|FJ] -57.23 0.0007319

[HJK|H] -57.23 0.0007306

[HJL|H] -57.23 0.0007306

[L|GL] -57.24 0.0007296

[K|GK] -57.24 0.0007296

[K|FK] -57.24 0.0007296

[K|EK] -57.24 0.0007296

[K|DK] -57.24 0.0007296

[L|EL] -57.24 0.0007296

[L|FL] -57.24 0.0007296

[L|DL] -57.24 0.0007296

[K|CK] -57.24 0.0007296

[L|CL] -57.24 0.0007296

[HKL|H] -57.24 0.0007295

[EJ|H] -57.24 0.0007286

[CJ|H] -57.24 0.0007286

[B|G] -57.24 0.0007277

[B|E] -57.24 0.0007277

[B|D] -57.24 0.0007277

[B|C] -57.24 0.0007277

[B|F] -57.24 0.0007277

[GJ|H] -57.24 0.0007264

[CL|H] -57.24 0.0007263

[CK|H] -57.24 0.0007263

[EL|H] -57.24 0.0007263

[EK|H] -57.24 0.0007263

[GL|H] -57.24 0.0007242

[GK|H] -57.24 0.0007242

[FJ|H] -57.24 0.0007239

[DJ|H] -57.24 0.0007239

[FK|H] -57.25 0.0007218

[FL|H] -57.25 0.0007218

[DK|H] -57.25 0.0007218

[DL|H] -57.25 0.0007218

[JK|H] -57.28 0.0006958

[JL|H] -57.28 0.0006958

[KL|H] -57.29 0.000694

[B|GH] -57.29 0.0006918

[B|DH] -57.29 0.0006918

[B|FH] -57.29 0.0006918

[B|EH] -57.29 0.0006918

[B|CH] -57.29 0.0006918

[H|K] -57.31 0.0006779

[H|L] -57.31 0.0006779

[H|J] -57.31 0.0006779

[C|K] -57.31 0.0006763

[C|L] -57.31 0.0006763

[E|K] -57.31 0.0006763

[C|J] -57.31 0.0006763

[E|L] -57.31 0.0006763

[E|J] -57.31 0.0006763

[H|G] -57.31 0.0006743

[H|E] -57.31 0.0006743

[H|D] -57.31 0.0006743

[H|C] -57.31 0.0006743

[H|F] -57.31 0.0006743

[G|K] -57.31 0.0006742

[G|L] -57.31 0.0006742

[G|J] -57.31 0.0006742

[B|ABH] -57.32 0.000673

[C|G] -57.32 0.0006727

[C|E] -57.32 0.0006727

[C|D] -57.32 0.0006727

[E|G] -57.32 0.0006727

[E|D] -57.32 0.0006727

[C|F] -57.32 0.0006727

[E|C] -57.32 0.0006727

[E|F] -57.32 0.0006727

[F|K] -57.32 0.000672

[F|L] -57.32 0.000672

[D|K] -57.32 0.000672

[F|J] -57.32 0.000672

[D|L] -57.32 0.000672

[D|J] -57.32 0.000672

[G|E] -57.32 0.0006707

[G|D] -57.32 0.0006707

[G|C] -57.32 0.0006707

[G|F] -57.32 0.0006707

[B|AB] -57.32 0.0006685

[F|G] -57.32 0.0006684

[F|E] -57.32 0.0006684

[F|D] -57.32 0.0006684

[D|G] -57.32 0.0006684

[F|C] -57.32 0.0006684

[D|E] -57.32 0.0006684

[D|C] -57.32 0.0006684

[D|F] -57.32 0.0006684

[C|BH] -57.32 0.0006682

[E|BH] -57.32 0.0006682

[G|BH] -57.33 0.0006662

[F|BH] -57.33 0.0006639

[D|BH] -57.33 0.0006639

[C|HK] -57.34 0.0006608

[C|HL] -57.34 0.0006608

[E|HK] -57.34 0.0006608

[E|HL] -57.34 0.0006608

[C|HJ] -57.34 0.0006608

[E|HJ] -57.34 0.0006608

[G|HK] -57.34 0.0006588

[G|HL] -57.34 0.0006588

[G|HJ] -57.34 0.0006588

[F|HL] -57.34 0.0006566

[F|HK] -57.34 0.0006566

[F|HJ] -57.34 0.0006566

[D|HL] -57.34 0.0006566

[D|HK] -57.34 0.0006566

[D|HJ] -57.34 0.0006566

[B|BDH] -57.37 0.0006395

[B|BCH] -57.37 0.0006395

[B|BFH] -57.37 0.0006395

[B|BEH] -57.37 0.0006395

[B|BGH] -57.37 0.0006395

[C|GH] -57.37 0.0006395

[C|DH] -57.37 0.0006395

[E|GH] -57.37 0.0006395

[E|DH] -57.37 0.0006395

[C|FH] -57.37 0.0006395

[C|EH] -57.37 0.0006395

[E|FH] -57.37 0.0006395

[E|CH] -57.37 0.0006395

[G|DH] -57.37 0.0006376

[G|FH] -57.37 0.0006376

[G|EH] -57.37 0.0006376

[G|CH] -57.37 0.0006376

[J|K] -57.37 0.0006368

[J|L] -57.37 0.0006368

[F|GH] -57.37 0.0006354

[F|DH] -57.37 0.0006354

[F|EH] -57.37 0.0006354

[D|GH] -57.37 0.0006354

[F|CH] -57.37 0.0006354

[D|FH] -57.37 0.0006354

[D|EH] -57.37 0.0006354

[D|CH] -57.37 0.0006354

[L|K] -57.38 0.0006349

[K|L] -57.38 0.0006349

[K|J] -57.38 0.0006349

[L|J] -57.38 0.0006349

[J|G] -57.38 0.0006335

[J|E] -57.38 0.0006335

[J|D] -57.38 0.0006335

[J|C] -57.38 0.0006335

[J|F] -57.38 0.0006335

[K|G] -57.38 0.0006315

[K|E] -57.38 0.0006315

[L|G] -57.38 0.0006315

[L|E] -57.38 0.0006315

[K|D] -57.38 0.0006315

[L|D] -57.38 0.0006315

[K|C] -57.38 0.0006315

[L|C] -57.38 0.0006315

[K|F] -57.38 0.0006315

[L|F] -57.38 0.0006315

[H|B] -57.39 0.000624

[H|ABH] -57.39 0.0006237

[C|B] -57.39 0.0006224

[E|B] -57.39 0.0006224

[J|HL] -57.4 0.0006223

[J|HK] -57.4 0.0006223

[ABC|C] -57.4 0.0006215

[ABE|E] -57.4 0.0006215

[G|B] -57.4 0.0006206

[K|HL] -57.4 0.0006204

[L|HK] -57.4 0.0006204

[K|HJ] -57.4 0.0006204

[L|HJ] -57.4 0.0006204

[ABG|G] -57.4 0.0006192

[F|B] -57.4 0.0006185

[D|B] -57.4 0.0006185

[ABD|D] -57.4 0.0006166

[ABF|F] -57.4 0.0006166

[H|HKL] -57.42 0.0006087

[H|HJK] -57.42 0.0006087

[H|HJL] -57.42 0.0006087

[J|GH] -57.43 0.0006022

[J|DH] -57.43 0.0006022

[J|FH] -57.43 0.0006022

[J|EH] -57.43 0.0006022

[J|CH] -57.43 0.0006022

[K|GH] -57.43 0.0006003

[K|DH] -57.43 0.0006003

[L|GH] -57.43 0.0006003

[L|DH] -57.43 0.0006003

[K|FH] -57.43 0.0006003

[K|EH] -57.43 0.0006003

[L|FH] -57.43 0.0006003

[L|EH] -57.43 0.0006003

[K|CH] -57.43 0.0006003

[L|CH] -57.43 0.0006003

[H|EHJ] -57.44 0.0005977

[H|CHL] -57.44 0.0005977

[H|GHL] -57.44 0.0005977

[H|GHK] -57.44 0.0005977

[H|FHK] -57.44 0.0005977

[H|DHK] -57.44 0.0005977

[H|FHL] -57.44 0.0005977

[H|DHJ] -57.44 0.0005977

[H|DHL] -57.44 0.0005977

[H|EHK] -57.44 0.0005977

[H|CHK] -57.44 0.0005977

[H|EHL] -57.44 0.0005977

[H|FHJ] -57.44 0.0005977

[H|CHJ] -57.44 0.0005977

[H|GHJ] -57.44 0.0005977

[C|CHL] -57.44 0.0005962

[E|EHJ] -57.44 0.0005962

[C|CHK] -57.44 0.0005962

[C|CHJ] -57.44 0.0005962

[E|EHK] -57.44 0.0005962

[E|EHL] -57.44 0.0005962

[G|GHL] -57.44 0.0005944

[G|GHK] -57.44 0.0005944

[G|GHJ] -57.44 0.0005944

[H|BDH] -57.44 0.0005926

[H|BCH] -57.44 0.0005926

[H|BFH] -57.44 0.0005926

[H|BEH] -57.44 0.0005926

[H|BGH] -57.44 0.0005926

[F|FHK] -57.44 0.0005924

[F|FHL] -57.44 0.0005924

[D|DHK] -57.44 0.0005924

[F|FHJ] -57.44 0.0005924

[D|DHJ] -57.44 0.0005924

[D|DHL] -57.44 0.0005924

[C|BCH] -57.45 0.0005911

[E|BEH] -57.45 0.0005911

[G|BGH] -57.45 0.0005894

[H|DGH] -57.45 0.0005892

[H|EGH] -57.45 0.0005892

[H|CGH] -57.45 0.0005892

[H|CEH] -57.45 0.0005892

[H|FGH] -57.45 0.0005892

[H|DFH] -57.45 0.0005892

[H|CFH] -57.45 0.0005892

[H|DEH] -57.45 0.0005892

[H|EFH] -57.45 0.0005892

[H|CDH] -57.45 0.0005892

[C|CGH] -57.45 0.0005877

[C|CEH] -57.45 0.0005877

[E|EGH] -57.45 0.0005877

[E|CEH] -57.45 0.0005877

[C|CFH] -57.45 0.0005877

[E|DEH] -57.45 0.0005877

[C|CDH] -57.45 0.0005877

[E|EFH] -57.45 0.0005877

[D|BDH] -57.45 0.0005874

[F|BFH] -57.45 0.0005874

[G|DGH] -57.46 0.000586

[G|EGH] -57.46 0.000586

[G|CGH] -57.46 0.000586

[G|FGH] -57.46 0.000586

[D|DGH] -57.46 0.000584

[F|FGH] -57.46 0.000584

[F|DFH] -57.46 0.000584

[F|CFH] -57.46 0.000584

[F|EFH] -57.46 0.000584

[D|DFH] -57.46 0.000584

[D|DEH] -57.46 0.000584

[D|CDH] -57.46 0.000584

[ABH|B] -57.46 0.0005806

[A|A] -57.47 0.00058

[ABC|B] -57.47 0.000575

[ABE|B] -57.47 0.000575

[ABG|B] -57.48 0.0005729

[J|HJK] -57.48 0.0005718

[J|HJL] -57.48 0.0005718

[ABD|B] -57.48 0.0005706

[ABF|B] -57.48 0.0005706

[K|HKL] -57.48 0.0005701

[L|HKL] -57.48 0.0005701

[K|HJK] -57.48 0.0005701

[L|HJL] -57.48 0.0005701

[AB|G] -57.5 0.0005621

[AB|E] -57.5 0.0005621

[AB|D] -57.5 0.0005621

[AB|C] -57.5 0.0005621

[AB|F] -57.5 0.0005621

[J|EHJ] -57.5 0.0005614

[J|DHJ] -57.5 0.0005614

[J|FHJ] -57.5 0.0005614

[J|CHJ] -57.5 0.0005614

[J|GHJ] -57.5 0.0005614

[L|CHL] -57.5 0.0005597

[L|GHL] -57.5 0.0005597

[K|GHK] -57.5 0.0005597

[K|FHK] -57.5 0.0005597

[K|DHK] -57.5 0.0005597

[L|FHL] -57.5 0.0005597

[K|EHK] -57.5 0.0005597

[K|CHK] -57.5 0.0005597

[L|DHL] -57.5 0.0005597

[L|EHL] -57.5 0.0005597

[BHK|K] -57.5 0.0005593

[BHL|L] -57.5 0.0005593

[BHJ|J] -57.51 0.0005571

[BCJ|J] -57.51 0.0005523

[BEJ|J] -57.51 0.0005523

[BEJ|E] -57.52 0.0005494

[BCJ|C] -57.52 0.0005494

[BCK|K] -57.52 0.0005493

[BCL|L] -57.52 0.0005493

[BEK|K] -57.52 0.0005493

[BEL|L] -57.52 0.0005493

[BGJ|J] -57.52 0.0005485

[BCL|C] -57.53 0.0005464

[BEL|E] -57.53 0.0005464

[BCK|C] -57.53 0.0005464

[BEK|E] -57.53 0.0005464

[BGK|K] -57.53 0.0005458

[BGL|L] -57.53 0.0005458

[BGJ|G] -57.53 0.0005456

[BDJ|J] -57.53 0.0005443

[BFJ|J] -57.53 0.0005443

[BH|K] -57.53 0.000543

[BH|L] -57.53 0.000543

[BH|J] -57.53 0.000543

[BGL|G] -57.53 0.0005429

[BGK|G] -57.53 0.0005429

[BFK|K] -57.53 0.0005418

[BDK|K] -57.53 0.0005418

[BDL|L] -57.53 0.0005418

[BFL|L] -57.53 0.0005418

[BC|K] -57.53 0.0005415

[BC|L] -57.53 0.0005415

[BC|J] -57.53 0.0005415

[BE|K] -57.53 0.0005415

[BE|L] -57.53 0.0005415

[BE|J] -57.53 0.0005415

[BDJ|D] -57.53 0.0005414

[BFJ|F] -57.53 0.0005414

[BG|K] -57.54 0.0005394

[BG|L] -57.54 0.0005394

[BG|J] -57.54 0.0005394

[BDK|D] -57.54 0.0005389

[BDL|D] -57.54 0.0005389

[BFK|F] -57.54 0.0005389

[BFL|F] -57.54 0.0005389

[BD|K] -57.54 0.000537

[BF|K] -57.54 0.000537

[BD|L] -57.54 0.000537

[BF|L] -57.54 0.000537

[BD|J] -57.54 0.000537

[BF|J] -57.54 0.000537

[BHK|B] -57.58 0.0005148

[BHL|B] -57.58 0.0005148

[BHJ|B] -57.59 0.0005127

[BCJ|B] -57.6 0.0005083

[BEJ|B] -57.6 0.0005083

[BCL|B] -57.6 0.0005055

[BCK|B] -57.6 0.0005055

[BEL|B] -57.6 0.0005055

[BEK|B] -57.6 0.0005055

[BGJ|B] -57.6 0.0005049

[B|GL] -57.61 0.0005044

[B|GK] -57.61 0.0005044

[B|FK] -57.61 0.0005044

[B|GJ] -57.61 0.0005044

[B|EK] -57.61 0.0005044

[B|DK] -57.61 0.0005044

[B|EL] -57.61 0.0005044

[B|FL] -57.61 0.0005044

[B|EJ] -57.61 0.0005044

[B|DL] -57.61 0.0005044

[B|DJ] -57.61 0.0005044

[B|CL] -57.61 0.0005044

[B|CK] -57.61 0.0005044

[B|CJ] -57.61 0.0005044

[B|FJ] -57.61 0.0005044

[BGL|B] -57.61 0.0005023

[BGK|B] -57.61 0.0005023

[BDJ|B] -57.61 0.000501

[BFJ|B] -57.61 0.000501

[BFK|B] -57.62 0.0004987

[BDK|B] -57.62 0.0004987

[BDL|B] -57.62 0.0004987

[BFL|B] -57.62 0.0004987

[I|BHI] -57.63 0.0004898

[I|HIL] -57.64 0.0004888

[I|HIK] -57.64 0.0004888

[I|HIJ] -57.64 0.0004888

[I|DHI] -57.65 0.0004839

[I|EHI] -57.65 0.0004839

[I|GHI] -57.65 0.0004839

[I|FHI] -57.65 0.0004839

[I|CHI] -57.65 0.0004839

[BHI|B] -57.66 0.0004774

[B|BDL] -57.69 0.0004649

[B|BGK] -57.69 0.0004649

[B|BGL] -57.69 0.0004649

[B|BEL] -57.69 0.0004649

[B|BGJ] -57.69 0.0004649

[B|BFK] -57.69 0.0004649

[B|BDK] -57.69 0.0004649

[B|BCK] -57.69 0.0004649

[B|BEK] -57.69 0.0004649

[B|BDJ] -57.69 0.0004649

[B|BCL] -57.69 0.0004649

[B|BFL] -57.69 0.0004649

[B|BCJ] -57.69 0.0004649

[B|BFJ] -57.69 0.0004649

[B|BEJ] -57.69 0.0004649

[CHI|C] -57.69 0.0004627

[EHI|E] -57.69 0.0004627

[GHI|G] -57.69 0.0004616

[HIJ|J] -57.7 0.0004609

[HIK|K] -57.7 0.0004608

[HIL|L] -57.7 0.0004608

[DHI|D] -57.7 0.0004603

[FHI|F] -57.7 0.0004603

[BEH|E] -57.7 0.0004584

[BCH|C] -57.7 0.0004584

[BCE|E] -57.7 0.0004575

[BCE|C] -57.7 0.0004575

[BGH|G] -57.7 0.000457

[BCG|G] -57.71 0.0004562

[BCG|C] -57.71 0.0004562

[BEG|G] -57.71 0.0004562

[BEG|E] -57.71 0.0004562

[BFH|F] -57.71 0.0004554

[BDH|D] -57.71 0.0004554

[BCF|C] -57.71 0.0004546

[BCD|D] -57.71 0.0004546

[BCF|F] -57.71 0.0004546

[BCD|C] -57.71 0.0004546

[BDE|E] -57.71 0.0004546

[BDE|D] -57.71 0.0004546

[BEF|E] -57.71 0.0004546

[BEF|F] -57.71 0.0004546

[BFG|G] -57.71 0.0004533

[BDG|G] -57.71 0.0004533

[BDG|D] -57.71 0.0004533

[BFG|F] -57.71 0.0004533

[BDF|D] -57.72 0.0004517

[BDF|F] -57.72 0.0004517

[BH|G] -57.72 0.0004501

[BH|E] -57.72 0.0004501

[BH|D] -57.72 0.0004501

[BH|C] -57.72 0.0004501

[BH|F] -57.72 0.0004501

[BC|G] -57.72 0.0004488

[BC|E] -57.72 0.0004488

[BC|D] -57.72 0.0004488

[BC|F] -57.72 0.0004488

[BE|G] -57.72 0.0004488

[BE|D] -57.72 0.0004488

[BE|C] -57.72 0.0004488

[BE|F] -57.72 0.0004488

[BG|E] -57.73 0.0004471

[BG|D] -57.73 0.0004471

[BG|C] -57.73 0.0004471

[BG|F] -57.73 0.0004471

[BD|G] -57.73 0.0004452

[BD|E] -57.73 0.0004452

[BF|G] -57.73 0.0004452

[BF|E] -57.73 0.0004452

[BF|D] -57.73 0.0004452

[BD|C] -57.73 0.0004452

[BD|F] -57.73 0.0004452

[BF|C] -57.73 0.0004452

[EJ|B] -57.74 0.000442

[CJ|B] -57.74 0.000442

[GJ|B] -57.74 0.0004407

[CL|B] -57.74 0.0004406

[CK|B] -57.74 0.0004406

[EL|B] -57.74 0.0004406

[EK|B] -57.74 0.0004406

[HJ|B] -57.74 0.0004404

[HK|B] -57.74 0.0004401

[HL|B] -57.74 0.0004401

[GK|B] -57.74 0.0004394

[GL|B] -57.74 0.0004394

[FJ|B] -57.74 0.0004392

[DJ|B] -57.74 0.0004392

[FK|B] -57.75 0.0004379

[FL|B] -57.75 0.0004379

[DK|B] -57.75 0.0004379

[DL|B] -57.75 0.0004379

[HI|K] -57.76 0.0004335

[HI|L] -57.76 0.0004335

[HI|J] -57.76 0.0004335

[HI|G] -57.76 0.0004312

[HI|E] -57.76 0.0004312

[HI|D] -57.76 0.0004312

[HI|C] -57.76 0.0004312

[HI|F] -57.76 0.0004312

[C|BCK] -57.77 0.0004298

[E|BEL] -57.77 0.0004298

[C|BCL] -57.77 0.0004298

[E|BEK] -57.77 0.0004298

[C|BCJ] -57.77 0.0004298

[E|BEJ] -57.77 0.0004298

[A|BH] -57.77 0.0004291

[G|BGK] -57.77 0.0004285

[G|BGL] -57.77 0.0004285

[G|BGJ] -57.77 0.0004285

[D|BDL] -57.77 0.000427

[F|BFK] -57.77 0.000427

[D|BDK] -57.77 0.000427

[F|BFL] -57.77 0.000427

[D|BDJ] -57.77 0.000427

[F|BFJ] -57.77 0.000427

[BCH|B] -57.78 0.0004241

[BEH|B] -57.78 0.0004241

[BCE|B] -57.78 0.0004233

[BGH|B] -57.78 0.0004229

[BCG|B] -57.78 0.0004221

[BEG|B] -57.78 0.0004221

[BFH|B] -57.78 0.0004214

[BDH|B] -57.78 0.0004214

[BCF|B] -57.79 0.0004207

[BCD|B] -57.79 0.0004207

[BDE|B] -57.79 0.0004207

[BEF|B] -57.79 0.0004207

[BFG|B] -57.79 0.0004194

[BDG|B] -57.79 0.0004194

[B|DG] -57.79 0.0004187

[B|EG] -57.79 0.0004187

[B|CG] -57.79 0.0004187

[B|CE] -57.79 0.0004187

[B|DE] -57.79 0.0004187

[B|FG] -57.79 0.0004187

[B|DF] -57.79 0.0004187

[B|EF] -57.79 0.0004187

[B|CF] -57.79 0.0004187

[B|CD] -57.79 0.0004187

[BDF|B] -57.79 0.000418

[J|BG] -57.8 0.000417

[J|BD] -57.8 0.000417

[J|BE] -57.8 0.000417

[J|BF] -57.8 0.000417

[J|BC] -57.8 0.000417

[K|BG] -57.8 0.0004157

[K|BD] -57.8 0.0004157

[L|BG] -57.8 0.0004157

[L|BD] -57.8 0.0004157

[K|BE] -57.8 0.0004157

[L|BE] -57.8 0.0004157

[K|BF] -57.8 0.0004157

[L|BF] -57.8 0.0004157

[K|BC] -57.8 0.0004157

[L|BC] -57.8 0.0004157

[CEG|G] -57.8 0.0004142

[CEG|E] -57.8 0.0004142

[CEG|C] -57.8 0.0004142

[CEH|E] -57.8 0.0004138

[CEH|C] -57.8 0.0004138

[CEF|E] -57.8 0.0004131

[CDE|E] -57.8 0.0004131

[CDE|D] -57.8 0.0004131

[CEF|C] -57.8 0.0004131

[CEF|F] -57.8 0.0004131

[CDE|C] -57.8 0.0004131

[CGH|G] -57.81 0.0004128

[EGH|G] -57.81 0.0004128

[EGH|E] -57.81 0.0004128

[CGH|C] -57.81 0.0004128

[CEJ|J] -57.81 0.0004123

[CDG|G] -57.81 0.0004122

[CFG|G] -57.81 0.0004122

[CDG|D] -57.81 0.0004122

[EFG|G] -57.81 0.0004122

[EFG|E] -57.81 0.0004122

[CDG|C] -57.81 0.0004122

[CFG|C] -57.81 0.0004122

[CFG|F] -57.81 0.0004122

[EFG|F] -57.81 0.0004122

[DEG|G] -57.81 0.0004122

[DEG|E] -57.81 0.0004122

[DEG|D] -57.81 0.0004122

[CDH|D] -57.81 0.0004118

[EFH|E] -57.81 0.0004118

[DEH|E] -57.81 0.0004118

[CDH|C] -57.81 0.0004118

[DEH|D] -57.81 0.0004118

[EFH|F] -57.81 0.0004118

[CFH|C] -57.81 0.0004118

[CFH|F] -57.81 0.0004118

[CHJ|J] -57.81 0.0004113

[EHJ|J] -57.81 0.0004113

[B|ABG] -57.81 0.0004113

[B|ABE] -57.81 0.0004113

[B|ABD] -57.81 0.0004113

[B|ABF] -57.81 0.0004113

[B|ABC] -57.81 0.0004113

[EGJ|J] -57.81 0.0004113

[CGJ|J] -57.81 0.0004113

[CDF|D] -57.81 0.0004111

[DEF|E] -57.81 0.0004111

[DEF|D] -57.81 0.0004111

[CDF|C] -57.81 0.0004111

[CDF|F] -57.81 0.0004111

[DEF|F] -57.81 0.0004111

[CEK|K] -57.81 0.0004109

[CEL|L] -57.81 0.0004109

[DGH|G] -57.81 0.0004108

[DGH|D] -57.81 0.0004108

[FGH|G] -57.81 0.0004108

[FGH|F] -57.81 0.0004108

[EHK|K] -57.81 0.0004105

[CHK|K] -57.81 0.0004105

[CHL|L] -57.81 0.0004105

[EHL|L] -57.81 0.0004105

[GHJ|J] -57.81 0.0004102

[DFG|G] -57.81 0.0004102

[DFG|D] -57.81 0.0004102

[DFG|F] -57.81 0.0004102

[CEJ|E] -57.81 0.0004101

[CEJ|C] -57.81 0.0004101

[CFJ|J] -57.81 0.00041

[EFJ|J] -57.81 0.00041

[CDJ|J] -57.81 0.00041

[DEJ|J] -57.81 0.00041

[EGK|K] -57.81 0.0004099

[CGK|K] -57.81 0.0004099

[CGL|L] -57.81 0.0004099

[EGL|L] -57.81 0.0004099

[I|H] -57.81 0.0004098

[DFH|D] -57.81 0.0004097

[DFH|F] -57.81 0.0004097

[GHK|K] -57.81 0.0004095

[GHL|L] -57.81 0.0004095

[CHJ|C] -57.81 0.0004092

[EHJ|E] -57.81 0.0004092

[EGJ|G] -57.81 0.0004091

[EGJ|E] -57.81 0.0004091

[CGJ|G] -57.81 0.0004091

[CGJ|C] -57.81 0.0004091

[FHJ|J] -57.81 0.000409

[DHJ|J] -57.81 0.000409

[FGJ|J] -57.81 0.000409

[DGJ|J] -57.81 0.000409

[CEL|E] -57.82 0.0004087

[CEK|E] -57.82 0.0004087

[CEL|C] -57.82 0.0004087

[CEK|C] -57.82 0.0004087

[EFK|K] -57.82 0.0004087

[CFK|K] -57.82 0.0004087

[CDK|K] -57.82 0.0004087

[EFL|L] -57.82 0.0004087

[CDL|L] -57.82 0.0004087

[DEK|K] -57.82 0.0004087

[CFL|L] -57.82 0.0004087

[DEL|L] -57.82 0.0004087

[EHK|E] -57.82 0.0004084

[EHL|E] -57.82 0.0004084

[CHL|C] -57.82 0.0004084

[CHK|C] -57.82 0.0004084

[FHK|K] -57.82 0.0004083

[DHK|K] -57.82 0.0004083

[FHL|L] -57.82 0.0004083

[DHL|L] -57.82 0.0004083

[GHJ|G] -57.82 0.0004081

[EFJ|E] -57.82 0.0004079

[CFJ|C] -57.82 0.0004079

[CFJ|F] -57.82 0.0004079

[CDJ|D] -57.82 0.0004079

[DEJ|E] -57.82 0.0004079

[EFJ|F] -57.82 0.0004079

[DEJ|D] -57.82 0.0004079

[CDJ|C] -57.82 0.0004079

[DFJ|J] -57.82 0.0004077

[CGL|G] -57.82 0.0004077

[EGL|G] -57.82 0.0004077

[EGL|E] -57.82 0.0004077

[EGK|G] -57.82 0.0004077

[CGK|G] -57.82 0.0004077

[EGK|E] -57.82 0.0004077

[CGL|C] -57.82 0.0004077

[CGK|C] -57.82 0.0004077

[FGK|K] -57.82 0.0004076

[FGL|L] -57.82 0.0004076

[DGK|K] -57.82 0.0004076

[DGL|L] -57.82 0.0004076

[GHL|G] -57.82 0.0004073

[GHK|G] -57.82 0.0004073

[FHJ|F] -57.82 0.0004068

[DHJ|D] -57.82 0.0004068

[FGJ|G] -57.82 0.0004068

[FGJ|F] -57.82 0.0004068

[DGJ|G] -57.82 0.0004068

[DGJ|D] -57.82 0.0004068

[EFK|E] -57.82 0.0004065

[EFL|E] -57.82 0.0004065

[CDL|D] -57.82 0.0004065

[EFK|F] -57.82 0.0004065

[CDL|C] -57.82 0.0004065

[CDK|D] -57.82 0.0004065

[EFL|F] -57.82 0.0004065

[CFK|C] -57.82 0.0004065

[CFK|F] -57.82 0.0004065

[CDK|C] -57.82 0.0004065

[DEK|E] -57.82 0.0004065

[DEK|D] -57.82 0.0004065

[CFL|C] -57.82 0.0004065

[CFL|F] -57.82 0.0004065

[DEL|E] -57.82 0.0004065

[DEL|D] -57.82 0.0004065

[CE|K] -57.82 0.0004065

[CE|L] -57.82 0.0004065

[CE|J] -57.82 0.0004065

[DFK|K] -57.82 0.0004065

[DFL|L] -57.82 0.0004065

[FHK|F] -57.82 0.0004061

[DHK|D] -57.82 0.0004061

[FHL|F] -57.82 0.0004061

[DHL|D] -57.82 0.0004061

[DFJ|D] -57.82 0.0004056

[DFJ|F] -57.82 0.0004056

[CG|K] -57.82 0.0004056

[CG|L] -57.82 0.0004056

[EG|K] -57.82 0.0004056

[CG|J] -57.82 0.0004056

[EG|L] -57.82 0.0004056

[EG|J] -57.82 0.0004056

[FGL|G] -57.82 0.0004055

[FGK|G] -57.82 0.0004055

[FGL|F] -57.82 0.0004055

[FGK|F] -57.82 0.0004055

[DGK|G] -57.82 0.0004055

[DGK|D] -57.82 0.0004055

[DGL|G] -57.82 0.0004055

[DGL|D] -57.82 0.0004055

[J|BGJ] -57.83 0.0004047

[J|BDJ] -57.83 0.0004047

[J|BCJ] -57.83 0.0004047

[J|BFJ] -57.83 0.0004047

[J|BEJ] -57.83 0.0004047

[CD|K] -57.83 0.0004045

[CF|K] -57.83 0.0004045

[EF|K] -57.83 0.0004045

[CD|L] -57.83 0.0004045

[DE|K] -57.83 0.0004045

[CF|L] -57.83 0.0004045

[EF|L] -57.83 0.0004045

[CD|J] -57.83 0.0004045

[DE|L] -57.83 0.0004045

[CF|J] -57.83 0.0004045

[EF|J] -57.83 0.0004045

[DE|J] -57.83 0.0004045

[CE|G] -57.83 0.0004043

[CE|D] -57.83 0.0004043

[CE|F] -57.83 0.0004043

[DFK|D] -57.83 0.0004043

[DFK|F] -57.83 0.0004043

[DFL|D] -57.83 0.0004043

[DFL|F] -57.83 0.0004043

[CH|K] -57.83 0.0004043

[EH|K] -57.83 0.0004043

[CH|L] -57.83 0.0004043

[EH|L] -57.83 0.0004043

[CH|J] -57.83 0.0004043

[EH|J] -57.83 0.0004043

[FG|K] -57.83 0.0004036

[FG|L] -57.83 0.0004036

[DG|K] -57.83 0.0004036

[FG|J] -57.83 0.0004036

[DG|L] -57.83 0.0004036

[DG|J] -57.83 0.0004036

[L|BDL] -57.83 0.0004035

[K|BGK] -57.83 0.0004035

[L|BGL] -57.83 0.0004035

[L|BEL] -57.83 0.0004035

[K|BFK] -57.83 0.0004035

[K|BDK] -57.83 0.0004035

[K|BCK] -57.83 0.0004035

[K|BEK] -57.83 0.0004035

[L|BCL] -57.83 0.0004035

[L|BFL] -57.83 0.0004035

[CG|E] -57.83 0.0004034

[CG|D] -57.83 0.0004034

[EG|D] -57.83 0.0004034

[CG|F] -57.83 0.0004034

[EG|C] -57.83 0.0004034

[EG|F] -57.83 0.0004034

[GH|K] -57.83 0.0004033

[GH|L] -57.83 0.0004033

[GH|J] -57.83 0.0004033

[DF|K] -57.83 0.0004025

[DF|L] -57.83 0.0004025

[DF|J] -57.83 0.0004025

[CD|G] -57.83 0.0004024

[CD|E] -57.83 0.0004024

[CF|G] -57.83 0.0004024

[EF|G] -57.83 0.0004024

[CF|E] -57.83 0.0004024

[CF|D] -57.83 0.0004024

[EF|D] -57.83 0.0004024

[DE|G] -57.83 0.0004024

[EF|C] -57.83 0.0004024

[CD|F] -57.83 0.0004024

[DE|C] -57.83 0.0004024

[DE|F] -57.83 0.0004024

[FH|K] -57.83 0.0004023

[DH|K] -57.83 0.0004023

[FH|L] -57.83 0.0004023

[DH|L] -57.83 0.0004023

[FH|J] -57.83 0.0004023

[DH|J] -57.83 0.0004023

[CH|G] -57.83 0.0004021

[CH|E] -57.83 0.0004021

[EH|G] -57.83 0.0004021

[CH|D] -57.83 0.0004021

[EH|D] -57.83 0.0004021

[EH|C] -57.83 0.0004021

[CH|F] -57.83 0.0004021

[EH|F] -57.83 0.0004021

[FG|E] -57.83 0.0004014

[FG|D] -57.83 0.0004014

[FG|C] -57.83 0.0004014

[DG|E] -57.83 0.0004014

[DG|C] -57.83 0.0004014

[DG|F] -57.83 0.0004014

[CJK|K] -57.83 0.0004013

[EJK|K] -57.83 0.0004013

[CJL|L] -57.83 0.0004013

[EJL|L] -57.83 0.0004013

[CJK|J] -57.83 0.0004013

[EJK|J] -57.83 0.0004013

[CJL|J] -57.83 0.0004013

[EJL|J] -57.83 0.0004013

[HJK|K] -57.83 0.0004013

[HJL|L] -57.83 0.0004013

[HJK|J] -57.83 0.0004013

[HJL|J] -57.83 0.0004013

[GH|E] -57.83 0.0004012

[GH|D] -57.83 0.0004012

[GH|C] -57.83 0.0004012

[GH|F] -57.83 0.0004012

[HKL|K] -57.84 0.0004007

[HKL|L] -57.84 0.0004007

[DF|G] -57.84 0.0004004

[DF|E] -57.84 0.0004004

[DF|C] -57.84 0.0004004

[EJ|K] -57.84 0.0004002

[CJ|K] -57.84 0.0004002

[EJ|L] -57.84 0.0004002

[CJ|L] -57.84 0.0004002

[FH|G] -57.84 0.0004002

[FH|E] -57.84 0.0004002

[DH|G] -57.84 0.0004002

[FH|D] -57.84 0.0004002

[DH|E] -57.84 0.0004002

[FH|C] -57.84 0.0004002

[DH|C] -57.84 0.0004002

[DH|F] -57.84 0.0004002

[GJK|K] -57.84 0.0004001

[GJK|J] -57.84 0.0004001

[GJL|L] -57.84 0.0004001

[GJL|J] -57.84 0.0004001

[CKL|K] -57.84 0.0004001

[CKL|L] -57.84 0.0004001

[EKL|K] -57.84 0.0004001

[EKL|L] -57.84 0.0004001

[EJK|E] -57.84 0.0003992

[EJL|E] -57.84 0.0003992

[CJK|C] -57.84 0.0003992

[CJL|C] -57.84 0.0003992

[GJ|K] -57.84 0.000399

[GJ|L] -57.84 0.000399

[HI|B] -57.84 0.000399

[CL|K] -57.84 0.000399

[EL|K] -57.84 0.000399

[CK|L] -57.84 0.000399

[CL|J] -57.84 0.000399

[CK|J] -57.84 0.000399

[EK|L] -57.84 0.000399

[EL|J] -57.84 0.000399

[EK|J] -57.84 0.000399

[GKL|K] -57.84 0.0003989

[GKL|L] -57.84 0.0003989

[FJK|K] -57.84 0.0003988

[FJL|L] -57.84 0.0003988

[FJL|J] -57.84 0.0003988

[FJK|J] -57.84 0.0003988

[DJK|K] -57.84 0.0003988

[DJL|L] -57.84 0.0003988

[DJK|J] -57.84 0.0003988

[DJL|J] -57.84 0.0003988

[HJ|K] -57.84 0.0003988

[HJ|L] -57.84 0.0003988

[HK|L] -57.84 0.0003985

[HL|K] -57.84 0.0003985

[HK|J] -57.84 0.0003985

[HL|J] -57.84 0.0003985

[EJ|G] -57.84 0.0003981

[EJ|D] -57.84 0.0003981

[CJ|G] -57.84 0.0003981

[CJ|E] -57.84 0.0003981

[CJ|D] -57.84 0.0003981

[EJ|C] -57.84 0.0003981

[EJ|F] -57.84 0.0003981

[CJ|F] -57.84 0.0003981

[GJK|G] -57.84 0.000398

[GJL|G] -57.84 0.000398

[EKL|E] -57.84 0.000398

[CKL|C] -57.84 0.000398

[GL|K] -57.84 0.0003978

[GK|L] -57.84 0.0003978

[GK|J] -57.84 0.0003978

[GL|J] -57.84 0.0003978

[FJ|K] -57.84 0.0003977

[FJ|L] -57.84 0.0003977

[DJ|K] -57.84 0.0003977

[DJ|L] -57.84 0.0003977

[FKL|K] -57.84 0.0003976

[FKL|L] -57.84 0.0003976

[DKL|K] -57.84 0.0003976

[DKL|L] -57.84 0.0003976

[H|KL] -57.84 0.0003971

[H|JK] -57.84 0.0003971

[H|JL] -57.84 0.0003971

[GJ|E] -57.84 0.0003969

[GJ|D] -57.84 0.0003969

[GJ|C] -57.84 0.0003969

[GJ|F] -57.84 0.0003969

[CL|G] -57.85 0.0003969

[CK|G] -57.85 0.0003969

[CL|E] -57.85 0.0003969

[CK|E] -57.85 0.0003969

[CL|D] -57.85 0.0003969

[CK|D] -57.85 0.0003969

[EL|G] -57.85 0.0003969

[EK|G] -57.85 0.0003969

[EL|D] -57.85 0.0003969

[EK|D] -57.85 0.0003969

[CL|F] -57.85 0.0003969

[CK|F] -57.85 0.0003969

[EL|C] -57.85 0.0003969

[EK|C] -57.85 0.0003969

[EL|F] -57.85 0.0003969

[EK|F] -57.85 0.0003969

[GKL|G] -57.85 0.0003968

[FJL|F] -57.85 0.0003967

[FJK|F] -57.85 0.0003967

[DJK|D] -57.85 0.0003967

[DJL|D] -57.85 0.0003967

[HJ|G] -57.85 0.0003967

[HJ|E] -57.85 0.0003967

[HJ|D] -57.85 0.0003967

[HJ|C] -57.85 0.0003967

[HJ|F] -57.85 0.0003967

[FK|L] -57.85 0.0003965

[FL|K] -57.85 0.0003965

[FK|J] -57.85 0.0003965

[DL|K] -57.85 0.0003965

[FL|J] -57.85 0.0003965

[DK|L] -57.85 0.0003965

[DK|J] -57.85 0.0003965

[DL|J] -57.85 0.0003965

[HK|G] -57.85 0.0003963

[HK|E] -57.85 0.0003963

[HK|D] -57.85 0.0003963

[HL|G] -57.85 0.0003963

[HK|C] -57.85 0.0003963

[HL|E] -57.85 0.0003963

[HK|F] -57.85 0.0003963

[HL|D] -57.85 0.0003963

[HL|C] -57.85 0.0003963

[HL|F] -57.85 0.0003963

[C|KL] -57.85 0.0003962

[E|KL] -57.85 0.0003962

[C|JK] -57.85 0.0003962

[C|JL] -57.85 0.0003962

[E|JK] -57.85 0.0003962

[E|JL] -57.85 0.0003962

[GK|E] -57.85 0.0003957

[GL|E] -57.85 0.0003957

[GK|D] -57.85 0.0003957

[GL|D] -57.85 0.0003957

[GK|C] -57.85 0.0003957

[GL|C] -57.85 0.0003957

[GK|F] -57.85 0.0003957

[GL|F] -57.85 0.0003957

[FJ|G] -57.85 0.0003956

[FJ|E] -57.85 0.0003956

[FJ|D] -57.85 0.0003956

[FJ|C] -57.85 0.0003956

[DJ|G] -57.85 0.0003956

[DJ|E] -57.85 0.0003956

[DJ|C] -57.85 0.0003956

[DJ|F] -57.85 0.0003956

[FKL|F] -57.85 0.0003955

[DKL|D] -57.85 0.0003955

[G|KL] -57.85 0.000395

[G|JK] -57.85 0.000395

[G|JL] -57.85 0.000395

[FK|G] -57.85 0.0003944

[FK|E] -57.85 0.0003944

[FK|D] -57.85 0.0003944

[FK|C] -57.85 0.0003944

[FL|G] -57.85 0.0003944

[FL|E] -57.85 0.0003944

[FL|D] -57.85 0.0003944

[DK|G] -57.85 0.0003944

[DK|E] -57.85 0.0003944

[FL|C] -57.85 0.0003944

[DL|G] -57.85 0.0003944

[DL|E] -57.85 0.0003944

[DK|C] -57.85 0.0003944

[DK|F] -57.85 0.0003944

[DL|C] -57.85 0.0003944

[DL|F] -57.85 0.0003944

[F|KL] -57.85 0.0003936

[D|KL] -57.85 0.0003936

[F|JK] -57.85 0.0003936

[F|JL] -57.85 0.0003936

[D|JK] -57.85 0.0003936

[D|JL] -57.85 0.0003936

[B|BDG] -57.85 0.0003934

[B|BDE] -57.85 0.0003934

[B|BFG] -57.85 0.0003934

[B|BEF] -57.85 0.0003934

[B|BEG] -57.85 0.0003934

[B|BDF] -57.85 0.0003934

[B|BCE] -57.85 0.0003934

[B|BCF] -57.85 0.0003934

[B|BCD] -57.85 0.0003934

[B|BCG] -57.85 0.0003934

[H|GL] -57.86 0.0003895

[H|GK] -57.86 0.0003895

[H|FK] -57.86 0.0003895

[H|GJ] -57.86 0.0003895

[H|EK] -57.86 0.0003895

[H|DK] -57.86 0.0003895

[H|EL] -57.86 0.0003895

[H|FL] -57.86 0.0003895

[H|EJ] -57.86 0.0003895

[H|DL] -57.86 0.0003895

[H|DJ] -57.86 0.0003895

[H|CL] -57.86 0.0003895

[H|CK] -57.86 0.0003895

[H|CJ] -57.86 0.0003895

[H|FJ] -57.86 0.0003895

[C|GL] -57.87 0.0003886

[C|GK] -57.87 0.0003886

[C|FK] -57.87 0.0003886

[E|GL] -57.87 0.0003886

[C|GJ] -57.87 0.0003886

[E|GK] -57.87 0.0003886

[C|EK] -57.87 0.0003886

[C|DK] -57.87 0.0003886

[C|EL] -57.87 0.0003886

[E|FK] -57.87 0.0003886

[C|FL] -57.87 0.0003886

[C|EJ] -57.87 0.0003886

[C|DL] -57.87 0.0003886

[C|DJ] -57.87 0.0003886

[E|GJ] -57.87 0.0003886

[E|DK] -57.87 0.0003886

[E|FL] -57.87 0.0003886

[E|DL] -57.87 0.0003886

[E|DJ] -57.87 0.0003886

[E|CL] -57.87 0.0003886

[E|CK] -57.87 0.0003886

[C|FJ] -57.87 0.0003886

[E|CJ] -57.87 0.0003886

[E|FJ] -57.87 0.0003886

[H|DG] -57.87 0.000388

[H|EG] -57.87 0.000388

[H|CG] -57.87 0.000388

[H|DE] -57.87 0.000388

[H|CE] -57.87 0.000388

[H|FG] -57.87 0.000388

[H|DF] -57.87 0.000388

[H|EF] -57.87 0.000388

[H|CF] -57.87 0.000388

[H|CD] -57.87 0.000388

[G|FK] -57.87 0.0003874

[G|EK] -57.87 0.0003874

[G|DK] -57.87 0.0003874

[G|EL] -57.87 0.0003874

[G|FL] -57.87 0.0003874

[G|EJ] -57.87 0.0003874

[G|DL] -57.87 0.0003874

[G|DJ] -57.87 0.0003874

[G|CL] -57.87 0.0003874

[G|CK] -57.87 0.0003874

[G|CJ] -57.87 0.0003874

[G|FJ] -57.87 0.0003874

[C|DG] -57.87 0.0003871

[C|EG] -57.87 0.0003871

[E|DG] -57.87 0.0003871

[C|DE] -57.87 0.0003871

[C|FG] -57.87 0.0003871

[C|DF] -57.87 0.0003871

[E|CG] -57.87 0.0003871

[E|FG] -57.87 0.0003871

[C|EF] -57.87 0.0003871

[E|DF] -57.87 0.0003871

[E|CF] -57.87 0.0003871

[E|CD] -57.87 0.0003871

[F|GL] -57.87 0.0003861

[F|GK] -57.87 0.0003861

[F|GJ] -57.87 0.0003861

[D|GL] -57.87 0.0003861

[F|EK] -57.87 0.0003861

[F|DK] -57.87 0.0003861

[D|GK] -57.87 0.0003861

[F|EL] -57.87 0.0003861

[F|EJ] -57.87 0.0003861

[D|FK] -57.87 0.0003861

[F|DL] -57.87 0.0003861

[F|DJ] -57.87 0.0003861

[F|CL] -57.87 0.0003861

[F|CK] -57.87 0.0003861

[D|GJ] -57.87 0.0003861

[F|CJ] -57.87 0.0003861

[D|EK] -57.87 0.0003861

[D|EL] -57.87 0.0003861

[D|FL] -57.87 0.0003861

[D|EJ] -57.87 0.0003861

[D|CL] -57.87 0.0003861

[D|CK] -57.87 0.0003861

[D|CJ] -57.87 0.0003861

[D|FJ] -57.87 0.0003861

[JKL|K] -57.87 0.0003859

[JKL|L] -57.87 0.0003859

[JKL|J] -57.87 0.0003859

[G|DE] -57.87 0.0003859

[G|CE] -57.87 0.0003859

[G|DF] -57.87 0.0003859

[G|EF] -57.87 0.0003859

[G|CF] -57.87 0.0003859

[G|CD] -57.87 0.0003859

[F|DG] -57.88 0.0003846

[F|EG] -57.88 0.0003846

[F|CG] -57.88 0.0003846

[F|DE] -57.88 0.0003846

[F|CE] -57.88 0.0003846

[D|EG] -57.88 0.0003846

[D|CG] -57.88 0.0003846

[D|CE] -57.88 0.0003846

[D|FG] -57.88 0.0003846

[F|CD] -57.88 0.0003846

[D|EF] -57.88 0.0003846

[D|CF] -57.88 0.0003846

[JL|K] -57.88 0.0003822

[JK|L] -57.88 0.0003822

[E|EKL] -57.88 0.0003816

[C|CJL] -57.88 0.0003816

[C|CKL] -57.88 0.0003816

[C|CJK] -57.88 0.0003816

[E|EJK] -57.88 0.0003816

[E|EJL] -57.88 0.0003816

[KL|J] -57.89 0.0003812

[G|GKL] -57.89 0.0003805

[G|GJL] -57.89 0.0003805

[G|GJK] -57.89 0.0003805

[E|ABE] -57.89 0.0003802

[C|ABC] -57.89 0.0003802

[JK|G] -57.89 0.0003802

[JL|G] -57.89 0.0003802

[JK|E] -57.89 0.0003802

[JL|E] -57.89 0.0003802

[JK|D] -57.89 0.0003802

[JL|D] -57.89 0.0003802

[JK|C] -57.89 0.0003802

[JL|C] -57.89 0.0003802

[JK|F] -57.89 0.0003802

[JL|F] -57.89 0.0003802

[KL|G] -57.89 0.0003792

[KL|E] -57.89 0.0003792

[KL|D] -57.89 0.0003792

[KL|C] -57.89 0.0003792

[KL|F] -57.89 0.0003792

[F|FJK] -57.89 0.0003792

[F|FKL] -57.89 0.0003792

[D|DKL] -57.89 0.0003792

[F|FJL] -57.89 0.0003792

[D|DJK] -57.89 0.0003792

[D|DJL] -57.89 0.0003792

[G|ABG] -57.89 0.0003791

[C|CEL] -57.89 0.0003781

[C|CEJ] -57.89 0.0003781

[E|EGL] -57.89 0.0003781

[C|CGK] -57.89 0.0003781

[C|CGL] -57.89 0.0003781

[E|EGK] -57.89 0.0003781

[E|EGJ] -57.89 0.0003781

[E|CEL] -57.89 0.0003781

[E|EFK] -57.89 0.0003781

[C|CGJ] -57.89 0.0003781

[E|CEJ] -57.89 0.0003781

[E|DEL] -57.89 0.0003781

[E|EFL] -57.89 0.0003781

[C|CFK] -57.89 0.0003781

[C|CEK] -57.89 0.0003781

[E|DEJ] -57.89 0.0003781

[C|CDK] -57.89 0.0003781

[E|DEK] -57.89 0.0003781

[C|CDJ] -57.89 0.0003781

[C|CDL] -57.89 0.0003781

[C|CFL] -57.89 0.0003781

[E|CEK] -57.89 0.0003781

[E|EFJ] -57.89 0.0003781

[C|CFJ] -57.89 0.0003781

[F|ABF] -57.89 0.0003778

[D|ABD] -57.89 0.0003778

[C|CEG] -57.9 0.0003771

[C|CDG] -57.9 0.0003771

[C|CEF] -57.9 0.0003771

[E|CEG] -57.9 0.0003771

[C|CFG] -57.9 0.0003771

[C|CDE] -57.9 0.0003771

[E|DEG] -57.9 0.0003771

[E|CEF] -57.9 0.0003771

[E|EFG] -57.9 0.0003771

[E|CDE] -57.9 0.0003771

[E|DEF] -57.9 0.0003771

[C|CDF] -57.9 0.0003771

[G|FGK] -57.9 0.0003769

[G|FGL] -57.9 0.0003769

[G|EGL] -57.9 0.0003769

[G|EGK] -57.9 0.0003769

[G|EGJ] -57.9 0.0003769

[G|DGK] -57.9 0.0003769

[G|CGK] -57.9 0.0003769

[G|DGL] -57.9 0.0003769

[G|CGL] -57.9 0.0003769

[G|DGJ] -57.9 0.0003769

[G|CGJ] -57.9 0.0003769

[G|FGJ] -57.9 0.0003769

[G|DFG] -57.9 0.0003759

[G|CEG] -57.9 0.0003759

[G|DEG] -57.9 0.0003759

[G|CDG] -57.9 0.0003759

[G|CFG] -57.9 0.0003759

[G|EFG] -57.9 0.0003759

[F|FGK] -57.9 0.0003757

[F|FGL] -57.9 0.0003757

[F|EFK] -57.9 0.0003757

[F|DFK] -57.9 0.0003757

[F|EFL] -57.9 0.0003757

[F|DFL] -57.9 0.0003757

[D|DFK] -57.9 0.0003757

[D|DGK] -57.9 0.0003757

[D|DGL] -57.9 0.0003757

[D|DEL] -57.9 0.0003757

[F|CFK] -57.9 0.0003757

[D|DGJ] -57.9 0.0003757

[F|DFJ] -57.9 0.0003757

[D|DFL] -57.9 0.0003757

[D|DEJ] -57.9 0.0003757

[F|EFJ] -57.9 0.0003757

[D|DEK] -57.9 0.0003757

[F|FGJ] -57.9 0.0003757

[F|CFL] -57.9 0.0003757

[D|DFJ] -57.9 0.0003757

[D|CDK] -57.9 0.0003757

[F|CFJ] -57.9 0.0003757

[D|CDJ] -57.9 0.0003757

[D|CDL] -57.9 0.0003757

[F|DFG] -57.9 0.0003747

[F|CEF] -57.9 0.0003747

[D|DFG] -57.9 0.0003747

[F|EFG] -57.9 0.0003747

[F|CFG] -57.9 0.0003747

[D|DEG] -57.9 0.0003747

[D|CDG] -57.9 0.0003747

[F|DEF] -57.9 0.0003747

[D|CDE] -57.9 0.0003747

[D|DEF] -57.9 0.0003747

[F|CDF] -57.9 0.0003747

[D|CDF] -57.9 0.0003747

[CE|B] -57.9 0.0003741

[CG|B] -57.91 0.0003733

[EG|B] -57.91 0.0003733

[J|KL] -57.91 0.0003731

[CD|B] -57.91 0.0003723

[CF|B] -57.91 0.0003723

[EF|B] -57.91 0.0003723

[DE|B] -57.91 0.0003723

[CH|B] -57.91 0.0003721

[EH|B] -57.91 0.0003721

[L|JK] -57.91 0.0003719

[K|JL] -57.91 0.0003719

[H|AB] -57.91 0.0003717

[FG|B] -57.91 0.0003714

[DG|B] -57.91 0.0003714

[GH|B] -57.91 0.0003712

[C|AB] -57.91 0.0003708

[E|AB] -57.91 0.0003708

[DF|B] -57.91 0.0003705

[FH|B] -57.91 0.0003703

[DH|B] -57.91 0.0003703

[H|BG] -57.92 0.0003699

[H|BD] -57.92 0.0003699

[H|BE] -57.92 0.0003699

[H|BF] -57.92 0.0003699

[H|BC] -57.92 0.0003699

[G|AB] -57.92 0.0003697

[C|BG] -57.92 0.000369

[C|BD] -57.92 0.000369

[C|BE] -57.92 0.000369

[E|BG] -57.92 0.000369

[E|BD] -57.92 0.000369

[C|BF] -57.92 0.000369

[E|BF] -57.92 0.000369

[E|BC] -57.92 0.000369

[F|AB] -57.92 0.0003684

[D|AB] -57.92 0.0003684

[G|BD] -57.92 0.0003679

[G|BE] -57.92 0.0003679

[G|BF] -57.92 0.0003679

[G|BC] -57.92 0.0003679

[F|BG] -57.92 0.0003667

[F|BD] -57.92 0.0003667

[F|BE] -57.92 0.0003667

[D|BG] -57.92 0.0003667

[F|BC] -57.92 0.0003667

[D|BE] -57.92 0.0003667

[D|BF] -57.92 0.0003667

[D|BC] -57.92 0.0003667

[J|GL] -57.93 0.0003659

[J|GK] -57.93 0.0003659

[J|FK] -57.93 0.0003659

[J|EK] -57.93 0.0003659

[J|DK] -57.93 0.0003659

[J|EL] -57.93 0.0003659

[J|FL] -57.93 0.0003659

[J|DL] -57.93 0.0003659

[J|CL] -57.93 0.0003659

[J|CK] -57.93 0.0003659

[J|JKL] -57.93 0.0003651

[K|GL] -57.93 0.0003648

[L|GK] -57.93 0.0003648

[L|FK] -57.93 0.0003648

[K|GJ] -57.93 0.0003648

[L|GJ] -57.93 0.0003648

[L|EK] -57.93 0.0003648

[K|EL] -57.93 0.0003648

[K|FL] -57.93 0.0003648

[L|DK] -57.93 0.0003648

[K|EJ] -57.93 0.0003648

[K|DL] -57.93 0.0003648

[L|EJ] -57.93 0.0003648

[K|DJ] -57.93 0.0003648

[K|CL] -57.93 0.0003648

[L|DJ] -57.93 0.0003648

At node N20:

split lnL Rel.Prob

[B|B] -54.17 0.01564

[H|H] -54.84 0.008041

[C|C] -55.01 0.00676

[E|E] -55.01 0.00676

[G|G] -55.04 0.006546

[F|F] -55.08 0.006303

[D|D] -55.08 0.006303

[J|J] -55.26 0.005272

[L|L] -55.29 0.005108

[K|K] -55.29 0.005108

[B|BH] -55.81 0.003032

[B|BHL] -55.83 0.002962

[B|BHK] -55.83 0.002962

[B|ABH] -55.99 0.002549

[B|AB] -56.11 0.002258

[BC|B] -56.17 0.002126

[BE|B] -56.17 0.002126

[BG|B] -56.2 0.002046

[BF|B] -56.25 0.001955

[BD|B] -56.25 0.001955

[BEJ|B] -56.29 0.001886

[BCJ|B] -56.29 0.001886

[BGJ|B] -56.35 0.001763

[B|BHJ] -56.36 0.001755

[B|BHI] -56.4 0.001686

[AB|B] -56.42 0.001651

[B|BF] -56.43 0.001631

[B|BC] -56.43 0.001631

[B|BE] -56.43 0.001631

[B|BD] -56.43 0.001631

[B|BG] -56.43 0.001631

[BDJ|B] -56.44 0.001622

[BFJ|B] -56.44 0.001622

[H|BH] -56.47 0.001562

[H|BHL] -56.5 0.001526

[H|BHK] -56.5 0.001526

[BH|B] -56.51 0.001503

[B|H] -56.53 0.001473

[L|BHL] -56.54 0.001463

[K|BHK] -56.54 0.001463

[B|ABC] -56.56 0.001439

[B|ABD] -56.56 0.001439

[B|ABF] -56.56 0.001439

[B|ABG] -56.56 0.001439

[B|ABE] -56.56 0.001439

[J|BH] -56.6 0.001373

[H|ABH] -56.65 0.001313

[ABE|B] -56.66 0.001297

[ABC|B] -56.66 0.001297

[BH|H] -56.67 0.001282

[J|BHJ] -56.67 0.001279

[A|A] -56.68 0.001275

[B|HL] -56.69 0.001265

[B|HK] -56.69 0.001265

[ABG|B] -56.7 0.001249

[C|B] -56.72 0.00122

[E|B] -56.72 0.00122

[C|BC] -56.73 0.001215

[E|BE] -56.73 0.001215

[ABD|B] -56.74 0.001196

[ABF|B] -56.74 0.001196

[G|B] -56.75 0.001181

[G|BG] -56.76 0.001176

[B|BCH] -56.76 0.001174

[B|BEH] -56.76 0.001174

[B|BDH] -56.76 0.001174

[B|BGH] -56.76 0.001174

[B|BFH] -56.76 0.001174

[BCK|B] -56.77 0.001165

[BEL|B] -56.77 0.001165

[BCL|B] -56.77 0.001165

[BEK|B] -56.77 0.001165

[BCE|B] -56.79 0.001141

[F|B] -56.79 0.001137

[D|B] -56.79 0.001137

[F|BF] -56.8 0.001132

[D|BD] -56.8 0.001132

[BC|C] -56.8 0.001125

[BE|E] -56.8 0.001125

[C|BH] -56.8 0.001124

[E|BH] -56.8 0.001124

[BEG|B] -56.81 0.001118

[BCG|B] -56.81 0.001118

[C|H] -56.81 0.001114

[E|H] -56.81 0.001114

[BGL|B] -56.82 0.001102

[BGK|B] -56.82 0.001102

[J|H] -56.83 0.001095

[C|AB] -56.84 0.001089

[E|AB] -56.84 0.001089

[G|BH] -56.84 0.001088

[BG|G] -56.84 0.001083

[BCF|B] -56.84 0.001079

[BEF|B] -56.84 0.001079

[BCD|B] -56.84 0.001079

[BDE|B] -56.84 0.001079

[G|H] -56.85 0.001079

[C|ABC] -56.85 0.001072

[E|ABE] -56.85 0.001072

[H|HL] -56.85 0.00107

[H|HK] -56.85 0.00107

[HJ|H] -56.85 0.001069

[C|CH] -56.86 0.001063

[E|EH] -56.86 0.001063

[G|AB] -56.87 0.001054

[F|BH] -56.87 0.001048

[D|BH] -56.87 0.001048

[J|HJ] -56.88 0.001046

[EJ|B] -56.88 0.00104

[CJ|B] -56.88 0.00104

[BFG|B] -56.88 0.001039

[BDG|B] -56.88 0.001039

[F|H] -56.88 0.001039

[D|H] -56.88 0.001039

[CH|H] -56.88 0.001039

[EH|H] -56.88 0.001039

[G|ABG] -56.88 0.001038

[BF|F] -56.89 0.001035

[BD|D] -56.89 0.001035

[B|BCK] -56.89 0.001033

[B|BDK] -56.89 0.001033

[B|BCL] -56.89 0.001033

[B|BFL] -56.89 0.001033

[B|BGK] -56.89 0.001033

[B|BFK] -56.89 0.001033

[B|BGL] -56.89 0.001033

[B|BEK] -56.89 0.001033

[B|BDL] -56.89 0.001033

[B|BEL] -56.89 0.001033

[BFL|B] -56.89 0.00103

[BDK|B] -56.89 0.00103

[BFK|B] -56.89 0.00103

[BDL|B] -56.89 0.00103

[G|GH] -56.89 0.00103

[L|HL] -56.9 0.001026

[K|HK] -56.9 0.001026

[F|AB] -56.91 0.001015

[D|AB] -56.91 0.001015

[GH|H] -56.91 0.001011

[GJ|B] -56.92 0.001005

[BDF|B] -56.92 0.001002

[F|ABF] -56.92 0.0009991

[D|ABD] -56.92 0.0009991

[BEJ|E] -56.92 0.0009981

[BCJ|C] -56.92 0.0009981

[F|FH] -56.93 0.0009914

[D|DH] -56.93 0.0009914

[FH|H] -56.94 0.0009799

[DH|H] -56.94 0.0009799

[BHJ|B] -56.94 0.0009772

[DJ|B] -56.96 0.0009639

[FJ|B] -56.96 0.0009639

[ABH|B] -56.97 0.0009543

[BC|H] -56.97 0.0009517

[BE|H] -56.97 0.0009517

[AB|H] -56.97 0.0009477

[AB|A] -56.98 0.0009386

[B|HI] -56.99 0.0009367

[BGJ|G] -56.99 0.0009331

[K|BH] -56.99 0.0009305

[L|BH] -56.99 0.0009305

[CJ|C] -57 0.0009218

[EJ|E] -57 0.0009218

[BG|H] -57.01 0.0009158

[H|BHJ] -57.02 0.0009037

[BCH|B] -57.03 0.0008977

[BEH|B] -57.03 0.0008977

[GJ|G] -57.04 0.0008901

[H|B] -57.04 0.0008898

[BF|H] -57.05 0.0008751

[BD|H] -57.05 0.0008751

[C|BCH] -57.06 0.0008744

[E|BEH] -57.06 0.0008744

[B|HJ] -57.06 0.0008741

[C|CL] -57.06 0.000869

[C|CK] -57.06 0.000869

[E|EL] -57.06 0.000869

[E|EK] -57.06 0.000869

[H|BHI] -57.06 0.0008687

[BGH|B] -57.06 0.0008675

[B|F] -57.06 0.0008667

[B|C] -57.06 0.0008667

[B|D] -57.06 0.0008667

[B|G] -57.06 0.0008667

[B|E] -57.06 0.0008667

[A|ABH] -57.07 0.0008591

[BFJ|F] -57.07 0.0008585

[BDJ|D] -57.07 0.0008585

[FJ|F] -57.08 0.0008541

[DJ|D] -57.08 0.0008541

[G|BGH] -57.09 0.0008466

[G|GL] -57.09 0.0008414

[G|GK] -57.09 0.0008414

[BHJ|H] -57.1 0.0008332

[BFH|B] -57.1 0.0008331

[BDH|B] -57.1 0.0008331

[F|BFH] -57.13 0.0008152

[D|BDH] -57.13 0.0008152

[ABH|H] -57.13 0.0008136

[F|FL] -57.13 0.0008102

[F|FK] -57.13 0.0008102

[D|DK] -57.13 0.0008102

[D|DL] -57.13 0.0008102

[CE|C] -57.13 0.0008092

[CE|E] -57.13 0.0008092

[BEJ|J] -57.13 0.0008076

[BCJ|J] -57.13 0.0008076

[B|A] -57.14 0.0008028

[BC|L] -57.15 0.0007992

[BE|L] -57.15 0.0007992

[BC|K] -57.15 0.0007992

[BE|K] -57.15 0.0007992

[CG|C] -57.15 0.0007949

[EG|G] -57.15 0.0007949

[EG|E] -57.15 0.0007949

[CG|G] -57.15 0.0007949

[H|HI] -57.16 0.0007906

[H|AB] -57.17 0.0007833

[EJ|H] -57.18 0.0007739

[CJ|H] -57.18 0.0007739

[CF|F] -57.18 0.0007725

[EF|F] -57.18 0.0007725

[CF|C] -57.18 0.0007725

[CD|C] -57.18 0.0007725

[CD|D] -57.18 0.0007725

[DE|D] -57.18 0.0007725

[EF|E] -57.18 0.0007725

[DE|E] -57.18 0.0007725

[HL|H] -57.18 0.000771

[HK|H] -57.18 0.000771

[C|HL] -57.18 0.0007701

[C|HK] -57.18 0.0007701

[E|HL] -57.18 0.0007701

[E|HK] -57.18 0.0007701

[C|BCK] -57.18 0.0007692

[C|BCL] -57.18 0.0007692

[E|BEK] -57.18 0.0007692

[E|BEL] -57.18 0.0007692

[BG|L] -57.18 0.000769

[BG|K] -57.18 0.000769

[C|L] -57.19 0.0007675

[C|K] -57.19 0.0007675

[E|L] -57.19 0.0007675

[E|K] -57.19 0.0007675

[BCH|H] -57.19 0.0007654

[BEH|H] -57.19 0.0007654

[HJ|B] -57.19 0.0007639

[A|AB] -57.19 0.0007611

[J|HL] -57.2 0.000756

[J|HK] -57.2 0.000756

[BGJ|J] -57.2 0.000755

[CE|B] -57.21 0.0007523

[J|L] -57.21 0.0007504

[J|K] -57.21 0.0007504

[FG|F] -57.21 0.0007503

[DG|D] -57.21 0.0007503

[DG|G] -57.21 0.0007503

[FG|G] -57.21 0.0007503

[CL|L] -57.21 0.0007498

[EL|L] -57.21 0.0007498

[CK|K] -57.21 0.0007498

[EK|K] -57.21 0.0007498

[GJ|H] -57.21 0.0007473

[EJ|J] -57.21 0.0007459

[CJ|J] -57.21 0.0007459

[G|HL] -57.21 0.0007457

[G|HK] -57.21 0.0007457

[G|BGK] -57.22 0.0007447

[G|BGL] -57.22 0.0007447

[G|L] -57.22 0.0007432

[G|K] -57.22 0.0007432

[L|H] -57.22 0.000742

[K|H] -57.22 0.000742

[BGH|H] -57.22 0.0007396

[H|HJ] -57.22 0.0007391

[EG|B] -57.22 0.000739

[CG|B] -57.22 0.000739

[ABE|A] -57.23 0.0007375

[ABC|A] -57.23 0.0007375

[B|BFJ] -57.23 0.0007367

[B|BEJ] -57.23 0.0007367

[B|BCJ] -57.23 0.0007367

[B|BGJ] -57.23 0.0007367

[B|BDJ] -57.23 0.0007367

[H|GH] -57.23 0.0007354

[H|CH] -57.23 0.0007354

[H|DH] -57.23 0.0007354

[H|EH] -57.23 0.0007354

[H|FH] -57.23 0.0007354

[BF|L] -57.23 0.0007348

[BD|L] -57.23 0.0007348

[BF|K] -57.23 0.0007348

[BD|K] -57.23 0.0007348

[CK|B] -57.23 0.0007315

[EL|B] -57.23 0.0007315

[CL|B] -57.23 0.0007315

[EK|B] -57.23 0.0007315

[J|BC] -57.24 0.0007298

[J|BF] -57.24 0.0007298

[J|BD] -57.24 0.0007298

[J|BE] -57.24 0.0007298

[J|BG] -57.24 0.0007298

[DF|F] -57.24 0.0007291

[DF|D] -57.24 0.0007291

[GL|L] -57.24 0.0007267

[GK|K] -57.24 0.0007267

[J|JL] -57.25 0.0007222

[J|JK] -57.25 0.0007222

[B|BEF] -57.25 0.0007216

[B|BCD] -57.25 0.0007216

[B|BDE] -57.25 0.0007216

[B|BCE] -57.25 0.0007216

[B|BDF] -57.25 0.0007216

[B|BCF] -57.25 0.0007216

[B|BFG] -57.25 0.0007216

[B|BCG] -57.25 0.0007216

[B|BDG] -57.25 0.0007216

[B|BEG] -57.25 0.0007216

[GJ|J] -57.25 0.0007203

[CF|B] -57.25 0.0007182

[EF|B] -57.25 0.0007182

[CD|B] -57.25 0.0007182

[DE|B] -57.25 0.0007182

[F|HL] -57.25 0.000718

[F|HK] -57.25 0.000718

[D|HL] -57.25 0.000718

[D|HK] -57.25 0.000718

[F|BFL] -57.25 0.0007171

[D|BDK] -57.25 0.0007171

[F|BFK] -57.25 0.0007171

[D|BDL] -57.25 0.0007171

[DJ|H] -57.25 0.000717

[FJ|H] -57.25 0.000717

[F|L] -57.26 0.0007156

[F|K] -57.26 0.0007156

[D|L] -57.26 0.0007156

[D|K] -57.26 0.0007156

[BEL|L] -57.26 0.0007134

[BCK|K] -57.26 0.0007134

[BCL|L] -57.26 0.0007134

[BEK|K] -57.26 0.0007134

[JL|L] -57.26 0.0007121

[JK|K] -57.26 0.0007121

[ABG|A] -57.26 0.0007105

[BFH|H] -57.26 0.0007103

[BDH|H] -57.26 0.0007103

[C|CD] -57.26 0.0007096

[C|CF] -57.26 0.0007096

[C|CE] -57.26 0.0007096

[C|CG] -57.26 0.0007096

[E|DE] -57.26 0.0007096

[E|EF] -57.26 0.0007096

[E|CE] -57.26 0.0007096

[E|EG] -57.26 0.0007096

[GK|B] -57.26 0.000709

[GL|B] -57.26 0.000709

[C|HI] -57.27 0.0007054

[E|HI] -57.27 0.0007054

[FL|L] -57.28 0.0007006

[DL|L] -57.28 0.0007006

[FK|K] -57.28 0.0007006

[DK|K] -57.28 0.0007006

[B|GH] -57.28 0.0006997

[B|CH] -57.28 0.0006997

[B|DH] -57.28 0.0006997

[B|EH] -57.28 0.0006997

[B|FH] -57.28 0.0006997

[DG|B] -57.28 0.0006975

[FG|B] -57.28 0.0006975

[B|DK] -57.28 0.0006969

[B|FL] -57.28 0.0006969

[B|DL] -57.28 0.0006969

[B|GL] -57.28 0.0006969

[B|GK] -57.28 0.0006969

[B|FK] -57.28 0.0006969

[B|CL] -57.28 0.0006969

[B|EL] -57.28 0.0006969

[B|CK] -57.28 0.0006969

[B|EK] -57.28 0.0006969

[BDJ|J] -57.29 0.0006947

[BFJ|J] -57.29 0.0006947

[J|HI] -57.29 0.0006921

[DJ|J] -57.29 0.0006911

[FJ|J] -57.29 0.0006911

[BHK|B] -57.29 0.0006891

[BHL|B] -57.29 0.0006891

[G|FG] -57.3 0.000687

[G|DG] -57.3 0.000687

[G|CG] -57.3 0.000687

[G|EG] -57.3 0.000687

[ABE|E] -57.3 0.0006864

[ABC|C] -57.3 0.0006864

[FL|B] -57.3 0.0006835

[FK|B] -57.3 0.0006835

[DK|B] -57.3 0.0006835

[DL|B] -57.3 0.0006835

[G|HI] -57.3 0.000683

[ABF|A] -57.31 0.0006799

[ABD|A] -57.31 0.0006799

[CE|H] -57.31 0.0006785

[DF|B] -57.31 0.0006778

[BC|A] -57.31 0.0006749

[BE|A] -57.31 0.0006749

[BGL|L] -57.31 0.0006748

[BGK|K] -57.31 0.0006748

[EG|H] -57.33 0.0006666

[CG|H] -57.33 0.0006666

[C|F] -57.33 0.0006628

[E|F] -57.33 0.0006628

[C|D] -57.33 0.0006628

[C|G] -57.33 0.0006628

[E|C] -57.33 0.0006628

[C|E] -57.33 0.0006628

[E|D] -57.33 0.0006628

[E|G] -57.33 0.0006628

[F|DF] -57.33 0.0006616

[F|EF] -57.33 0.0006616

[F|FG] -57.33 0.0006616

[F|CF] -57.33 0.0006616

[D|DF] -57.33 0.0006616

[D|CD] -57.33 0.0006616

[D|DE] -57.33 0.0006616

[D|DG] -57.33 0.0006616

[ABG|G] -57.33 0.0006612

[F|HI] -57.34 0.0006577

[D|HI] -57.34 0.0006577

[H|HIL] -57.35 0.0006519

[H|HIK] -57.35 0.0006519

[BG|A] -57.35 0.0006495

[J|F] -57.35 0.0006493

[J|C] -57.35 0.0006493

[J|D] -57.35 0.0006493

[J|G] -57.35 0.0006493

[J|E] -57.35 0.0006493

[CK|C] -57.35 0.0006481

[CL|C] -57.35 0.0006481

[EL|E] -57.35 0.0006481

[EK|E] -57.35 0.0006481

[CF|H] -57.35 0.0006478

[EF|H] -57.35 0.0006478

[CD|H] -57.35 0.0006478

[DE|H] -57.35 0.0006478

[BHI|B] -57.36 0.0006453

[CH|C] -57.36 0.0006447

[EH|E] -57.36 0.0006447

[C|CJ] -57.36 0.0006431

[E|EJ] -57.36 0.0006431

[G|F] -57.36 0.0006417

[G|C] -57.36 0.0006417

[G|D] -57.36 0.0006417

[G|E] -57.36 0.0006417

[L|KL] -57.37 0.000639

[K|KL] -57.37 0.000639

[C|CHL] -57.37 0.0006382

[C|CHK] -57.37 0.0006382

[E|EHL] -57.37 0.0006382

[E|EHK] -57.37 0.0006382

[ABF|F] -57.38 0.0006327

[ABD|D] -57.38 0.0006327

[BFL|L] -57.38 0.0006309

[BDK|K] -57.38 0.0006309

[BFK|K] -57.38 0.0006309

[BDL|L] -57.38 0.0006309

[J|FJ] -57.38 0.0006294

[J|DJ] -57.38 0.0006294

[J|CJ] -57.38 0.0006294

[J|GJ] -57.38 0.0006294

[J|EJ] -57.38 0.0006294

[DG|H] -57.38 0.0006291

[FG|H] -57.38 0.0006291

[GK|G] -57.39 0.0006281

[GL|G] -57.39 0.0006281

[GH|G] -57.39 0.0006276

[L|HIL] -57.39 0.0006251

[K|HIK] -57.39 0.0006251

[CHJ|H] -57.39 0.000625

[EHJ|H] -57.39 0.000625

[C|CHI] -57.39 0.0006238

[E|EHI] -57.39 0.0006238

[G|GJ] -57.39 0.0006226

[BF|A] -57.4 0.0006206

[BD|A] -57.4 0.0006206

[G|GHL] -57.4 0.0006179

[G|GHK] -57.4 0.0006179

[D|F] -57.4 0.0006179

[F|C] -57.4 0.0006179

[F|D] -57.4 0.0006179

[F|G] -57.4 0.0006179

[D|C] -57.4 0.0006179

[F|E] -57.4 0.0006179

[D|G] -57.4 0.0006179

[D|E] -57.4 0.0006179

[BCK|C] -57.4 0.0006166

[BCL|C] -57.4 0.0006166

[BEL|E] -57.4 0.0006166

[BEK|E] -57.4 0.0006166

[J|HIJ] -57.41 0.0006139

[C|BF] -57.41 0.0006137

[C|BD] -57.41 0.0006137

[C|BE] -57.41 0.0006137

[E|BF] -57.41 0.0006137

[E|BC] -57.41 0.0006137

[C|BG] -57.41 0.0006137

[E|BD] -57.41 0.0006137

[E|BG] -57.41 0.0006137

[DF|H] -57.41 0.0006114

[HI|H] -57.42 0.0006099

[FH|F] -57.42 0.0006083

[DH|D] -57.42 0.0006083

[GHJ|H] -57.42 0.0006056

[FL|F] -57.42 0.0006056

[FK|F] -57.42 0.0006056

[DK|D] -57.42 0.0006056

[DL|D] -57.42 0.0006056

[H|BCH] -57.42 0.0006047

[H|BEH] -57.42 0.0006047

[H|BDH] -57.42 0.0006047

[H|BGH] -57.42 0.0006047

[H|BFH] -57.42 0.0006047

[BCE|C] -57.42 0.000604

[BCE|E] -57.42 0.000604

[G|GHI] -57.43 0.000604

[F|FJ] -57.43 0.0005995

[D|DJ] -57.43 0.0005995

[J|HJL] -57.44 0.0005971

[J|HJK] -57.44 0.0005971

[CH|B] -57.44 0.000597

[EH|B] -57.44 0.000597

[F|FHL] -57.44 0.000595

[F|FHK] -57.44 0.000595

[D|DHK] -57.44 0.000595

[D|DHL] -57.44 0.000595

[G|BF] -57.44 0.0005942

[G|BC] -57.44 0.0005942

[G|BD] -57.44 0.0005942

[G|BE] -57.44 0.0005942

[BEG|G] -57.45 0.0005919

[BCG|C] -57.45 0.0005919

[BEG|E] -57.45 0.0005919

[BCG|G] -57.45 0.0005919

[BHK|H] -57.45 0.0005875

[BHL|H] -57.45 0.0005875

[FHJ|H] -57.46 0.0005836

[DHJ|H] -57.46 0.0005836

[BGL|G] -57.46 0.0005832

[BGK|G] -57.46 0.0005832

[H|HKL] -57.46 0.0005819

[F|FHI] -57.46 0.0005816

[D|DHI] -57.46 0.0005816

[GH|B] -57.46 0.0005812

[K|DK] -57.47 0.0005763

[L|DL] -57.47 0.0005763

[L|FL] -57.47 0.0005763

[L|GL] -57.47 0.0005763

[L|CL] -57.47 0.0005763

[K|GK] -57.47 0.0005763

[K|FK] -57.47 0.0005763

[L|EL] -57.47 0.0005763

[K|CK] -57.47 0.0005763

[K|EK] -57.47 0.0005763

[F|BC] -57.48 0.0005721

[F|BE] -57.48 0.0005721

[F|BD] -57.48 0.0005721

[D|BF] -57.48 0.0005721

[D|BC] -57.48 0.0005721

[F|BG] -57.48 0.0005721

[D|BE] -57.48 0.0005721

[D|BG] -57.48 0.0005721

[BH|L] -57.48 0.0005719

[BH|K] -57.48 0.0005719

[BCF|F] -57.48 0.000571

[BEF|F] -57.48 0.000571

[BCF|C] -57.48 0.000571

[BCD|C] -57.48 0.000571

[BCD|D] -57.48 0.000571

[BEF|E] -57.48 0.000571

[BDE|D] -57.48 0.000571

[BDE|E] -57.48 0.000571

[BC|F] -57.49 0.0005684

[BE|F] -57.49 0.0005684

[BC|D] -57.49 0.0005684

[BE|C] -57.49 0.0005684

[BE|D] -57.49 0.0005684

[BC|G] -57.49 0.0005684

[BC|E] -57.49 0.0005684

[BE|G] -57.49 0.0005684

[AB|F] -57.49 0.0005656

[AB|C] -57.49 0.0005656

[AB|D] -57.49 0.0005656

[AB|G] -57.49 0.0005656

[AB|E] -57.49 0.0005656

[FH|B] -57.49 0.0005633

[DH|B] -57.49 0.0005633

[A|BH] -57.5 0.0005625

[CEH|H] -57.5 0.0005609

[BC|J] -57.5 0.0005589

[BE|J] -57.5 0.0005589

[L|HKL] -57.5 0.000558

[K|HKL] -57.5 0.000558

[H|L] -57.51 0.0005557

[H|K] -57.51 0.0005557

[HL|L] -57.51 0.0005537

[HK|K] -57.51 0.0005537

[EGH|H] -57.52 0.0005512

[CGH|H] -57.52 0.0005512

[HL|B] -57.52 0.0005508

[HK|B] -57.52 0.0005508

[BHI|H] -57.52 0.0005502

[BFG|F] -57.52 0.0005498

[BFG|G] -57.52 0.0005498

[BDG|D] -57.52 0.0005498

[BDG|G] -57.52 0.0005498

[C|BCJ] -57.52 0.0005487

[E|BEJ] -57.52 0.0005487

[BG|F] -57.52 0.000547

[BG|C] -57.52 0.000547

[BG|D] -57.52 0.000547

[BG|E] -57.52 0.000547

[BFL|F] -57.53 0.0005453

[BFK|F] -57.53 0.0005453

[BDK|D] -57.53 0.0005453

[BDL|D] -57.53 0.0005453

[CK|H] -57.53 0.0005441

[CL|H] -57.53 0.0005441

[EL|H] -57.53 0.0005441

[EK|H] -57.53 0.0005441

[ABH|A] -57.53 0.0005427

[BG|J] -57.54 0.0005379

[EJ|L] -57.54 0.0005376

[CJ|L] -57.54 0.0005376

[EJ|K] -57.54 0.0005376

[CJ|K] -57.54 0.0005376

[C|BCD] -57.54 0.0005375

[C|BCE] -57.54 0.0005375

[E|BEF] -57.54 0.0005375

[C|BCF] -57.54 0.0005375

[C|BCG] -57.54 0.0005375

[E|BCE] -57.54 0.0005375

[E|BDE] -57.54 0.0005375

[E|BEG] -57.54 0.0005375

[HJ|J] -57.54 0.0005371

[J|BFJ] -57.54 0.000537

[J|BEJ] -57.54 0.000537

[J|BCJ] -57.54 0.000537

[J|BGJ] -57.54 0.000537

[J|BDJ] -57.54 0.000537

[C|J] -57.54 0.0005368

[E|J] -57.54 0.0005368

[CDH|H] -57.55 0.0005351

[EFH|H] -57.55 0.0005351

[CFH|H] -57.55 0.0005351

[DEH|H] -57.55 0.0005351

[C|HJ] -57.55 0.000532

[E|HJ] -57.55 0.000532

[G|BGJ] -57.55 0.0005313

[C|GH] -57.55 0.0005305

[E|GH] -57.55 0.0005305

[C|DH] -57.55 0.0005305

[C|EH] -57.55 0.0005305

[C|FH] -57.55 0.0005305

[E|CH] -57.55 0.0005305

[E|DH] -57.55 0.0005305

[E|FH] -57.55 0.0005305

[BDF|F] -57.56 0.0005303

[BDF|D] -57.56 0.0005303

[GK|H] -57.56 0.0005273

[GL|H] -57.56 0.0005273

[BD|F] -57.57 0.0005227

[BF|C] -57.57 0.0005227

[BF|D] -57.57 0.0005227

[BD|C] -57.57 0.0005227

[BF|G] -57.57 0.0005227

[BF|E] -57.57 0.0005227

[BD|G] -57.57 0.0005227

[BD|E] -57.57 0.0005227

[KL|L] -57.57 0.0005218

[KL|K] -57.57 0.0005218

[G|BFG] -57.57 0.0005204

[G|BCG] -57.57 0.0005204

[G|BDG] -57.57 0.0005204

[G|BEG] -57.57 0.0005204

[J|GH] -57.57 0.0005203

[J|CH] -57.57 0.0005203

[J|DH] -57.57 0.0005203

[J|EH] -57.57 0.0005203

[J|FH] -57.57 0.0005203

[G|J] -57.58 0.0005198

[GJ|L] -57.58 0.0005191

[GJ|K] -57.58 0.0005191

[FGH|H] -57.58 0.000519

[DGH|H] -57.58 0.000519

[JK|H] -57.58 0.0005188

[JL|H] -57.58 0.0005188

[B|FJ] -57.58 0.0005157

[B|DJ] -57.58 0.0005157

[B|CJ] -57.58 0.0005157

[B|GJ] -57.58 0.0005157

[B|EJ] -57.58 0.0005157

[G|HJ] -57.58 0.0005151

[BF|J] -57.59 0.0005139

[BD|J] -57.59 0.0005139

[G|CH] -57.59 0.0005136

[G|DH] -57.59 0.0005136

[G|FH] -57.59 0.0005136

[G|EH] -57.59 0.0005136

[K|HL] -57.59 0.0005123

[L|HK] -57.59 0.0005123

[F|BFJ] -57.59 0.0005116

[D|BDJ] -57.59 0.0005116

[K|BCK] -57.59 0.0005101

[K|BDK] -57.59 0.0005101

[L|BCL] -57.59 0.0005101

[L|BFL] -57.59 0.0005101

[K|BGK] -57.59 0.0005101

[K|BFK] -57.59 0.0005101

[L|BGL] -57.59 0.0005101

[L|BDL] -57.59 0.0005101

[K|BEK] -57.59 0.0005101

[L|BEL] -57.59 0.0005101

[K|L] -57.6 0.0005085

[L|K] -57.6 0.0005085

[FL|H] -57.6 0.0005084

[FK|H] -57.6 0.0005084

[DK|H] -57.6 0.0005084

[DL|H] -57.6 0.0005084

[DFH|H] -57.61 0.0005039

[F|BEF] -57.61 0.0005011

[F|BDF] -57.61 0.0005011

[D|BCD] -57.61 0.0005011

[F|BCF] -57.61 0.0005011

[F|BFG] -57.61 0.0005011

[D|BDE] -57.61 0.0005011

[D|BDF] -57.61 0.0005011

[D|BDG] -57.61 0.0005011

[F|J] -57.61 0.0005005

[D|J] -57.61 0.0005005

[DJ|L] -57.62 0.0004981

[FJ|L] -57.62 0.0004981

[DJ|K] -57.62 0.0004981

[FJ|K] -57.62 0.0004981

[JK|J] -57.62 0.000498

[JL|J] -57.62 0.000498

[F|HJ] -57.62 0.000496

[D|HJ] -57.62 0.000496

[F|GH] -57.62 0.0004946

[F|CH] -57.62 0.0004946

[D|GH] -57.62 0.0004946

[F|DH] -57.62 0.0004946

[F|EH] -57.62 0.0004946

[D|CH] -57.62 0.0004946

[D|EH] -57.62 0.0004946

[D|FH] -57.62 0.0004946

[L|BF] -57.63 0.0004945

[K|BF] -57.63 0.0004945

[L|BC] -57.63 0.0004945

[K|BC] -57.63 0.0004945

[K|BD] -57.63 0.0004945

[L|BE] -57.63 0.0004945

[K|BE] -57.63 0.0004945

[L|BD] -57.63 0.0004945

[K|BG] -57.63 0.0004945

[L|BG] -57.63 0.0004945

[L|JL] -57.64 0.0004893

[K|JK] -57.64 0.0004893

[CEJ|C] -57.64 0.000489

[CEJ|E] -57.64 0.000489

[C|KL] -57.64 0.0004862

[E|KL] -57.64 0.0004862

[A|ABC] -57.64 0.000485

[A|ABD] -57.64 0.000485

[A|ABF] -57.64 0.000485

[A|ABG] -57.64 0.000485

[A|ABE] -57.64 0.000485

[EGJ|G] -57.66 0.0004799

[CGJ|C] -57.66 0.0004799

[EGJ|E] -57.66 0.0004799

[CGJ|G] -57.66 0.0004799

[H|F] -57.66 0.0004782

[H|C] -57.66 0.0004782

[H|D] -57.66 0.0004782

[H|G] -57.66 0.0004782

[H|E] -57.66 0.0004782

[J|KL] -57.66 0.0004772

[A|B] -57.66 0.0004759

[BCH|C] -57.67 0.0004751

[BEH|E] -57.67 0.0004751

[CE|L] -57.67 0.0004714

[CE|K] -57.67 0.0004714

[G|KL] -57.67 0.0004707

[BH|A] -57.67 0.0004705

[K|HI] -57.68 0.000469

[L|HI] -57.68 0.000469

[B|DE] -57.68 0.0004687

[B|CD] -57.68 0.0004687

[B|DF] -57.68 0.0004687

[B|EF] -57.68 0.0004687

[B|CE] -57.68 0.0004687

[B|CF] -57.68 0.0004687

[B|FG] -57.68 0.0004687

[B|DG] -57.68 0.0004687

[B|CG] -57.68 0.0004687

[B|EG] -57.68 0.0004687

[CFJ|F] -57.69 0.0004642

[DEJ|D] -57.69 0.0004642

[CDJ|C] -57.69 0.0004642

[CFJ|C] -57.69 0.0004642

[CDJ|D] -57.69 0.0004642

[DEJ|E] -57.69 0.0004642

[EFJ|F] -57.69 0.0004642

[EFJ|E] -57.69 0.0004642

[EJ|F] -57.69 0.0004638

[CJ|F] -57.69 0.0004638

[EJ|C] -57.69 0.0004638

[EJ|D] -57.69 0.0004638

[EJ|G] -57.69 0.0004638

[CJ|D] -57.69 0.0004638

[CJ|G] -57.69 0.0004638

[CJ|E] -57.69 0.0004638

[EG|L] -57.69 0.0004631

[CG|L] -57.69 0.0004631

[EG|K] -57.69 0.0004631

[CG|K] -57.69 0.0004631

[C|CKL] -57.69 0.0004628

[E|EKL] -57.69 0.0004628

[BGH|G] -57.7 0.0004591

[C|CDH] -57.71 0.0004553

[C|CGH] -57.71 0.0004553

[C|CFH] -57.71 0.0004553

[E|DEH] -57.71 0.0004553

[E|EGH] -57.71 0.0004553

[C|CEH] -57.71 0.0004553

[E|EFH] -57.71 0.0004553

[E|CEH] -57.71 0.0004553

[HIJ|H] -57.71 0.0004547

[F|KL] -57.71 0.0004533

[D|KL] -57.71 0.0004533

[CF|L] -57.72 0.0004501

[EF|L] -57.72 0.0004501

[CD|L] -57.72 0.0004501

[DE|L] -57.72 0.0004501

[CF|K] -57.72 0.0004501

[EF|K] -57.72 0.0004501

[CD|K] -57.72 0.0004501

[DE|K] -57.72 0.0004501

[FGJ|F] -57.72 0.0004483

[DGJ|D] -57.72 0.0004483

[DGJ|G] -57.72 0.0004483

[FGJ|G] -57.72 0.0004483

[G|GKL] -57.72 0.0004481

[GJ|F] -57.72 0.0004478

[GJ|C] -57.72 0.0004478

[GJ|D] -57.72 0.0004478

[GJ|E] -57.72 0.0004478

[C|CHJ] -57.73 0.0004443

[E|EHJ] -57.73 0.0004443

[H|GHL] -57.74 0.0004414

[H|GHK] -57.74 0.0004414

[H|FHL] -57.74 0.0004414

[H|FHK] -57.74 0.0004414

[H|CHL] -57.74 0.0004414

[H|DHK] -57.74 0.0004414

[H|DHL] -57.74 0.0004414

[H|CHK] -57.74 0.0004414

[H|EHL] -57.74 0.0004414

[H|EHK] -57.74 0.0004414

[H|BF] -57.74 0.000441

[H|BC] -57.74 0.000441

[H|BE] -57.74 0.000441

[H|BD] -57.74 0.000441

[H|BG] -57.74 0.000441

[BFH|F] -57.74 0.0004409

[BDH|D] -57.74 0.0004409

[G|CGH] -57.74 0.0004409

[G|DGH] -57.74 0.0004409

[G|FGH] -57.74 0.0004409

[G|EGH] -57.74 0.0004409

[K|F] -57.74 0.0004399

[L|F] -57.74 0.0004399

[L|C] -57.74 0.0004399

[K|C] -57.74 0.0004399

[K|D] -57.74 0.0004399

[L|D] -57.74 0.0004399

[L|G] -57.74 0.0004399

[K|G] -57.74 0.0004399

[K|E] -57.74 0.0004399

[L|E] -57.74 0.0004399

[EHL|H] -57.74 0.0004392

[EHK|H] -57.74 0.0004392

[CHK|H] -57.74 0.0004392

[CHL|H] -57.74 0.0004392

[HJL|H] -57.74 0.0004389

[HJK|H] -57.74 0.0004389

[C|DK] -57.75 0.0004372

[C|FL] -57.75 0.0004372

[C|DL] -57.75 0.0004372

[C|GL] -57.75 0.0004372

[C|GK] -57.75 0.0004372

[C|FK] -57.75 0.0004372

[C|EL] -57.75 0.0004372

[E|DK] -57.75 0.0004372

[E|DL] -57.75 0.0004372

[E|FL] -57.75 0.0004372

[C|EK] -57.75 0.0004372

[E|GL] -57.75 0.0004372

[E|CL] -57.75 0.0004372

[E|GK] -57.75 0.0004372

[E|FK] -57.75 0.0004372

[E|CK] -57.75 0.0004372

[DG|L] -57.75 0.0004371

[FG|L] -57.75 0.0004371

[DG|K] -57.75 0.0004371

[FG|K] -57.75 0.0004371

[HI|B] -57.75 0.0004365

[J|GHJ] -57.75 0.0004349

[J|CHJ] -57.75 0.0004349

[J|DHJ] -57.75 0.0004349

[J|EHJ] -57.75 0.0004349

[J|FHJ] -57.75 0.0004349

[H|HIJ] -57.76 0.0004338

[DFJ|F] -57.76 0.0004336

[DFJ|D] -57.76 0.0004336

[CHI|H] -57.76 0.000432

[EHI|H] -57.76 0.000432

[D|DKL] -57.76 0.0004315

[F|FKL] -57.76 0.0004315

[H|GHI] -57.76 0.0004314

[H|FHI] -57.76 0.0004314

[H|DHI] -57.76 0.0004314

[H|EHI] -57.76 0.0004314

[H|CHI] -57.76 0.0004314

[G|GHJ] -57.76 0.0004302

[DJ|F] -57.77 0.0004297

[DJ|C] -57.77 0.0004297

[FJ|C] -57.77 0.0004297

[FJ|D] -57.77 0.0004297

[DJ|G] -57.77 0.0004297

[DJ|E] -57.77 0.0004297

[FJ|G] -57.77 0.0004297

[FJ|E] -57.77 0.0004297

[J|DK] -57.77 0.0004287

[J|DL] -57.77 0.0004287

[J|FL] -57.77 0.0004287

[J|GL] -57.77 0.0004287

[J|GK] -57.77 0.0004287

[J|CL] -57.77 0.0004287

[J|FK] -57.77 0.0004287

[J|EL] -57.77 0.0004287

[J|CK] -57.77 0.0004287

[J|EK] -57.77 0.0004287

[GHK|H] -57.77 0.0004265

[GHL|H] -57.77 0.0004265

[DF|L] -57.78 0.0004248

[DF|K] -57.78 0.0004248

[EJL|L] -57.78 0.0004246

[CJL|L] -57.78 0.0004246

[EJK|K] -57.78 0.0004246

[CJK|K] -57.78 0.0004246

[F|DFH] -57.78 0.0004245

[F|FGH] -57.78 0.0004245

[F|CFH] -57.78 0.0004245

[D|CDH] -57.78 0.0004245

[D|DFH] -57.78 0.0004245

[D|DGH] -57.78 0.0004245

[D|DEH] -57.78 0.0004245

[F|EFH] -57.78 0.0004245

[G|DK] -57.78 0.0004233

[G|DL] -57.78 0.0004233

[G|FL] -57.78 0.0004233

[G|CL] -57.78 0.0004233

[G|FK] -57.78 0.0004233

[G|EL] -57.78 0.0004233

[G|CK] -57.78 0.0004233

[G|EK] -57.78 0.0004233

[L|GHL] -57.78 0.0004232

[K|GHK] -57.78 0.0004232

[L|FHL] -57.78 0.0004232

[K|FHK] -57.78 0.0004232

[L|CHL] -57.78 0.0004232

[K|DHK] -57.78 0.0004232

[L|DHL] -57.78 0.0004232

[K|CHK] -57.78 0.0004232

[L|EHL] -57.78 0.0004232

[K|EHK] -57.78 0.0004232

[BHL|L] -57.78 0.000422

[BHK|K] -57.78 0.000422

[H|HJL] -57.78 0.0004219

[H|HJK] -57.78 0.0004219

[GHI|H] -57.79 0.0004201

[BHJ|J] -57.79 0.0004185

[CEG|C] -57.8 0.0004171

[CEG|G] -57.8 0.0004171

[CEG|E] -57.8 0.0004171

[F|FHJ] -57.8 0.0004143

[D|DHJ] -57.8 0.0004143

[FHL|H] -57.81 0.0004122

[FHK|H] -57.81 0.0004122

[DHL|H] -57.81 0.0004122

[DHK|H] -57.81 0.0004122

[GJL|L] -57.81 0.0004108

[GJK|K] -57.81 0.0004108

[F|DK] -57.82 0.0004076

[F|DL] -57.82 0.0004076

[F|GL] -57.82 0.0004076

[F|GK] -57.82 0.0004076

[F|CL] -57.82 0.0004076

[F|EL] -57.82 0.0004076

[F|CK] -57.82 0.0004076

[D|FL] -57.82 0.0004076

[D|GL] -57.82 0.0004076

[F|EK] -57.82 0.0004076

[D|GK] -57.82 0.0004076

[D|FK] -57.82 0.0004076

[D|CL] -57.82 0.0004076

[D|EL] -57.82 0.0004076

[D|CK] -57.82 0.0004076

[D|EK] -57.82 0.0004076

[CE|F] -57.82 0.0004071

[CE|D] -57.82 0.0004071

[CE|G] -57.82 0.0004071

[FHI|H] -57.82 0.0004067

[DHI|H] -57.82 0.0004067

[CEF|F] -57.82 0.0004057

[CDE|C] -57.82 0.0004057

[CDE|D] -57.82 0.0004057

[CEF|C] -57.82 0.0004057

[CDE|E] -57.82 0.0004057

[CEF|E] -57.82 0.0004057

[L|HJL] -57.83 0.0004046

[K|HJK] -57.83 0.0004046

[EG|F] -57.84 0.0004

[CG|F] -57.84 0.0004

[EG|C] -57.84 0.0004

[EG|D] -57.84 0.0004

[CG|D] -57.84 0.0004

[CG|E] -57.84 0.0004

[BH|J] -57.84 0.0003999

[EFG|F] -57.84 0.0003972

[CFG|F] -57.84 0.0003972

[CFG|C] -57.84 0.0003972

[EFG|G] -57.84 0.0003972

[CFG|G] -57.84 0.0003972

[EFG|E] -57.84 0.0003972

[CDG|C] -57.84 0.0003972

[CDG|D] -57.84 0.0003972

[DEG|D] -57.84 0.0003972

[CDG|G] -57.84 0.0003972

[DEG|G] -57.84 0.0003972

[DEG|E] -57.84 0.0003972

[BH|F] -57.85 0.000396

[BH|C] -57.85 0.000396

[BH|D] -57.85 0.000396

[BH|G] -57.85 0.000396

[BH|E] -57.85 0.000396

[CEJ|J] -57.85 0.0003957

[CEL|L] -57.85 0.0003952

[CEK|K] -57.85 0.0003952

[DJL|L] -57.85 0.0003951

[DJK|K] -57.85 0.0003951

[FJL|L] -57.85 0.0003951

[FJK|K] -57.85 0.0003951

[J|JKL] -57.85 0.000393

[E|DEL] -57.86 0.0003924

[C|CFL] -57.86 0.0003924

[C|CDL] -57.86 0.0003924

[C|CDK] -57.86 0.0003924

[E|DEK] -57.86 0.0003924

[C|CGK] -57.86 0.0003924

[C|CGL] -57.86 0.0003924

[C|CEK] -57.86 0.0003924

[C|CFK] -57.86 0.0003924

[C|CEL] -57.86 0.0003924

[E|EFL] -57.86 0.0003924

[E|EGK] -57.86 0.0003924

[E|EFK] -57.86 0.0003924

[E|EGL] -57.86 0.0003924

[E|CEK] -57.86 0.0003924

[E|CEL] -57.86 0.0003924

[CD|F] -57.87 0.0003887

[DE|F] -57.87 0.0003887

[EF|C] -57.87 0.0003887

[CF|D] -57.87 0.0003887

[EF|D] -57.87 0.0003887

[CF|G] -57.87 0.0003887

[DE|C] -57.87 0.0003887

[EF|G] -57.87 0.0003887

[CD|G] -57.87 0.0003887

[CF|E] -57.87 0.0003887

[DE|G] -57.87 0.0003887

[CD|E] -57.87 0.0003887

[H|J] -57.87 0.0003887

[EGJ|J] -57.87 0.0003883

[CGJ|J] -57.87 0.0003883

[CHJ|C] -57.87 0.000388

[EHJ|E] -57.87 0.000388

[EGL|L] -57.87 0.0003878

[CGK|K] -57.87 0.0003878

[EGK|K] -57.87 0.0003878

[CGL|L] -57.87 0.0003878

[CDF|F] -57.87 0.0003864

[DEF|F] -57.87 0.0003864

[CDF|C] -57.87 0.0003864

[CDF|D] -57.87 0.0003864

[DEF|D] -57.87 0.0003864

[DEF|E] -57.87 0.0003864

[HJ|L] -57.88 0.0003834

[HJ|K] -57.88 0.0003834

[KL|H] -57.89 0.0003802

[G|DGL] -57.89 0.0003799

[G|FGL] -57.89 0.0003799

[G|DGK] -57.89 0.0003799

[G|FGK] -57.89 0.0003799

[G|EGK] -57.89 0.0003799

[G|EGL] -57.89 0.0003799

[G|CGK] -57.89 0.0003799

[G|CGL] -57.89 0.0003799

[CK|L] -57.89 0.0003779

[EK|L] -57.89 0.0003779

[CL|K] -57.89 0.0003779

[EL|K] -57.89 0.0003779

[DG|F] -57.89 0.0003775

[DG|C] -57.89 0.0003775

[FG|C] -57.89 0.0003775

[FG|D] -57.89 0.0003775

[DG|E] -57.89 0.0003775

[FG|E] -57.89 0.0003775

[DEL|L] -57.9 0.0003761

[CFL|L] -57.9 0.0003761

[CDK|K] -57.9 0.0003761

[CDL|L] -57.9 0.0003761

[DEK|K] -57.9 0.0003761

[CFK|K] -57.9 0.0003761

[EFL|L] -57.9 0.0003761

[EFK|K] -57.9 0.0003761

[GHJ|G] -57.9 0.0003759

[DEJ|J] -57.9 0.0003756

[CDJ|J] -57.9 0.0003756

[CFJ|J] -57.9 0.0003756

[EFJ|J] -57.9 0.0003756

[DFG|F] -57.9 0.0003739

[DFG|D] -57.9 0.0003739

[DFG|G] -57.9 0.0003739

[CH|L] -57.91 0.0003729

[EH|L] -57.91 0.0003729

[CH|K] -57.91 0.0003729

[EH|K] -57.91 0.0003729

[C|JL] -57.91 0.0003723

[C|JK] -57.91 0.0003723

[E|JL] -57.91 0.0003723

[E|JK] -57.91 0.0003723

[CJL|C] -57.92 0.000367

[CJK|C] -57.92 0.000367

[EJL|E] -57.92 0.000367

[EJK|E] -57.92 0.000367

[DF|C] -57.92 0.0003668

[DF|G] -57.92 0.0003668

[DF|E] -57.92 0.0003668

[GK|L] -57.93 0.0003663

[GL|K] -57.93 0.0003663

[F|DFL] -57.93 0.0003658

[D|DEL] -57.93 0.0003658

[D|DFL] -57.93 0.0003658

[F|DFK] -57.93 0.0003658

[F|CFL] -57.93 0.0003658

[F|FGL] -57.93 0.0003658

[F|EFL] -57.93 0.0003658

[F|FGK] -57.93 0.0003658

[D|DFK] -57.93 0.0003658

[F|EFK] -57.93 0.0003658

[D|DEK] -57.93 0.0003658

[D|DGL] -57.93 0.0003658

[F|CFK] -57.93 0.0003658

[D|DGK] -57.93 0.0003658

[D|CDL] -57.93 0.0003658

[D|CDK] -57.93 0.0003658

[DGL|L] -57.93 0.0003644

[DGK|K] -57.93 0.0003644

[FGK|K] -57.93 0.0003644

[FGL|L] -57.93 0.0003644

[GH|L] -57.93 0.000363

[GH|K] -57.93 0.000363

[DGJ|J] -57.93 0.0003627

[FGJ|J] -57.93 0.0003627

[FHJ|F] -57.94 0.0003623

[DHJ|D] -57.94 0.0003623

[G|JL] -57.94 0.0003605

[G|JK] -57.94 0.0003605

[JK|L] -57.94 0.0003603

[JL|K] -57.94 0.0003603

[C|DF] -57.95 0.000357

[C|DE] -57.95 0.000357

[C|EF] -57.95 0.000357

[C|FG] -57.95 0.000357

[C|DG] -57.95 0.000357

[C|EG] -57.95 0.000357

[E|DF] -57.95 0.000357

[E|CD] -57.95 0.000357

[E|FG] -57.95 0.000357

[E|CF] -57.95 0.000357

[E|CG] -57.95 0.000357

[E|DG] -57.95 0.000357

[L|J] -57.95 0.0003556

[K|J] -57.95 0.0003556

[GJK|G] -57.96 0.0003551

[GJL|G] -57.96 0.0003551

[K|HJ] -57.96 0.0003539

[L|HJ] -57.96 0.0003539

[DFL|L] -57.96 0.0003534

[DFK|K] -57.96 0.0003534

[FK|L] -57.96 0.0003531

[DK|L] -57.96 0.0003531

[FL|K] -57.96 0.0003531

[DL|K] -57.96 0.0003531

[K|GH] -57.96 0.0003526

[L|GH] -57.96 0.0003526

[L|CH] -57.96 0.0003526

[K|CH] -57.96 0.0003526

[K|DH] -57.96 0.0003526

[L|DH] -57.96 0.0003526

[K|EH] -57.96 0.0003526

[K|FH] -57.96 0.0003526

[L|EH] -57.96 0.0003526

[L|FH] -57.96 0.0003526

[FH|L] -57.97 0.0003518

[FH|K] -57.97 0.0003518

[DH|L] -57.97 0.0003518

[DH|K] -57.97 0.0003518

[DFJ|J] -57.97 0.0003508

[C|CJL] -57.97 0.0003508

[C|CJK] -57.97 0.0003508

[E|EJL] -57.97 0.0003508

[E|EJK] -57.97 0.0003508

[J|CD] -57.97 0.0003497

[J|DF] -57.97 0.0003497

[J|DE] -57.97 0.0003497

[J|EF] -57.97 0.0003497

[J|FG] -57.97 0.0003497

[J|CF] -57.97 0.0003497

[J|CE] -57.97 0.0003497

[J|CG] -57.97 0.0003497

[J|DG] -57.97 0.0003497

[J|EG] -57.97 0.0003497

[H|KL] -57.97 0.0003487

[CEH|C] -57.98 0.0003482

[CEH|E] -57.98 0.0003482

[F|JL] -57.98 0.0003471

[F|JK] -57.98 0.0003471

[D|JL] -57.98 0.0003471

[D|JK] -57.98 0.0003471

[G|DE] -57.98 0.0003457

[G|CD] -57.98 0.0003457

[G|DF] -57.98 0.0003457

[G|EF] -57.98 0.0003457

[G|CE] -57.98 0.0003457

[G|CF] -57.98 0.0003457

[J|DJL] -57.99 0.0003433

[J|DJK] -57.99 0.0003433

[J|GJL] -57.99 0.0003433

[J|GJK] -57.99 0.0003433

[J|CJL] -57.99 0.0003433

[J|EJL] -57.99 0.0003433

[J|FJL] -57.99 0.0003433

[J|FJK] -57.99 0.0003433

[J|EJK] -57.99 0.0003433

[J|CJK] -57.99 0.0003433

[CGH|C] -57.99 0.0003421

[EGH|G] -57.99 0.0003421

[CGH|G] -57.99 0.0003421

[EGH|E] -57.99 0.0003421

[CEL|C] -57.99 0.0003416

[CEK|C] -57.99 0.0003416

[CEK|E] -57.99 0.0003416

[CEL|E] -57.99 0.0003416

[FJL|F] -58 0.0003415

[FJK|F] -58 0.0003415

[DJK|D] -58 0.0003415

[DJL|D] -58 0.0003415

[G|GJL] -58 0.0003396

[G|GJK] -58 0.0003396

[CGK|C] -58.01 0.0003352

[CGK|G] -58.01 0.0003352

[EGK|G] -58.01 0.0003352

[EGK|E] -58.01 0.0003352

[CGL|C] -58.01 0.0003352

[EGL|G] -58.01 0.0003352

[EGL|E] -58.01 0.0003352

[CGL|G] -58.01 0.0003352

[F|CD] -58.02 0.0003329

[F|DE] -58.02 0.0003329

[F|CE] -58.02 0.0003329

[F|CG] -58.02 0.0003329

[F|DG] -58.02 0.0003329

[F|EG] -58.02 0.0003329

[D|EF] -58.02 0.0003329

[D|FG] -58.02 0.0003329

[D|CG] -58.02 0.0003329

[D|CF] -58.02 0.0003329

[D|CE] -58.02 0.0003329

[D|EG] -58.02 0.0003329

[EFH|F] -58.02 0.0003322

[CFH|F] -58.02 0.0003322

[CDH|C] -58.02 0.0003322

[CDH|D] -58.02 0.0003322

[CFH|C] -58.02 0.0003322

[DEH|D] -58.02 0.0003322

[EFH|E] -58.02 0.0003322

[DEH|E] -58.02 0.0003322

[HJ|F] -58.03 0.0003304

[HJ|C] -58.03 0.0003304

[HJ|D] -58.03 0.0003304

[HJ|G] -58.03 0.0003304

[HJ|E] -58.03 0.0003304

[CE|J] -58.03 0.0003297

[D|DJL] -58.04 0.000327

[D|DJK] -58.04 0.000327

[F|FJL] -58.04 0.000327

[F|FJK] -58.04 0.000327

[CK|F] -58.04 0.0003261

[EL|F] -58.04 0.0003261

[CL|F] -58.04 0.0003261

[EK|F] -58.04 0.0003261

[EL|C] -58.04 0.0003261

[CK|D] -58.04 0.0003261

[CL|D] -58.04 0.0003261

[EL|D] -58.04 0.0003261

[CK|G] -58.04 0.0003261

[EK|C] -58.04 0.0003261

[CL|G] -58.04 0.0003261

[CK|E] -58.04 0.0003261

[EL|G] -58.04 0.0003261

[EK|D] -58.04 0.0003261

[CL|E] -58.04 0.0003261

[EK|G] -58.04 0.0003261

[CFL|F] -58.04 0.0003251

[CFK|F] -58.04 0.0003251

[CFL|C] -58.04 0.0003251

[DEL|D] -58.04 0.0003251

[CDK|C] -58.04 0.0003251

[CDK|D] -58.04 0.0003251

[CFK|C] -58.04 0.0003251

[EFL|F] -58.04 0.0003251

[DEK|D] -58.04 0.0003251

[DEL|E] -58.04 0.0003251

[CDL|C] -58.04 0.0003251

[CDL|D] -58.04 0.0003251

[EFK|F] -58.04 0.0003251

[DEK|E] -58.04 0.0003251

[EFL|E] -58.04 0.0003251

[EFK|E] -58.04 0.0003251

[EG|J] -58.05 0.0003239

[CG|J] -58.05 0.0003239

[C|FJ] -58.05 0.0003235

[C|DJ] -58.05 0.0003235

[C|GJ] -58.05 0.0003235

[C|EJ] -58.05 0.0003235

[E|FJ] -58.05 0.0003235

[E|DJ] -58.05 0.0003235

[E|CJ] -58.05 0.0003235

[E|GJ] -58.05 0.0003235

[FGH|F] -58.05 0.0003221

[FGH|G] -58.05 0.0003221

[DGH|D] -58.05 0.0003221

[DGH|G] -58.05 0.0003221

[CH|F] -58.06 0.0003216

[EH|F] -58.06 0.0003216

[CH|D] -58.06 0.0003216

[EH|C] -58.06 0.0003216

[CH|G] -58.06 0.0003216

[EH|D] -58.06 0.0003216

[CH|E] -58.06 0.0003216

[EH|G] -58.06 0.0003216

[HIK|H] -58.06 0.0003209

[HIL|H] -58.06 0.0003209

[HKL|H] -58.07 0.0003171

[GK|F] -58.07 0.000316

[GL|F] -58.07 0.000316

[GK|C] -58.07 0.000316

[GK|D] -58.07 0.000316

[GL|C] -58.07 0.000316

[GL|D] -58.07 0.000316

[GK|E] -58.07 0.000316

[GL|E] -58.07 0.000316

[EHL|L] -58.07 0.0003154

[EHK|K] -58.07 0.0003154

[CHL|L] -58.07 0.0003154

[CHK|K] -58.07 0.0003154

[HJL|L] -58.08 0.0003152

[HJK|K] -58.08 0.0003152

[FGK|F] -58.08 0.000315

[FGL|F] -58.08 0.000315

[DGK|D] -58.08 0.000315

[DGK|G] -58.08 0.000315

[DGL|D] -58.08 0.000315

[FGK|G] -58.08 0.000315

[DGL|G] -58.08 0.000315

[FGL|G] -58.08 0.000315

[H|CDH] -58.08 0.0003149

[H|DFH] -58.08 0.0003149

[H|CGH] -58.08 0.0003149

[H|DGH] -58.08 0.0003149

[H|FGH] -58.08 0.0003149

[H|DEH] -58.08 0.0003149

[H|CFH] -58.08 0.0003149

[H|EGH] -58.08 0.0003149

[H|EFH] -58.08 0.0003149

[H|CEH] -58.08 0.0003149

[CF|J] -58.08 0.0003148

[EF|J] -58.08 0.0003148

[CD|J] -58.08 0.0003148

[DE|J] -58.08 0.0003148

[CHJ|J] -58.08 0.0003139

[EHJ|J] -58.08 0.0003139

[G|FJ] -58.08 0.0003132

[G|DJ] -58.08 0.0003132

[G|CJ] -58.08 0.0003132

[G|EJ] -58.08 0.0003132

[H|DK] -58.08 0.0003132

[H|DL] -58.08 0.0003132

[H|FL] -58.08 0.0003132

[H|GL] -58.08 0.0003132

[H|CL] -58.08 0.0003132

[H|GK] -58.08 0.0003132

[H|FK] -58.08 0.0003132

[H|EL] -58.08 0.0003132

[H|CK] -58.08 0.0003132

[H|EK] -58.08 0.0003132

[GH|F] -58.08 0.0003131

[GH|C] -58.08 0.0003131

[GH|D] -58.08 0.0003131

[GH|E] -58.08 0.0003131

[DFH|F] -58.08 0.0003128

[DFH|D] -58.08 0.0003128

[C|CDE] -58.09 0.0003115

[E|DEF] -58.09 0.0003115

[C|CDG] -58.09 0.0003115

[E|DEG] -58.09 0.0003115

[E|CDE] -58.09 0.0003115

[C|CDF] -58.09 0.0003115

[C|CFG] -58.09 0.0003115

[C|CEF] -58.09 0.0003115

[E|EFG] -58.09 0.0003115

[C|CEG] -58.09 0.0003115

[E|CEF] -58.09 0.0003115

[E|CEG] -58.09 0.0003115

[JK|F] -58.09 0.0003105

[JL|F] -58.09 0.0003105

[JK|C] -58.09 0.0003105

[JK|D] -58.09 0.0003105

[JL|C] -58.09 0.0003105

[JL|D] -58.09 0.0003105

[JK|G] -58.09 0.0003105

[JL|G] -58.09 0.0003105

[JK|E] -58.09 0.0003105

[JL|E] -58.09 0.0003105

[H|GHJ] -58.1 0.0003073

[H|CHJ] -58.1 0.0003073

[H|DHJ] -58.1 0.0003073

[H|EHJ] -58.1 0.0003073

[H|FHJ] -58.1 0.0003073

[L|DKL] -58.1 0.0003069

[K|DKL] -58.1 0.0003069

[L|CKL] -58.1 0.0003069

[K|CKL] -58.1 0.0003069

[L|FKL] -58.1 0.0003069

[K|FKL] -58.1 0.0003069

[L|EKL] -58.1 0.0003069

[K|EKL] -58.1 0.0003069

[L|GKL] -58.1 0.0003069

[K|GKL] -58.1 0.0003069

[A|BF] -58.1 0.0003069

[A|BC] -58.1 0.0003069

[A|BD] -58.1 0.0003069

[A|BE] -58.1 0.0003069

[A|BG] -58.1 0.0003069

[GHL|L] -58.1 0.0003063

[GHK|K] -58.1 0.0003063

[DG|J] -58.11 0.0003057

[FG|J] -58.11 0.0003057

[DFL|F] -58.11 0.0003054

[DFK|F] -58.11 0.0003054

[DFL|D] -58.11 0.0003054

[DFK|D] -58.11 0.0003054

[DK|F] -58.11 0.0003047

[DL|F] -58.11 0.0003047

[FL|C] -58.11 0.0003047

[FK|C] -58.11 0.0003047

[FL|D] -58.11 0.0003047

[DK|C] -58.11 0.0003047

[FK|D] -58.11 0.0003047

[DL|C] -58.11 0.0003047

[FL|G] -58.11 0.0003047

[FK|G] -58.11 0.0003047

[DK|G] -58.11 0.0003047

[FL|E] -58.11 0.0003047

[DL|G] -58.11 0.0003047

[FK|E] -58.11 0.0003047

[DK|E] -58.11 0.0003047

[DL|E] -58.11 0.0003047

[GHJ|J] -58.11 0.0003042

[EKL|L] -58.11 0.000304

[CKL|L] -58.11 0.000304

[EKL|K] -58.11 0.000304

[CKL|K] -58.11 0.000304

[DH|F] -58.11 0.0003035

[FH|C] -58.11 0.0003035

[FH|D] -58.11 0.0003035

[FH|G] -58.11 0.0003035

[DH|C] -58.11 0.0003035

[FH|E] -58.11 0.0003035

[DH|G] -58.11 0.0003035

[DH|E] -58.11 0.0003035

[F|DJ] -58.12 0.0003016

[F|CJ] -58.12 0.0003016

[F|GJ] -58.12 0.0003016

[D|FJ] -58.12 0.0003016

[F|EJ] -58.12 0.0003016

[D|CJ] -58.12 0.0003016

[D|GJ] -58.12 0.0003016

[D|EJ] -58.12 0.0003016

[G|DEG] -58.12 0.0003016

[G|CDG] -58.12 0.0003016

[G|EFG] -58.12 0.0003016

[G|CFG] -58.12 0.0003016

[G|DFG] -58.12 0.0003016

[G|CEG] -58.12 0.0003016

[DF|J] -58.13 0.0002971

[EJL|J] -58.13 0.000297

[EJK|J] -58.13 0.000297

[CJL|J] -58.13 0.000297

[CJK|J] -58.13 0.000297

[FHL|L] -58.14 0.0002961

[DHL|L] -58.14 0.0002961

[FHK|K] -58.14 0.0002961

[DHK|K] -58.14 0.0002961

[GKL|L] -58.14 0.0002947

[GKL|K] -58.14 0.0002947

[FHJ|J] -58.15 0.0002931

[DHJ|J] -58.15 0.0002931

[L|DK] -58.16 0.0002905

[K|DL] -58.16 0.0002905

[K|FL] -58.16 0.0002905

[K|GL] -58.16 0.0002905

[L|GK] -58.16 0.0002905

[K|CL] -58.16 0.0002905

[L|FK] -58.16 0.0002905

[K|EL] -58.16 0.0002905

[L|CK] -58.16 0.0002905

[L|EK] -58.16 0.0002905

[F|DEF] -58.16 0.0002904

[D|DEF] -58.16 0.0002904

[D|DEG] -58.16 0.0002904

[D|CDE] -58.16 0.0002904

[F|EFG] -58.16 0.0002904

[D|CDG] -58.16 0.0002904

[F|CDF] -58.16 0.0002904

[F|CFG] -58.16 0.0002904

[F|DFG] -58.16 0.0002904

[F|CEF] -58.16 0.0002904

[D|CDF] -58.16 0.0002904

[D|DFG] -58.16 0.0002904

[JKL|L] -58.16 0.0002896

[JKL|K] -58.16 0.0002896

[C|CFJ] -58.17 0.0002874

[C|CDJ] -58.17 0.0002874

[C|CEJ] -58.17 0.0002874

[C|CGJ] -58.17 0.0002874

[E|EFJ] -58.17 0.0002874

[E|CEJ] -58.17 0.0002874

[E|DEJ] -58.17 0.0002874

[E|EGJ] -58.17 0.0002874

[GJK|J] -58.17 0.0002873

[GJL|J] -58.17 0.0002873

[FKL|L] -58.18 0.0002841

At node N12:

split lnL Rel.Prob

[CJ|B] -53.68 0.02559

[EJ|B] -53.68 0.02559

[GJ|B] -53.8 0.02276

[FJ|B] -53.95 0.01956

[DJ|B] -53.95 0.01956

[B|B] -54.2 0.01525

[C|B] -54.48 0.01152

[E|B] -54.48 0.01152

[BCJ|B] -54.53 0.01097

[BEJ|B] -54.53 0.01097

[G|B] -54.6 0.01019

[BGJ|B] -54.65 0.009653

[CG|B] -54.66 0.009547

[EG|B] -54.66 0.009547

[F|B] -54.76 0.008705

[D|B] -54.76 0.008705

[CE|B] -54.77 0.008565

[CF|B] -54.82 0.008175

[CD|B] -54.82 0.008175

[DE|B] -54.82 0.008175

[EF|B] -54.82 0.008175

[BFJ|B] -54.84 0.008047

[BDJ|B] -54.84 0.008047

[E|E] -54.94 0.00727

[C|C] -54.94 0.00727

[G|G] -55.06 0.006434

[FG|B] -55.13 0.006009

[DG|B] -55.13 0.006009

[F|F] -55.22 0.005495

[D|D] -55.22 0.005495

[HJ|B] -55.22 0.005451

[DF|B] -55.29 0.0051

[BC|B] -55.32 0.004958

[BE|B] -55.32 0.004958

[J|BD] -55.41 0.004518

[J|BC] -55.41 0.004518

[J|BG] -55.41 0.004518

[J|BF] -55.41 0.004518

[J|BE] -55.41 0.004518

[BG|B] -55.44 0.004405

[J|BH] -55.46 0.004301

[C|BC] -55.55 0.003952

[E|BE] -55.55 0.003952

[BD|B] -55.6 0.003758

[BF|B] -55.6 0.003758

[G|BG] -55.67 0.003497

[C|AB] -55.68 0.003473

[E|AB] -55.68 0.003473

[BEG|B] -55.69 0.003421

[BCG|B] -55.69 0.003421

[J|J] -55.74 0.003243

[CL|B] -55.76 0.003207

[CK|B] -55.76 0.003207

[EK|B] -55.76 0.003207

[EL|B] -55.76 0.003207

[G|AB] -55.8 0.003074

[BCE|B] -55.81 0.003047

[F|BF] -55.83 0.002987

[D|BD] -55.83 0.002987

[BCF|B] -55.85 0.002917

[BCD|B] -55.85 0.002917

[BEF|B] -55.85 0.002917

[BDE|B] -55.85 0.002917

[GK|B] -55.87 0.002865

[GL|B] -55.87 0.002865

[C|ABC] -55.91 0.002751

[E|ABE] -55.91 0.002751

[EJ|E] -55.96 0.002626

[CJ|C] -55.96 0.002626

[F|AB] -55.96 0.002625

[D|AB] -55.96 0.002625

[CH|B] -56 0.002509

[EH|B] -56 0.002509

[FL|B] -56.02 0.002468

[FK|B] -56.02 0.002468

[DL|B] -56.02 0.002468

[DK|B] -56.02 0.002468

[EGJ|G] -56.02 0.002454

[CGJ|G] -56.02 0.002454

[EGJ|E] -56.02 0.002454

[CGJ|C] -56.02 0.002454

[G|ABG] -56.03 0.002435

[J|BDJ] -56.07 0.002345

[J|BCJ] -56.07 0.002345

[J|BGJ] -56.07 0.002345

[J|BFJ] -56.07 0.002345

[J|BEJ] -56.07 0.002345

[GJ|G] -56.07 0.002335

[J|BHJ] -56.11 0.002242

[GH|B] -56.11 0.002239

[BFG|B] -56.17 0.002124

[BDG|B] -56.17 0.002124

[F|ABF] -56.19 0.002079

[D|ABD] -56.19 0.002079

[DEJ|E] -56.2 0.002056

[DEJ|D] -56.2 0.002056

[CFJ|F] -56.2 0.002056

[EFJ|E] -56.2 0.002056

[CFJ|C] -56.2 0.002056

[EFJ|F] -56.2 0.002056

[CDJ|D] -56.2 0.002056

[CDJ|C] -56.2 0.002056

[CEJ|E] -56.21 0.002027

[CEJ|C] -56.21 0.002027

[FJ|F] -56.22 0.002007

[DJ|D] -56.22 0.002007

[H|B] -56.23 0.002

[C|BD] -56.23 0.001993

[C|BG] -56.23 0.001993

[C|BE] -56.23 0.001993

[E|BD] -56.23 0.001993

[C|BF] -56.23 0.001993

[E|BC] -56.23 0.001993

[E|BG] -56.23 0.001993

[E|BF] -56.23 0.001993

[FH|B] -56.26 0.001927

[DH|B] -56.26 0.001927

[C|BH] -56.3 0.001864

[E|BH] -56.3 0.001864

[BDF|B] -56.34 0.001792

[G|BC] -56.35 0.001764

[G|BD] -56.35 0.001764

[G|BE] -56.35 0.001764

[G|BF] -56.35 0.001764

[G|BH] -56.42 0.00165

[C|BCF] -56.46 0.001579

[C|BCG] -56.46 0.001579

[E|BEG] -56.46 0.001579

[C|BCD] -56.46 0.001579

[C|BCE] -56.46 0.001579

[E|BCE] -56.46 0.001579

[E|BEF] -56.46 0.001579

[E|BDE] -56.46 0.001579

[C|BCH] -56.49 0.00154

[E|BEH] -56.49 0.00154

[F|BD] -56.51 0.001506

[F|BC] -56.51 0.001506

[F|BE] -56.51 0.001506

[F|BG] -56.51 0.001506

[D|BC] -56.51 0.001506

[D|BG] -56.51 0.001506

[D|BE] -56.51 0.001506

[D|BF] -56.51 0.001506

[F|BH] -56.58 0.001409

[D|BH] -56.58 0.001409

[G|BEG] -56.59 0.001397

[G|BCG] -56.59 0.001397

[G|BDG] -56.59 0.001397

[G|BFG] -56.59 0.001397

[FGJ|G] -56.6 0.001378

[DGJ|G] -56.6 0.001378

[FGJ|F] -56.6 0.001378

[DGJ|D] -56.6 0.001378

[G|BGH] -56.61 0.001363

[J|G] -56.62 0.001352

[J|D] -56.62 0.001352

[J|E] -56.62 0.001352

[J|F] -56.62 0.001352

[J|C] -56.62 0.001352

[CJ|G] -56.65 0.001317

[EJ|G] -56.65 0.001317

[CJ|F] -56.65 0.001317

[CJ|D] -56.65 0.001317

[EJ|F] -56.65 0.001317

[EJ|D] -56.65 0.001317

[CJ|E] -56.65 0.001317

[EJ|C] -56.65 0.001317

[C|BCL] -56.71 0.001231

[C|BCK] -56.71 0.001231

[E|BEK] -56.71 0.001231

[E|BEL] -56.71 0.001231

[C|BCJ] -56.73 0.001214

[E|BEJ] -56.73 0.001214

[J|H] -56.74 0.0012

[F|BCF] -56.74 0.001193

[D|BCD] -56.74 0.001193

[F|BEF] -56.74 0.001193

[F|BDF] -56.74 0.001193

[F|BFG] -56.74 0.001193

[D|BDE] -56.74 0.001193

[D|BDF] -56.74 0.001193

[D|BDG] -56.74 0.001193

[BHJ|B] -56.75 0.001191

[GJ|E] -56.76 0.001172

[GJ|F] -56.76 0.001172

[GJ|D] -56.76 0.001172

[GJ|C] -56.76 0.001172

[CJ|H] -56.76 0.001171

[EJ|H] -56.76 0.001171

[BCL|B] -56.77 0.001168

[BCK|B] -56.77 0.001168

[BEK|B] -56.77 0.001168

[BEL|B] -56.77 0.001168

[F|BFH] -56.77 0.001164

[D|BDH] -56.77 0.001164

[CG|G] -56.77 0.001163

[EG|G] -56.77 0.001163

[CG|C] -56.77 0.001163

[EG|E] -56.77 0.001163

[DFJ|D] -56.79 0.001142

[DFJ|F] -56.79 0.001142

[H|H] -56.82 0.00111

[G|BGL] -56.84 0.001089

[G|BGK] -56.84 0.001089

[G|BGJ] -56.85 0.001074

[CE|E] -56.88 0.001043

[CE|C] -56.88 0.001043

[GJ|H] -56.88 0.001041

[BGK|B] -56.89 0.001033

[BGL|B] -56.89 0.001033

[FJ|G] -56.91 0.001007

[FJ|E] -56.91 0.001007

[FJ|D] -56.91 0.001007

[DJ|G] -56.91 0.001007

[FJ|C] -56.91 0.001007

[DJ|E] -56.91 0.001007

[DJ|F] -56.91 0.001007

[DJ|C] -56.91 0.001007

[CD|D] -56.93 0.0009956

[DE|E] -56.93 0.0009956

[DE|D] -56.93 0.0009956

[CF|F] -56.93 0.0009956

[CD|C] -56.93 0.0009956

[CF|C] -56.93 0.0009956

[EF|E] -56.93 0.0009956

[EF|F] -56.93 0.0009956

[F|BFK] -56.99 0.0009304

[D|BDL] -56.99 0.0009304

[D|BDK] -56.99 0.0009304

[F|BFL] -56.99 0.0009304

[D|BDJ] -57.01 0.0009172

[F|BFJ] -57.01 0.0009172

[FJ|H] -57.03 0.0008949

[DJ|H] -57.03 0.0008949

[BH|B] -57.04 0.0008875

[BCH|B] -57.04 0.0008869

[BEH|B] -57.04 0.0008869

[BFL|B] -57.06 0.0008698

[BDL|B] -57.06 0.0008698

[BFK|B] -57.06 0.0008698

[BDK|B] -57.06 0.0008698

[BGH|B] -57.16 0.0007905

[J|GJ] -57.21 0.0007508

[J|CJ] -57.21 0.0007508

[J|EJ] -57.21 0.0007508

[J|DJ] -57.21 0.0007508

[J|FJ] -57.21 0.0007508

[B|AB] -57.23 0.0007353

[DG|G] -57.23 0.0007319

[FG|G] -57.23 0.0007319

[DG|D] -57.23 0.0007319

[FG|F] -57.23 0.0007319

[C|G] -57.27 0.0007075

[C|D] -57.27 0.0007075

[C|F] -57.27 0.0007075

[C|E] -57.27 0.0007075

[E|G] -57.27 0.0007075

[E|F] -57.27 0.0007075

[E|D] -57.27 0.0007075

[E|C] -57.27 0.0007075

[HL|B] -57.27 0.0007038

[HK|B] -57.27 0.0007038

[BEJ|E] -57.3 0.000681

[BCJ|C] -57.3 0.000681

[BDH|B] -57.31 0.0006744

[BFH|B] -57.31 0.0006744

[J|HJ] -57.33 0.0006664

[H|BH] -57.38 0.0006325

[C|H] -57.39 0.0006266

[E|H] -57.39 0.0006266

[G|D] -57.39 0.0006261

[G|E] -57.39 0.0006261

[G|F] -57.39 0.0006261

[G|C] -57.39 0.0006261

[CJ|J] -57.39 0.0006235

[EJ|J] -57.39 0.0006235

[DF|D] -57.4 0.0006212

[DF|F] -57.4 0.0006212

[C|CF] -57.42 0.0006071

[C|CD] -57.42 0.0006071

[C|CG] -57.42 0.0006071

[E|EG] -57.42 0.0006071

[C|CE] -57.42 0.0006071

[E|DE] -57.42 0.0006071

[E|CE] -57.42 0.0006071

[E|EF] -57.42 0.0006071

[H|AB] -57.43 0.0006038

[BGJ|G] -57.43 0.0005994

[ABE|B] -57.46 0.0005854

[ABC|B] -57.46 0.0005854

[CG|E] -57.46 0.0005828

[CG|D] -57.46 0.0005828

[CG|F] -57.46 0.0005828

[EG|F] -57.46 0.0005828

[EG|D] -57.46 0.0005828

[EG|C] -57.46 0.0005828

[EGJ|J] -57.46 0.0005828

[CGJ|J] -57.46 0.0005828

[J|EG] -57.46 0.0005819

[J|CF] -57.46 0.0005819

[J|CG] -57.46 0.0005819

[J|CD] -57.46 0.0005819

[J|DE] -57.46 0.0005819

[J|CE] -57.46 0.0005819

[J|FG] -57.46 0.0005819

[J|EF] -57.46 0.0005819

[J|DF] -57.46 0.0005819

[J|DG] -57.46 0.0005819

[CEG|G] -57.47 0.0005757

[CEG|E] -57.47 0.0005757

[CEG|C] -57.47 0.0005757

[GJ|J] -57.51 0.0005546

[G|H] -57.51 0.0005545

[C|CH] -57.52 0.0005506

[E|EH] -57.52 0.0005506

[G|EG] -57.54 0.0005372

[G|CG] -57.54 0.0005372

[G|DG] -57.54 0.0005372

[G|FG] -57.54 0.0005372

[F|E] -57.55 0.0005347

[F|G] -57.55 0.0005347

[F|D] -57.55 0.0005347

[F|C] -57.55 0.0005347

[D|G] -57.55 0.0005347

[D|E] -57.55 0.0005347

[D|F] -57.55 0.0005347

[D|C] -57.55 0.0005347

[J|CH] -57.56 0.0005295

[J|GH] -57.56 0.0005295

[J|FH] -57.56 0.0005295

[J|DH] -57.56 0.0005295

[J|EH] -57.56 0.0005295

[CE|G] -57.57 0.0005229

[CE|F] -57.57 0.0005229

[CE|D] -57.57 0.0005229

[B|BC] -57.57 0.0005212

[B|BG] -57.57 0.0005212

[B|BD] -57.57 0.0005212

[B|BE] -57.57 0.0005212

[B|BF] -57.57 0.0005212

[ABG|B] -57.57 0.000521

[EJL|E] -57.58 0.0005175

[EJK|E] -57.58 0.0005175

[CJL|C] -57.58 0.0005175

[CJK|C] -57.58 0.0005175

[CG|H] -57.58 0.0005173

[EG|H] -57.58 0.0005173

[CHJ|C] -57.59 0.0005096

[EHJ|E] -57.59 0.0005096

[BDJ|D] -57.61 0.0004997

[BFJ|F] -57.61 0.0004997

[CD|G] -57.62 0.000499

[CF|G] -57.62 0.000499

[DE|G] -57.62 0.000499

[CF|D] -57.62 0.000499

[CD|F] -57.62 0.000499

[CF|E] -57.62 0.000499

[DE|F] -57.62 0.000499

[CD|E] -57.62 0.000499

[EF|G] -57.62 0.000499

[EF|D] -57.62 0.000499

[DE|C] -57.62 0.000499

[EF|C] -57.62 0.000499

[CDE|E] -57.62 0.0004955

[CDE|D] -57.62 0.0004955

[CEF|E] -57.62 0.0004955

[CEF|F] -57.62 0.0004955

[CDE|C] -57.62 0.0004955

[CEF|C] -57.62 0.0004955

[B|BH] -57.64 0.0004892

[DEJ|J] -57.64 0.0004884

[CFJ|J] -57.64 0.0004884

[EFJ|J] -57.64 0.0004884

[CDJ|J] -57.64 0.0004884

[G|GH] -57.64 0.0004872

[CEJ|J] -57.65 0.0004814

[HJ|H] -57.65 0.0004811

[L|BD] -57.65 0.00048

[L|BC] -57.65 0.00048

[L|BF] -57.65 0.00048

[L|BG] -57.65 0.00048

[K|BC] -57.65 0.00048

[K|BG] -57.65 0.00048

[L|BE] -57.65 0.00048

[K|BD] -57.65 0.00048

[K|BE] -57.65 0.00048

[K|BF] -57.65 0.00048

[FJ|J] -57.66 0.0004766

[DJ|J] -57.66 0.0004766

[F|H] -57.67 0.0004736

[D|H] -57.67 0.0004736

[CE|H] -57.69 0.0004641

[H|ABH] -57.69 0.0004636

[GJL|G] -57.69 0.0004624

[GJK|G] -57.69 0.0004624

[DEG|G] -57.69 0.0004617

[CDG|G] -57.69 0.0004617

[EFG|G] -57.69 0.0004617

[CFG|G] -57.69 0.0004617

[DEG|E] -57.69 0.0004617

[DEG|D] -57.69 0.0004617

[CDG|D] -57.69 0.0004617

[EFG|E] -57.69 0.0004617

[EFG|F] -57.69 0.0004617

[CFG|F] -57.69 0.0004617

[CDG|C] -57.69 0.0004617

[CFG|C] -57.69 0.0004617

[AB|B] -57.7 0.000459

[F|CF] -57.7 0.0004588

[F|DF] -57.7 0.0004588

[F|EF] -57.7 0.0004588

[D|CD] -57.7 0.0004588

[F|FG] -57.7 0.0004588

[D|DE] -57.7 0.0004588

[D|DF] -57.7 0.0004588

[D|DG] -57.7 0.0004588

[L|BH] -57.7 0.0004567

[K|BH] -57.7 0.0004567

[GHJ|G] -57.71 0.0004544

[CHJ|H] -57.73 0.0004469

[EHJ|H] -57.73 0.0004469

[CF|H] -57.74 0.0004429

[CD|H] -57.74 0.0004429

[DE|H] -57.74 0.0004429

[EF|H] -57.74 0.0004429

[ABF|B] -57.74 0.0004426

[ABD|B] -57.74 0.0004426

[F|FH] -57.8 0.0004161

[D|DH] -57.8 0.0004161

[GHJ|H] -57.84 0.0003985

[DEF|E] -57.84 0.0003976

[DEF|D] -57.84 0.0003976

[DEF|F] -57.84 0.0003976

[CDF|D] -57.84 0.0003976

[CDF|F] -57.84 0.0003976

[CDF|C] -57.84 0.0003976

[FJK|F] -57.85 0.000396

[FJL|F] -57.85 0.000396

[DJL|D] -57.85 0.000396

[DJK|D] -57.85 0.000396

[C|CL] -57.85 0.000394

[C|CK] -57.85 0.000394

[E|EK] -57.85 0.000394

[E|EL] -57.85 0.000394

[C|CJ] -57.87 0.0003886

[E|EJ] -57.87 0.0003886

[FHJ|F] -57.87 0.0003882

[DHJ|D] -57.87 0.0003882

[BC|A] -57.88 0.000382

[BE|A] -57.88 0.000382

[J|CL] -57.89 0.0003799

[J|DL] -57.89 0.0003799

[J|FK] -57.89 0.0003799

[J|CK] -57.89 0.0003799

[J|DK] -57.89 0.0003799

[J|GK] -57.89 0.0003799

[J|GL] -57.89 0.0003799

[J|EK] -57.89 0.0003799

[J|FL] -57.89 0.0003799

[J|EL] -57.89 0.0003799

[FG|E] -57.92 0.0003668

[FG|D] -57.92 0.0003668

[DG|E] -57.92 0.0003668

[DG|F] -57.92 0.0003668

[FG|C] -57.92 0.0003668

[DG|C] -57.92 0.0003668

[B|ABC] -57.93 0.0003629

[B|ABG] -57.93 0.0003629

[B|ABD] -57.93 0.0003629

[B|ABF] -57.93 0.0003629

[B|ABE] -57.93 0.0003629

[B|ABH] -57.95 0.0003586

[J|HI] -57.96 0.0003547

[HI|B] -57.96 0.000354

[G|GK] -57.97 0.0003487

[G|GL] -57.97 0.0003487

[L|L] -57.98 0.0003455

[K|K] -57.98 0.0003455

[H|BD] -57.99 0.0003449

[H|BC] -57.99 0.0003449

[H|BG] -57.99 0.0003449

[H|BE] -57.99 0.0003449

[H|BF] -57.99 0.0003449

[G|GJ] -57.99 0.0003439

[FHJ|H] -58 0.0003404

[DHJ|H] -58 0.0003404

[BG|A] -58 0.0003394

[J|HK] -58.01 0.0003377

[J|HL] -58.01 0.0003377

[EL|E] -58.03 0.000329

[CL|C] -58.03 0.000329

[EK|E] -58.03 0.000329

[CK|C] -58.03 0.000329

[FGJ|J] -58.04 0.0003272

[DGJ|J] -58.04 0.0003272

[FG|H] -58.04 0.0003256

[DG|H] -58.04 0.0003256

[J|K] -58.05 0.0003225

[J|L] -58.05 0.0003225

[JL|G] -58.06 0.0003207

[JL|E] -58.06 0.0003207

[JL|D] -58.06 0.0003207

[JL|F] -58.06 0.0003207

[JK|G] -58.06 0.0003207

[JK|D] -58.06 0.0003207

[JK|E] -58.06 0.0003207

[JL|C] -58.06 0.0003207

[JK|F] -58.06 0.0003207

[JK|C] -58.06 0.0003207

[CJ|L] -58.07 0.0003155

[CJ|K] -58.07 0.0003155

[EJ|L] -58.07 0.0003155

[EJ|K] -58.07 0.0003155

[DF|G] -58.09 0.0003114

[DF|E] -58.09 0.0003114

[DF|C] -58.09 0.0003114

[BE|E] -58.1 0.0003079

[BC|C] -58.1 0.0003079

[C|EG] -58.11 0.0003043

[E|CF] -58.11 0.0003043

[E|CG] -58.11 0.0003043

[C|DF] -58.11 0.0003043

[C|DG] -58.11 0.0003043

[C|DE] -58.11 0.0003043

[C|EF] -58.11 0.0003043

[E|FG] -58.11 0.0003043

[C|FG] -58.11 0.0003043

[E|CD] -58.11 0.0003043

[E|DF] -58.11 0.0003043

[E|DG] -58.11 0.0003043

[CH|C] -58.11 0.000304

[EH|E] -58.11 0.000304

[F|FK] -58.13 0.0002978

[D|DL] -58.13 0.0002978

[F|FL] -58.13 0.0002978

[D|DK] -58.13 0.0002978

[GK|G] -58.15 0.0002939

[GL|G] -58.15 0.0002939

[F|FJ] -58.15 0.0002937

[D|DJ] -58.15 0.0002937

[BF|A] -58.16 0.0002895

[BD|A] -58.16 0.0002895

[JL|H] -58.18 0.0002852

[JK|H] -58.18 0.0002852

[GJ|L] -58.19 0.0002806

[GJ|K] -58.19 0.0002806

[C|GH] -58.21 0.0002765

[C|FH] -58.21 0.0002765

[E|FH] -58.21 0.0002765

[E|CH] -58.21 0.0002765

[C|EH] -58.21 0.0002765

[E|GH] -58.21 0.0002765

[C|DH] -58.21 0.0002765

[E|DH] -58.21 0.0002765

[DF|H] -58.21 0.0002763

[HJ|G] -58.21 0.0002754

[HJ|F] -58.21 0.0002754

[HJ|E] -58.21 0.0002754

[HJ|D] -58.21 0.0002754

[HJ|C] -58.21 0.0002754

[BG|G] -58.22 0.0002735

[GH|G] -58.23 0.0002713

[DFJ|J] -58.23 0.0002711

[G|CF] -58.23 0.0002693

[G|CD] -58.23 0.0002693

[G|CE] -58.23 0.0002693

[G|DE] -58.23 0.0002693

[G|EF] -58.23 0.0002693

[G|DF] -58.23 0.0002693

[CH|H] -58.24 0.0002666

[EH|H] -58.24 0.0002666

[DFG|G] -58.25 0.000265

[DFG|D] -58.25 0.000265

[DFG|F] -58.25 0.000265

[H|BCH] -58.26 0.0002625

[H|BDH] -58.26 0.0002625

[H|BFH] -58.26 0.0002625

[H|BGH] -58.26 0.0002625

[H|BEH] -58.26 0.0002625

[FL|F] -58.29 0.0002532

[DL|D] -58.29 0.0002532

[DK|D] -58.29 0.0002532

[FK|F] -58.29 0.0002532

[L|BDL] -58.31 0.0002491

[L|BCL] -58.31 0.0002491

[L|BGL] -58.31 0.0002491

[K|BDK] -58.31 0.0002491

[K|BGK] -58.31 0.0002491

[K|BCK] -58.31 0.0002491

[K|BFK] -58.31 0.0002491

[L|BEL] -58.31 0.0002491

[K|BEK] -58.31 0.0002491

[L|BFL] -58.31 0.0002491

[J|EGJ] -58.32 0.0002467

[J|CFJ] -58.32 0.0002467

[J|DEJ] -58.32 0.0002467

[J|CGJ] -58.32 0.0002467

[J|EFJ] -58.32 0.0002467

[J|FGJ] -58.32 0.0002467

[J|DGJ] -58.32 0.0002467

[J|CEJ] -58.32 0.0002467

[J|DFJ] -58.32 0.0002467

[J|CDJ] -58.32 0.0002467

[G|CH] -58.33 0.0002447

[G|FH] -58.33 0.0002447

[G|DH] -58.33 0.0002447

[G|EH] -58.33 0.0002447

[FJ|K] -58.34 0.0002412

[FJ|L] -58.34 0.0002412

[DJ|K] -58.34 0.0002412

[DJ|L] -58.34 0.0002412

[L|BHL] -58.36 0.0002381

[K|BHK] -58.36 0.0002381

[GH|H] -58.36 0.000238

[FH|F] -58.38 0.0002335

[DH|D] -58.38 0.0002335

[BD|D] -58.38 0.0002334

[BF|F] -58.38 0.0002334

[J|GHJ] -58.39 0.0002311

[J|FHJ] -58.39 0.0002311

[J|CHJ] -58.39 0.0002311

[J|EHJ] -58.39 0.0002311

[J|DHJ] -58.39 0.0002311

[F|EG] -58.39 0.00023

[F|CG] -58.39 0.00023

[F|CD] -58.39 0.00023

[F|DE] -58.39 0.00023

[F|CE] -58.39 0.00023

[F|DG] -58.39 0.00023

[D|CF] -58.39 0.00023

[D|EG] -58.39 0.00023

[D|CG] -58.39 0.00023

[D|FG] -58.39 0.00023

[D|CE] -58.39 0.00023

[D|EF] -58.39 0.00023

[CGK|G] -58.44 0.0002187

[CGL|G] -58.44 0.0002187

[EGL|G] -58.44 0.0002187

[EGL|E] -58.44 0.0002187

[EGK|G] -58.44 0.0002187

[CGK|C] -58.44 0.0002187

[CGL|C] -58.44 0.0002187

[EGK|E] -58.44 0.0002187

[CGH|G] -58.45 0.000216

[EGH|G] -58.45 0.000216

[CGH|C] -58.45 0.000216

[EGH|E] -58.45 0.000216

[BEG|G] -58.47 0.0002124

[BCG|G] -58.47 0.0002124

[BEG|E] -58.47 0.0002124

[BCG|C] -58.47 0.0002124

[F|CH] -58.49 0.000209

[F|GH] -58.49 0.000209

[F|EH] -58.49 0.000209

[F|DH] -58.49 0.000209

[D|CH] -58.49 0.000209

[D|FH] -58.49 0.000209

[D|GH] -58.49 0.000209

[D|EH] -58.49 0.000209

[B|BEG] -58.49 0.0002082

[B|BCF] -58.49 0.0002082

[B|BCG] -58.49 0.0002082

[B|BCD] -58.49 0.0002082

[B|BCE] -58.49 0.0002082

[B|BEF] -58.49 0.0002082

[B|BDE] -58.49 0.0002082

[B|BDF] -58.49 0.0002082

[B|BDG] -58.49 0.0002082

[B|BFG] -58.49 0.0002082

[FH|H] -58.51 0.0002048

[DH|H] -58.51 0.0002048

[B|BCH] -58.51 0.0002031

[B|BDH] -58.51 0.0002031

[B|BFH] -58.51 0.0002031

[B|BGH] -58.51 0.0002031

[B|BEH] -58.51 0.0002031

[H|BHK] -58.53 0.0002007

[H|BHL] -58.53 0.0002007

[H|BHJ] -58.54 0.0001979

[C|DL] -58.54 0.0001979

[C|FK] -58.54 0.0001979

[E|CL] -58.54 0.0001979

[C|GK] -58.54 0.0001979

[E|DL] -58.54 0.0001979

[C|GL] -58.54 0.0001979

[E|FK] -58.54 0.0001979

[C|DK] -58.54 0.0001979

[E|CK] -58.54 0.0001979

[E|GK] -58.54 0.0001979

[E|GL] -58.54 0.0001979

[C|FL] -58.54 0.0001979

[C|EK] -58.54 0.0001979

[C|EL] -58.54 0.0001979

[E|DK] -58.54 0.0001979

[E|FL] -58.54 0.0001979

[J|JK] -58.54 0.0001971

[J|JL] -58.54 0.0001971

[C|GJ] -58.56 0.000195

[E|GJ] -58.56 0.000195

[C|FJ] -58.56 0.000195

[C|DJ] -58.56 0.000195

[C|EJ] -58.56 0.000195

[E|FJ] -58.56 0.000195

[E|CJ] -58.56 0.000195

[E|DJ] -58.56 0.000195

[CEL|E] -58.56 0.0001948

[CEK|E] -58.56 0.0001948

[CEL|C] -58.56 0.0001948

[CEK|C] -58.56 0.0001948

[CEH|E] -58.57 0.0001917

[CEH|C] -58.57 0.0001917

[H|BHI] -58.58 0.0001908

[CGH|H] -58.58 0.0001894

[EGH|H] -58.58 0.0001894

[BCE|E] -58.59 0.0001892

[BCE|C] -58.59 0.0001892

[B|A] -58.59 0.0001879

[CFL|F] -58.6 0.0001868

[CFL|C] -58.6 0.0001868

[DEL|E] -58.6 0.0001868

[DEL|D] -58.6 0.0001868

[CDL|D] -58.6 0.0001868

[DEK|E] -58.6 0.0001868

[DEK|D] -58.6 0.0001868

[CDK|D] -58.6 0.0001868

[EFK|E] -58.6 0.0001868

[EFL|E] -58.6 0.0001868

[EFK|F] -58.6 0.0001868

[CDL|C] -58.6 0.0001868

[EFL|F] -58.6 0.0001868

[CDK|C] -58.6 0.0001868

[CFK|F] -58.6 0.0001868

[CFK|C] -58.6 0.0001868

[C|HI] -58.61 0.000185

[E|HI] -58.61 0.000185

[CDH|D] -58.61 0.0001841

[EFH|E] -58.61 0.0001841

[CFH|F] -58.61 0.0001841

[CDH|C] -58.61 0.0001841

[EFH|F] -58.61 0.0001841

[CFH|C] -58.61 0.0001841

[DEH|E] -58.61 0.0001841

[DEH|D] -58.61 0.0001841

[BCF|F] -58.63 0.0001812

[BCD|D] -58.63 0.0001812

[BEF|E] -58.63 0.0001812

[BEF|F] -58.63 0.0001812

[BCF|C] -58.63 0.0001812

[BCD|C] -58.63 0.0001812

[BDE|E] -58.63 0.0001812

[BDE|D] -58.63 0.0001812

[C|HK] -58.66 0.0001757

[C|HL] -58.66 0.0001757

[E|HK] -58.66 0.0001757

[E|HL] -58.66 0.0001757

[G|CL] -58.66 0.0001751

[G|DL] -58.66 0.0001751

[G|FK] -58.66 0.0001751

[G|CK] -58.66 0.0001751

[G|DK] -58.66 0.0001751

[G|FL] -58.66 0.0001751

[G|EK] -58.66 0.0001751

[G|EL] -58.66 0.0001751

[C|CEF] -58.66 0.0001749

[C|CFG] -58.66 0.0001749

[C|CDG] -58.66 0.0001749

[C|CDF] -58.66 0.0001749

[C|CEG] -58.66 0.0001749

[E|CEF] -58.66 0.0001749

[C|CDE] -58.66 0.0001749

[E|CEG] -58.66 0.0001749

[E|CDE] -58.66 0.0001749

[E|EFG] -58.66 0.0001749

[E|DEG] -58.66 0.0001749

[E|DEF] -58.66 0.0001749

[C|HJ] -58.67 0.0001732

[E|HJ] -58.67 0.0001732

[G|EJ] -58.68 0.0001726

[G|CJ] -58.68 0.0001726

[G|FJ] -58.68 0.0001726

[G|DJ] -58.68 0.0001726

[C|L] -58.7 0.0001685

[C|K] -58.7 0.0001685

[E|L] -58.7 0.0001685

[E|K] -58.7 0.0001685

[CEH|H] -58.7 0.0001681

[C|J] -58.72 0.0001662

[E|J] -58.72 0.0001662

[C|CDH] -58.72 0.0001658

[C|CFH] -58.72 0.0001658

[C|CGH] -58.72 0.0001658

[C|CEH] -58.72 0.0001658

[E|EFH] -58.72 0.0001658

[E|DEH] -58.72 0.0001658

[E|CEH] -58.72 0.0001658

[E|EGH] -58.72 0.0001658

[CL|G] -58.72 0.0001653

[CL|D] -58.72 0.0001653

[CL|F] -58.72 0.0001653

[EL|G] -58.72 0.0001653

[CK|G] -58.72 0.0001653

[CL|E] -58.72 0.0001653

[EK|G] -58.72 0.0001653

[CK|F] -58.72 0.0001653

[EL|F] -58.72 0.0001653

[EL|D] -58.72 0.0001653

[CK|E] -58.72 0.0001653

[EK|F] -58.72 0.0001653

[CK|D] -58.72 0.0001653

[EK|D] -58.72 0.0001653

[EL|C] -58.72 0.0001653

[EK|C] -58.72 0.0001653

[G|HI] -58.73 0.0001637

[B|BDL] -58.74 0.0001624

[B|BGL] -58.74 0.0001624

[B|BCL] -58.74 0.0001624

[B|BGK] -58.74 0.0001624

[B|BDK] -58.74 0.0001624

[B|BCK] -58.74 0.0001624

[B|BFK] -58.74 0.0001624

[B|BEK] -58.74 0.0001624

[B|BEL] -58.74 0.0001624

[B|BFL] -58.74 0.0001624

[BCJ|J] -58.74 0.0001617

[BEJ|J] -58.74 0.0001617

[BHL|B] -58.74 0.0001616

[BHK|B] -58.74 0.0001616

[CDH|H] -58.74 0.0001615

[CFH|H] -58.74 0.0001615

[EFH|H] -58.74 0.0001615

[DEH|H] -58.74 0.0001615

[B|BDJ] -58.75 0.0001601

[B|BCJ] -58.75 0.0001601

[B|BGJ] -58.75 0.0001601

[B|BEJ] -58.75 0.0001601

[B|BFJ] -58.75 0.0001601

[J|HIJ] -58.77 0.000157

[G|HK] -58.78 0.0001555

[G|HL] -58.78 0.0001555

[BC|G] -58.78 0.0001553

[BE|G] -58.78 0.0001553

[BC|F] -58.78 0.0001553

[BC|E] -58.78 0.0001553

[BC|D] -58.78 0.0001553

[BE|F] -58.78 0.0001553

[BE|D] -58.78 0.0001553

[BE|C] -58.78 0.0001553

[B|BHK] -58.78 0.0001552

[B|BHL] -58.78 0.0001552

[G|CFG] -58.79 0.0001548

[G|CDG] -58.79 0.0001548

[G|CEG] -58.79 0.0001548

[G|DFG] -58.79 0.0001548

[G|EFG] -58.79 0.0001548

[G|DEG] -58.79 0.0001548

[G|HJ] -58.8 0.0001532

[B|BHJ] -58.8 0.0001531

[CH|G] -58.8 0.0001527

[CH|F] -58.8 0.0001527

[CH|E] -58.8 0.0001527

[CH|D] -58.8 0.0001527

[EH|G] -58.8 0.0001527

[EH|F] -58.8 0.0001527

[EH|D] -58.8 0.0001527

[EH|C] -58.8 0.0001527

[JL|J] -58.81 0.0001512

[JL|L] -58.81 0.0001512

[JK|K] -58.81 0.0001512

[JK|J] -58.81 0.0001512

[F|CL] -58.82 0.0001496

[F|DL] -58.82 0.0001496

[F|GK] -58.82 0.0001496

[F|CK] -58.82 0.0001496

[F|GL] -58.82 0.0001496

[F|DK] -58.82 0.0001496

[D|CL] -58.82 0.0001496

[F|EK] -58.82 0.0001496

[D|FK] -58.82 0.0001496

[F|EL] -58.82 0.0001496

[D|CK] -58.82 0.0001496

[D|GL] -58.82 0.0001496

[D|GK] -58.82 0.0001496

[D|FL] -58.82 0.0001496

[D|EK] -58.82 0.0001496

[D|EL] -58.82 0.0001496

[G|K] -58.82 0.0001491

[G|L] -58.82 0.0001491

[J|GJK] -58.83 0.0001489

[J|FJK] -58.83 0.0001489

[J|CJL] -58.83 0.0001489

[J|FJL] -58.83 0.0001489

[J|CJK] -58.83 0.0001489

[J|GJL] -58.83 0.0001489

[J|DJL] -58.83 0.0001489

[J|DJK] -58.83 0.0001489

[J|EJK] -58.83 0.0001489

[J|EJL] -58.83 0.0001489

[GL|F] -58.83 0.0001476

[GK|F] -58.83 0.0001476

[GL|E] -58.83 0.0001476

[GK|E] -58.83 0.0001476

[GL|D] -58.83 0.0001476

[GK|D] -58.83 0.0001476

[GL|C] -58.83 0.0001476

[GK|C] -58.83 0.0001476

[B|BHI] -58.83 0.0001476

[F|GJ] -58.84 0.0001474

[F|CJ] -58.84 0.0001474

[F|EJ] -58.84 0.0001474

[F|DJ] -58.84 0.0001474

[D|GJ] -58.84 0.0001474

[D|CJ] -58.84 0.0001474

[D|EJ] -58.84 0.0001474

[D|FJ] -58.84 0.0001474

[G|J] -58.84 0.000147

[CL|H] -58.84 0.0001468

[EL|H] -58.84 0.0001468

[CK|H] -58.84 0.0001468

[EK|H] -58.84 0.0001468

[G|FGH] -58.84 0.0001467

[G|CGH] -58.84 0.0001467

[G|DGH] -58.84 0.0001467

[G|EGH] -58.84 0.0001467

[L|G] -58.86 0.0001435

[L|D] -58.86 0.0001435

[K|G] -58.86 0.0001435

[L|E] -58.86 0.0001435

[L|F] -58.86 0.0001435

[K|E] -58.86 0.0001435

[K|D] -58.86 0.0001435

[K|F] -58.86 0.0001435

[L|C] -58.86 0.0001435

[K|C] -58.86 0.0001435

[BGJ|J] -58.87 0.0001424

[F|HI] -58.89 0.0001398

[D|HI] -58.89 0.0001398

[CG|L] -58.89 0.0001391

[CG|K] -58.89 0.0001391

[EG|L] -58.89 0.0001391

[EG|K] -58.89 0.0001391

[BC|H] -58.9 0.0001382

[BE|H] -58.9 0.0001382

[BG|E] -58.9 0.000138

[BG|D] -58.9 0.000138

[BG|F] -58.9 0.000138

[BG|C] -58.9 0.000138

At node N10:

split lnL Rel.Prob

[CG|J] -52.21 0.1106

[EG|J] -52.21 0.1106

[EF|J] -52.44 0.08811

[CD|J] -52.44 0.08811

[CF|J] -52.44 0.08811

[DE|J] -52.44 0.08811

[CE|J] -52.55 0.07881

[FG|J] -53.11 0.0453

[DG|J] -53.11 0.0453

[DF|J] -53.41 0.03355

[C|J] -54.66 0.009579

[E|J] -54.66 0.009579

[G|J] -54.76 0.008679

[CGJ|J] -54.78 0.008523

[EGJ|J] -54.78 0.008523

[F|J] -54.98 0.006938

[D|J] -54.98 0.006938

[CDJ|J] -55.06 0.006398

[EFJ|J] -55.06 0.006398

[DEJ|J] -55.06 0.006398

[CFJ|J] -55.06 0.006398

[CEJ|J] -55.42 0.004484

[FGJ|J] -55.78 0.003126

[DGJ|J] -55.78 0.003126

[CEG|C] -55.82 0.003018

[CEG|G] -55.82 0.003018

[CEG|E] -55.82 0.003018

[CEF|C] -56.08 0.002327

[CDE|C] -56.08 0.002327

[CDE|D] -56.08 0.002327

[CEF|E] -56.08 0.002327

[CEF|F] -56.08 0.002327

[CDE|E] -56.08 0.002327

[DFJ|J] -56.12 0.002222

[CDG|C] -56.26 0.001944

[CDG|G] -56.26 0.001944

[CDG|D] -56.26 0.001944

[CFG|C] -56.26 0.001944

[CFG|G] -56.26 0.001944

[CFG|F] -56.26 0.001944

[EFG|G] -56.26 0.001944

[DEG|G] -56.26 0.001944

[DEG|D] -56.26 0.001944

[DEG|E] -56.26 0.001944

[EFG|E] -56.26 0.001944

[EFG|F] -56.26 0.001944

[CJ|J] -56.4 0.001675

[EJ|J] -56.4 0.001675

[GJ|J] -56.44 0.001611

[BC|J] -56.54 0.001465

[BE|J] -56.54 0.001465

[BG|J] -56.58 0.001406

[CDF|C] -56.58 0.001403

[CDF|D] -56.58 0.001403

[CDF|F] -56.58 0.001403

[DEF|D] -56.58 0.001403

[DEF|E] -56.58 0.001403

[DEF|F] -56.58 0.001403

[FJ|J] -56.7 0.001242

[DJ|J] -56.7 0.001242

[BF|J] -56.84 0.001084

[BD|J] -56.84 0.001084

[C|CJ] -57.08 0.0008498

[E|EJ] -57.08 0.0008498

[J|J] -57.18 0.0007741

[G|GJ] -57.18 0.0007699

[CG|C] -57.25 0.0007201

[EG|G] -57.25 0.0007201

[CG|G] -57.25 0.0007201

[EG|E] -57.25 0.0007201

[EH|J] -57.29 0.0006933

[CH|J] -57.29 0.0006933

At node N8:

split lnL Rel.Prob

[G|CE] -52.41 0.0907

[D|CE] -52.67 0.06994

[F|CE] -52.67 0.06994

[DG|C] -52.82 0.06037

[FG|C] -52.82 0.06037

[FG|E] -52.82 0.06037

[DG|E] -52.82 0.06037

[DF|C] -53.13 0.04422

[DF|E] -53.13 0.04422

[G|C] -53.14 0.04386

[G|E] -53.14 0.04386

[D|C] -53.4 0.03382

[F|C] -53.4 0.03382

[D|E] -53.4 0.03382

[F|E] -53.4 0.03382

[G|CEG] -54.77 0.008568

[D|CDE] -55.03 0.006606

[F|CEF] -55.03 0.006606

[G|CG] -55.05 0.006472

[G|EG] -55.05 0.006472

[D|CD] -55.31 0.004991

[F|CF] -55.31 0.004991

[F|EF] -55.31 0.004991

[D|DE] -55.31 0.004991

[E|CE] -55.39 0.004636

[C|CE] -55.39 0.004636

[CG|C] -55.41 0.004546

[EG|E] -55.41 0.004546

[G|CJ] -55.56 0.003912

[G|EJ] -55.56 0.003912

[FG|F] -55.59 0.003772

[DG|D] -55.59 0.003772

[DG|G] -55.59 0.003772

[FG|G] -55.59 0.003772

[CDG|C] -55.77 0.003171

[CFG|C] -55.77 0.003171

[DEG|E] -55.77 0.003171

[EFG|E] -55.77 0.003171

[CD|C] -55.79 0.003114

[EF|E] -55.79 0.003114

[DE|E] -55.79 0.003114

[CF|C] -55.79 0.003114

[DFG|F] -55.79 0.003092

[DFG|D] -55.79 0.003092

[DFG|G] -55.79 0.003092

[D|CJ] -55.82 0.003017

[F|CJ] -55.82 0.003017

[F|EJ] -55.82 0.003017

[D|EJ] -55.82 0.003017

[J|CE] -55.88 0.002822

[G|G] -55.9 0.002769

[DF|F] -55.9 0.002762

[DF|D] -55.9 0.002762

[GJ|C] -55.91 0.002746

[GJ|E] -55.91 0.002746

[FG|J] -56.09 0.002297

[DG|J] -56.09 0.002297

[E|E] -56.1 0.002266

[C|C] -56.1 0.002266

At node N4:

split lnL Rel.Prob

[DF|G] -50.47 0.636

[D|G] -52.04 0.1319

[F|G] -52.04 0.1319

[DFG|G] -53.83 0.02202

[FG|G] -54.95 0.007207

[DG|G] -54.95 0.007207

[G|G] -55.11 0.006113

[D|DG] -55.55 0.003924

[F|FG] -55.55 0.003924

At node N2:

split lnL Rel.Prob

[F|D] -50.07 0.9476

[F|DF] -54.64 0.009765

At node N7:

split lnL Rel.Prob

[E|C] -50.3 0.749

[C|C] -53.19 0.04153

[E|E] -53.19 0.04153

[E|CE] -53.49 0.03084

[CE|C] -53.49 0.03084

[E|CG] -54.71 0.009164

[EG|C] -54.71 0.009164

[E|CD] -54.89 0.007635

[E|CF] -54.89 0.007635

[DE|C] -54.89 0.007635

[EF|C] -54.89 0.007635

[E|CJ] -54.94 0.00723

[EJ|C] -54.94 0.00723

At node N19:

split lnL Rel.Prob

[BH|K] -52.81 0.06101

[BH|L] -52.81 0.06101

[ABH|H] -53.02 0.04942

[BHL|L] -54.03 0.01798

[BHK|K] -54.03 0.01798

[H|KL] -54.14 0.01619

[H|HKL] -54.3 0.0138

[AB|E] -54.52 0.01098

[AB|C] -54.52 0.01098

[AB|G] -54.53 0.01096

[AB|D] -54.53 0.01094

[AB|F] -54.53 0.01094

[BH|H] -54.6 0.01016

[B|CK] -54.61 0.01007

[B|CL] -54.61 0.01007

[B|EL] -54.61 0.01007

[B|EK] -54.61 0.01007

[B|GL] -54.61 0.01004

[B|GK] -54.61 0.01004

[B|DL] -54.62 0.01001

[B|DK] -54.62 0.01001

[B|FL] -54.62 0.01001

[B|FK] -54.62 0.01001

[H|H] -54.67 0.00953

[AB|H] -54.68 0.009355

[H|L] -54.69 0.009326

[H|K] -54.69 0.009326

[B|HL] -54.77 0.008617

[B|HK] -54.77 0.008617

[BH|J] -54.85 0.007893

[BC|L] -55.07 0.006381

[BC|K] -55.07 0.006381

[BE|K] -55.07 0.006381

[BE|L] -55.07 0.006381

[BG|L] -55.07 0.006362

[BG|K] -55.07 0.006362

[BF|L] -55.07 0.006341

[BF|K] -55.07 0.006341

[BD|L] -55.07 0.006341

[BD|K] -55.07 0.006341

[BH|C] -55.14 0.005945

[BH|E] -55.14 0.005945

[BH|G] -55.14 0.005934

[BH|D] -55.14 0.005923

[BH|F] -55.14 0.005923

[ABH|B] -55.15 0.005884

[H|HL] -55.3 0.005054

[H|HK] -55.3 0.005054

[ABE|E] -55.54 0.003987

[ABC|C] -55.54 0.003987

[ABG|G] -55.54 0.003981

[ABD|D] -55.54 0.003973

[ABF|F] -55.54 0.003973

[HL|L] -55.7 0.003391

[HK|K] -55.7 0.003391

[B|B] -55.75 0.003219

[B|E] -55.77 0.003155

[B|C] -55.77 0.003155

[B|G] -55.77 0.003149

[H|JL] -55.78 0.003143

[H|JK] -55.78 0.003143

[B|D] -55.78 0.003142

[B|F] -55.78 0.003142

[H|CL] -55.88 0.002839

[H|CK] -55.88 0.002839

[H|EL] -55.88 0.002839

[H|EK] -55.88 0.002839

[H|GK] -55.88 0.002834

[H|GL] -55.88 0.002834

[H|DL] -55.88 0.002829

[H|DK] -55.88 0.002829

[H|FL] -55.88 0.002829

[H|FK] -55.88 0.002829

[B|BEL] -55.94 0.002664

[B|BEK] -55.94 0.002664

[B|BCL] -55.94 0.002664

[B|BCK] -55.94 0.002664

[B|BGK] -55.94 0.002656

[B|BGL] -55.94 0.002656

[B|H] -55.95 0.002648

[B|BFL] -55.95 0.002647

[B|BFK] -55.95 0.002647

[B|BDL] -55.95 0.002647

[B|BDK] -55.95 0.002647

[L|L] -56.03 0.002436

[K|K] -56.03 0.002436

[BHJ|J] -56.08 0.002327

[B|BHL] -56.09 0.002297

[B|BHK] -56.09 0.002297

[HI|L] -56.31 0.001843

[HI|K] -56.31 0.001843

[BHJ|H] -56.33 0.001809

[AB|B] -56.33 0.0018

[CH|L] -56.34 0.00179

[CH|K] -56.34 0.00179

[EH|L] -56.34 0.00179

[EH|K] -56.34 0.00179

[GH|K] -56.34 0.001787

[GH|L] -56.34 0.001787

[FH|L] -56.34 0.001784

[FH|K] -56.34 0.001784

[DH|L] -56.34 0.001784

[DH|K] -56.34 0.001784

[BHK|H] -56.34 0.001781

[BHL|H] -56.34 0.001781

[HJ|L] -56.37 0.001739

[HJ|K] -56.37 0.001739

[HK|L] -56.38 0.001711

[HL|K] -56.38 0.001711

[BCH|C] -56.41 0.00167

[BEH|E] -56.41 0.00167

[BGH|G] -56.41 0.001667

[BDH|D] -56.41 0.001664

[BFH|F] -56.41 0.001664

[BHI|H] -56.43 0.001639

[E|KL] -56.52 0.001489

[C|KL] -56.52 0.001489

[G|KL] -56.52 0.001486

[D|KL] -56.53 0.001483

[F|KL] -56.53 0.001483

[BCH|H] -56.53 0.001471

[BEH|H] -56.53 0.001471

[BGH|H] -56.54 0.001468

[BDH|H] -56.54 0.001466

[BFH|H] -56.54 0.001466

[H|HJK] -56.66 0.001293

[H|HJL] -56.66 0.001293

[C|CKL] -56.66 0.001292

[E|EKL] -56.66 0.001292

[G|GKL] -56.67 0.001289

[D|DKL] -56.67 0.001287

[F|FKL] -56.67 0.001287

[B|CJ] -56.69 0.001256

[B|EJ] -56.69 0.001256

[B|GJ] -56.7 0.001252

[B|DJ] -56.7 0.001248

[B|FJ] -56.7 0.001248

[H|EHL] -56.71 0.001231

[H|EHK] -56.71 0.001231

[H|CHL] -56.71 0.001231

[H|CHK] -56.71 0.001231

[H|GHL] -56.71 0.001229

[H|GHK] -56.71 0.001229

[H|DHK] -56.72 0.001227

[H|DHL] -56.72 0.001227

[H|FHL] -56.72 0.001227

[H|FHK] -56.72 0.001227

[BH|B] -56.73 0.001209

[H|J] -56.73 0.001206

[BC|C] -56.75 0.001188

[BE|E] -56.75 0.001188

[BG|G] -56.75 0.001186

[BD|D] -56.75 0.001184

[BF|F] -56.75 0.001184

[BCL|L] -56.82 0.001109

[BCK|K] -56.82 0.001109

[BEK|K] -56.82 0.001109

[BEL|L] -56.82 0.001109

[BGL|L] -56.82 0.001105

[BGK|K] -56.82 0.001105

[BFL|L] -56.82 0.001101

[BDL|L] -56.82 0.001101

[BDK|K] -56.82 0.001101

[BFK|K] -56.82 0.001101

[B|BC] -56.85 0.001069

[B|BE] -56.85 0.001069

[H|C] -56.85 0.001069

[H|E] -56.85 0.001069

[H|G] -56.86 0.001067

[B|BG] -56.86 0.001067

[B|HJ] -56.86 0.001066

[H|D] -56.86 0.001065

[H|F] -56.86 0.001065

[B|BF] -56.86 0.001064

[B|BD] -56.86 0.001064

[B|CE] -56.92 0.001002

[C|C] -56.92 0.001001

[E|E] -56.92 0.001001

[B|CG] -56.92 0.001001

[B|EG] -56.92 0.001001

[G|G] -56.92 0.0009995

[B|DE] -56.92 0.0009989

[B|CD] -56.92 0.0009989

[B|CF] -56.92 0.0009989

[B|EF] -56.92 0.0009989

[D|D] -56.92 0.0009975

[F|F] -56.92 0.0009975

[B|DG] -56.92 0.0009973

[B|FG] -56.92 0.0009973

[B|DF] -56.93 0.0009954

[B|BH] -57.04 0.0008902

[B|EH] -57.06 0.0008698

[B|CH] -57.06 0.0008698

[B|GH] -57.06 0.0008683

[B|FH] -57.06 0.0008666

[B|DH] -57.06 0.0008666

[C|L] -57.07 0.0008629

[E|L] -57.07 0.0008629

[E|K] -57.07 0.0008629

[C|K] -57.07 0.0008629

[G|L] -57.07 0.0008615

[G|K] -57.07 0.0008615

[D|L] -57.07 0.00086

[F|L] -57.07 0.00086

[F|K] -57.07 0.00086

[D|K] -57.07 0.00086

[L|KL] -57.11 0.0008276

[K|KL] -57.11 0.0008276

[BE|J] -57.11 0.0008248

[BC|J] -57.11 0.0008248

[BG|J] -57.12 0.0008224

[BF|J] -57.12 0.0008196

[BD|J] -57.12 0.0008196

[H|BHL] -57.14 0.0008042

[H|BHK] -57.14 0.0008042

[H|HIL] -57.24 0.0007261

[H|HIK] -57.24 0.0007261

[H|HJ] -57.39 0.0006246

[BC|E] -57.42 0.0006068

[BE|C] -57.42 0.0006068

[BC|G] -57.42 0.0006058

[BE|G] -57.42 0.0006058

[BG|C] -57.42 0.0006058

[BG|E] -57.42 0.0006058

[BC|D] -57.42 0.0006046

[BE|D] -57.42 0.0006046

[BC|F] -57.42 0.0006046

[BE|F] -57.42 0.0006046

[BD|C] -57.42 0.0006046

[BD|E] -57.42 0.0006046

[BF|E] -57.42 0.0006046

[BF|C] -57.42 0.0006046

[BD|G] -57.43 0.0006036

[BG|D] -57.43 0.0006036

[BF|G] -57.43 0.0006036

[BG|F] -57.43 0.0006036

[BF|D] -57.43 0.0006025

[BD|F] -57.43 0.0006025

[H|EH] -57.43 0.0005998

[H|CH] -57.43 0.0005998

[H|GH] -57.43 0.0005989

[H|DH] -57.44 0.0005979

[H|FH] -57.44 0.0005979

[A|BC] -57.53 0.0005437

[A|BE] -57.53 0.0005437

[A|BG] -57.53 0.0005427

[A|BF] -57.53 0.0005417

[A|BD] -57.53 0.0005417

[BC|H] -57.59 0.000514

[BE|H] -57.59 0.000514

[C|CK] -57.59 0.0005132

[C|CL] -57.59 0.0005132

[E|EL] -57.59 0.0005132

[E|EK] -57.59 0.0005132

[BG|H] -57.59 0.0005132

[G|GK] -57.59 0.0005124

[G|GL] -57.59 0.0005124

[BF|H] -57.59 0.0005122

[BD|H] -57.59 0.0005122

[D|DL] -57.59 0.0005114

[D|DK] -57.59 0.0005114

[F|FL] -57.59 0.0005114

[F|FK] -57.59 0.0005114

[HIL|L] -57.62 0.0004973

[HIK|K] -57.62 0.0004973

[B|HI] -57.63 0.0004923

[CHK|K] -57.65 0.000481

[CHL|L] -57.65 0.000481

[EHL|L] -57.65 0.000481

[EHK|K] -57.65 0.000481

[GHK|K] -57.65 0.0004802

[GHL|L] -57.65 0.0004802

[DHK|K] -57.66 0.0004793

[FHK|K] -57.66 0.0004793

[FHL|L] -57.66 0.0004793

[DHL|L] -57.66 0.0004793

[A|BH] -57.7 0.0004598

[HJ|J] -57.75 0.0004384

[HJL|L] -57.76 0.0004321

[HJK|K] -57.76 0.0004321

[HKL|L] -57.78 0.0004251

[HKL|K] -57.78 0.0004251

[J|KL] -57.78 0.0004238

[ABE|B] -57.79 0.000418

[ABC|B] -57.79 0.000418

[ABG|B] -57.79 0.0004173

[ABF|B] -57.8 0.0004165

[ABD|B] -57.8 0.0004165

[CH|C] -57.84 0.0004007

[EH|E] -57.84 0.0004007

[GH|G] -57.84 0.0004001

[DH|D] -57.84 0.0003995

[FH|F] -57.84 0.0003995

[L|JKL] -57.9 0.000375

[K|JKL] -57.9 0.000375

[J|JKL] -57.9 0.000375

[L|CKL] -57.93 0.0003658

[K|CKL] -57.93 0.0003658

[L|EKL] -57.93 0.0003658

[K|EKL] -57.93 0.0003658

[L|GKL] -57.93 0.0003652

[K|GKL] -57.93 0.0003652

[L|DKL] -57.93 0.0003645

[K|DKL] -57.93 0.0003645

[L|FKL] -57.93 0.0003645

[K|FKL] -57.93 0.0003645

[H|CJ] -57.96 0.0003542

[H|EJ] -57.96 0.0003542

[H|GJ] -57.96 0.0003536

[H|DJ] -57.96 0.000353

[H|FJ] -57.96 0.000353

[CH|H] -57.96 0.0003529

[EH|H] -57.96 0.0003529

[GH|H] -57.96 0.0003524

[FH|H] -57.97 0.0003518

[DH|H] -57.97 0.0003518

[L|HKL] -57.99 0.000345

[K|HKL] -57.99 0.000345

[HJ|H] -58 0.000341

[H|CE] -58 0.0003398

[H|CG] -58 0.0003392

[H|EG] -58 0.0003392

[H|CD] -58 0.0003386

[H|DE] -58 0.0003386

[H|CF] -58 0.0003386

[H|EF] -58 0.0003386

[H|DG] -58.01 0.0003381

[H|FG] -58.01 0.0003381

[H|DF] -58.01 0.0003375

[HL|H] -58.01 0.0003359

[HK|H] -58.01 0.0003359

[J|J] -58.07 0.0003157

[H|BH] -58.09 0.0003117

[HI|H] -58.14 0.0002958

[C|JL] -58.16 0.0002888

[C|JK] -58.16 0.0002888

[E|JL] -58.16 0.0002888

[E|JK] -58.16 0.0002888

[G|JL] -58.16 0.0002883

[G|JK] -58.16 0.0002883

[F|JL] -58.17 0.0002878

[F|JK] -58.17 0.0002878

[D|JL] -58.17 0.0002878

[D|JK] -58.17 0.0002878

[CL|L] -58.18 0.0002839

[CK|K] -58.18 0.0002839

[EL|L] -58.18 0.0002839

[EK|K] -58.18 0.0002839

[GL|L] -58.18 0.0002834

[GK|K] -58.18 0.0002834

[DL|L] -58.18 0.0002829

[FL|L] -58.18 0.0002829

[FK|K] -58.18 0.0002829

[DK|K] -58.18 0.0002829

[H|HI] -58.21 0.0002743

[B|BEJ] -58.25 0.0002659

[B|BCJ] -58.25 0.0002659

[B|BGJ] -58.25 0.0002651

[B|BFJ] -58.25 0.0002642

[B|BDJ] -58.25 0.0002642

[A|B] -58.26 0.0002632

[B|BCE] -58.26 0.0002608

[E|CL] -58.27 0.0002607

[E|CK] -58.27 0.0002607

[C|EL] -58.27 0.0002607

[C|EK] -58.27 0.0002607

[B|BEG] -58.27 0.0002603

[B|BCG] -58.27 0.0002603

[G|CK] -58.27 0.0002603

[C|GK] -58.27 0.0002603

[G|CL] -58.27 0.0002603

[E|GK] -58.27 0.0002603

[C|GL] -58.27 0.0002603

[E|GL] -58.27 0.0002603

[G|EL] -58.27 0.0002603

[G|EK] -58.27 0.0002603

[B|BCD] -58.27 0.0002599

[B|BDE] -58.27 0.0002599

[B|BCF] -58.27 0.0002599

[B|BEF] -58.27 0.0002599

[C|DK] -58.27 0.0002598

[E|DL] -58.27 0.0002598

[C|DL] -58.27 0.0002598

[E|DK] -58.27 0.0002598

[D|CK] -58.27 0.0002598

[F|CK] -58.27 0.0002598

[F|CL] -58.27 0.0002598

[C|FL] -58.27 0.0002598

[C|FK] -58.27 0.0002598

[D|CL] -58.27 0.0002598

[E|FL] -58.27 0.0002598

[E|FK] -58.27 0.0002598

[F|EL] -58.27 0.0002598

[D|EL] -58.27 0.0002598

[D|EK] -58.27 0.0002598

[F|EK] -58.27 0.0002598

[B|BDG] -58.27 0.0002594

[B|BFG] -58.27 0.0002594

[G|DL] -58.27 0.0002594

[G|DK] -58.27 0.0002594

[D|GK] -58.27 0.0002594

[F|GK] -58.27 0.0002594

[G|FL] -58.27 0.0002594

[D|GL] -58.27 0.0002594

[G|FK] -58.27 0.0002594

[F|GL] -58.27 0.0002594

[B|BDF] -58.27 0.000259

[F|DL] -58.27 0.0002589

[F|DK] -58.27 0.0002589

[D|FL] -58.27 0.0002589

[D|FK] -58.27 0.0002589

[J|L] -58.33 0.000244

[J|K] -58.33 0.000244

[L|K] -58.35 0.0002405

[K|L] -58.35 0.0002405

[HI|J] -58.35 0.0002389

[B|BCH] -58.38 0.0002322

[B|BEH] -58.38 0.0002322

[B|BGH] -58.38 0.0002318

[CH|J] -58.38 0.0002316

[EH|J] -58.38 0.0002316

[B|BDH] -58.38 0.0002313

[B|BFH] -58.38 0.0002313

[GH|J] -58.39 0.0002312

[FH|J] -58.39 0.0002308

[DH|J] -58.39 0.0002308

[C|HL] -58.4 0.0002283

[E|HL] -58.4 0.0002283

[C|HK] -58.4 0.0002283

[E|HK] -58.4 0.0002283

[G|HL] -58.4 0.0002279

[G|HK] -58.4 0.0002279

[F|HL] -58.4 0.0002275

[D|HL] -58.4 0.0002275

[D|HK] -58.4 0.0002275

[F|HK] -58.4 0.0002275

[B|BHJ] -58.41 0.0002253

[HL|J] -58.43 0.0002215

[HK|J] -58.43 0.0002215

[CE|L] -58.45 0.0002164

[CE|K] -58.45 0.0002164

[CG|K] -58.45 0.0002161

[CG|L] -58.45 0.0002161

[EG|K] -58.45 0.0002161

[EG|L] -58.45 0.0002161

[CF|K] -58.45 0.0002157

[CD|K] -58.45 0.0002157

[CF|L] -58.45 0.0002157

[DE|K] -58.45 0.0002157

[DE|L] -58.45 0.0002157

[CD|L] -58.45 0.0002157

[EF|L] -58.45 0.0002157

[EF|K] -58.45 0.0002157

[BHJ|B] -58.46 0.0002154

[FG|L] -58.46 0.0002154

[FG|K] -58.46 0.0002154

[DG|K] -58.46 0.0002154

[DG|L] -58.46 0.0002154

[DF|L] -58.46 0.000215

[DF|K] -58.46 0.000215

[BHL|B] -58.47 0.000212

[BHK|B] -58.47 0.000212

[HI|C] -58.48 0.0002112

[HI|E] -58.48 0.0002112

[HI|G] -58.48 0.0002108

[HI|D] -58.48 0.0002104

[HI|F] -58.48 0.0002104

[CH|E] -58.51 0.0002047

[EH|C] -58.51 0.0002047

[CH|G] -58.51 0.0002044

[EH|G] -58.51 0.0002044

[GH|C] -58.51 0.0002044

[GH|E] -58.51 0.0002044

[CH|D] -58.51 0.0002041

[CH|F] -58.51 0.0002041

[FH|C] -58.51 0.0002041

[FH|E] -58.51 0.0002041

[DH|C] -58.51 0.0002041

[EH|D] -58.51 0.0002041

[DH|E] -58.51 0.0002041

[EH|F] -58.51 0.0002041

[FH|G] -58.51 0.0002038

[DH|G] -58.51 0.0002038

[GH|D] -58.51 0.0002038

[GH|F] -58.51 0.0002038

[FH|D] -58.51 0.0002034

[DH|F] -58.51 0.0002034

[HJ|E] -58.54 0.0001983

[HJ|C] -58.54 0.0001983

[HJ|G] -58.54 0.000198

[HJ|D] -58.54 0.0001977

[HJ|F] -58.54 0.0001977

[HL|C] -58.55 0.0001952

[HK|C] -58.55 0.0001952

[HL|E] -58.55 0.0001952

[HK|E] -58.55 0.0001952

[BHI|B] -58.56 0.0001951

[HK|G] -58.56 0.0001949

[HL|G] -58.56 0.0001949

[HL|D] -58.56 0.0001945

[HK|D] -58.56 0.0001945

[HL|F] -58.56 0.0001945

[HK|F] -58.56 0.0001945

[H|BC] -58.62 0.0001837

[H|BE] -58.62 0.0001837

[H|BG] -58.62 0.0001834

[H|BF] -58.62 0.0001831

[H|BD] -58.62 0.0001831

[BCE|E] -58.64 0.0001789

[BCE|C] -58.64 0.0001789

[BCG|G] -58.64 0.0001786

[BEG|G] -58.64 0.0001786

[BCG|C] -58.64 0.0001786

[BEG|E] -58.64 0.0001786

[BCD|D] -58.65 0.0001783

[BDE|D] -58.65 0.0001783

[BCD|C] -58.65 0.0001783

[BCF|C] -58.65 0.0001783

[BEF|E] -58.65 0.0001783

[BDE|E] -58.65 0.0001783

[BCF|F] -58.65 0.0001783

At node N15:

split lnL Rel.Prob

[BH|A] -51.23 0.2959

[H|AB] -51.39 0.2524

[H|B] -52.83 0.05964

[ABH|A] -52.89 0.05613

[B|A] -53.02 0.04951

[H|ABH] -53.33 0.03613

[B|B] -54.31 0.01358

[AB|A] -54.42 0.01222

[H|BH] -54.52 0.011

[BC|A] -54.57 0.01048

[BE|A] -54.57 0.01048

[BG|A] -54.57 0.01047

[BD|A] -54.57 0.01045

[BF|A] -54.57 0.01045

[BH|B] -54.7 0.009169

[H|H] -54.8 0.00834

[HK|B] -55.04 0.006558

[HL|B] -55.04 0.006558

[B|AB] -55.05 0.006492

[H|BC] -55.39 0.004634

[H|BE] -55.39 0.004634

[H|BG] -55.39 0.00463

[H|BF] -55.39 0.004625

[H|BD] -55.39 0.004625

[EH|B] -55.54 0.00398

[CH|B] -55.54 0.00398

[GH|B] -55.54 0.003976

[FH|B] -55.54 0.003972

[DH|B] -55.54 0.003972

[HJ|B] -55.55 0.003948

[C|AB] -55.71 0.003345

[E|AB] -55.71 0.003345

[G|AB] -55.71 0.003341

[D|AB] -55.72 0.003336

[F|AB] -55.72 0.003336

[HI|B] -55.77 0.003165

[BHK|B] -56.06 0.002354

[BHL|B] -56.06 0.002354

[BHJ|B] -56.57 0.001417

[BCH|B] -56.65 0.001305

At node N18:

split lnL Rel.Prob

[K|L] -50.75 0.4805

[KL|L] -53.08 0.04662

[K|KL] -53.08 0.04662

[K|HL] -53.6 0.02758

[HK|L] -53.6 0.02758

[K|K] -53.68 0.02543

[L|L] -53.68 0.02543

[K|EL] -53.96 0.01929

[EK|L] -53.96 0.01929

[K|CL] -53.96 0.01929

[CK|L] -53.96 0.01929

[K|GL] -53.96 0.01927

[GK|L] -53.96 0.01927

[DK|L] -53.96 0.01925

[K|DL] -53.96 0.01925

[FK|L] -53.96 0.01925

[K|FL] -53.96 0.01925

[JK|L] -54.19 0.01539

[K|JL] -54.19 0.01539

[HKL|L] -55.53 0.004009

[K|HKL] -55.53 0.004009

[K|EKL] -55.92 0.002713

[EKL|L] -55.92 0.002713

[K|CKL] -55.92 0.002713

[CKL|L] -55.92 0.002713

[GKL|L] -55.92 0.002711

[K|GKL] -55.92 0.002711

[FKL|L] -55.92 0.002708

[DKL|L] -55.92 0.002708

[K|DKL] -55.92 0.002708

[K|FKL] -55.92 0.002708

[K|H] -56.03 0.002449

[H|L] -56.03 0.002449

[JKL|L] -56.18 0.002094

[K|JKL] -56.18 0.002094

[K|E] -56.34 0.001795

[E|L] -56.34 0.001795

***END OF LAGRANGE ANCESTRAL AREA RECONSTRUCTION RESULTS***

**SUPPLEMENTARY INFORMATION FILE S1B**

***THE FOLLOWING ARE THE LAGRANGE ANCESTRAL HOST RECONSTRUCTION RESULTS***

Lagrange: likelihood analysis of geographic range evolution

Version: 20130526

Author: Richard Ree <rree@fieldmuseum.org>

https://github.com/rhr/lagrange-python

Newick tree with interior nodes labeled:

(((((((lineage3_F:0.0166580112435,lineage3_D:0.0166580112435)N2:0.0336261725424,lineage3_G:0.0502841837859)N4:0.0821043170163,(lineage3_E:0.071546218251,lineage3_C:0.071546218251)N7:0.0608422825512)N8:0.0464777274104,lineage3_J:0.178866228213)N10:0.856628905793,lineage4_B:1.03549513401)N12:2.16772909703,((lineage2_H:0.389153852001,lineage2_A:0.389153852001)N15:0.512338691808,(lineage2_K:0.188078442474,lineage2_L:0.188078442474)N18:0.713414101335)N19:2.30173168723)N20:3.33210518255,lineage1_I:6.53532941359)N22:0.0;

Cladogram (branch lengths not to scale):

--------+ [AB] lineage3_F

------N2+

------N4+ --------+ [FN] lineage3_D

: :

------N8+ ----------------+ [C] lineage3_G

: :

: : ------------+ [EN] lineage3_E

-----N10+ ----------N7+

: : ------------+ [O] lineage3_C

-----N12+ :

: : --------------------------------+ [D] lineage3_J

: :

: ----------------------------------------+ [I] lineage4_B

-----N20+

: : ----------------+ [GK] lineage2_H

: : -------------N15+

: : : ----------------+ [H] lineage2_A

N22+ -------------N19+

: : ----------------+ [JK] lineage2_K

: -------------N18+

: ----------------+ [L] lineage2_L

:

--------------------------------------------------------+ [M] lineage1_I

***RESULTS OF RUN FROM Margo_host.lagrange.py INPUT FILE SUBMITTED TO DRYAD***

Global ML at root node:

-lnL = 62.23

dispersal = 3.383

extinction = 1.783

Ancestral range subdivision/inheritance scenarios ('splits') at

internal nodes.

* Split format: [left|right], where 'left' and 'right' are the ranges

inherited by each descendant branch (on the printed tree, 'left' is

the upper branch, and 'right' the lower branch).

* Only splits within 2 log-likelihood units of the maximum for each

node are shown. 'Rel.Prob' is the relative probability (fraction of

the global likelihood) of a split.

At node N22:

split lnL Rel.Prob

[N|N] -66.73 0.01113

[O|O] -66.73 0.01113

[E|E] -66.73 0.01113

[F|F] -66.73 0.01113

[K|K] -66.74 0.0111

[J|J] -66.74 0.0111

[L|L] -66.74 0.0111

[G|G] -66.74 0.01106

[C|C] -66.75 0.01096

[A|A] -66.75 0.01096

[B|B] -66.75 0.01096

[D|D] -66.79 0.01051

[I|I] -66.88 0.009596

[M|M] -67.01 0.008432

[H|H] -68.09 0.002855

[NO|N] -68.48 0.00194

[NO|O] -68.48 0.00194

[EN|N] -68.48 0.00194

[EN|E] -68.48 0.00194

[FN|N] -68.48 0.00194

[FN|F] -68.48 0.00194

[EO|O] -68.48 0.00194

[EO|E] -68.48 0.00194

[FO|O] -68.48 0.00194

[FO|F] -68.48 0.00194

[EF|F] -68.48 0.00194

[EF|E] -68.48 0.00194

[N|FN] -68.48 0.001939

[N|EN] -68.48 0.001939

[N|NO] -68.48 0.001939

[O|FO] -68.48 0.001939

[O|EO] -68.48 0.001939

[O|NO] -68.48 0.001939

[E|EN] -68.48 0.001939

[E|EO] -68.48 0.001939

[E|EF] -68.48 0.001939

[F|FN] -68.48 0.001939

[F|FO] -68.48 0.001939

[F|EF] -68.48 0.001939

[KN|N] -68.48 0.001938

[JN|N] -68.48 0.001938

[LN|N] -68.48 0.001938

[KO|O] -68.48 0.001938

[EK|E] -68.48 0.001938

[FK|F] -68.48 0.001938

[JO|O] -68.48 0.001938

[EJ|E] -68.48 0.001938

[LO|O] -68.48 0.001938

[EL|E] -68.48 0.001938

[FJ|F] -68.48 0.001938

[FL|F] -68.48 0.001938

[N|LN] -68.48 0.001938

[N|KN] -68.48 0.001938

[N|JN] -68.48 0.001938

[O|KO] -68.48 0.001938

[O|LO] -68.48 0.001938

[O|JO] -68.48 0.001938

[E|EK] -68.48 0.001938

[E|EL] -68.48 0.001938

[E|EJ] -68.48 0.001938

[F|FK] -68.48 0.001938

[F|FL] -68.48 0.001938

[F|FJ] -68.48 0.001938

[N|GN] -68.48 0.001937

[O|GO] -68.48 0.001937

[E|EG] -68.48 0.001937

[F|FG] -68.48 0.001937

[GN|N] -68.48 0.001936

[GO|O] -68.48 0.001936

[EG|E] -68.48 0.001936

[FG|F] -68.48 0.001936

[KN|K] -68.48 0.001936

[JN|J] -68.48 0.001936

[LN|L] -68.48 0.001936

[KO|K] -68.48 0.001936

[EK|K] -68.48 0.001936

[FK|K] -68.48 0.001936

[JO|J] -68.48 0.001935

[EJ|J] -68.48 0.001935

[LO|L] -68.48 0.001935

[EL|L] -68.48 0.001935

[FJ|J] -68.48 0.001935

[FL|L] -68.48 0.001935

[K|KN] -68.48 0.001935

[K|FK] -68.48 0.001935

[K|KO] -68.48 0.001935

[K|EK] -68.48 0.001935

[J|JN] -68.48 0.001935

[J|JO] -68.48 0.001935

[J|FJ] -68.48 0.001935

[J|EJ] -68.48 0.001935

[L|LN] -68.48 0.001935

[L|LO] -68.48 0.001935

[L|FL] -68.48 0.001935

[L|EL] -68.48 0.001935

[JK|K] -68.48 0.001934

[JK|J] -68.48 0.001934

[KL|K] -68.48 0.001934

[KL|L] -68.48 0.001934

[JL|L] -68.48 0.001934

[JL|J] -68.48 0.001934

[K|KL] -68.48 0.001934

[K|JK] -68.48 0.001934

[J|JK] -68.48 0.001934

[J|JL] -68.48 0.001934

[L|KL] -68.48 0.001934

[L|JL] -68.48 0.001934

[K|GK] -68.48 0.001933

[J|GJ] -68.48 0.001933

[L|GL] -68.48 0.001933

[N|BN] -68.48 0.001933

[N|AN] -68.48 0.001933

[N|CN] -68.48 0.001933

[O|BO] -68.48 0.001933

[O|CO] -68.48 0.001933

[O|AO] -68.48 0.001933

[E|BE] -68.48 0.001933

[E|AE] -68.48 0.001933

[E|CE] -68.48 0.001933

[F|AF] -68.48 0.001933

[F|BF] -68.48 0.001933

[F|CF] -68.48 0.001933

[GK|K] -68.48 0.001932

[GJ|J] -68.48 0.001932

[GL|L] -68.48 0.001932

[GN|G] -68.48 0.001932

[GO|G] -68.48 0.001932

[EG|G] -68.48 0.001932

[FG|G] -68.48 0.001932

[CN|N] -68.48 0.001931

[AN|N] -68.48 0.001931

[BN|N] -68.48 0.001931

[CO|O] -68.48 0.001931

[CE|E] -68.48 0.001931

[BO|O] -68.48 0.001931

[AO|O] -68.48 0.001931

[AE|E] -68.48 0.001931

[BE|E] -68.48 0.001931

[CF|F] -68.48 0.001931

[BF|F] -68.48 0.001931

[AF|F] -68.48 0.001931

[G|GN] -68.48 0.001931

[G|GO] -68.48 0.001931

[G|FG] -68.48 0.001931

[G|EG] -68.48 0.001931

[GK|G] -68.48 0.00193

[GJ|G] -68.48 0.00193

[GL|G] -68.48 0.00193

[G|GK] -68.49 0.001929

[G|GL] -68.49 0.001929

[G|GJ] -68.49 0.001929

[K|AK] -68.49 0.001929

[K|CK] -68.49 0.001929

[K|BK] -68.49 0.001929

[J|AJ] -68.49 0.001929

[J|BJ] -68.49 0.001929

[J|CJ] -68.49 0.001929

[L|AL] -68.49 0.001929

[L|BL] -68.49 0.001929

[L|CL] -68.49 0.001929

[CK|K] -68.49 0.001927

[BK|K] -68.49 0.001927

[AK|K] -68.49 0.001927

[CJ|J] -68.49 0.001927

[CL|L] -68.49 0.001927

[BJ|J] -68.49 0.001927

[AJ|J] -68.49 0.001927

[BL|L] -68.49 0.001927

[AL|L] -68.49 0.001927

[G|AG] -68.49 0.001925

[G|BG] -68.49 0.001925

[G|CG] -68.49 0.001925

[CG|G] -68.49 0.001923

[BG|G] -68.49 0.001923

[AG|G] -68.49 0.001923

[CN|C] -68.49 0.001918

[AN|A] -68.49 0.001918

[BN|B] -68.49 0.001918

[CO|C] -68.49 0.001918

[CE|C] -68.49 0.001918

[BO|B] -68.49 0.001918

[AO|A] -68.49 0.001918

[AE|A] -68.49 0.001918

[BE|B] -68.49 0.001918

[CF|C] -68.49 0.001918

[BF|B] -68.49 0.001918

[AF|A] -68.49 0.001918

[C|CN] -68.49 0.001917

[C|CO] -68.49 0.001917

[C|CF] -68.49 0.001917

[C|CE] -68.49 0.001917

[B|BN] -68.49 0.001917

[A|AN] -68.49 0.001917

[B|BO] -68.49 0.001917

[A|AF] -68.49 0.001917

[B|BF] -68.49 0.001917

[B|BE] -68.49 0.001917

[A|AE] -68.49 0.001917

[A|AO] -68.49 0.001917

[CK|C] -68.49 0.001917

[BK|B] -68.49 0.001917

[AK|A] -68.49 0.001917

[CJ|C] -68.49 0.001917

[CL|C] -68.49 0.001917

[BJ|B] -68.49 0.001917

[AJ|A] -68.49 0.001917

[BL|B] -68.49 0.001917

[AL|A] -68.49 0.001917

[C|CK] -68.49 0.001915

[C|CL] -68.49 0.001915

[C|CJ] -68.49 0.001915

[A|AK] -68.49 0.001915

[A|AL] -68.49 0.001915

[B|BJ] -68.49 0.001915

[A|AJ] -68.49 0.001915

[B|BL] -68.49 0.001915

[B|BK] -68.49 0.001915

[C|CG] -68.49 0.001915

[A|AG] -68.49 0.001915

[B|BG] -68.49 0.001915

[CG|C] -68.49 0.001915

[AG|A] -68.49 0.001915

[BG|B] -68.49 0.001915

[DN|N] -68.49 0.001913

[DO|O] -68.49 0.001913

[DE|E] -68.49 0.001913

[DF|F] -68.49 0.001913

[N|DN] -68.49 0.001911

[O|DO] -68.49 0.001911

[E|DE] -68.49 0.001911

[C|AC] -68.49 0.001911

[C|BC] -68.49 0.001911

[F|DF] -68.49 0.001911

[B|AB] -68.49 0.001911

[A|AC] -68.49 0.001911

[A|AB] -68.49 0.001911

[B|BC] -68.49 0.001911

[BC|B] -68.5 0.00191

[AC|A] -68.5 0.00191

[BC|C] -68.5 0.00191

[AC|C] -68.5 0.00191

[AB|A] -68.5 0.00191

[AB|B] -68.5 0.00191

[DK|K] -68.5 0.001909

[DJ|J] -68.5 0.001909

[DL|L] -68.5 0.001909

[K|DK] -68.5 0.001907

[J|DJ] -68.5 0.001907

[L|DL] -68.5 0.001907

[DG|G] -68.5 0.001905

[G|DG] -68.5 0.001903

[N|IN] -68.52 0.00187

[O|IO] -68.52 0.00187

[E|EI] -68.52 0.00187

[F|FI] -68.52 0.00187

[IN|N] -68.52 0.00187

[IO|O] -68.52 0.00187

[EI|E] -68.52 0.00187

[FI|F] -68.52 0.00187

[G|GI] -68.52 0.001862

[GI|G] -68.52 0.001862

[DN|D] -68.52 0.001858

[DO|D] -68.52 0.001858

[DE|D] -68.52 0.001858

[DF|D] -68.52 0.001858

[D|DN] -68.52 0.001858

[D|DF] -68.52 0.001858

[D|DE] -68.52 0.001858

[D|DO] -68.52 0.001858

[DK|D] -68.52 0.001857

[DJ|D] -68.52 0.001857

[DL|D] -68.52 0.001857

[D|DK] -68.52 0.001857

[D|DL] -68.52 0.001857

[D|DJ] -68.52 0.001857

[D|DG] -68.52 0.001857

[DG|D] -68.52 0.001855

[N|O] -68.52 0.001855

[N|F] -68.52 0.001855

[N|E] -68.52 0.001855

[O|N] -68.52 0.001855

[O|F] -68.52 0.001855

[O|E] -68.52 0.001855

[E|N] -68.52 0.001855

[E|O] -68.52 0.001855

[E|F] -68.52 0.001855

[F|N] -68.52 0.001855

[F|O] -68.52 0.001855

[F|E] -68.52 0.001855

[K|N] -68.53 0.001852

[K|O] -68.53 0.001852

[K|F] -68.53 0.001852

[K|E] -68.53 0.001852

[J|N] -68.53 0.001852

[J|O] -68.53 0.001852

[J|F] -68.53 0.001852

[J|E] -68.53 0.001852

[L|N] -68.53 0.001852

[L|O] -68.53 0.001852

[L|F] -68.53 0.001852

[L|E] -68.53 0.001852

[N|K] -68.53 0.001852

[N|L] -68.53 0.001852

[N|J] -68.53 0.001852

[O|K] -68.53 0.001852

[O|L] -68.53 0.001852

[O|J] -68.53 0.001852

[E|K] -68.53 0.001852

[E|L] -68.53 0.001852

[E|J] -68.53 0.001852

[F|K] -68.53 0.001852

[F|L] -68.53 0.001852

[F|J] -68.53 0.001852

[N|G] -68.53 0.00185

[O|G] -68.53 0.00185

[E|G] -68.53 0.00185

[F|G] -68.53 0.00185

[K|L] -68.53 0.001849

[K|J] -68.53 0.001849

[J|K] -68.53 0.001849

[J|L] -68.53 0.001849

[L|K] -68.53 0.001849

[L|J] -68.53 0.001849

[C|CI] -68.53 0.001849

[B|BI] -68.53 0.001849

[A|AI] -68.53 0.001849

[CI|C] -68.53 0.001849

[BI|B] -68.53 0.001849

[AI|A] -68.53 0.001849

[G|N] -68.53 0.001848

[G|O] -68.53 0.001848

[G|F] -68.53 0.001848

[G|E] -68.53 0.001848

[K|G] -68.53 0.001848

[J|G] -68.53 0.001848

[L|G] -68.53 0.001848

[G|K] -68.53 0.001846

[G|L] -68.53 0.001846

[G|J] -68.53 0.001846

[N|A] -68.53 0.001842

[N|B] -68.53 0.001842

[N|C] -68.53 0.001842

[O|A] -68.53 0.001842

[O|B] -68.53 0.001842

[O|C] -68.53 0.001842

[E|A] -68.53 0.001842

[E|B] -68.53 0.001842

[E|C] -68.53 0.001842

[F|A] -68.53 0.001842

[F|B] -68.53 0.001842

[F|C] -68.53 0.001842

[K|A] -68.53 0.001839

[K|B] -68.53 0.001839

[K|C] -68.53 0.001839

[J|A] -68.53 0.001839

[J|B] -68.53 0.001839

[J|C] -68.53 0.001839

[L|A] -68.53 0.001839

[L|B] -68.53 0.001839

[L|C] -68.53 0.001839

[C|N] -68.53 0.001839

[C|O] -68.53 0.001839

[C|F] -68.53 0.001839

[C|E] -68.53 0.001839

[B|N] -68.53 0.001839

[A|N] -68.53 0.001839

[B|O] -68.53 0.001839

[A|O] -68.53 0.001839

[B|F] -68.53 0.001839

[B|E] -68.53 0.001839

[A|F] -68.53 0.001839

[A|E] -68.53 0.001839

[C|K] -68.53 0.001837

[C|L] -68.53 0.001837

[C|J] -68.53 0.001837

[B|K] -68.53 0.001837

[A|K] -68.53 0.001837

[B|L] -68.53 0.001837

[B|J] -68.53 0.001837

[A|L] -68.53 0.001837

[A|J] -68.53 0.001837

[G|A] -68.53 0.001836

[G|B] -68.53 0.001836

[G|C] -68.53 0.001836

[C|G] -68.54 0.001835

[B|G] -68.54 0.001835

[A|G] -68.54 0.001835

[C|A] -68.54 0.001826

[C|B] -68.54 0.001826

[B|A] -68.54 0.001826

[B|C] -68.54 0.001826

[A|B] -68.54 0.001826

[A|C] -68.54 0.001826

[G|GM] -68.55 0.001809

[GM|G] -68.55 0.001804

[D|N] -68.55 0.001804

[D|O] -68.55 0.001804

[D|F] -68.55 0.001804

[D|E] -68.55 0.001804

[N|D] -68.55 0.001802

[O|D] -68.55 0.001802

[E|D] -68.55 0.001802

[F|D] -68.55 0.001802

[D|K] -68.55 0.001801

[D|L] -68.55 0.001801

[D|J] -68.55 0.001801

[D|G] -68.55 0.001799

[K|D] -68.55 0.001799

[J|D] -68.55 0.001799

[L|D] -68.55 0.001799

[C|CM] -68.56 0.001796

[B|BM] -68.56 0.001796

[A|AM] -68.56 0.001796

[G|D] -68.56 0.001796

[CM|C] -68.56 0.001791

[BM|B] -68.56 0.001791

[AM|A] -68.56 0.001791

[IN|I] -68.59 0.001737

[IO|I] -68.59 0.001737

[EI|I] -68.59 0.001737

[FI|I] -68.59 0.001737

[I|IN] -68.59 0.001736

[I|EI] -68.59 0.001736

[I|IO] -68.59 0.001736

[I|FI] -68.59 0.001736

[I|GI] -68.59 0.001735

[GI|I] -68.59 0.001734

[I|AI] -68.59 0.001731

[I|CI] -68.59 0.001731

[I|BI] -68.59 0.001731

[CI|I] -68.59 0.00173

[BI|I] -68.59 0.00173

[AI|I] -68.59 0.00173

[N|I] -68.6 0.001723

[O|I] -68.6 0.001723

[E|I] -68.6 0.001723

[F|I] -68.6 0.001723

[I|N] -68.6 0.001721

[I|O] -68.6 0.001721

At node N20:

split lnL Rel.Prob

[N|N] -67.31 0.006228

[O|O] -67.32 0.00619

[E|E] -67.32 0.006186

[F|F] -67.32 0.006176

[G|G] -67.34 0.006092

[K|K] -67.34 0.006049

[J|J] -67.35 0.006019

[L|L] -67.35 0.006012

[C|C] -67.36 0.005956

[A|A] -67.36 0.005947

[B|B] -67.36 0.005947

[D|D] -67.42 0.005609

[I|I] -67.46 0.005387

[M|M] -67.8 0.003824

[NO|O] -68.37 0.002164

[NO|N] -68.37 0.002164

[EN|E] -68.37 0.002163

[EN|N] -68.37 0.002163

[FN|F] -68.37 0.002159

[FN|N] -68.37 0.002159

[N|GN] -68.37 0.002157

[N|KN] -68.38 0.002153

[N|EN] -68.38 0.00215

[N|NO] -68.38 0.00215

[N|FN] -68.38 0.00215

[EO|E] -68.38 0.002149

[EO|O] -68.38 0.002149

[FO|O] -68.38 0.002145

[FO|F] -68.38 0.002145

[EF|E] -68.38 0.002144

[EF|F] -68.38 0.002144

[O|GO] -68.38 0.002144

[E|EG] -68.38 0.002142

[N|JN] -68.38 0.002142

[GN|G] -68.38 0.002141

[O|KO] -68.38 0.00214

[N|LN] -68.38 0.002139

[E|EK] -68.38 0.002139

[F|FG] -68.38 0.002139

[O|EO] -68.38 0.002137

[O|NO] -68.38 0.002137

[O|FO] -68.38 0.002137

[GN|N] -68.38 0.002137

[E|EO] -68.38 0.002136

[E|EN] -68.38 0.002136

[E|EF] -68.38 0.002136

[F|FK] -68.38 0.002135

[F|EF] -68.39 0.002132

[F|FN] -68.39 0.002132

[F|FO] -68.39 0.002132

[O|JO] -68.39 0.002129

[N|AN] -68.39 0.002129

[N|CN] -68.39 0.002129

[N|BN] -68.39 0.002129

[E|EJ] -68.39 0.002128

[GO|G] -68.39 0.002127

[O|LO] -68.39 0.002126

[JN|N] -68.39 0.002126

[LN|N] -68.39 0.002126

[KN|N] -68.39 0.002126

[CN|N] -68.39 0.002126

[EG|G] -68.39 0.002126

[E|EL] -68.39 0.002125

[F|FJ] -68.39 0.002124

[KN|K] -68.39 0.002124

[GO|O] -68.39 0.002123

[AN|N] -68.39 0.002123

[BN|N] -68.39 0.002123

[EG|E] -68.39 0.002122

[FG|G] -68.39 0.002122

[F|FL] -68.39 0.002121

[FG|F] -68.39 0.002118

[G|GK] -68.39 0.002116

[O|BO] -68.39 0.002116

[O|CO] -68.39 0.002116

[O|AO] -68.39 0.002116

[E|AE] -68.39 0.002114

[E|BE] -68.39 0.002114

[E|CE] -68.39 0.002114

[JN|J] -68.39 0.002113

[KO|O] -68.39 0.002113

[LO|O] -68.39 0.002113

[JO|O] -68.39 0.002113

[G|EG] -68.39 0.002113

[G|FG] -68.39 0.002113

[G|GO] -68.39 0.002113

[G|GN] -68.39 0.002113

[CO|O] -68.39 0.002112

[EL|E] -68.4 0.002111

[EK|E] -68.4 0.002111

[EJ|E] -68.4 0.002111

[CE|E] -68.4 0.002111

[LN|L] -68.4 0.002111

[F|BF] -68.4 0.002111

[F|AF] -68.4 0.002111

[F|CF] -68.4 0.002111

[KO|K] -68.4 0.00211

[BO|O] -68.4 0.002109

[AO|O] -68.4 0.002109

[EK|K] -68.4 0.002109

[AE|E] -68.4 0.002108

[BE|E] -68.4 0.002108

[FJ|F] -68.4 0.002108

[FK|F] -68.4 0.002108

[FL|F] -68.4 0.002108

[CF|F] -68.4 0.002107

[FK|K] -68.4 0.002105

[G|GJ] -68.4 0.002105

[AF|F] -68.4 0.002104

[BF|F] -68.4 0.002104

[K|GK] -68.4 0.002104

[CN|C] -68.4 0.002103

[G|GL] -68.4 0.002102

[AN|A] -68.4 0.0021

[BN|B] -68.4 0.0021

[JO|J] -68.4 0.0021

[EJ|J] -68.4 0.002099

[LO|L] -68.4 0.002097

[K|KN] -68.4 0.002097

[K|EK] -68.4 0.002097

[K|KO] -68.4 0.002097

[K|FK] -68.4 0.002097

[DN|N] -68.4 0.002096

[EL|L] -68.4 0.002096

[FJ|J] -68.4 0.002095

[J|GJ] -68.4 0.002093

[FL|L] -68.4 0.002092

[G|AG] -68.4 0.002092

[G|BG] -68.4 0.002092

[G|CG] -68.4 0.002092

[L|GL] -68.41 0.00209

[CO|C] -68.41 0.00209

[GL|G] -68.41 0.002089

[GK|G] -68.41 0.002089

[GJ|G] -68.41 0.002089

[J|JK] -68.41 0.002089

[K|JK] -68.41 0.002089

[CG|G] -68.41 0.002089

[CE|C] -68.41 0.002089

[BO|B] -68.41 0.002087

[AO|A] -68.41 0.002087

[K|KL] -68.41 0.002086

[L|KL] -68.41 0.002086

[AG|G] -68.41 0.002086

[BG|G] -68.41 0.002086

[J|EJ] -68.41 0.002086

[J|FJ] -68.41 0.002086

[J|JO] -68.41 0.002086

[J|JN] -68.41 0.002086

[AE|A] -68.41 0.002085

[BE|B] -68.41 0.002085

[CF|C] -68.41 0.002085

[IN|N] -68.41 0.002084

[GK|K] -68.41 0.002083

[L|EL] -68.41 0.002083

[L|LO] -68.41 0.002083

[L|LN] -68.41 0.002083

[L|FL] -68.41 0.002083

[DO|O] -68.41 0.002083

[AF|A] -68.41 0.002082

[BF|B] -68.41 0.002082

[DE|E] -68.41 0.002082

[C|CG] -68.41 0.00208

[N|DN] -68.41 0.002078

[DF|F] -68.41 0.002078

[A|AG] -68.41 0.002077

[B|BG] -68.41 0.002077

[C|CK] -68.41 0.002077

[K|AK] -68.41 0.002076

[K|BK] -68.41 0.002076

[K|CK] -68.41 0.002076

[J|JL] -68.41 0.002075

[L|JL] -68.41 0.002075

[A|AK] -68.41 0.002074

[B|BK] -68.41 0.002074

[C|CO] -68.41 0.002074

[C|CN] -68.41 0.002074

[C|CF] -68.41 0.002074

[C|CE] -68.41 0.002074

[GJ|J] -68.41 0.002073

[JK|K] -68.41 0.002073

[KL|K] -68.41 0.002073

[CK|K] -68.41 0.002073

[A|AN] -68.41 0.002071

[A|AE] -68.41 0.002071

[B|BE] -68.41 0.002071

[A|AF] -68.41 0.002071

[B|BO] -68.41 0.002071

[B|BN] -68.41 0.002071

[B|BF] -68.41 0.002071

[A|AO] -68.41 0.002071

[IO|O] -68.41 0.002071

[GL|L] -68.41 0.00207

[AK|K] -68.41 0.00207

[BK|K] -68.41 0.00207

[EI|E] -68.42 0.002069

[C|CJ] -68.42 0.002066

[FI|F] -68.42 0.002065

[O|DO] -68.42 0.002065

[J|AJ] -68.42 0.002065

[J|BJ] -68.42 0.002065

[J|CJ] -68.42 0.002065

[E|DE] -68.42 0.002064

[C|CL] -68.42 0.002063

[A|AJ] -68.42 0.002063

[B|BJ] -68.42 0.002063

[CG|C] -68.42 0.002063

[N|IN] -68.42 0.002063

[JK|J] -68.42 0.002062

[JL|J] -68.42 0.002062

[L|CL] -68.42 0.002062

[L|AL] -68.42 0.002062

[L|BL] -68.42 0.002062

[CJ|J] -68.42 0.002062

[A|AL] -68.42 0.002061

[B|BL] -68.42 0.002061

[F|DF] -68.42 0.00206

[AG|A] -68.42 0.00206

[BG|B] -68.42 0.00206

[N|G] -68.42 0.00206

[JL|L] -68.42 0.00206

[KL|L] -68.42 0.00206

[CL|L] -68.42 0.00206

[DG|G] -68.42 0.00206

[N|E] -68.42 0.002059

[N|O] -68.42 0.002059

[N|F] -68.42 0.002059

[AJ|J] -68.42 0.002059

[BJ|J] -68.42 0.002059

[AL|L] -68.42 0.002057

[BL|L] -68.42 0.002057

[N|K] -68.42 0.002056

[C|AC] -68.42 0.002053

[C|BC] -68.42 0.002053

[CJ|C] -68.42 0.002053

[CK|C] -68.42 0.002053

[CL|C] -68.42 0.002053

[O|IO] -68.42 0.00205

[A|AC] -68.42 0.00205

[A|AB] -68.42 0.00205

[B|AB] -68.42 0.00205

[B|BC] -68.42 0.00205

[AJ|A] -68.42 0.00205

[AK|A] -68.42 0.00205

[AL|A] -68.42 0.00205

[BK|B] -68.42 0.00205

[BJ|B] -68.42 0.00205

[BL|B] -68.42 0.00205

[AC|A] -68.42 0.002049

[BC|B] -68.42 0.002049

[BC|C] -68.42 0.002049

[AC|C] -68.42 0.002049

[E|EI] -68.42 0.002049

[GI|G] -68.43 0.002047

[O|G] -68.43 0.002047

[O|E] -68.43 0.002047

[O|F] -68.43 0.002047

[O|N] -68.43 0.002047

[AB|A] -68.43 0.002046

[AB|B] -68.43 0.002046

[E|G] -68.43 0.002046

[E|F] -68.43 0.002046

[E|O] -68.43 0.002046

[E|N] -68.43 0.002046

[F|FI] -68.43 0.002046

[N|J] -68.43 0.002045

[DK|K] -68.43 0.002043

[O|K] -68.43 0.002043

[N|L] -68.43 0.002043

[F|G] -68.43 0.002043

[F|E] -68.43 0.002042

[F|O] -68.43 0.002042

[F|N] -68.43 0.002042

[E|K] -68.43 0.002042

[G|DG] -68.43 0.002042

[F|K] -68.43 0.002038

[DJ|J] -68.43 0.002033

[O|J] -68.43 0.002033

[E|J] -68.43 0.002032

[DL|L] -68.43 0.002031

[O|L] -68.43 0.00203

[N|A] -68.43 0.00203

[N|C] -68.43 0.00203

[N|B] -68.43 0.00203

[E|L] -68.43 0.002029

[F|J] -68.44 0.002028

[G|GI] -68.44 0.002027

[K|DK] -68.44 0.002026

[F|L] -68.44 0.002026

[DN|D] -68.44 0.002022

[O|B] -68.44 0.002017

[O|A] -68.44 0.002017

[O|C] -68.44 0.002017

[E|A] -68.44 0.002016

[E|B] -68.44 0.002016

[E|C] -68.44 0.002016

[J|DJ] -68.44 0.002015

[G|E] -68.44 0.002014

[G|F] -68.44 0.002014

[G|O] -68.44 0.002014

[G|N] -68.44 0.002014

[L|DL] -68.44 0.002013

[F|A] -68.44 0.002012

[F|B] -68.44 0.002012

[F|C] -68.44 0.002012

[CI|C] -68.44 0.002012

[G|K] -68.44 0.002011

[IN|I] -68.44 0.00201

[DO|D] -68.44 0.002009

[AI|A] -68.44 0.002009

[BI|B] -68.44 0.002009

[DE|D] -68.45 0.002008

[J|G] -68.45 0.002004

[L|G] -68.45 0.002004

[K|G] -68.45 0.002004

[DF|D] -68.45 0.002004

[J|E] -68.45 0.002004

[J|F] -68.45 0.002004

[K|E] -68.45 0.002004

[J|O] -68.45 0.002004

[J|N] -68.45 0.002004

[K|N] -68.45 0.002004

[L|E] -68.45 0.002004

[K|O] -68.45 0.002004

[K|F] -68.45 0.002004

[L|O] -68.45 0.002004

[L|N] -68.45 0.002004

[L|F] -68.45 0.002004

[D|DG] -68.45 0.002002

[G|J] -68.45 0.002001

[J|K] -68.45 0.002

[L|K] -68.45 0.002

[C|G] -68.45 0.001999

[C|O] -68.45 0.001999

[C|E] -68.45 0.001999

[C|N] -68.45 0.001999

[C|F] -68.45 0.001999

[D|DK] -68.45 0.001998

[G|L] -68.45 0.001998

[IO|I] -68.45 0.001997

[I|GI] -68.45 0.001997

[A|G] -68.45 0.001996

[B|G] -68.45 0.001996

[EI|I] -68.45 0.001996

[A|E] -68.45 0.001996

[B|E] -68.45 0.001996

[A|N] -68.45 0.001996

[A|F] -68.45 0.001996

[B|O] -68.45 0.001996

[B|F] -68.45 0.001996

[B|N] -68.45 0.001996

[A|O] -68.45 0.001996

[D|DO] -68.45 0.001995

[D|DF] -68.45 0.001995

[D|DE] -68.45 0.001995

[D|DN] -68.45 0.001995

[C|K] -68.45 0.001995

[FI|I] -68.45 0.001992

[A|K] -68.45 0.001992

[B|K] -68.45 0.001992

[I|EI] -68.45 0.00199

[I|IN] -68.45 0.00199

[I|FI] -68.45 0.00199

[I|IO] -68.45 0.00199

[K|J] -68.45 0.00199

[L|J] -68.45 0.00199

[C|CI] -68.45 0.001989

[D|DJ] -68.46 0.001988

[J|L] -68.46 0.001988

[K|L] -68.46 0.001988

[A|AI] -68.46 0.001987

[B|BI] -68.46 0.001987

[D|DL] -68.46 0.001985

[C|J] -68.46 0.001985

[G|A] -68.46 0.001985

[G|C] -68.46 0.001985

[G|B] -68.46 0.001985

[DG|D] -68.46 0.001983

[C|L] -68.46 0.001983

[A|J] -68.46 0.001982

[B|J] -68.46 0.001982

[A|L] -68.46 0.00198

[B|L] -68.46 0.00198

[J|A] -68.46 0.001975

[K|B] -68.46 0.001975

[J|B] -68.46 0.001975

[K|A] -68.46 0.001975

[J|C] -68.46 0.001975

[K|C] -68.46 0.001975

[L|A] -68.46 0.001975

[L|B] -68.46 0.001975

[L|C] -68.46 0.001975

[DJ|D] -68.46 0.001973

[DK|D] -68.46 0.001973

[DL|D] -68.46 0.001973

[GI|I] -68.46 0.001972

[I|BI] -68.46 0.00197

[I|CI] -68.46 0.00197

[I|AI] -68.46 0.00197

[C|B] -68.46 0.00197

[C|A] -68.46 0.00197

[B|A] -68.47 0.001967

[A|B] -68.47 0.001967

[B|C] -68.47 0.001967

[A|C] -68.47 0.001967

[CI|I] -68.47 0.001961

[N|D] -68.47 0.001959

[BI|I] -68.47 0.001958

[AI|I] -68.47 0.001958

[D|G] -68.47 0.001951

[D|O] -68.47 0.00195

[D|E] -68.47 0.00195

[D|N] -68.47 0.00195

[D|F] -68.47 0.00195

[O|D] -68.48 0.001947

[D|K] -68.48 0.001947

[E|D] -68.48 0.001946

[F|D] -68.48 0.001943

[D|J] -68.48 0.001937

[D|L] -68.48 0.001935

[I|G] -68.49 0.001918

[I|E] -68.49 0.001917

[I|F] -68.49 0.001917

[I|O] -68.49 0.001917

[I|N] -68.49 0.001917

[N|I] -68.49 0.001917

[G|D] -68.49 0.001916

[K|D] -68.5 0.001906

[J|D] -68.5 0.001906

[L|D] -68.5 0.001906

[O|I] -68.5 0.001905

[E|I] -68.5 0.001904

[F|I] -68.5 0.001901

[I|B] -68.51 0.001889

[I|C] -68.51 0.001889

[I|A] -68.51 0.001889

[G|I] -68.51 0.001875

[C|I] -68.52 0.00186

At node N12:

split lnL Rel.Prob

[I|I] -66.7 0.01148

[N|N] -66.85 0.009856

[N|I] -66.95 0.008932

[N|IN] -66.98 0.00867

[O|O] -67.16 0.007244

[IN|I] -67.17 0.007168

[E|E] -67.2 0.006991

[O|I] -67.26 0.006565

[D|D] -67.29 0.006405

[O|IO] -67.29 0.006372

[E|I] -67.3 0.006336

[DN|N] -67.31 0.006266

[F|F] -67.31 0.006258

[E|EI] -67.33 0.00615

[F|I] -67.41 0.005671

[F|FI] -67.44 0.005505

[C|C] -67.49 0.005212

[IO|I] -67.51 0.005119

[G|G] -67.52 0.005082

[EI|I] -67.55 0.004928

[G|I] -67.57 0.004815

[C|I] -67.59 0.004724

[A|A] -67.61 0.004636

[B|B] -67.61 0.004636

[G|GI] -67.61 0.004632

[C|CI] -67.62 0.004588

[FI|I] -67.67 0.004362

[DO|O] -67.68 0.004295

[I|HI] -67.7 0.004233

[A|I] -67.71 0.004202

[B|I] -67.71 0.004202

[DE|E] -67.72 0.004144

[A|AI] -67.74 0.004081

[B|BI] -67.74 0.004081

[CI|I] -67.8 0.00384

[I|FI] -67.8 0.003828

[I|EI] -67.8 0.003828

[I|IO] -67.8 0.003828

[I|IN] -67.8 0.003828

[I|AI] -67.8 0.003813

[I|CI] -67.8 0.003813

[I|BI] -67.8 0.003813

[D|F] -67.8 0.003812

[D|E] -67.8 0.003812

[D|O] -67.8 0.003812

[D|N] -67.8 0.003812

[I|GI] -67.81 0.003787

[NO|O] -67.82 0.003765

[NO|N] -67.82 0.003765

[GI|I] -67.83 0.003732

[K|K] -67.83 0.003705

[J|J] -67.83 0.003705

[L|L] -67.83 0.003705

[DF|F] -67.84 0.003663

[D|G] -67.85 0.003644

[EN|E] -67.87 0.003564

[EN|N] -67.87 0.003564

[DN|D] -67.88 0.003531

[AI|I] -67.92 0.003408

[BI|I] -67.92 0.003408

[I|H] -67.94 0.003324

[N|F] -67.96 0.00326

[N|E] -67.96 0.00326

[N|O] -67.96 0.00326

[N|A] -67.96 0.003258

[N|B] -67.96 0.003258

[N|C] -67.96 0.003258

[FN|F] -67.98 0.003203

[FN|N] -67.98 0.003203

[N|FN] -67.98 0.003186

[N|EN] -67.98 0.003186

[N|NO] -67.98 0.003186

[N|CN] -67.99 0.003176

[N|AN] -67.99 0.003176

[N|BN] -67.99 0.003176

[CN|C] -68 0.003132

[CN|N] -68 0.00312

[N|G] -68.01 0.003117

[N|GN] -68.01 0.0031

[DG|G] -68.03 0.003056

[KN|N] -68.04 0.003021

[JN|N] -68.04 0.003021

[LN|N] -68.04 0.003021

[GN|N] -68.08 0.002888

[GN|G] -68.12 0.002767

[AN|A] -68.13 0.002756

[BN|B] -68.13 0.002756

[AN|N] -68.13 0.002746

[BN|N] -68.13 0.002746

[D|DF] -68.15 0.002695

[D|DO] -68.15 0.002695

[D|DE] -68.15 0.002695

[D|DN] -68.15 0.002695

[D|DG] -68.19 0.002595

[EO|E] -68.19 0.002586

[EO|O] -68.19 0.002586

[IN|N] -68.22 0.002519

[DO|D] -68.26 0.002421

[N|KN] -68.27 0.002397

[N|JN] -68.27 0.002397

[N|LN] -68.27 0.002397

[O|F] -68.27 0.002396

[O|E] -68.27 0.002396

[O|N] -68.27 0.002396

[O|A] -68.27 0.002394

[O|B] -68.27 0.002394

[O|C] -68.27 0.002394

[D|K] -68.27 0.002386

[D|J] -68.27 0.002386

[D|L] -68.27 0.002386

[FO|F] -68.28 0.002378

[FO|O] -68.28 0.002378

[O|FO] -68.29 0.002342

[O|EO] -68.29 0.002342

[O|NO] -68.29 0.002342

[DE|D] -68.29 0.002336

[O|BO] -68.29 0.002335

[O|AO] -68.29 0.002335

[O|CO] -68.29 0.002335

[E|F] -68.3 0.002312

[E|N] -68.3 0.002312

[E|O] -68.3 0.002312

[E|A] -68.3 0.002311

[E|B] -68.3 0.002311

[E|C] -68.3 0.002311

[EF|F] -68.31 0.002305

[EF|E] -68.31 0.002305

[O|G] -68.31 0.002291

[O|GO] -68.32 0.002278

[N|DN] -68.32 0.002274

[E|EN] -68.33 0.00226

[E|EF] -68.33 0.00226

[E|EO] -68.33 0.00226

[E|BE] -68.33 0.002253

[E|AE] -68.33 0.002253

[E|CE] -68.33 0.002253

[CO|C] -68.35 0.002211

[E|G] -68.35 0.002211

[CO|O] -68.35 0.002203

[E|EG] -68.35 0.002199

[KO|O] -68.36 0.002193

[JO|O] -68.36 0.002193

[LO|O] -68.36 0.002193

[CE|C] -68.38 0.002144

[CE|E] -68.38 0.002136

[EK|E] -68.39 0.002118

[EJ|E] -68.39 0.002118

[EL|E] -68.39 0.002118

[DK|K] -68.41 0.002076

[DJ|J] -68.41 0.002076

[DL|L] -68.41 0.002076

[GO|O] -68.41 0.002074

[F|E] -68.41 0.00207

[F|O] -68.41 0.00207

[F|N] -68.41 0.00207

[F|A] -68.42 0.002068

[F|C] -68.42 0.002068

[F|B] -68.42 0.002068

[DF|D] -68.42 0.002064

[N|K] -68.43 0.002041

[N|J] -68.43 0.00204

[N|L] -68.43 0.00204

[F|FN] -68.44 0.002023

[F|FO] -68.44 0.002023

[F|EF] -68.44 0.002023

[F|BF] -68.44 0.002017

[F|CF] -68.44 0.002017

[F|AF] -68.44 0.002017

[EG|E] -68.45 0.002

[GO|G] -68.46 0.001987

[F|G] -68.46 0.001979

[AO|A] -68.46 0.001978

[BO|B] -68.46 0.001978

[BO|O] -68.46 0.00197

[AO|O] -68.46 0.00197

[F|FG] -68.47 0.001968

[K|F] -68.47 0.001958

[K|E] -68.47 0.001958

[K|O] -68.47 0.001958

[K|N] -68.47 0.001958

[J|F] -68.47 0.001958

[J|E] -68.47 0.001958

[J|O] -68.47 0.001958

[J|N] -68.47 0.001958

[L|F] -68.47 0.001958

[L|E] -68.47 0.001958

[L|O] -68.47 0.001958

[L|N] -68.47 0.001958

[K|A] -68.47 0.001957

[K|C] -68.47 0.001957

[K|B] -68.47 0.001957

[J|A] -68.47 0.001957

[J|C] -68.47 0.001957

[J|B] -68.47 0.001957

[L|A] -68.47 0.001957

[L|C] -68.47 0.001957

[L|B] -68.47 0.001957

[EG|G] -68.49 0.001916

[AE|A] -68.5 0.001909

[BE|B] -68.5 0.001909

[AE|E] -68.5 0.001901

[BE|E] -68.5 0.001901

[FK|F] -68.5 0.001893

[FJ|F] -68.5 0.001893

[FL|F] -68.5 0.001893

[KN|K] -68.5 0.001892

[JN|J] -68.5 0.001892

[LN|L] -68.5 0.001892

[CF|C] -68.51 0.001876

[K|G] -68.52 0.001872

[J|G] -68.52 0.001872

[L|G] -68.52 0.001872

[CF|F] -68.52 0.001869

[DK|D] -68.52 0.001868

[DJ|D] -68.52 0.001868

[DL|D] -68.52 0.001868

[N|D] -68.55 0.001812

[IO|O] -68.56 0.001798

[DG|D] -68.56 0.001798

[FG|F] -68.57 0.001779

[D|DK] -68.57 0.001772

[D|DJ] -68.57 0.001772

[D|DL] -68.57 0.001772

[H|H] -68.58 0.001762

[O|KO] -68.58 0.001761

[O|JO] -68.58 0.001761

[O|LO] -68.58 0.001761

[G|F] -68.58 0.001758

[G|E] -68.58 0.001758

[G|O] -68.58 0.001758

[G|N] -68.58 0.001758

[G|C] -68.58 0.001756

[G|A] -68.58 0.001756

[G|B] -68.58 0.001756

[CK|C] -68.59 0.001744

[CJ|C] -68.59 0.001744

[CL|C] -68.59 0.001744

[EI|E] -68.59 0.001731

[C|F] -68.6 0.001724

[C|E] -68.6 0.001724

[C|O] -68.6 0.001724

[C|N] -68.6 0.001724

[C|A] -68.6 0.001723

[C|B] -68.6 0.001723

[FG|G] -68.61 0.001704

[E|EK] -68.61 0.0017

[E|EJ] -68.61 0.0017

[E|EL] -68.61 0.0017

[C|CN] -68.62 0.001688

[C|CO] -68.62 0.001688

[C|CF] -68.62 0.001688

[C|CE] -68.62 0.001688

[C|AC] -68.62 0.001683

[C|BC] -68.62 0.001683

[G|FG] -68.63 0.001674

[G|EG] -68.63 0.001674

[G|GN] -68.63 0.001674

[G|GO] -68.63 0.001674

[AF|A] -68.63 0.001673

[BF|B] -68.63 0.001673

[O|DO] -68.63 0.001671

[G|CG] -68.63 0.001669

[G|AG] -68.63 0.001669

[G|BG] -68.63 0.001669

[BF|F] -68.63 0.001667

[AF|F] -68.63 0.001667

[C|G] -68.64 0.001649

[C|CG] -68.65 0.001642

[CG|C] -68.66 0.001625

[HI|H] -68.66 0.001621

[E|DE] -68.66 0.001613

[GK|G] -68.69 0.001578

[GJ|G] -68.69 0.001578

[GL|G] -68.69 0.001578

[AK|A] -68.7 0.001553

[BK|B] -68.7 0.001553

[AJ|A] -68.7 0.001553

[BJ|B] -68.7 0.001553

[AL|A] -68.7 0.001553

[BL|B] -68.7 0.001553

[CG|G] -68.7 0.001551

[A|F] -68.71 0.001534

[A|E] -68.71 0.001534

[B|F] -68.71 0.001534

[A|O] -68.71 0.001534

[B|E] -68.71 0.001534

[A|N] -68.71 0.001534

[B|O] -68.71 0.001534

[B|N] -68.71 0.001534

[A|B] -68.72 0.001533

[A|C] -68.72 0.001533

[B|A] -68.72 0.001533

[B|C] -68.72 0.001533

[FI|F] -68.72 0.001533

[F|FK] -68.72 0.001522

[F|FJ] -68.72 0.001522

[F|FL] -68.72 0.001522

[A|AN] -68.74 0.001501

[A|AO] -68.74 0.001501

[B|BO] -68.74 0.001501

[A|AE] -68.74 0.001501

[B|BN] -68.74 0.001501

[A|AF] -68.74 0.001501

[B|BF] -68.74 0.001501

[B|BE] -68.74 0.001501

[O|K] -68.74 0.0015

[O|J] -68.74 0.0015

[O|L] -68.74 0.0015

[A|AC] -68.74 0.001497

[A|AB] -68.74 0.001497

[B|AB] -68.74 0.001497

[B|BC] -68.74 0.001497

[AC|A] -68.76 0.00147

[BC|B] -68.76 0.00147

[BC|C] -68.76 0.00147

[AC|C] -68.76 0.00147

[A|G] -68.76 0.001467

[B|G] -68.76 0.001467

[A|AG] -68.76 0.001461

[B|BG] -68.76 0.001461

[E|K] -68.77 0.001447

[E|J] -68.77 0.001447

[E|L] -68.77 0.001447

[F|DF] -68.78 0.001444

[K|FK] -68.78 0.00144

[K|KN] -68.78 0.00144

[K|KO] -68.78 0.00144

[K|EK] -68.78 0.00144

[J|FJ] -68.78 0.00144

[J|JO] -68.78 0.00144

[J|JN] -68.78 0.00144

[J|EJ] -68.78 0.00144

[L|FL] -68.78 0.00144

[L|EL] -68.78 0.00144

[L|LN] -68.78 0.00144

[L|LO] -68.78 0.00144

[AG|A] -68.78 0.001439

[BG|B] -68.78 0.001439

[K|BK] -68.78 0.001436

[K|AK] -68.78 0.001436

[K|CK] -68.78 0.001436

[J|AJ] -68.78 0.001436

[J|BJ] -68.78 0.001436

[J|CJ] -68.78 0.001436

[L|AL] -68.78 0.001436

[L|BL] -68.78 0.001436

[L|CL] -68.78 0.001436

[HI|I] -68.79 0.001423

[M|M] -68.79 0.00142

[K|GK] -68.81 0.001389

[J|GJ] -68.81 0.001389

[L|GL] -68.81 0.001389

[I|F] -68.82 0.001386

[I|E] -68.82 0.001386

[I|O] -68.82 0.001386

[I|N] -68.82 0.001386

[I|A] -68.82 0.001385

[I|B] -68.82 0.001385

[I|C] -68.82 0.001385

[AG|G] -68.82 0.001374

[BG|G] -68.82 0.001374

[KO|K] -68.83 0.001373

[JO|J] -68.83 0.001373

[LO|L] -68.83 0.001373

[CI|C] -68.84 0.001354

[O|D] -68.86 0.001332

[EK|K] -68.86 0.001326

[EJ|J] -68.86 0.001326

[EL|L] -68.86 0.001326

[I|G] -68.86 0.001325

[AB|A] -68.87 0.001309

[AB|B] -68.87 0.001309

[F|K] -68.88 0.001296

[F|J] -68.88 0.001296

[F|L] -68.88 0.001296

[E|D] -68.89 0.001286

[C|CK] -68.9 0.00127

[C|CJ] -68.9 0.00127

[C|CL] -68.9 0.00127

[GI|G] -68.91 0.001256

[G|GK] -68.92 0.001249

[G|GJ] -68.92 0.001249

[G|GL] -68.92 0.001249

[J|K] -68.94 0.001226

[K|J] -68.94 0.001226

[K|L] -68.94 0.001226

[L|K] -68.94 0.001226

[J|L] -68.94 0.001226

[L|J] -68.94 0.001226

[AI|A] -68.96 0.001202

[BI|B] -68.96 0.001202

[C|CM] -68.97 0.001193

[FK|K] -68.97 0.001186

[FJ|J] -68.97 0.001186

[FL|L] -68.97 0.001186

[G|DG] -68.97 0.001183

[G|GM] -68.98 0.001174

[F|D] -69 0.001151

[A|AK] -69.02 0.00113

[B|BK] -69.02 0.00113

[A|AJ] -69.02 0.00113

[B|BJ] -69.02 0.00113

[A|AL] -69.02 0.00113

[B|BL] -69.02 0.00113

[JK|K] -69.05 0.001102

[JK|J] -69.05 0.001102

[KL|L] -69.05 0.001102

[KL|K] -69.05 0.001102

[JL|L] -69.05 0.001102

[JL|J] -69.05 0.001102

[G|K] -69.05 0.0011

[G|J] -69.05 0.0011

[G|L] -69.05 0.0011

[K|D] -69.06 0.001089

[J|D] -69.06 0.001089

[L|D] -69.06 0.001089

[CK|K] -69.06 0.001088

At node N10:

split lnL Rel.Prob

[N|D] -64.63 0.0909

[N|DN] -64.72 0.08368

[O|D] -65.61 0.03409

[O|DO] -65.7 0.03138

[E|D] -65.75 0.02966

[N|N] -65.79 0.02853

[CN|N] -65.81 0.02789

[E|DE] -65.84 0.02731

[DN|D] -66.07 0.02167

[NO|O] -66.11 0.02067

[NO|N] -66.11 0.02067

[F|D] -66.46 0.01465

[EN|N] -66.52 0.01377

[EN|E] -66.52 0.01377

[F|DF] -66.54 0.01349

[O|O] -66.77 0.0107

[N|K] -66.88 0.00962

[N|J] -66.88 0.00962

[N|L] -66.88 0.00962

[N|F] -66.9 0.009454

[N|O] -66.9 0.009454

[N|E] -66.9 0.009454

[E|E] -66.91 0.009309

[CE|E] -66.94 0.009031

[BN|N] -66.96 0.008852

[AN|N] -66.96 0.008852

[CO|O] -66.97 0.008758

[N|G] -66.98 0.008727

[D|D] -67.1 0.0077

[FN|F] -67.35 0.005974

[FN|N] -67.35 0.005974

[F|F] -67.62 0.004597

[C|K] -67.64 0.004474

[C|J] -67.64 0.004474

[C|L] -67.64 0.004474

[C|O] -67.66 0.004397

[C|F] -67.66 0.004397

[C|N] -67.66 0.004397

[C|E] -67.66 0.004397

[C|G] -67.74 0.004059

[BE|E] -67.76 0.004

[AE|E] -67.76 0.004

[EF|F] -67.77 0.003958

[EF|E] -67.77 0.003958

[BO|O] -67.78 0.003905

[AO|O] -67.78 0.003905

[FO|O] -67.79 0.003863

[FO|F] -67.79 0.003863

[K|D] -67.86 0.003608

[J|D] -67.86 0.003608

[L|D] -67.86 0.003608

[O|K] -67.86 0.003608

[O|J] -67.86 0.003608

[O|L] -67.86 0.003608

[DE|D] -67.87 0.003569

[O|F] -67.88 0.003545

[O|N] -67.88 0.003545

[O|E] -67.88 0.003545

[DO|D] -67.88 0.003526

[DF|D] -67.88 0.003523

[K|DK] -67.94 0.003326

[J|DJ] -67.94 0.003326

[L|DL] -67.94 0.003326

[O|G] -67.96 0.003273

[EO|O] -67.96 0.003263

[EO|E] -67.96 0.003263

[N|FN] -67.96 0.003258

[N|EN] -67.96 0.003258

[N|NO] -67.96 0.003258

[IN|N] -67.98 0.003192

[N|KN] -67.99 0.003178

[N|JN] -67.99 0.003178

[N|LN] -67.99 0.003178

[G|D] -67.99 0.003173

[E|K] -68 0.003139

[E|J] -68 0.003139

[E|L] -68 0.003139

[N|GN] -68 0.00313

[E|F] -68.02 0.003085

[E|N] -68.02 0.003085

[E|O] -68.02 0.003085

[G|DG] -68.07 0.002918

[E|G] -68.1 0.002847

[CN|C] -68.1 0.002825

[CF|F] -68.15 0.002704

[KN|K] -68.18 0.002613

[JN|J] -68.18 0.002613

[LN|L] -68.18 0.002613

[D|DF] -68.25 0.002445

[D|DO] -68.25 0.002445

[D|DE] -68.25 0.002445

[D|DN] -68.25 0.002445

[KN|N] -68.26 0.002423

[JN|N] -68.26 0.002423

[LN|N] -68.26 0.002423

[D|DG] -68.26 0.002418

[GN|N] -68.26 0.002417

[D|DK] -68.31 0.002298

[D|DJ] -68.31 0.002298

[D|DL] -68.31 0.002298

[GN|G] -68.33 0.002252

[DN|N] -68.41 0.002071

[N|IN] -68.46 0.001983

[B|K] -68.59 0.001735

[A|K] -68.59 0.001735

[B|J] -68.59 0.001735

[A|J] -68.59 0.001735

[B|L] -68.59 0.001735

[A|L] -68.59 0.001735

[N|CN] -68.6 0.001717

[N|BN] -68.6 0.001717

[N|AN] -68.6 0.001717

[B|O] -68.61 0.001705

[A|F] -68.61 0.001705

[B|F] -68.61 0.001705

[A|O] -68.61 0.001705

[B|N] -68.61 0.001705

[B|E] -68.61 0.001705

[A|E] -68.61 0.001705

[A|N] -68.61 0.001705

[B|G] -68.69 0.001574

[A|G] -68.69 0.001574

[F|K] -68.7 0.00155

[F|J] -68.7 0.00155

[F|L] -68.7 0.00155

[F|O] -68.72 0.001523

[F|N] -68.72 0.001523

[F|E] -68.72 0.001523

[F|G] -68.8 0.001406

[C|C] -68.85 0.001343

[O|FO] -68.94 0.001222

[O|NO] -68.94 0.001222

[O|EO] -68.94 0.001222

[K|K] -68.96 0.001206

[J|J] -68.96 0.001206

[L|L] -68.96 0.001206

[O|KO] -68.97 0.001192

[O|JO] -68.97 0.001192

[O|LO] -68.97 0.001192

[O|GO] -68.98 0.001174

[E|EF] -69.08 0.001063

[E|EO] -69.08 0.001063

[E|EN] -69.08 0.001063

[E|EK] -69.11 0.001037

[E|EJ] -69.11 0.001037

[E|EL] -69.11 0.001037

[E|EG] -69.12 0.001021

[N|B] -69.19 0.0009569

[N|A] -69.19 0.0009569

[N|C] -69.19 0.0009569

[G|G] -69.22 0.0009254

[CE|C] -69.23 0.0009148

[BF|F] -69.25 0.0008989

[AF|F] -69.25 0.0008989

[BN|B] -69.25 0.0008966

[AN|A] -69.25 0.0008966

[CO|C] -69.26 0.0008871

[N|I] -69.32 0.0008412

[CK|K] -69.34 0.0008199

[CJ|J] -69.34 0.0008199

[CL|L] -69.34 0.0008199

[C|CK] -69.36 0.0008025

[C|CJ] -69.36 0.0008025

[C|CL] -69.36 0.0008025

[C|CO] -69.37 0.000799

[C|CN] -69.37 0.000799

[C|CF] -69.37 0.000799

[C|CE] -69.37 0.000799

[EI|E] -69.41 0.0007647

[IO|O] -69.43 0.0007465

[C|CG] -69.43 0.0007463

[O|IO] -69.44 0.0007436

[CG|G] -69.53 0.0006772

[E|EI] -69.58 0.000647

[O|BO] -69.58 0.0006437

[O|CO] -69.58 0.0006437

[O|AO] -69.58 0.0006437

[EK|K] -69.66 0.0005972

[EJ|J] -69.66 0.0005972

[EL|L] -69.66 0.0005972

[KO|K] -69.67 0.0005895

[JO|J] -69.67 0.0005895

[LO|L] -69.67 0.0005895

[I|O] -69.7 0.0005721

[I|F] -69.7 0.0005721

[I|E] -69.7 0.0005721

[I|N] -69.7 0.0005721

[E|AE] -69.72 0.0005601

[E|BE] -69.72 0.0005601

[E|CE] -69.72 0.0005601

[EK|E] -69.73 0.0005537

[EJ|E] -69.73 0.0005537

[EL|E] -69.73 0.0005537

[KO|O] -69.75 0.0005466

[JO|O] -69.75 0.0005466

[LO|O] -69.75 0.0005466

[EG|E] -69.77 0.000532

[GO|O] -69.78 0.0005304

[I|G] -69.78 0.0005288

[DK|D] -69.78 0.000526

[DJ|D] -69.78 0.000526

At node N8:

split lnL Rel.Prob

[N|N] -64.37 0.1177

[N|NO] -64.5 0.1034

[CN|N] -64.79 0.07799

[N|EN] -65.06 0.05934

[C|O] -65.78 0.02888

[C|N] -65.84 0.02729

[C|E] -65.84 0.02729

[N|O] -66.24 0.01817

[F|O] -66.24 0.01817

[N|DN] -66.29 0.01729

[F|N] -66.3 0.01718

[F|E] -66.3 0.01718

[N|E] -66.3 0.01718

[BN|N] -66.31 0.01697

[AN|N] -66.31 0.01697

[B|O] -66.69 0.0116

[A|O] -66.69 0.0116

[B|N] -66.75 0.01097

[B|E] -66.75 0.01097

[A|N] -66.75 0.01097

[A|E] -66.75 0.01097

[O|O] -66.82 0.01018

[E|E] -66.88 0.009619

[O|NO] -67.01 0.008454

[E|EO] -67.01 0.008454

[O|EO] -67.01 0.008454

[FN|N] -67.11 0.007607

[C|CE] -67.16 0.007287

[C|CN] -67.16 0.007287

[DN|N] -67.18 0.007125

[F|F] -67.33 0.006134

[C|C] -67.43 0.005557

[E|EN] -67.56 0.004849

[N|KN] -67.59 0.0047

[N|JN] -67.59 0.0047

[N|LN] -67.59 0.0047

[F|EF] -67.62 0.004594

[N|FN] -67.62 0.004594

[F|FN] -67.62 0.004594

[N|GN] -67.68 0.004303

[N|D] -67.69 0.00426

[F|D] -67.69 0.00426

[C|CO] -67.69 0.004254

[CF|F] -67.74 0.004065

[CF|C] -67.75 0.004019

[CN|C] -67.75 0.004019

[NO|O] -67.79 0.003876

[FO|O] -67.79 0.003876

[KN|N] -67.83 0.003704

[JN|N] -67.83 0.003704

[LN|N] -67.83 0.003704

[NO|N] -67.84 0.003663

[EN|N] -67.84 0.003663

[EN|E] -67.84 0.003663

[EF|E] -67.84 0.003663

[CO|O] -67.85 0.003627

[GN|N] -67.9 0.003474

[CE|E] -67.91 0.003428

[N|IN] -67.97 0.003236

[B|BE] -68.07 0.002928

[B|BN] -68.07 0.002928

[A|AE] -68.07 0.002928

[A|AN] -68.07 0.002928

[N|CN] -68.08 0.002896

[N|AN] -68.08 0.002896

[N|BN] -68.08 0.002896

[F|FO] -68.16 0.002679

[IN|N] -68.23 0.002487

[BO|O] -68.24 0.002461

[AO|O] -68.24 0.002461

[AE|E] -68.3 0.002326

[BE|E] -68.3 0.002326

[B|B] -68.34 0.002233

[A|A] -68.34 0.002233

[B|BO] -68.61 0.001709

[A|AO] -68.61 0.001709

[BC|C] -68.63 0.001675

[BC|B] -68.63 0.001675

[AC|C] -68.63 0.001675

[AC|A] -68.63 0.001675

[K|O] -68.68 0.001584

[J|O] -68.68 0.001584

[L|O] -68.68 0.001584

[C|K] -68.71 0.001543

[C|J] -68.71 0.001543

[C|L] -68.71 0.001543

[K|N] -68.74 0.001497

[K|E] -68.74 0.001497

[J|N] -68.74 0.001497

[J|E] -68.74 0.001497

[L|N] -68.74 0.001497

[L|E] -68.74 0.001497

[E|O] -68.75 0.001485

[C|F] -68.79 0.001423

[E|DE] -68.8 0.001413

[O|N] -68.8 0.001404

[E|N] -68.8 0.001404

[O|E] -68.8 0.001404

[G|O] -68.85 0.001339

[D|O] -68.89 0.001292

[G|N] -68.91 0.001265

[G|E] -68.91 0.001265

[C|G] -68.93 0.001234

[D|N] -68.94 0.001221

[D|E] -68.94 0.001221

[I|O] -68.96 0.001199

[I|N] -69.02 0.001133

[I|E] -69.02 0.001133

[N|K] -69.18 0.0009648

[F|K] -69.18 0.0009648

[N|J] -69.18 0.0009648

[F|J] -69.18 0.0009648

[F|L] -69.18 0.0009648

[N|L] -69.18 0.0009648

[D|D] -69.2 0.0009415

[N|F] -69.25 0.0008952

[BF|F] -69.27 0.0008842

[AF|F] -69.27 0.0008842

[BN|B] -69.28 0.0008744

[BF|B] -69.28 0.0008744

[AF|A] -69.28 0.0008744

[AN|A] -69.28 0.0008744

[N|G] -69.38 0.0007916

[F|G] -69.38 0.0007916

[O|DO] -69.4 0.0007719

[F|I] -69.44 0.0007404

[N|I] -69.44 0.0007404

[C|I] -69.46 0.0007254

[F|DF] -69.55 0.0006641

At node N4:

split lnL Rel.Prob

[N|C] -63.48 0.2886

[F|C] -64.71 0.08428

[N|CN] -64.91 0.06883

[B|C] -65.02 0.06146

[A|C] -65.02 0.06146

[BN|B] -65.69 0.03159

[AN|A] -65.69 0.03159

[BN|N] -65.69 0.03154

[AN|N] -65.69 0.03154

[N|N] -65.99 0.02328

[F|CF] -66.14 0.0201

[B|BC] -66.46 0.01466

[A|AC] -66.46 0.01466

[CN|C] -66.67 0.01182

[BF|B] -66.92 0.009226

[AF|A] -66.92 0.009226

[BF|F] -66.92 0.00921

[AF|F] -66.92 0.00921

[N|O] -67 0.008558

[F|F] -67.01 0.008462

[N|E] -67.03 0.008289

[B|B] -67.37 0.005861

[A|A] -67.37 0.005861

[F|O] -67.5 0.00518

[N|K] -67.5 0.005172

[N|J] -67.5 0.005172

[N|L] -67.5 0.005172

[N|F] -67.55 0.004911

[F|N] -67.55 0.004911

[F|E] -67.55 0.004911

[N|I] -67.56 0.004853

[N|G] -67.62 0.004589

[B|O] -67.67 0.004369

[A|O] -67.67 0.004369

[N|B] -67.72 0.004135

[N|A] -67.72 0.004135

[B|N] -67.72 0.004128

[A|N] -67.72 0.004128

[B|E] -67.72 0.004128

[A|E] -67.72 0.004128

[CF|C] -67.9 0.003451

[BC|C] -68.22 0.002517

[AC|C] -68.22 0.002517

[C|C] -68.31 0.002303

[O|C] -68.6 0.00172

[F|K] -68.64 0.001652

[F|J] -68.64 0.001652

[F|L] -68.64 0.001652

[E|C] -68.66 0.001625

[F|I] -68.74 0.001493

[F|G] -68.81 0.001391

[FN|N] -68.84 0.001357

At node N2:

split lnL Rel.Prob

[B|N] -63.34 0.3299

[A|N] -63.34 0.3299

[B|F] -64.19 0.1409

[A|F] -64.19 0.1409

[B|BN] -67.45 0.005414

[A|AN] -67.45 0.005414

At node N7:

split lnL Rel.Prob

[N|O] -63.71 0.2282

[N|NO] -64.5 0.1039

[E|O] -64.59 0.09478

[EN|N] -64.64 0.09005

[EN|E] -64.64 0.09005

[N|N] -65.26 0.0484

[E|EO] -65.38 0.04315

[E|E] -66.11 0.02074

[NO|O] -66.15 0.01999

[N|D] -66.29 0.01737

[N|C] -66.57 0.01306

[N|F] -66.64 0.01219

[E|C] -66.91 0.009314

[N|A] -66.99 0.00863

[N|B] -66.99 0.00863

[EO|O] -67.03 0.008301

[N|K] -67.04 0.008205

[N|J] -67.04 0.008205

[N|L] -67.04 0.008205

[E|N] -67.1 0.007693

[E|F] -67.1 0.007693

[N|E] -67.1 0.007693

[N|I] -67.13 0.007453

[N|G] -67.19 0.007061

[E|D] -67.36 0.005924

[O|O] -67.42 0.0056

[E|A] -67.56 0.004882

[E|B] -67.56 0.004882

[E|K] -67.9 0.003472

[E|J] -67.9 0.003472

[E|L] -67.9 0.003472

[E|I] -68.05 0.002987

[E|G] -68.09 0.002869

[C|O] -68.22 0.002519

[F|O] -68.41 0.002077

[N|DN] -68.52 0.001856

[N|CN] -68.67 0.00161

[DN|D] -68.72 0.001523

[D|O] -68.73 0.001514

[N|FN] -68.74 0.001499

[B|O] -68.86 0.001321

[A|O] -68.86 0.001321

[DN|N] -68.96 0.001205

[O|CO] -69 0.001149

[E|CE] -69 0.001148

[CN|N] -69 0.001148

[C|CO] -69.01 0.001147

[CN|C] -69.01 0.001144

[FN|N] -69.08 0.001068

[FN|F] -69.08 0.001068

[N|BN] -69.08 0.001064

[N|AN] -69.08 0.001064

[N|KN] -69.17 0.0009759

[N|JN] -69.17 0.0009759

[N|LN] -69.17 0.0009759

[O|NO] -69.2 0.0009458

[E|EN] -69.2 0.0009458

[O|FO] -69.2 0.0009458

[F|FO] -69.2 0.0009458

[N|EN] -69.2 0.0009458

At node N19:

split lnL Rel.Prob

[GI|G] -66.62 0.01241

[I|I] -66.82 0.01019

[K|K] -66.9 0.009426

[G|G] -66.93 0.009128

[I|N] -67.11 0.007613

[I|O] -67.11 0.007613

[I|E] -67.11 0.007613

[I|F] -67.11 0.007613

[GK|K] -67.13 0.007467

[I|C] -67.17 0.007194

[I|A] -67.17 0.007194

[I|B] -67.17 0.007194

[I|G] -67.18 0.00713

[G|K] -67.18 0.007113

[G|J] -67.18 0.007113

[G|L] -67.27 0.006535

[GI|I] -67.34 0.006082

[HI|I] -67.35 0.006032

[G|GK] -67.37 0.005859

[G|GJ] -67.37 0.005859

[K|JK] -67.37 0.005858

[J|J] -67.39 0.005754

[K|KL] -67.42 0.005587

[IN|N] -67.42 0.005583

[IO|O] -67.42 0.005583

[EI|E] -67.42 0.005583

[FI|F] -67.42 0.005583

[L|L] -67.48 0.005286

[CI|C] -67.48 0.005271

[AI|A] -67.48 0.005271

[BI|B] -67.48 0.005271

[G|GL] -67.49 0.005239

[H|I] -67.55 0.004934

[N|N] -67.58 0.004748

[O|O] -67.58 0.004747

[E|E] -67.58 0.004747

[F|F] -67.58 0.004747

[C|C] -67.64 0.004473

[A|A] -67.64 0.004473

[B|B] -67.64 0.004473

[GJ|J] -67.65 0.004433

[I|IN] -67.69 0.004255

[I|IO] -67.69 0.004255

[I|EI] -67.69 0.004255

[I|FI] -67.69 0.004255

[I|CI] -67.73 0.004086

[I|AI] -67.73 0.004086

[I|BI] -67.73 0.004086

[GL|L] -67.74 0.004073

[I|GI] -67.75 0.004025

[G|D] -67.77 0.003963

[J|JK] -67.87 0.003576

[N|K] -67.9 0.003467

[N|J] -67.9 0.003467

[O|K] -67.9 0.003467

[O|J] -67.9 0.003467

[E|J] -67.9 0.003467

[E|K] -67.9 0.003467

[F|K] -67.9 0.003467

[F|J] -67.9 0.003467

[C|J] -67.9 0.003455

[C|K] -67.9 0.003455

[A|J] -67.9 0.003455

[B|J] -67.9 0.003455

[A|K] -67.9 0.003455

[B|K] -67.9 0.003455

[L|KL] -67.92 0.003411

[L|JL] -67.92 0.003411

[J|JL] -67.92 0.003411

[G|N] -67.97 0.003222

[G|O] -67.97 0.003222

[G|E] -67.97 0.003222

[G|F] -67.97 0.003222

[KN|K] -67.98 0.003201

[KO|K] -67.98 0.003201

[EK|K] -67.98 0.003201

[FK|K] -67.98 0.003201

[CK|K] -67.98 0.003185

[BK|K] -67.98 0.003185

[AK|K] -67.98 0.003185

[N|L] -67.98 0.003185

[O|L] -67.98 0.003185

[E|L] -67.98 0.003185

[F|L] -67.98 0.003185

[C|L] -67.99 0.003173

[A|L] -67.99 0.003173

[B|L] -67.99 0.003173

[GK|G] -67.99 0.003172

[D|D] -68 0.003146

[K|J] -68.01 0.003118

[G|C] -68.03 0.003045

[G|A] -68.03 0.003045

[G|B] -68.03 0.003045

[G|DG] -68.05 0.002966

[N|JN] -68.07 0.002912

[N|KN] -68.07 0.002912

[O|KO] -68.07 0.002912

[O|JO] -68.07 0.002912

[E|EK] -68.07 0.002912

[E|EJ] -68.07 0.002912

[F|FJ] -68.07 0.002912

[F|FK] -68.07 0.002912

[C|CK] -68.09 0.002869

[C|CJ] -68.09 0.002869

[A|AK] -68.09 0.002869

[A|AJ] -68.09 0.002869

[B|BK] -68.09 0.002869

[B|BJ] -68.09 0.002869

[K|L] -68.09 0.002864

[K|DK] -68.11 0.002821

[K|KN] -68.18 0.002621

[K|KO] -68.18 0.002621

[K|EK] -68.18 0.002621

[K|FK] -68.18 0.002621

[N|LN] -68.19 0.002605

[O|LO] -68.19 0.002605

[E|EL] -68.19 0.002605

[F|FL] -68.19 0.002605

[K|CK] -68.19 0.002579

[K|AK] -68.19 0.002579

[K|BK] -68.19 0.002579

[G|GN] -68.2 0.002576

[G|GO] -68.2 0.002575

[G|EG] -68.2 0.002575

[G|FG] -68.2 0.002575

[IN|I] -68.2 0.002568

[IO|I] -68.2 0.002568

[EI|I] -68.2 0.002568

[FI|I] -68.2 0.002568

[K|GK] -68.2 0.002565

[C|CL] -68.2 0.002564

[A|AL] -68.2 0.002564

[B|BL] -68.2 0.002564

[CI|I] -68.2 0.002553

[AI|I] -68.2 0.002553

[BI|I] -68.2 0.002553

[GN|N] -68.22 0.002514

[GO|O] -68.22 0.002513

[EG|E] -68.22 0.002513

[FG|F] -68.22 0.002513

[G|CG] -68.23 0.002501

[G|AG] -68.23 0.002501

[G|BG] -68.23 0.002501

[DG|D] -68.24 0.002471

[CG|C] -68.28 0.002375

[AG|A] -68.28 0.002375

[BG|B] -68.28 0.002375

[GN|G] -68.28 0.002359

[GO|G] -68.28 0.002359

[EG|G] -68.28 0.002359

[FG|G] -68.28 0.002359

[CG|G] -68.29 0.002348

[AG|G] -68.29 0.002348

[BG|G] -68.29 0.002348

[KL|K] -68.44 0.002015

[JK|J] -68.44 0.002015

[JK|K] -68.44 0.002015

[DK|K] -68.46 0.00198

[JN|J] -68.47 0.001965

[JO|J] -68.47 0.001965

[EJ|J] -68.47 0.001965

[FJ|J] -68.47 0.001965

[CJ|J] -68.47 0.001955

[BJ|J] -68.47 0.001955

[AJ|J] -68.47 0.001955

[N|D] -68.48 0.001932

[O|D] -68.48 0.001931

[E|D] -68.48 0.001931

[F|D] -68.48 0.001931

[L|K] -68.5 0.001903

[L|J] -68.5 0.001903

[J|K] -68.5 0.001903

[GL|G] -68.51 0.001883

[GJ|G] -68.51 0.001883

[D|K] -68.52 0.001869

[D|J] -68.52 0.001869

[DG|G] -68.52 0.00186

[KL|L] -68.53 0.001851

[LN|L] -68.55 0.001805

[LO|L] -68.55 0.001805

[EL|L] -68.55 0.001805

[FL|L] -68.55 0.001805

[GM|G] -68.56 0.001796

[CL|L] -68.56 0.001796

[AL|L] -68.56 0.001796

[BL|L] -68.56 0.001796

[H|HI] -68.58 0.001759

[J|L] -68.58 0.001748

[K|D] -68.59 0.001737

[J|DJ] -68.6 0.001722

[D|L] -68.6 0.001717

[D|DK] -68.6 0.001713

[D|DJ] -68.6 0.001713

[G|GI] -68.65 0.001644

[J|JN] -68.67 0.0016

[J|JO] -68.67 0.0016

[J|EJ] -68.67 0.0016

[J|FJ] -68.67 0.0016

[J|CJ] -68.69 0.001574

[J|AJ] -68.69 0.001574

[J|BJ] -68.69 0.001574

[H|H] -68.69 0.001572

[O|N] -68.69 0.00157

[N|O] -68.69 0.00157

[N|E] -68.69 0.00157

[E|N] -68.69 0.00157

[N|F] -68.69 0.00157

[F|N] -68.69 0.00157

[O|E] -68.69 0.00157

[E|O] -68.69 0.00157

[O|F] -68.69 0.00157

[F|O] -68.69 0.00157

[E|F] -68.69 0.00157

[F|E] -68.69 0.00157

[J|GJ] -68.69 0.001566

[C|N] -68.69 0.001565

[A|N] -68.69 0.001565

[B|N] -68.69 0.001565

[C|O] -68.69 0.001565

[C|E] -68.69 0.001565

[B|O] -68.69 0.001565

[A|O] -68.69 0.001565

[B|E] -68.69 0.001565

[A|E] -68.69 0.001565

[C|F] -68.69 0.001565

[A|F] -68.69 0.001565

[B|F] -68.69 0.001565

[G|GM] -68.7 0.001552

[L|DL] -68.71 0.00154

[D|DL] -68.72 0.001531

[N|DN] -68.74 0.001492

[O|DO] -68.74 0.001492

[E|DE] -68.74 0.001492

[F|DF] -68.74 0.001492

[N|C] -68.75 0.001484

[N|A] -68.75 0.001484

[N|B] -68.75 0.001484

[O|C] -68.75 0.001484

[E|C] -68.75 0.001484

[O|A] -68.75 0.001484

[O|B] -68.75 0.001484

[E|A] -68.75 0.001484

[E|B] -68.75 0.001484

[F|C] -68.75 0.001484

[F|A] -68.75 0.001484

[F|B] -68.75 0.001484

[C|A] -68.75 0.001479

[C|B] -68.75 0.001479

[A|C] -68.75 0.001479

[B|C] -68.75 0.001479

[B|A] -68.75 0.001479

[A|B] -68.75 0.001479

[N|G] -68.76 0.001471

[O|G] -68.76 0.001471

[E|G] -68.76 0.001471

[F|G] -68.76 0.001471

[C|G] -68.76 0.001466

[B|G] -68.76 0.001466

[A|G] -68.76 0.001466

[KN|N] -68.77 0.001449

[KO|O] -68.77 0.001449

[EK|E] -68.77 0.001449

[FK|F] -68.77 0.001449

[L|LN] -68.78 0.001431

[L|LO] -68.78 0.001431

[L|EL] -68.78 0.001431

[L|FL] -68.78 0.001431

[G|I] -68.79 0.001427

[K|N] -68.8 0.001412

[K|O] -68.8 0.001412

[K|E] -68.8 0.001412

[K|F] -68.8 0.001412

[L|CL] -68.8 0.001407

[L|AL] -68.8 0.001407

[L|BL] -68.8 0.001407

[L|GL] -68.81 0.0014

[CK|C] -68.83 0.001369

[AK|A] -68.83 0.001369

[BK|B] -68.83 0.001369

[K|C] -68.85 0.001335

[K|A] -68.85 0.001335

[K|B] -68.85 0.001335

[K|G] -68.86 0.001323

[O|NO] -68.88 0.001301

[N|NO] -68.88 0.001301

[E|EN] -68.88 0.001301

[N|EN] -68.88 0.001301

[N|FN] -68.88 0.001301

[F|FN] -68.88 0.001301

[E|EO] -68.88 0.001301

[O|EO] -68.88 0.001301

[O|FO] -68.88 0.001301

[F|FO] -68.88 0.001301

[E|EF] -68.88 0.001301

[F|EF] -68.88 0.001301

[G|M] -68.89 0.001282

[C|CN] -68.91 0.001266

[A|AN] -68.91 0.001266

[B|BN] -68.91 0.001266

[C|CO] -68.91 0.001266

[C|CE] -68.91 0.001266

[B|BO] -68.91 0.001266

[A|AO] -68.91 0.001266

[B|BE] -68.91 0.001266

[A|AE] -68.91 0.001266

[C|CF] -68.91 0.001266

[A|AF] -68.91 0.001266

[B|BF] -68.91 0.001266

[N|CN] -68.91 0.001265

[N|AN] -68.91 0.001265

[N|BN] -68.91 0.001265

[O|CO] -68.91 0.001265

[E|CE] -68.91 0.001265

[O|BO] -68.91 0.001265

[O|AO] -68.91 0.001265

[E|BE] -68.91 0.001265

[E|AE] -68.91 0.001265

[F|CF] -68.91 0.001265

[F|AF] -68.91 0.001265

[F|BF] -68.91 0.001265

[N|GN] -68.92 0.001253

[O|GO] -68.92 0.001253

[E|EG] -68.92 0.001253

[F|FG] -68.92 0.001253

[NO|O] -68.93 0.001233

[NO|N] -68.93 0.001233

[EN|E] -68.93 0.001233

[EN|N] -68.93 0.001233

[FN|F] -68.93 0.001233

[FN|N] -68.93 0.001233

[EO|E] -68.93 0.001233

[EO|O] -68.93 0.001233

[FO|F] -68.93 0.001233

[FO|O] -68.93 0.001233

[EF|F] -68.93 0.001233

[EF|E] -68.93 0.001233

[C|AC] -68.94 0.00123

[A|AC] -68.94 0.00123

[C|BC] -68.94 0.00123

[B|BC] -68.94 0.00123

[A|AB] -68.94 0.00123

[B|AB] -68.94 0.00123

[CN|N] -68.94 0.001227

[AN|N] -68.94 0.001227

[BN|N] -68.94 0.001227

[CO|O] -68.94 0.001227

[CE|E] -68.94 0.001227

[AO|O] -68.94 0.001227

[BO|O] -68.94 0.001227

[BE|E] -68.94 0.001227

[AE|E] -68.94 0.001227

[CF|F] -68.94 0.001227

[BF|F] -68.94 0.001227

[AF|F] -68.94 0.001227

[C|CG] -68.95 0.001217

[A|AG] -68.95 0.001217

[B|BG] -68.95 0.001217

[JL|J] -68.96 0.001201

[DJ|J] -68.97 0.001188

[CN|C] -68.99 0.001165

[AN|A] -68.99 0.001165

[BN|B] -68.99 0.001165

[CO|C] -68.99 0.001165

[CE|C] -68.99 0.001165

[AO|A] -68.99 0.001165

[BO|B] -68.99 0.001165

[AE|A] -68.99 0.001165

[BE|B] -68.99 0.001165

[CF|C] -68.99 0.001165

[BF|B] -68.99 0.001165

[AF|A] -68.99 0.001165

[BC|C] -68.99 0.001159

[BC|B] -68.99 0.001159

[AC|A] -68.99 0.001159

[AC|C] -68.99 0.001159

[AB|A] -68.99 0.001159

[AB|B] -68.99 0.001159

[M|M] -69.03 0.001124

[DK|D] -69.03 0.001118

[JL|L] -69.04 0.001103

[DN|D] -69.05 0.001102

[DO|D] -69.05 0.001102

[DE|D] -69.05 0.001102

[DF|D] -69.05 0.001102

[DL|L] -69.06 0.001091

[L|D] -69.08 0.00106

[J|D] -69.08 0.00106

[I|HI] -69.22 0.0009253

[HI|H] -69.25 0.0009015

[LN|N] -69.26 0.0008896

[JN|N] -69.26 0.0008896

[LO|O] -69.26 0.0008896

[JO|O] -69.26 0.0008896

[EL|E] -69.26 0.0008896

[EJ|E] -69.26 0.0008896

[FL|F] -69.26 0.0008896

[FJ|F] -69.26 0.0008896

[M|C] -69.26 0.0008865

[M|B] -69.26 0.0008865

[M|A] -69.26 0.0008865

[DN|N] -69.27 0.0008839

[DO|O] -69.27 0.0008838

[DE|E] -69.27 0.0008838

[DF|F] -69.27 0.0008838

[M|G] -69.27 0.0008787

At node N15:

split lnL Rel.Prob

[G|I] -64.2 0.1404

[GI|I] -64.94 0.06715

[I|H] -65.06 0.05931

[I|HI] -65.4 0.04235

[G|GI] -65.41 0.04171

[GK|G] -65.5 0.03819

[I|I] -65.66 0.03238

[N|I] -66.26 0.01794

[O|I] -66.26 0.01794

[E|I] -66.26 0.01794

[F|I] -66.26 0.01794

[C|I] -66.29 0.01731

[B|I] -66.29 0.01731

[A|I] -66.29 0.01731

[G|G] -66.51 0.01391

[HI|H] -66.51 0.01388

[H|H] -67.17 0.007196

[IN|I] -67.22 0.006805

[IO|I] -67.22 0.006805

[EI|I] -67.22 0.006805

[FI|I] -67.22 0.006805

[K|N] -67.25 0.006668

[K|O] -67.25 0.006668

[K|E] -67.25 0.006668

[K|F] -67.25 0.006668

[CI|I] -67.26 0.006587

[AI|I] -67.26 0.006587

[BI|I] -67.26 0.006587

[K|C] -67.27 0.006528

[K|B] -67.27 0.006528

[K|A] -67.27 0.006528

[K|G] -67.33 0.006129

[N|IN] -67.47 0.005336

[O|IO] -67.47 0.005336

[E|EI] -67.47 0.005336

[F|FI] -67.47 0.005336

[C|CI] -67.5 0.005147

[A|AI] -67.5 0.005147

[B|BI] -67.5 0.005147

[GK|K] -67.55 0.004921

[G|N] -67.56 0.004843

[G|O] -67.56 0.004843

[G|E] -67.56 0.004843

[G|F] -67.56 0.004843

[G|C] -67.59 0.004724

[G|B] -67.59 0.004724

[G|A] -67.59 0.004724

[I|IN] -67.9 0.003473

[I|IO] -67.9 0.003473

[I|EI] -67.9 0.003473

[I|FI] -67.9 0.003473

[I|GI] -67.92 0.003382

[I|CI] -67.93 0.003372

[I|AI] -67.93 0.003372

[I|BI] -67.93 0.003372

[KN|N] -67.97 0.003229

[KO|O] -67.97 0.003229

[EK|E] -67.97 0.003229

[FK|F] -67.97 0.003229

[CK|C] -67.99 0.003161

[BK|B] -67.99 0.003161

[AK|A] -67.99 0.003161

[H|HI] -68.09 0.002853

[GJ|G] -68.19 0.002593

[GL|G] -68.21 0.002543

[K|K] -68.23 0.0025

[DG|G] -68.27 0.002385

[GN|N] -68.28 0.002368

[GO|O] -68.28 0.002368

[EG|E] -68.28 0.002368

[FG|F] -68.28 0.002368

[CG|C] -68.31 0.002309

[AG|A] -68.31 0.002309

[BG|B] -68.31 0.002309

[GN|G] -68.35 0.002218

[GO|G] -68.35 0.002218

[EG|G] -68.35 0.002218

[FG|G] -68.35 0.002218

[CG|G] -68.37 0.002162

[AG|G] -68.37 0.002162

[BG|G] -68.37 0.002162

[N|N] -68.49 0.001913

[O|O] -68.49 0.001913

[E|E] -68.49 0.001913

[F|F] -68.49 0.001913

[C|C] -68.54 0.001821

[B|B] -68.54 0.001821

[A|A] -68.54 0.001821

[GI|G] -68.55 0.001804

[GM|G] -68.56 0.001797

[G|GN] -68.61 0.001697

[G|GO] -68.61 0.001697

[G|EG] -68.61 0.001697

[G|FG] -68.61 0.001697

[G|CG] -68.64 0.001655

[G|AG] -68.64 0.001655

[G|BG] -68.64 0.001655

[HI|I] -68.82 0.001375

[K|KN] -68.87 0.001311

[K|KO] -68.87 0.001311

[K|EK] -68.87 0.001311

[K|FK] -68.87 0.001311

[K|CK] -68.89 0.001283

[K|BK] -68.89 0.001283

[K|AK] -68.89 0.001283

[K|GK] -68.95 0.001214

[G|GK] -69.1 0.001047

[G|GJ] -69.1 0.001047

[G|GL] -69.12 0.001027

[G|DG] -69.21 0.0009316

[K|J] -69.23 0.0009162

[K|L] -69.25 0.000901

[D|N] -69.31 0.0008496

[D|O] -69.31 0.0008496

[D|E] -69.31 0.0008496

[D|F] -69.31 0.0008496

[D|G] -69.39 0.0007795

[J|N] -69.41 0.000762

[J|O] -69.41 0.0007619

[J|E] -69.41 0.0007619

[J|F] -69.41 0.0007619

[L|N] -69.43 0.0007475

[L|O] -69.43 0.0007475

[L|E] -69.43 0.0007475

[L|F] -69.43 0.0007475

[J|C] -69.44 0.000746

[J|A] -69.44 0.000746

[J|B] -69.44 0.000746

[L|C] -69.45 0.0007316

[L|B] -69.45 0.0007316

[L|A] -69.45 0.0007316

[G|GM] -69.47 0.0007219

[J|G] -69.5 0.0007004

[L|G] -69.52 0.0006869

[G|K] -69.53 0.0006807

[G|J] -69.53 0.0006807

[K|D] -69.54 0.0006737

[G|L] -69.55 0.0006676

[N|O] -69.63 0.0006163

[O|N] -69.63 0.0006163

[N|E] -69.63 0.0006163

[E|N] -69.63 0.0006163

[N|F] -69.63 0.0006163

[F|N] -69.63 0.0006163

[O|E] -69.63 0.0006163

[E|O] -69.63 0.0006163

[O|F] -69.63 0.0006163

[F|O] -69.63 0.0006163

[F|E] -69.63 0.0006163

[E|F] -69.63 0.0006163

[N|C] -69.65 0.0006014

[N|B] -69.65 0.0006014

[N|A] -69.65 0.0006014

[O|C] -69.65 0.0006014

[E|C] -69.65 0.0006014

[O|B] -69.65 0.0006014

[O|A] -69.65 0.0006014

[E|B] -69.65 0.0006014

[E|A] -69.65 0.0006014

[F|C] -69.65 0.0006014

[F|B] -69.65 0.0006014

[F|A] -69.65 0.0006014

[C|N] -69.66 0.0005974

[B|N] -69.66 0.0005974

[A|N] -69.66 0.0005974

[C|O] -69.66 0.0005974

[C|E] -69.66 0.0005974

[A|O] -69.66 0.0005974

[B|O] -69.66 0.0005974

[A|E] -69.66 0.0005974

[B|E] -69.66 0.0005974

[C|F] -69.66 0.0005974

[A|F] -69.66 0.0005974

[B|F] -69.66 0.0005974

[A|C] -69.68 0.0005826

[B|C] -69.68 0.0005826

[C|A] -69.68 0.0005826

[C|B] -69.68 0.0005826

[A|B] -69.68 0.0005826

[B|A] -69.68 0.0005826

[N|G] -69.71 0.0005648

[O|G] -69.71 0.0005648

[E|G] -69.71 0.0005648

[F|G] -69.71 0.0005648

[C|G] -69.75 0.0005472

[A|G] -69.75 0.0005472

[B|G] -69.75 0.0005472

[M|C] -69.83 0.0005028

[M|B] -69.83 0.0005027

[M|A] -69.83 0.0005027

[G|D] -69.85 0.0004937

[M|G] -69.89 0.0004725

[JK|K] -69.95 0.0004452

[JK|J] -69.95 0.0004452

[G|M] -69.96 0.00044

[KL|K] -69.97 0.0004378

[KL|L] -69.97 0.0004378

[DK|K] -70.02 0.0004144

[H|I] -70.07 0.0003956

[KN|K] -70.08 0.0003896

[KO|K] -70.08 0.0003896

At node N18:

split lnL Rel.Prob

[JK|K] -64.88 0.07132

[JK|J] -64.88 0.07132

[K|L] -64.91 0.06908

[K|KL] -64.92 0.06852

[J|L] -64.94 0.06672

[J|JL] -64.95 0.06617

[K|K] -65.83 0.02733

[J|J] -65.87 0.0265

[KL|L] -66.28 0.01746

[JL|L] -66.32 0.01686

[K|D] -66.81 0.01033

[J|D] -66.84 0.009971

[L|L] -66.86 0.009815

[K|G] -66.98 0.008649

[K|N] -67 0.008561

[K|O] -67 0.008561

[K|E] -67 0.008561

[K|F] -67 0.008561

[K|C] -67.01 0.008415

[K|A] -67.01 0.008415

[K|B] -67.01 0.008415

[J|G] -67.01 0.008402

[J|K] -67.02 0.008348

[K|J] -67.02 0.008348

[J|N] -67.03 0.008296

[J|O] -67.03 0.008296

[J|E] -67.03 0.008296

[J|F] -67.03 0.008296

[J|C] -67.04 0.008155

[J|A] -67.04 0.008155

[J|B] -67.04 0.008155

[D|L] -67.86 0.003617

[D|DL] -67.86 0.003606

[L|GL] -67.87 0.003562

[K|GK] -67.9 0.003452

[K|DK] -67.93 0.003364

[J|GJ] -67.93 0.003354

[L|LN] -67.95 0.003282

[L|LO] -67.95 0.003282

[L|EL] -67.95 0.003282

[L|FL] -67.95 0.003282

[K|KN] -67.95 0.003281

[K|KO] -67.95 0.003281

[K|EK] -67.95 0.003281

[K|FK] -67.95 0.003281

[L|CL] -67.95 0.003281

[L|AL] -67.95 0.003281

[L|BL] -67.95 0.003281

[K|CK] -67.96 0.003267

[K|AK] -67.96 0.003267

[K|BK] -67.96 0.003267

[J|DJ] -67.96 0.003247

[J|JN] -67.99 0.00318

[J|JO] -67.99 0.00318

[J|EJ] -67.99 0.00318

[J|FJ] -67.99 0.00318

[J|CJ] -67.99 0.003166

[J|AJ] -67.99 0.003166

[J|BJ] -67.99 0.003166

[G|L] -67.99 0.003161

[G|GL] -68 0.003127

[N|L] -68.01 0.003099

[O|L] -68.01 0.003099

[E|L] -68.01 0.003099

[F|L] -68.01 0.003099

[C|L] -68.02 0.00307

[A|L] -68.02 0.00307

[B|L] -68.02 0.00307

[N|LN] -68.02 0.003069

[O|LO] -68.02 0.003069

[E|EL] -68.02 0.003069

[F|FL] -68.02 0.003069

[L|KL] -68.02 0.003066

[J|JK] -68.02 0.003066

[K|JK] -68.02 0.003066

[C|CL] -68.03 0.00304

[A|AL] -68.03 0.00304

[B|BL] -68.03 0.00304

[L|JL] -68.06 0.002961

[L|DL] -68.07 0.002922

[DK|D] -68.18 0.002621

[DJ|D] -68.21 0.00253

[GK|K] -68.22 0.002523

[GJ|J] -68.25 0.002452

[KN|K] -68.3 0.00233

[KO|K] -68.3 0.00233

[EK|K] -68.3 0.00233

[FK|K] -68.3 0.00233

[CK|K] -68.3 0.00233

[AK|K] -68.3 0.00233

[BK|K] -68.3 0.00233

[JN|J] -68.33 0.002258

[JO|J] -68.33 0.002258

[EJ|J] -68.33 0.002258

[FJ|J] -68.33 0.002258

[CJ|J] -68.33 0.002258

[BJ|J] -68.33 0.002258

[AJ|J] -68.33 0.002258

[GK|G] -68.36 0.002181

[KN|N] -68.37 0.002161

[KO|O] -68.37 0.002161

[EK|E] -68.37 0.002161

[FK|F] -68.37 0.002161

[CK|C] -68.39 0.002124

[AK|A] -68.39 0.002124

[BK|B] -68.39 0.002124

[GJ|G] -68.39 0.002118

[KL|K] -68.4 0.002109

[JN|N] -68.4 0.002094

[JO|O] -68.4 0.002094

[EJ|E] -68.4 0.002094

[FJ|F] -68.4 0.002094

[DK|K] -68.41 0.00208

[CJ|C] -68.42 0.002058

[AJ|A] -68.42 0.002058

[BJ|B] -68.42 0.002058

[JL|J] -68.43 0.002037

[DJ|J] -68.45 0.002008

[D|D] -68.57 0.001773

[N|N] -68.98 0.001176

[O|O] -68.98 0.001176

[E|E] -68.98 0.001176

[F|F] -68.98 0.001176

[C|C] -69.01 0.001146

[A|A] -69.01 0.001146

[B|B] -69.01 0.001146

[G|G] -69.05 0.0011

[GL|L] -69.38 0.0007894

[DL|L] -69.43 0.0007505

[LN|L] -69.43 0.0007463

[LO|L] -69.43 0.0007463

[EL|L] -69.43 0.0007463

[FL|L] -69.43 0.0007463

[CL|L] -69.44 0.0007443

[AL|L] -69.44 0.0007443

[BL|L] -69.44 0.0007443

[G|D] -69.89 0.0004739

[N|D] -69.91 0.0004639

[O|D] -69.91 0.0004639

[E|D] -69.91 0.0004639

[F|D] -69.91 0.0004639

[D|G] -69.93 0.0004568

[D|K] -69.93 0.0004528

[D|N] -69.94 0.0004505

[D|O] -69.94 0.0004505

[D|E] -69.94 0.0004505

[D|F] -69.94 0.0004505

[L|D] -69.95 0.0004462

[D|J] -69.97 0.000437

[G|K] -70.08 0.0003932

[G|N] -70.09 0.0003865

[G|O] -70.09 0.0003865

[G|E] -70.09 0.0003865

[G|F] -70.09 0.0003865

[N|K] -70.09 0.0003864

[O|K] -70.09 0.0003864

[E|K] -70.09 0.0003864

***RESULTS OF RUN FROM Margo_host2.lagrange.py INPUT FILE SUBMITTED TO DRYAD***

Global ML at root node:

-lnL = 62.82

dispersal = 3.612

extinction = 0.5202

Ancestral range subdivision/inheritance scenarios ('splits') at

internal nodes.

* Split format: [left|right], where 'left' and 'right' are the ranges

inherited by each descendant branch (on the printed tree, 'left' is

the upper branch, and 'right' the lower branch).

* Only splits within 2 log-likelihood units of the maximum for each

node are shown. 'Rel.Prob' is the relative probability (fraction of

the global likelihood) of a split.

At node N22:

split lnL Rel.Prob

[M|M] -66.77 0.01934

[G|G] -66.84 0.018

[I|I] -66.85 0.01788

[H|H] -67.21 0.01246

[C|C] -67.28 0.01154

[B|B] -67.3 0.01135

[A|A] -67.3 0.01135

[N|N] -67.45 0.009752

[O|O] -67.51 0.00922

[E|E] -67.55 0.00885

[F|F] -67.56 0.008769

[L|L] -67.62 0.008226

[K|K] -67.7 0.007624

[J|J] -67.75 0.007276

[D|D] -67.78 0.007043

[GI|G] -67.94 0.005974

[G|GM] -68.07 0.005279

[I|G] -68.13 0.004936

[G|M] -68.2 0.004621

[I|GI] -68.22 0.004546

[GM|M] -68.36 0.003936

[HI|I] -68.37 0.003915

[C|M] -68.39 0.00383

[I|C] -68.39 0.003818

[I|A] -68.39 0.003818

[I|B] -68.39 0.003818

[B|M] -68.4 0.003768

[A|M] -68.4 0.003768

[C|CM] -68.41 0.003742

[M|GM] -68.43 0.003682

[B|BM] -68.43 0.003681

[A|AM] -68.43 0.003681

[GI|I] -68.45 0.003608

[H|I] -68.46 0.003564

[CI|C] -68.47 0.003522

[BI|B] -68.49 0.003453

[AI|A] -68.49 0.003453

[I|CI] -68.54 0.003288

[I|AI] -68.54 0.003288

[I|BI] -68.54 0.003288

[CM|M] -68.57 0.003205

[GN|G] -68.58 0.003159

[M|CM] -68.58 0.003149

[M|BM] -68.58 0.003149

[M|AM] -68.58 0.003149

[BM|M] -68.58 0.003145

[AM|M] -68.58 0.003145

[G|CG] -68.6 0.003092

[G|BG] -68.6 0.003092

[G|AG] -68.6 0.003092

[IN|I] -68.61 0.00308

[IN|N] -68.62 0.003033

[GO|G] -68.64 0.002983

[I|E] -68.65 0.002937

[I|F] -68.65 0.002937

[I|O] -68.65 0.002934

[I|N] -68.65 0.002934

[IO|I] -68.67 0.002881

[CG|G] -68.68 0.002863

[EG|G] -68.68 0.002856

[GL|G] -68.68 0.002855

[IO|O] -68.69 0.002837

[FG|G] -68.69 0.002829

[BG|G] -68.7 0.002815

[AG|G] -68.7 0.002815

[G|GK] -68.7 0.002796

[G|GI] -68.71 0.002763

[G|GL] -68.72 0.002759

[G|GJ] -68.72 0.002754

[CI|I] -68.72 0.002749

[EI|I] -68.72 0.002739

[G|FG] -68.72 0.002737

[G|EG] -68.72 0.002737

[G|GO] -68.72 0.002735

[G|GN] -68.72 0.002735

[N|G] -68.72 0.002734

[G|DG] -68.73 0.002727

[FI|I] -68.73 0.00271

[I|FI] -68.74 0.002704

[I|EI] -68.74 0.002704

[I|IO] -68.74 0.002701

[I|IN] -68.74 0.002701

[EI|E] -68.74 0.002699

[BI|I] -68.74 0.002695

[AI|I] -68.74 0.002695

[FI|F] -68.75 0.00267

[O|G] -68.78 0.002585

[GK|G] -68.79 0.002569

[C|CG] -68.79 0.002563

[DG|G] -68.79 0.002562

[GM|G] -68.79 0.002555

[B|BG] -68.81 0.002521

[A|AG] -68.81 0.002521

[GJ|G] -68.81 0.002518

[N|GN] -68.82 0.002493

[C|G] -68.82 0.002486

[E|G] -68.82 0.002479

[H|HI] -68.83 0.00247

[F|G] -68.83 0.002456

[B|G] -68.84 0.002446

[A|G] -68.84 0.002446

[O|GO] -68.87 0.002357

[G|C] -68.89 0.002321

[G|A] -68.89 0.002321

[G|B] -68.89 0.002321

[HI|H] -68.91 0.00228

[L|G] -68.91 0.002263

[E|EG] -68.91 0.002262

[F|FG] -68.92 0.002241

[CG|C] -68.94 0.002215

[BG|B] -68.95 0.002178

[AG|A] -68.95 0.002178

[N|C] -68.98 0.002115

[N|A] -68.98 0.002115

[N|B] -68.98 0.002115

[M|G] -68.99 0.002092

[L|GL] -69 0.002081

[I|HI] -69.01 0.002066

[K|G] -69.01 0.002059

[D|G] -69.02 0.002041

[J|G] -69.03 0.002006

[O|C] -69.04 0.002

[O|A] -69.04 0.002

[O|B] -69.04 0.002

[CN|C] -69.04 0.001992

[AN|A] -69.06 0.001963

[BN|B] -69.06 0.001963

[C|AC] -69.07 0.001929

[C|BC] -69.07 0.001929

[C|A] -69.08 0.001923

[C|B] -69.08 0.001923

[K|GK] -69.08 0.001919

[E|C] -69.08 0.001918

[E|A] -69.08 0.001918

[E|B] -69.08 0.001918

[F|C] -69.09 0.0019

[F|A] -69.09 0.0019

[F|B] -69.09 0.0019

[A|AC] -69.09 0.001897

[B|BC] -69.09 0.001897

[B|AB] -69.09 0.001897

[A|AB] -69.09 0.001897

[B|C] -69.09 0.001892

[A|C] -69.09 0.001892

[B|A] -69.09 0.001892

[A|B] -69.09 0.001892

[CO|C] -69.1 0.001886

[GN|N] -69.1 0.001878

[AO|A] -69.11 0.001857

[BO|B] -69.11 0.001857

[D|DG] -69.11 0.001855

[G|K] -69.11 0.001851

[J|GJ] -69.12 0.001842

[G|L] -69.13 0.001817

[G|J] -69.14 0.001813

[G|I] -69.14 0.001811

[CE|C] -69.14 0.001811

[N|CN] -69.14 0.001796

[N|AN] -69.14 0.001796

[N|BN] -69.14 0.001796

[CF|C] -69.15 0.001794

[BC|C] -69.15 0.001786

[AC|C] -69.15 0.001786

[BC|B] -69.15 0.001786

[AC|A] -69.15 0.001786

[G|E] -69.15 0.001785

[G|F] -69.15 0.001785

[G|O] -69.15 0.001783

[G|N] -69.15 0.001783

[AE|A] -69.15 0.001782

[BE|B] -69.15 0.001782

[CL|C] -69.15 0.001781

[GO|O] -69.16 0.001773

[AF|A] -69.16 0.001765

[BF|B] -69.16 0.001765

[AB|A] -69.17 0.001757

[AB|B] -69.17 0.001757

[L|C] -69.17 0.001751

[L|A] -69.17 0.001751

[L|B] -69.17 0.001751

[AL|A] -69.17 0.001745

[BL|B] -69.17 0.001745

[I|H] -69.18 0.001736

[GL|L] -69.18 0.001729

[G|D] -69.19 0.001725

[EG|E] -69.2 0.001699

[O|CO] -69.2 0.001698

[O|AO] -69.2 0.001698

[O|BO] -69.2 0.001698

[N|K] -69.21 0.001687

[C|CK] -69.21 0.001684

[FG|F] -69.21 0.001683

[C|CI] -69.23 0.001656

[N|L] -69.23 0.001656

[B|BK] -69.23 0.001656

[A|AK] -69.23 0.001656

[N|J] -69.23 0.001653

[C|CL] -69.23 0.001653

[N|I] -69.23 0.001651

[C|CJ] -69.23 0.001649

[C|CE] -69.24 0.001635

[C|CF] -69.24 0.001635

[C|CO] -69.24 0.001633

[C|CN] -69.24 0.001633

[E|CE] -69.24 0.00163

[E|BE] -69.24 0.00163

[E|AE] -69.24 0.00163

[A|AI] -69.24 0.001629

[B|BI] -69.24 0.001629

[N|E] -69.24 0.001627

[N|F] -69.24 0.001627

[B|BL] -69.24 0.001626

[A|AL] -69.24 0.001626

[N|O] -69.24 0.001625

[B|BJ] -69.25 0.001622

[A|AJ] -69.25 0.001622

[M|C] -69.25 0.001619

[M|A] -69.25 0.001619

[M|B] -69.25 0.001619

[F|CF] -69.25 0.001615

[F|AF] -69.25 0.001615

[F|BF] -69.25 0.001615

[CK|C] -69.25 0.001614

[CM|C] -69.25 0.001609

[B|BE] -69.26 0.001608

[A|AE] -69.26 0.001608

[B|BF] -69.26 0.001608

[A|AF] -69.26 0.001608

[B|BN] -69.26 0.001607

[B|BO] -69.26 0.001607

[A|AO] -69.26 0.001607

[A|AN] -69.26 0.001607

[O|K] -69.26 0.001595

[K|C] -69.27 0.001593

[K|A] -69.27 0.001593

[K|B] -69.27 0.001593

[GK|K] -69.27 0.001585

[NO|O] -69.27 0.001585

[NO|N] -69.27 0.001585

[AK|A] -69.27 0.001583

[BK|B] -69.27 0.001583

[AM|A] -69.27 0.001579

[BM|B] -69.27 0.001579

[N|D] -69.28 0.001572

[LN|L] -69.28 0.00157

[CJ|C] -69.28 0.00157

[O|L] -69.28 0.001566

[O|J] -69.28 0.001562

[O|I] -69.29 0.001561

[J|C] -69.29 0.001552

[J|A] -69.29 0.001552

[J|B] -69.29 0.001552

[LN|N] -69.3 0.001541

[BJ|B] -69.3 0.001541

[AJ|A] -69.3 0.001541

[O|E] -69.3 0.001538

[O|F] -69.3 0.001538

[O|N] -69.3 0.001537

[C|K] -69.3 0.001534

[CN|N] -69.3 0.001531

[E|K] -69.31 0.00153

[EN|E] -69.31 0.001529

[EN|N] -69.31 0.001528

[N|KN] -69.31 0.001526

[GJ|J] -69.31 0.001522

[FN|F] -69.31 0.001517

[F|K] -69.31 0.001516

[FN|N] -69.31 0.001516

[B|K] -69.32 0.001509

[A|K] -69.32 0.001509

[AN|N] -69.32 0.001509

[BN|N] -69.32 0.001509

[C|L] -69.32 0.001506

[L|CL] -69.32 0.001504

[L|BL] -69.32 0.001504

[L|AL] -69.32 0.001504

[C|J] -69.32 0.001503

[E|L] -69.32 0.001502

[C|I] -69.32 0.001501

[E|J] -69.33 0.001498

[E|I] -69.33 0.001497

[N|IN] -69.33 0.001496

[N|LN] -69.33 0.001492

[N|JN] -69.33 0.001488

[F|L] -69.33 0.001488

[O|D] -69.33 0.001486

[F|J] -69.34 0.001485

[F|I] -69.34 0.001483

[B|L] -69.34 0.001482

[A|L] -69.34 0.001482

[C|E] -69.34 0.001479

[C|F] -69.34 0.001479

[B|J] -69.34 0.001478

[A|J] -69.34 0.001478

[C|O] -69.34 0.001478

[C|N] -69.34 0.001478

[B|I] -69.34 0.001477

[A|I] -69.34 0.001477

[E|F] -69.34 0.001475

[E|O] -69.34 0.001474

[E|N] -69.34 0.001474

[DG|D] -69.34 0.001474

[N|FN] -69.34 0.001473

[N|EN] -69.34 0.001473

[N|NO] -69.34 0.001471

[LO|L] -69.35 0.001466

[N|DN] -69.35 0.001463

[F|E] -69.35 0.001462

[F|O] -69.35 0.00146

[F|N] -69.35 0.00146

[B|E] -69.36 0.001455

[B|F] -69.36 0.001455

[A|E] -69.36 0.001455

[A|F] -69.36 0.001455

[B|O] -69.36 0.001454

[B|N] -69.36 0.001454

[A|O] -69.36 0.001454

[A|N] -69.36 0.001454

[CO|O] -69.36 0.001449

[EO|E] -69.36 0.001447

[EO|O] -69.36 0.001446

[O|KO] -69.36 0.001443

[LO|O] -69.37 0.001439

[KN|K] -69.37 0.001437

[FO|F] -69.37 0.001435

[FO|O] -69.37 0.001434

[AO|O] -69.37 0.001427

[BO|O] -69.37 0.001427

[E|D] -69.38 0.001426

[O|IO] -69.38 0.001415

[F|D] -69.39 0.001413

[O|LO] -69.39 0.001411

[O|JO] -69.39 0.001407

[L|K] -69.4 0.001397

[CL|L] -69.4 0.001395

[K|CK] -69.4 0.001394

[K|BK] -69.4 0.001394

[K|AK] -69.4 0.001394

[CE|E] -69.4 0.001393

[O|FO] -69.4 0.001392

[O|EO] -69.4 0.001392

[EL|L] -69.4 0.001391

[O|NO] -69.4 0.001391

[E|EK] -69.4 0.001385

[KN|N] -69.41 0.001384

[O|DO] -69.41 0.001383

[CF|F] -69.41 0.00138

[EF|E] -69.41 0.001377

[EF|F] -69.41 0.001377

[FL|L] -69.41 0.001375

[F|FK] -69.41 0.001373

[AE|E] -69.42 0.00137

[BE|E] -69.42 0.00137

[L|J] -69.42 0.001368

[BL|L] -69.42 0.001367

[AL|L] -69.42 0.001367

[EL|E] -69.42 0.001366

[JN|J] -69.42 0.001366

[DN|N] -69.42 0.001366

[E|EI] -69.42 0.001358

[BF|F] -69.42 0.001358

[AF|F] -69.42 0.001358

[E|EL] -69.43 0.001354

[E|EJ] -69.43 0.001351

[FL|F] -69.43 0.00135

[KO|K] -69.43 0.001347

[L|E] -69.43 0.001347

[L|F] -69.43 0.001347

[F|FI] -69.43 0.001346

[L|O] -69.43 0.001346

[L|N] -69.43 0.001346

[JN|N] -69.44 0.001343

[F|FL] -69.44 0.001342

[F|FJ] -69.44 0.001339

[E|EF] -69.44 0.001336

[E|EO] -69.44 0.001335

[E|EN] -69.44 0.001335

[J|CJ] -69.44 0.001331

[J|AJ] -69.44 0.001331

[J|BJ] -69.44 0.001331

[E|DE] -69.45 0.001328

[F|EF] -69.45 0.001324

[F|FO] -69.45 0.001323

[F|FN] -69.45 0.001323

[DN|D] -69.45 0.001321

[F|DF] -69.46 0.001316

[L|D] -69.47 0.001301

[KO|O] -69.47 0.001298

[CK|K] -69.48 0.001287

[EK|K] -69.48 0.001284

[DO|O] -69.48 0.001283

[JO|J] -69.48 0.001283

[L|KL] -69.48 0.001281

[FK|K] -69.49 0.00127

[AK|K] -69.5 0.001263

[BK|K] -69.5 0.001263

[JO|O] -69.5 0.001261

[D|K] -69.5 0.00126

[KL|K] -69.5 0.001253

[L|JL] -69.51 0.00125

[K|L] -69.51 0.001247

[K|J] -69.51 0.001245

[DO|D] -69.51 0.001241

[J|K] -69.52 0.001238

[EK|E] -69.52 0.001238

[L|EL] -69.52 0.001237

[L|FL] -69.52 0.001237

[D|L] -69.52 0.001237

[L|LO] -69.52 0.001235

[L|LN] -69.52 0.001235

[D|J] -69.52 0.001234

[KL|L] -69.52 0.00123

[L|DL] -69.52 0.001229

[CJ|J] -69.53 0.001227

[DE|E] -69.53 0.001226

[K|E] -69.53 0.001225

[K|F] -69.53 0.001225

[FK|F] -69.53 0.001225

[K|O] -69.53 0.001224

[K|N] -69.53 0.001224

[EJ|J] -69.53 0.001224

[J|L] -69.54 0.001215

[D|E] -69.54 0.001214

[D|F] -69.54 0.001214

[DF|F] -69.54 0.001214

[D|O] -69.54 0.001213

[D|N] -69.54 0.001213

[DL|L] -69.54 0.001211

At node N20:

split lnL Rel.Prob

[I|GI] -64.92 0.1227

[IN|I] -65.71 0.05548

[I|G] -66.18 0.03491

[IO|I] -66.18 0.03486

[CI|I] -66.69 0.02094

[I|I] -66.83 0.0182

[EI|I] -66.84 0.01803

[BI|I] -67 0.01531

[AI|I] -67 0.01531

[FI|I] -67.02 0.01506

[GI|G] -67.09 0.01405

[IN|N] -67.17 0.013

[N|G] -67.27 0.01174

[G|GL] -67.3 0.01133

[I|EI] -67.37 0.01058

[I|FI] -67.37 0.01058

[N|L] -67.41 0.01019

[I|CI] -67.44 0.009844

[I|BI] -67.44 0.009844

[I|AI] -67.44 0.009844

[G|GI] -67.46 0.009641

[GI|I] -67.54 0.008964

[I|IO] -67.56 0.008803

[I|IN] -67.56 0.008803

[HI|I] -67.57 0.008643

[IO|O] -67.63 0.008168

[O|G] -67.67 0.007871

[I|HI] -67.75 0.007242

[N|I] -67.77 0.007132

[O|L] -67.81 0.006832

[H|I] -67.88 0.006377

[L|GL] -67.9 0.006214

[I|C] -68.01 0.005573

[I|B] -68.01 0.005573

[I|A] -68.01 0.005573

[I|F] -68.05 0.005377

[I|E] -68.05 0.005377

[CI|C] -68.06 0.005313

[N|GN] -68.07 0.005288

[C|G] -68.08 0.005229

[N|LN] -68.12 0.005031

[I|H] -68.12 0.005029

[I|O] -68.13 0.004955

[I|N] -68.13 0.004955

[E|G] -68.16 0.00483

[O|I] -68.17 0.004783

[EI|E] -68.21 0.004585

[C|L] -68.22 0.004547

[GN|G] -68.28 0.004265

[F|G] -68.28 0.004252

[E|L] -68.3 0.004192

[A|G] -68.3 0.004163

[B|G] -68.3 0.004163

[BI|B] -68.37 0.003883

[AI|A] -68.37 0.003883

[FI|F] -68.39 0.00383

[F|L] -68.42 0.003691

[A|L] -68.44 0.00362

[B|L] -68.44 0.00362

[N|K] -68.45 0.003588

[O|GO] -68.46 0.003547

[O|LO] -68.51 0.003374

[LN|L] -68.57 0.003187

[C|I] -68.57 0.003183

[GO|G] -68.64 0.002971

[N|IN] -68.64 0.002969

[E|I] -68.65 0.002935

[K|KL] -68.71 0.002782

[F|I] -68.78 0.002584

[B|I] -68.8 0.002534

[A|I] -68.8 0.002534

[G|L] -68.81 0.0025

[O|K] -68.85 0.002406

[C|CG] -68.85 0.002401

[L|KL] -68.86 0.002399

[G|G] -68.86 0.002386

[H|HI] -68.88 0.002341

[D|G] -68.88 0.002338

[C|CL] -68.9 0.002301

[E|EG] -68.91 0.002269

[HI|H] -68.94 0.002203

[E|EL] -68.94 0.002194

[LO|L] -68.96 0.002167

[CG|G] -68.98 0.002118

[DG|G] -69.02 0.002043

[D|L] -69.02 0.002028

[EG|G] -69.03 0.002016

[F|FG] -69.04 0.001998

[O|IO] -69.04 0.001991

[F|FL] -69.07 0.001932

[B|BG] -69.08 0.001912

[A|AG] -69.08 0.001912

[N|D] -69.09 0.001893

[N|C] -69.1 0.001873

[N|A] -69.1 0.001873

[N|B] -69.1 0.001873

[N|J] -69.12 0.001849

[A|AL] -69.13 0.001832

[B|BL] -69.13 0.001832

[FG|G] -69.14 0.001808

[N|E] -69.14 0.001807

[N|F] -69.14 0.001807

[J|G] -69.17 0.001752

[BG|G] -69.17 0.001752

[AG|G] -69.17 0.001752

[G|I] -69.17 0.00175

[K|G] -69.17 0.001744

[G|GK] -69.21 0.001691

[N|O] -69.22 0.001665

[G|GM] -69.23 0.001656

[C|K] -69.26 0.001601

[G|GJ] -69.3 0.001537

[N|KN] -69.3 0.001534

[J|L] -69.31 0.001521

[K|L] -69.32 0.001514

[L|G] -69.32 0.001503

[CL|L] -69.33 0.00149

[E|K] -69.34 0.001476

[E|EI] -69.35 0.001469

[N|N] -69.36 0.001442

[C|CI] -69.38 0.001426

[EL|L] -69.38 0.001423

[C|M] -69.45 0.001327

[G|CG] -69.47 0.001302

[G|BG] -69.47 0.001302

[G|AG] -69.47 0.001302

[F|K] -69.47 0.0013

[F|FI] -69.47 0.001293

[G|FG] -69.48 0.001285

[G|EG] -69.48 0.001285

[A|K] -69.49 0.001275

[B|K] -69.49 0.001275

[M|G] -69.49 0.001273

[O|D] -69.49 0.001269

[G|DG] -69.49 0.001269

[FL|L] -69.5 0.001257

[O|C] -69.5 0.001256

[O|B] -69.5 0.001256

[O|A] -69.5 0.001256

[O|J] -69.52 0.00124

[H|H] -69.52 0.001237

[G|GO] -69.52 0.001232

[G|GN] -69.52 0.001232

[O|F] -69.54 0.001212

[O|E] -69.54 0.001212

[KN|K] -69.54 0.001208

[AL|L] -69.55 0.0012

[BL|L] -69.55 0.0012

[GJ|G] -69.59 0.001147

[B|BI] -69.6 0.001136

[A|AI] -69.6 0.001136

[GK|G] -69.61 0.001132

[L|L] -69.61 0.001127

[O|N] -69.62 0.001117

[D|DG] -69.64 0.001097

[K|GK] -69.66 0.001076

[B|M] -69.68 0.001057

[A|M] -69.68 0.001057

[O|KO] -69.7 0.001029

[DL|L] -69.72 0.001015

[D|DL] -69.73 0.001003

[J|GJ] -69.75 0.000983

[O|O] -69.76 0.0009672

[J|JL] -69.79 0.000945

[GM|G] -69.83 0.0009069

[G|K] -69.86 0.0008802

[C|CM] -69.88 0.0008598

[GL|G] -69.89 0.0008563

[KO|K] -69.9 0.0008435

[C|A] -69.91 0.000835

[C|B] -69.91 0.000835

[NO|O] -69.92 0.00083

[NO|N] -69.92 0.00083

[C|J] -69.92 0.0008252

[L|JL] -69.94 0.0008104

[C|F] -69.95 0.0008067

[C|E] -69.95 0.0008067

[CN|C] -69.96 0.0007948

[GL|L] -69.98 0.0007813

[E|D] -69.98 0.0007789

[N|JN] -69.98 0.0007787

[E|C] -69.99 0.0007709

[E|A] -69.99 0.0007709

[E|B] -69.99 0.0007709

[N|CN] -69.99 0.0007708

[N|AN] -69.99 0.0007708

[N|BN] -69.99 0.0007708

[DN|D] -69.99 0.0007704

[N|FN] -69.99 0.0007678

[N|EN] -69.99 0.0007678

[EN|E] -70 0.0007662

[E|J] -70 0.0007609

[N|DN] -70.01 0.0007573

[E|F] -70.03 0.0007438

[C|O] -70.03 0.0007434

[C|N] -70.03 0.0007434

[AN|A] -70.04 0.0007356

[BN|B] -70.04 0.0007356

[CN|N] -70.04 0.000734

[FN|F] -70.04 0.0007331

[G|M] -70.05 0.0007282

[D|K] -70.07 0.0007142

[C|CK] -70.08 0.0007067

[EN|N] -70.08 0.0007061

[C|C] -70.08 0.0007047

[N|NO] -70.09 0.0006974

[F|D] -70.11 0.0006857

[E|O] -70.11 0.0006854

[E|N] -70.11 0.0006854

[B|BM] -70.11 0.0006846

[A|AM] -70.11 0.0006846

[L|CL] -70.11 0.0006843

[L|AL] -70.11 0.0006843

[L|BL] -70.11 0.0006843

[L|FL] -70.11 0.0006814

[L|EL] -70.11 0.0006814

[BN|N] -70.12 0.0006794

[AN|N] -70.12 0.0006794

[F|C] -70.12 0.0006787

[F|A] -70.12 0.0006787

[F|B] -70.12 0.0006787

[FN|N] -70.12 0.0006756

[DN|N] -70.13 0.0006702

[F|J] -70.13 0.0006699

[JL|L] -70.13 0.0006683

[E|EK] -70.14 0.0006654

[M|GM] -70.14 0.0006648

[A|C] -70.14 0.0006648

[B|C] -70.14 0.0006648

[B|A] -70.14 0.0006648

[A|B] -70.14 0.0006648

[KL|L] -70.15 0.0006603

[A|J] -70.15 0.000657

[B|J] -70.15 0.000657

[F|E] -70.15 0.0006548

[E|E] -70.17 0.000644

[L|LO] -70.17 0.0006428

[L|LN] -70.17 0.0006428

[A|F] -70.17 0.0006422

[A|E] -70.17 0.0006422

[B|F] -70.17 0.0006422

[B|E] -70.17 0.0006422

[GN|N] -70.18 0.0006376

[L|DL] -70.18 0.0006366

[JN|J] -70.2 0.0006237

[CK|K] -70.23 0.0006071

[F|O] -70.24 0.0006034

[F|N] -70.24 0.0006034

[CO|C] -70.25 0.0005955

[B|O] -70.26 0.0005919

[A|O] -70.26 0.0005919

[B|N] -70.26 0.0005919

[A|N] -70.26 0.0005919

[F|FK] -70.27 0.0005858

[EK|K] -70.27 0.0005812

[EO|E] -70.29 0.0005721

[F|F] -70.3 0.000567

[JN|N] -70.31 0.000563

[A|AK] -70.31 0.0005626

[B|BK] -70.31 0.0005626

[KN|N] -70.31 0.0005624

[A|A] -70.31 0.0005611

[B|B] -70.31 0.0005611

[DO|D] -70.32 0.0005557

[CO|O] -70.33 0.00055

[FO|F] -70.34 0.0005413

[BO|B] -70.35 0.0005398

[AO|A] -70.35 0.0005398

[J|K] -70.35 0.0005356

[EO|O] -70.37 0.0005272

[O|JO] -70.38 0.0005222

[LN|N] -70.38 0.0005221

[FK|K] -70.38 0.0005219

[O|CO] -70.39 0.000517

[O|BO] -70.39 0.000517

[O|AO] -70.39 0.000517

[O|FO] -70.39 0.000515

[O|EO] -70.39 0.000515

[O|DO] -70.41 0.0005079

[AK|K] -70.42 0.0005028

[BK|K] -70.42 0.0005028

[FO|O] -70.43 0.0004989

[BO|O] -70.43 0.0004985

[AO|O] -70.43 0.0004985

[DO|O] -70.46 0.0004834

[O|NO] -70.49 0.0004677

[G|D] -70.5 0.0004647

[K|K] -70.51 0.0004601

[L|K] -70.51 0.0004594

[G|C] -70.51 0.0004588

[G|B] -70.51 0.0004588

[G|A] -70.51 0.0004588

[G|J] -70.52 0.0004537

[CE|E] -70.52 0.0004519

[CE|C] -70.53 0.0004509

[DK|K] -70.54 0.0004452

[GO|O] -70.54 0.0004441

At node N12:

split lnL Rel.Prob

[N|I] -64.71 0.1509

[N|IN] -64.92 0.1225

[O|I] -65.26 0.08728

[O|IO] -65.47 0.07083

[IN|I] -65.79 0.0514

[C|I] -65.98 0.04243

[E|I] -66.16 0.0354

[C|CI] -66.19 0.03444

[E|EI] -66.37 0.02874

[IO|I] -66.43 0.02702

[F|I] -66.48 0.02592

[A|I] -66.51 0.02498

[B|I] -66.51 0.02498

[F|FI] -66.68 0.02105

[B|BI] -66.72 0.02027

[A|AI] -66.72 0.02027

[I|I] -67.16 0.01314

[CI|I] -67.29 0.01145

[I|HI] -67.36 0.01068

[I|H] -67.52 0.009135

[EI|I] -67.66 0.007969

[DN|N] -67.85 0.006589

[G|I] -67.94 0.005965

[AI|I] -68.08 0.005223

[BI|I] -68.08 0.005223

[G|GI] -68.15 0.004846

[FI|I] -68.17 0.004751

[DO|O] -68.54 0.003279

[D|O] -68.64 0.002977

[D|N] -68.64 0.002977

[N|F] -68.72 0.00276

[N|E] -68.72 0.00276

[K|G] -68.99 0.002099

[D|G] -69.04 0.002002

[N|C] -69.1 0.001879

[N|A] -69.1 0.001879

[N|B] -69.1 0.001879

[HI|I] -69.21 0.001678

[O|F] -69.26 0.001596

[O|E] -69.26 0.001596

[HI|H] -69.35 0.00147

[NO|O] -69.37 0.001437

[NO|N] -69.37 0.001437

[EN|E] -69.39 0.001409

[CN|C] -69.41 0.001376

[N|O] -69.44 0.001331

[N|N] -69.45 0.001327

[N|FN] -69.59 0.001154

[N|EN] -69.59 0.001154

[D|K] -69.6 0.001141

[O|C] -69.65 0.001087

[O|A] -69.65 0.001087

[O|B] -69.65 0.001087

[CN|N] -69.66 0.001074

[DN|D] -69.68 0.001051

[FN|F] -69.69 0.001043

[GI|I] -69.69 0.001042

[I|GI] -69.69 0.001041

[N|G] -69.78 0.0009494

[D|J] -69.82 0.0009111

[N|CN] -69.88 0.0008637

[N|BN] -69.88 0.0008637

[N|AN] -69.88 0.0008637

[H|HI] -69.88 0.0008587

[H|I] -69.91 0.0008378

[EN|N] -69.91 0.0008374

[D|DO] -69.92 0.000825

[D|DN] -69.92 0.000825

[CO|C] -69.97 0.000788

[AN|A] -69.98 0.0007817

[BN|B] -69.98 0.0007817

[O|N] -69.99 0.0007697

[O|O] -70 0.0007675

[EO|E] -70.09 0.0006957

[E|E] -70.12 0.0006796

[N|NO] -70.13 0.0006726

[KN|N] -70.13 0.0006702

[O|FO] -70.14 0.0006673

[O|EO] -70.14 0.0006673

[DG|G] -70.14 0.0006636

[I|CI] -70.14 0.0006612

[I|BI] -70.14 0.0006612

[I|AI] -70.14 0.0006612

[I|FI] -70.16 0.0006534

[I|EI] -70.16 0.0006534

[D|E] -70.17 0.0006475

[D|F] -70.17 0.0006475

[JN|N] -70.17 0.0006426

[I|IO] -70.18 0.0006408

[I|IN] -70.18 0.0006408

[J|F] -70.19 0.0006336

[J|E] -70.19 0.0006336

[FN|N] -70.21 0.0006196

At node N10:

split lnL Rel.Prob

[N|D] -64.04 0.2967

[O|D] -64.92 0.1232

[N|DN] -65.17 0.09592

[CN|N] -65.66 0.05871

[NO|O] -66.01 0.04146

[NO|N] -66.01 0.04146

[O|DO] -66.05 0.03983

[CO|O] -66.48 0.02584

[EN|N] -66.49 0.02556

[DN|D] -66.62 0.02246

[N|O] -66.89 0.01711

[N|N] -66.89 0.01711

[N|J] -67.26 0.01186

[N|K] -67.26 0.01184

[C|O] -67.44 0.009866

[C|N] -67.44 0.009866

[AO|O] -67.65 0.008013

[BO|O] -67.65 0.008013

[AN|N] -67.69 0.007687

[BN|N] -67.69 0.007687

[C|J] -67.76 0.007208

[O|N] -67.77 0.007106

[O|O] -67.77 0.007105

[C|K] -67.77 0.007092

[O|J] -68.14 0.004923

[O|K] -68.14 0.004915

[E|O] -68.46 0.003574

[E|N] -68.46 0.003574

[E|J] -68.72 0.002744

[E|K] -68.75 0.002665

[DO|D] -68.79 0.002565

[A|O] -69.04 0.001988

[B|O] -69.04 0.001988

[B|N] -69.04 0.001988

[A|N] -69.04 0.001988

[K|D] -69.1 0.001872

[FN|N] -69.18 0.001732

[J|D] -69.18 0.001731

[EO|O] -69.19 0.001722

[N|NO] -69.19 0.001712

At node N8:

split lnL Rel.Prob

[N|NO] -64.35 0.2164

[N|N] -64.56 0.1765

[CN|N] -64.72 0.15

[C|O] -65.28 0.08559

[N|O] -65.72 0.05517

[N|E] -65.9 0.04612

[C|N] -65.9 0.04606

[N|EN] -66.15 0.03573

[B|O] -66.48 0.02589

[A|O] -66.48 0.02589

[B|N] -67.1 0.01393

[A|N] -67.1 0.01393

[N|DN] -67.15 0.01323

[BN|N] -67.74 0.007308

[AN|N] -67.74 0.007308

[O|O] -68.08 0.005224

[C|CN] -68.09 0.005158

[O|EO] -68.46 0.003572

[O|NO] -68.49 0.003447

[DN|N] -68.53 0.003326

[NO|O] -68.63 0.003019

[CO|O] -68.76 0.002647

[CN|C] -68.76 0.002641

[C|CO] -68.78 0.002577

At node N4:

split lnL Rel.Prob

[N|C] -63.18 0.6998

[B|C] -65.35 0.07996

[A|C] -65.35 0.07996

[N|CN] -66.28 0.03139

[AN|A] -67.01 0.01515

[BN|B] -67.01 0.01515

[AN|N] -67.16 0.0131

[BN|N] -67.16 0.0131

[CN|C] -67.67 0.007879

At node N2:

split lnL Rel.Prob

[A|N] -63.53 0.4916

[B|N] -63.53 0.4916

At node N7:

split lnL Rel.Prob

[N|O] -63.47 0.5249

[E|O] -64.73 0.1489

[N|NO] -65.53 0.06662

[EN|E] -65.65 0.05939

[EN|N] -66 0.04178

[N|N] -66.4 0.02794

[E|EO] -66.79 0.0189

[N|D] -66.99 0.0155

[NO|O] -67.14 0.01337

[N|C] -67.25 0.01199

[E|E] -67.48 0.009483

[N|B] -67.79 0.006975

[N|A] -67.79 0.006975

At node N19:

split lnL Rel.Prob

[GI|G] -63.61 0.4554

[G|L] -64.86 0.1298

[G|GL] -65.69 0.05692

[K|KL] -65.73 0.05444

[G|K] -65.83 0.04964

[GL|L] -66.47 0.02594

[G|GK] -66.62 0.0224

[G|G] -66.87 0.01741

[GK|K] -67.16 0.01309

[L|L] -67.25 0.01191

[I|E] -67.78 0.007015

[I|F] -67.79 0.006949

[I|G] -67.85 0.006553

[EI|E] -68.03 0.005453

[FI|F] -68.04 0.005402

[I|C] -68.14 0.004923

[I|B] -68.15 0.00484

[I|A] -68.15 0.00484

[I|N] -68.33 0.004047

[I|O] -68.39 0.003823

[G|J] -68.42 0.003709

[GK|G] -68.45 0.003594

[GM|G] -68.47 0.003539

[K|L] -68.47 0.003533

[G|GM] -68.49 0.003473

[J|JL] -68.59 0.003137

[L|JL] -68.59 0.003136

[GI|I] -68.59 0.003126

[CI|C] -68.6 0.003084

[BI|B] -68.62 0.003032

[AI|A] -68.62 0.003032

[L|KL] -68.69 0.002844

[IN|N] -69 0.002068

[IO|O] -69.06 0.001954

[G|M] -69.07 0.001926

[M|G] -69.14 0.001812

[G|GJ] -69.24 0.001631

[I|EI] -69.41 0.001377

[I|FI] -69.42 0.001364

[I|GI] -69.48 0.001282

[E|L] -69.51 0.001244

[K|JK] -69.52 0.001232

[F|L] -69.54 0.001216

[G|E] -69.56 0.001191

[G|F] -69.58 0.001163

[K|J] -69.73 0.0009997

At node N15:

split lnL Rel.Prob

[G|I] -63.15 0.7199

[GI|I] -65.15 0.0974

[G|GI] -65.67 0.05803

[GK|G] -66.24 0.03295

[G|G] -67.1 0.01383

[I|H] -67.32 0.01113

[E|I] -67.92 0.006091

[F|I] -67.93 0.006028

[I|I] -68.25 0.004391

[I|HI] -68.38 0.003871

At node N18:

split lnL Rel.Prob

[K|L] -63.89 0.3449

[J|L] -64.83 0.1337

[K|G] -64.94 0.1207

[G|L] -64.98 0.1151

[K|KL] -66.23 0.03328

[KL|L] -66.41 0.02779

[JK|J] -66.66 0.02164

[JK|K] -66.73 0.02002

[L|L] -67.02 0.01507

[J|JL] -67.17 0.0129

[G|GL] -67.32 0.01111

[JL|L] -67.37 0.0106

[GK|G] -67.45 0.009788

[K|GK] -67.89 0.006316

[K|K] -67.92 0.006104

[E|L] -67.98 0.005776

[F|L] -67.98 0.00574

[K|E] -68.05 0.005361

[K|F] -68.05 0.005344

[K|D] -68.35 0.003988

[GK|K] -68.36 0.003947

[C|L] -68.39 0.003826

[A|L] -68.4 0.003792

[B|L] -68.4 0.003792

[K|C] -68.4 0.003788

[K|A] -68.4 0.003773

[K|B] -68.4 0.003773

[GL|L] -68.42 0.003726

[D|L] -68.55 0.003248

[K|N] -68.74 0.002689

***END OF LAGRANGE ANCESTRAL HOST RECONSTRUCTION RESULTS***
